# Characterization of the angiomodulatory effects of Interleukin 11 cis- and trans-signaling in the retina

**DOI:** 10.1186/s12974-024-03223-3

**Published:** 2024-09-18

**Authors:** Paula Liang, Jan Ness, Julian Rapp, Stefaniya Boneva, Melanie Schwämmle, Malte Jung, Günther Schlunck, Hansjürgen Agostini, Felicitas Bucher

**Affiliations:** 1 Eye Center, Medical Center, Faculty of Medicine, University of Freiburg, Klinik für Augenheilkunde, Kilianstrasse 5, 79106 Freiburg im Breisgau, Germany; 2 Institute of Pharmaceutical Sciences, Faculty of Chemistry and Pharmacy, University of Freiburg, Freiburg, Germany; 3 Department of Medicine I, Medical Center - Faculty of Medicine, University of Freiburg, Freiburg, Germany; 4 Faculty of Biology, University of Freiburg, Freiburg, Germany

**Keywords:** Angiogenesis, Endothelial cell, Diabetic retinopathy, STAT3, IL-6 family cytokines, Interleukin 11, Trans-signaling, Oxygen in retinopathy, Müller cell

## Abstract

**Background:**

The IL-6 cytokine family, with its crucial and pleiotropic intracellular signaling pathway STAT3, is a promising target for treating vasoproliferative retinal diseases. Previous research has shown that IL-6 cis-signaling (via membrane-bound receptors) and trans-signaling (via soluble receptors) can have distinct effects on target cells, leading to their application in various disease treatments. While IL-6 has been extensively studied, less is known about the angiogenic effects of IL-11, another member of the IL-6 family, in the retina. Therefore, the aim of this study was to characterize the effects of IL-11 on retinal angiogenesis.

**Main text:**

In vitreous samples from proliferative diabetic retinopathy (PDR) patients, elevated levels of IL-11Rα, but not IL-11, were detected. In vitro studies using vascular endothelial cells revealed distinct effects of cis- and trans-signaling: cis-signaling (IL-11 alone) had antiangiogenic effects, while trans-signaling (IL-11 + sIL-11Rα) had proangiogenic and pro-migratory effects. These differences can be attributed to their individual signaling responses and associated transcriptomic changes. Notably, no differences in cis- and trans-signaling were detected in primary mouse Müller cell cultures. STAT3 and STAT1 siRNA knockdown experiments revealed opposing effects on IL-11 signaling, with STAT3 functioning as an antiproliferative and proapoptotic player while STAT1 acts in opposition to STAT3. In vivo, both IL-11 and IL-11 + sIL-11Rα led to a reduction in retinal neovascularization. Immunohistochemical staining revealed Müller cell activation in response to treatment, suggesting that IL-11 affects multiple retinal cell types in vivo beyond vascular endothelial cells.

**Conclusions:**

Cis- and trans-signaling by IL-11 have contrasting angiomodulatory effects on endothelial cells in vitro. In vivo, cis- and trans-signaling also influence Müller cells, ultimately determining the overall angiomodulatory impact on the retina, highlighting the intricate interplay between vascular and glial cells in the retina.

**Supplementary Information:**

The online version contains supplementary material available at 10.1186/s12974-024-03223-3.

## Introduction

Pathological new vessel formation is a fundamental component of tumorigenesis and the progression of vasoproliferative eye diseases such as proliferative diabetic retinopathy (PDR). As one of the most common vasoproliferative diseases in the eye and a major cause of vision loss in the Western world [1], DR, which is driven by a shift to proangiogenic cytokines, often progresses to a proliferative state (PDR) when untreated [2, 3]. Current therapeutic options for PDR, including the intravitreal injections of anti-vascular endothelial growth factor antibodies (anti-VEGF-A), may not fully halt disease progression in all patients [4, 5] suggesting that alternative therapeutic approaches are needed.

Recent evidence suggests that diabetes impacts the retina beyond vascular changes. Chronic inflammation and the inflammation-associated interleukin 6 (IL-6) cytokine family may also contribute to vasoproliferative disease progression [2, 6]. IL-6 has been shown to be activated in areas of retinal neovascularization [7, 8] and is significantly upregulated in the vitreous of PDR patients [9]. The IL-6 family includes multiple pleiotropic cytokines like IL-6, interleukin 11 (IL-11), ciliary neurotrophic factor (CNTF) and oncostatin M (OSM), all utilizing glycoprotein 130 (gp130) as a receptor component and STAT3 as a crucial intracellular signaling pathway. Previous studies showed that STAT3 concentrations were significantly higher in serum samples from patients with DR and PDR. The IL-6/ STAT3 axis can impair the blood-retinal barrier and activate microglia which are the key inflammatory mediators in DR [10, 11]. However, although all IL-6 family members share the STAT3 signaling pathway, their effects on vascular endothelial cells are not fully understood due to their unique specific receptor subunits resulting in diverse and context-dependent effects [12]. For instance, CNTF, expressed in astrocytes [13] and Müller cells (data not shown) in the eye, has an anti-angiogenic effect on vascular endothelial cells [14]. In contrast, OSM, primarily produced by immune system cells [15, 16], has a proangiogenic effect [17].

IL-11 is a less well-studied member of the IL-6 family. Discovered in 1990 as a hematopoietic cytokine, recent research has suggested that IL-11 also plays a role in inflammatory and autoimmune diseases [18] and fibrotic diseases and promotes tumorigenesis in multiple organs [19–25]. Its receptor, the IL-11 receptor (IL-11R) complex, consists of the α-subunit IL-11Rα and the common glycoprotein130 (gp130) receptor [26, 27]. IL-11, along with IL-6, is the only cytokine of the IL-6-family that utilizes gp130 homodimers. There are two major signaling mechanisms for IL-11: cis-signaling, in which IL-11 binds to membrane-bound IL-11Rα, and trans-signaling, in which IL-11 binds to the soluble IL-11Rα subunit which in turn assembles gp130 homodimers that are solely responsible for intracellular signal transduction [28–31]. Unlike the ubiquitously expressed gp130 receptor, IL-11Rα is expressed only in specific cell types like endothelial cells, epithelial cells and fibroblasts [18]. Thus, cis-signaling is restricted to cells expressing IL-11R, while trans-signaling can activate a broader range of cells through the soluble receptor component [30].

The homodimerization of gp130 causes the phosphorylation of different Janus kinases (JAKs). Studies have shown that JAK1 is essential for the trans-signaling of IL-6 and IL-11 as their signaling is impaired in JAK1-negative cells [32–34]. However, it should also be noted that the activation of certain Janus kinases depends on the cell type [32].

Despite these findings the exact role of IL-11 cis- and trans-signaling in disease development, particularly in the eye and in retinal vasoproliferative disease, is largely unknown. A previously published study observed elevated levels of IL-11 in the vitreous of PDR patients and high concentrations of IL-11Rα in MIO-M1 Müller cells [35, 36]. The aim of the current study is to characterize the angiogenic effects of IL-11 cis- and trans-signaling in retinal angiogenesis, especially in the presence of VEGF, a key driver of retinal neovascular disease. We quantified IL-11 and IL-11Rα levels in the vitreous of PDR patients and tested IL-11 cis- and trans-signaling angiomodulatory effects in in vitro and in vivo angiogenesis models.

## Results

### IL-11Rα but not IL-11 is significantly increased in the vitreous of PDR patients

**Fig. 1:**
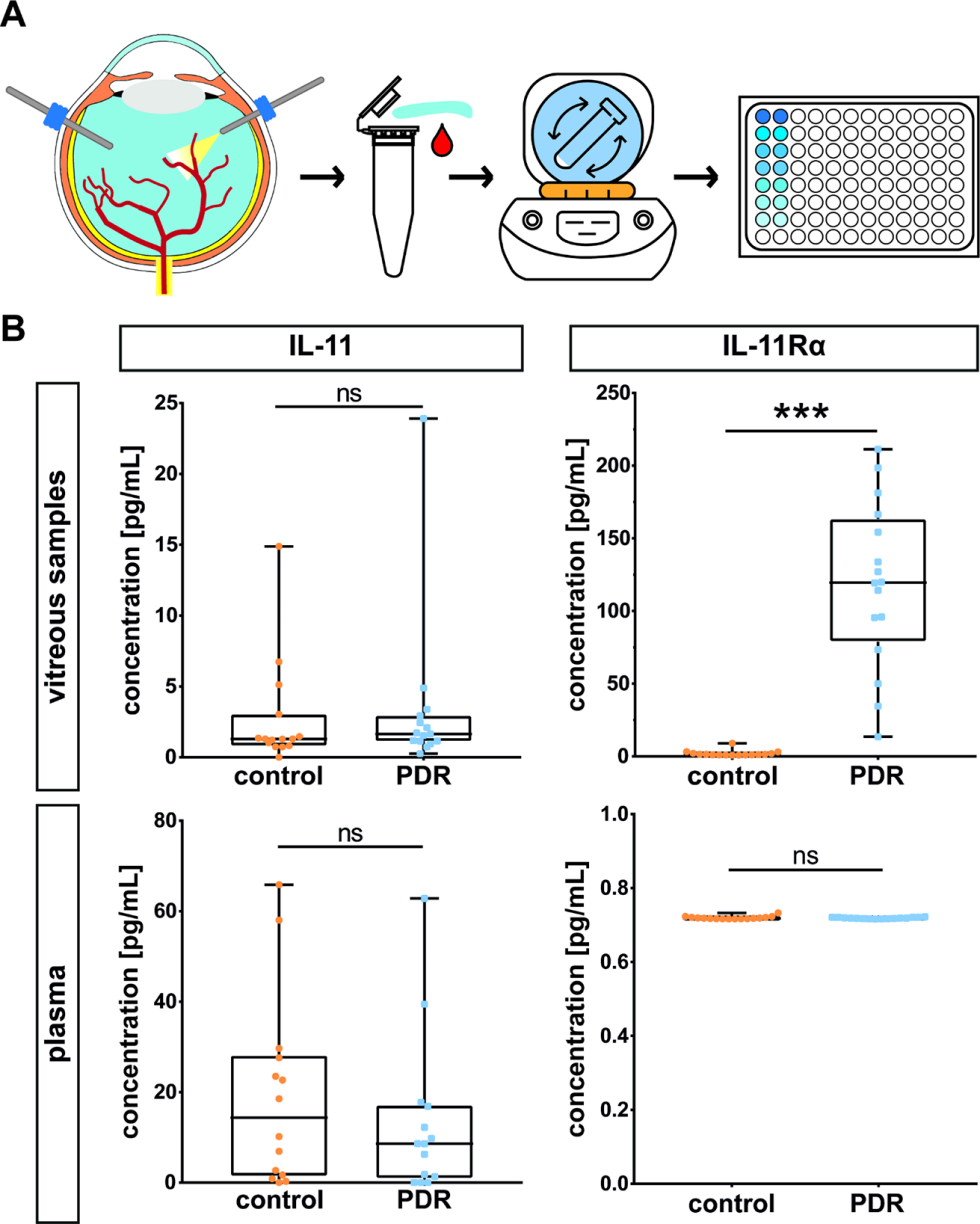
IL-11Rα is significantly increased in human vitreous samples. (**A**) Experimental setup: Following vitrectomy, undiluted vitreous and plasma samples were centrifuged and the supernatants were frozen until ELISA was performed. (**B**) Interleaved box and whiskers visualizing the protein levels of IL-11 (*N*=15) and IL-11Rα (*N*=16) in vitreous and corresponding plasma samples in control (macular pucker) and PDR (proliferative diabetic retinopathy) group, as measured by ELISA. The whiskers represent the minimum and maximum values while the line in between represents the median value. Statistical testing: Mann Whitney test. *p<0.001

To better understand the role of IL-11 in disease pathogenesis, we compared the concentrations of IL-11 in human vitreous and plasma samples from 15 PDR patients to those from 15 control patients with macular pucker (Fig. 1A + Table 1). A total of 16 patients per group were analyzed for IL-11Rα levels (Table 2).

Overall, IL-11 levels were lower in the vitreous than in the plasma and there was no significant difference in IL-11 concentrations between the PDR and control groups (Fig. 1B). In contrast, the vitreous levels of IL-11Rα were significantly greater in the PDR group than in the control group (Fig. 1B). Although some vitreous samples were contaminated with blood, parallel analysis of patient blood samples did not reflect the yields of expression of IL-11Ra as in the vitreous. Plasma levels of IL-11Rα remained similar between the control and PDR group (Fig. 1B).

**Table 1:** Clinical characteristics of patient samples analyzed by IL-11 ELISA

	PDR	Control
Number of patients	15	15
Mean Age (±SD)	62(±14)	71(±6)
Sex (n female, %)	6 (40%)	7 (47%)

A total of 15 samples were analyzed for each group

**Table 2:** Clinical characteristics of patient samples analyzed by IL-11Rα ELISA

	PDR	Control
Number of patients	16	16
Mean Age (±SD)	68(±9)	71(±7)
Sex (n female, %)	8 (50%)	8 (50%)

A total of 16 samples were analyzed for each group

### Distinct angio-modulatory effects of IL-11 cis- and trans-signaling in vitro

**Fig. 2:**
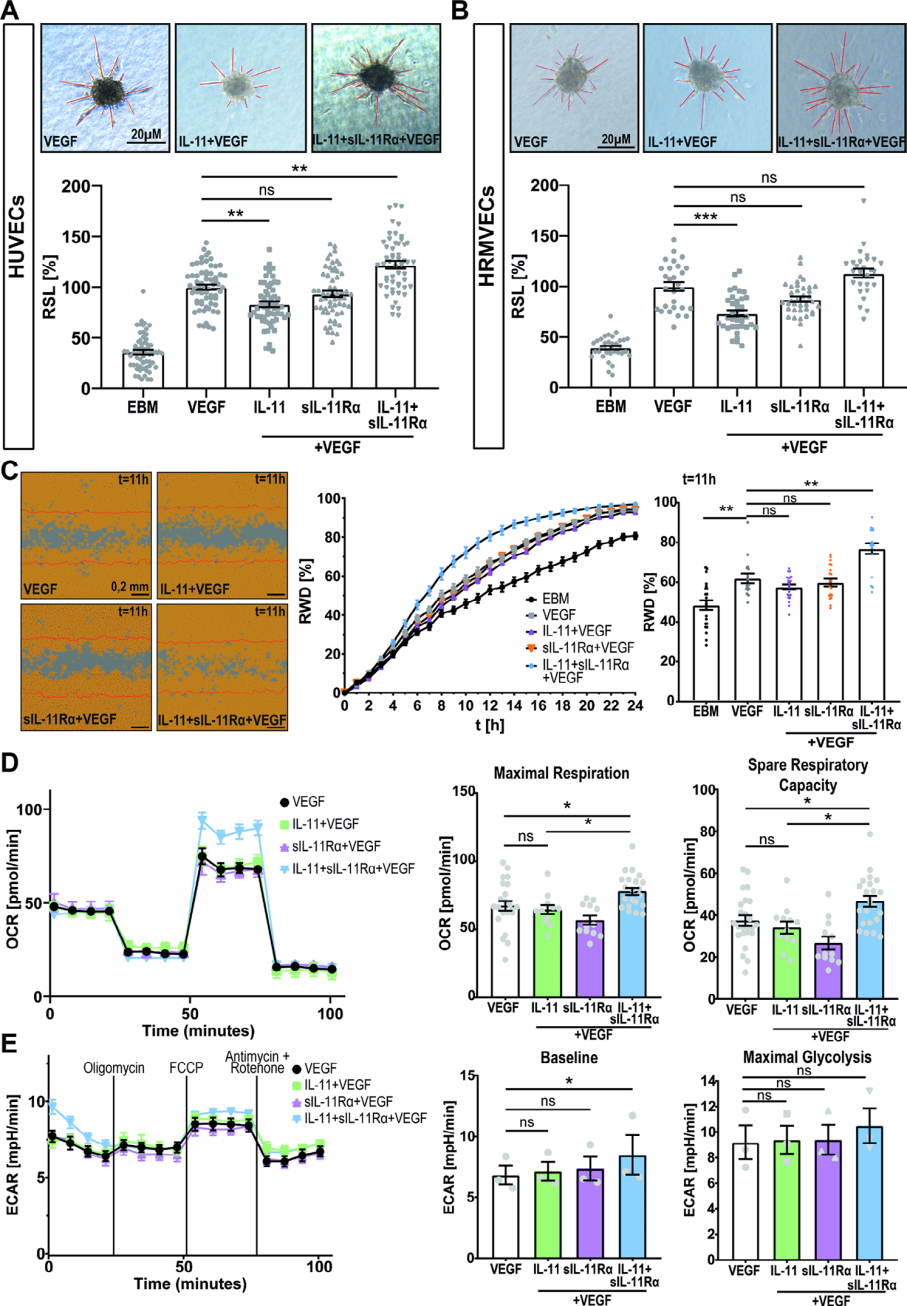
In the presence of VEGF, IL-11 cis-signaling has anti-angiogenic effects on vascular endothelial cells while trans-signaling enhances their pro-angiogenic and pro-migratory potential associated with an enhanced respiratory rate. (**A**) Spheroid sprouting assay with HUVECs exposed to endothelial basal medium (EBM = negative control), VEGF (positive control), IL-11 + VEGF or IL-11 + sIL-11Rα+ VEGF for 17 h. *N* = 3 independent experiments with 15-22 spheroids per group and experiment. Statistical test: Kruskal-Wallis Test adjusted for multiple testing, *p<0.01. Relative sprouting length (RSL), scale bar 20 µM. (**B**) Spheroid sprouting assay with HRMVECs exposed to VEGF (positive control), IL-11 + VEGF or IL-11 + sIL-11Rα + VEGF for 17 h. *N* = 2 independent experiments with 10-22 spheroids per group and experiment. Statistical test: Kruskal-Wallis Test adjusted for multiple testing, *p<0.01. Relative sprouting length (RSL), scale bar 20 µM. (**C**) Scratch wound assay using HUVECs: Migratory effect of IL-11 + sIL-11Rα + VEGF and IL-11 + VEGF on HUVECs over 24 h. *N* = 3 independent experiments each including 6-8 technical replicates. Relative Wound Density (RWD). Exemplary analysis of the conditions 11 h after stimulation, statistical test: Kruskal-Wallis Test adjusted for multiple testing, *p<0.01. Scale bar 200 µM. (**D**) Representative graph of the oxygen consumption rate (OCR) of HUVECs after pretreatment with above mentioned cytokines for 15 h. *N* = 3 with each 4-8 technical replicates. Statistical test: Kruskal-Wallis Test adjusted for multiple testing, *p<0.05. (**E**) Representative graph of the normalized extracellular acidification rate (ECAR) of HUVECs after pretreatment with above mentioned cytokines for 15 h. *N* = 3 with each 4-8 technical replicates. Statistical test: Friedman Test adjusted for multiple testing, *p<0.05

To elucidate the angio-modulatory potential of IL-11 cis- and trans-signaling, we then performed in vitro angiogenesis experiments using HUVECs and HRMVECs representative for macro- and microvascular endothelial cells. The optimal concentrations for IL-11 (100 ng/mL) and sIL-11Rα (400 ng/mL) were established through a dose-response analysis, ensuring maximal experimental sensitivity and reproducibility (Suppl. Fig. S1A). In the spheroid sprouting assay using HUVECs, stimulation with IL-11 (cis-signaling) significantly reduced VEGF-induced vascular endothelial cell sprouting (Fig. 2A) which was not mediated through an anti-apoptotic effect on cells (Suppl. Fig. S1B). In contrast, cells stimulated with IL-11 + sIL-11Rα (trans-signaling) showed significantly enhanced endothelial cell sprouting in the presence and absence of VEGF (Fig. 2A + Suppl. Fig. S2A). HRMVECs showed similar results (Fig. 2B + Suppl. Fig. S2B) despite not expressing IL-11Rα on RNA level (Suppl. Fig. S3). In the scratch wound assay, IL-11 + sIL-11Rα in the presence of VEGF increased vascular endothelial cell migration compared to VEGF treatment alone, while IL-11 + VEGF or sIL-11Rα + VEGF treatment did not significantly affect vascular endothelial cell migration (Fig. 2C + Suppl. Fig. S2C).

As enhanced cell migration and sprouting require increased energy levels, we next determined the effect of IL-11 cis- and trans-signaling on mitochondrial activity (Fig. 2D, E + Suppl. Fig. S2D, E). Seahorse experiments revealed a significant increase in maximal respiration and spare respiratory capacity in response to IL-11 + sIL-11Rα + VEGF compared to IL-11 + VEGF (Fig. 2D + Suppl. Fig. S2D) indicating a difference in vascular endothelial cell behavior between cis- and trans-signaling with trans-signaling increasing mitochondrial metabolism. Measurement of extracellular acidification rate (ECAR) revealed that the baseline glycolytic activity was significantly enhanced for the IL-11 + sIL-11Rα + VEGF group compared to VEGF. However, the maximal glycolytic capacity remained unchanged across all treatment groups likely due to higher variability between biological replicates compared to the OCR data (Fig. 2E + Suppl. Fig. S2E).

### IL-11 trans-signaling activates a broad range of intracellular signaling pathways whereas IL-11 cis-signaling specifically activates STAT3 signaling

**Fig. 3:**
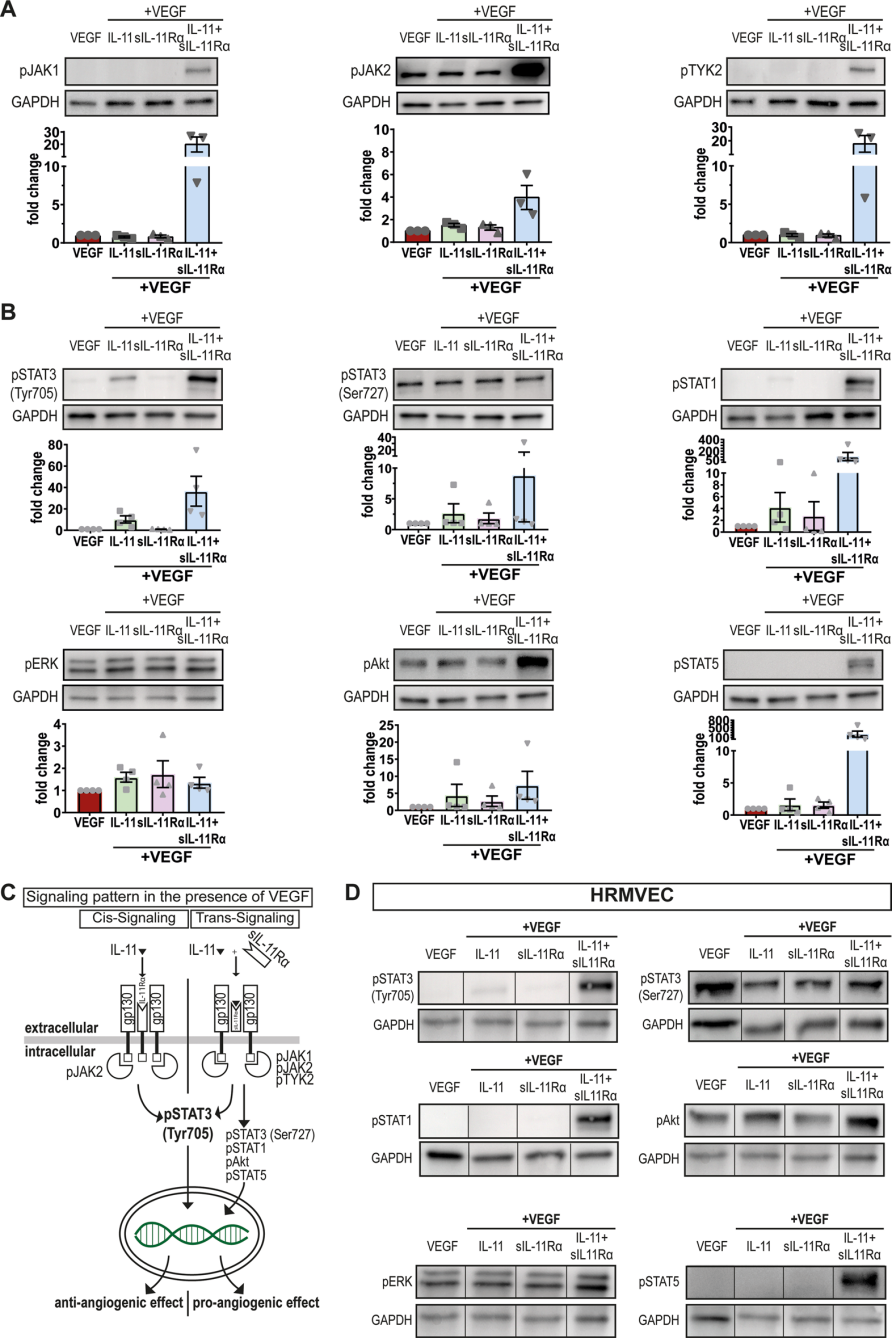
IL-11 trans-signaling activates intracellular signaling pathways beyond STAT3. (**A**) Scatter plots with bar visualizing the JAK activation in HUVECs after stimulation with VEGF, IL-11+VEGF, sIL-11Rα+VEGF or IL-11+sIL-11Rα+VEGF for 15 min. Representative images of *N* = 3 independent experiments. (**B**) Scatter plots with bar visualizing activated signaling pathways in HUVECs after stimulation with VEGF, IL-11+VEGF, sIL-11Rα+VEGF or IL-11+sIL-11Rα+VEGF for 15 min. Representative images of *N* = 4 independent experiments. (**C**) Graphical summary of the activated pathways by IL-11 or IL-11+sIL-11Rα and their angiogenic effects in HUVECs. (**D**) Western blot of activated signaling pathways in HRMVECs after stimulation with VEGF, IL-11+VEGF, sIL-11Rα+VEGF or IL-11+sIL-11Rα+VEGF for 15 min. *N* = 1 experiment

We next hypothesized that the observed divergent effects of IL-11 and IL-11 + sIL-11Rα on vascular endothelial cells can be explained by distinct intracellular signaling responses. In the classical JAK/ STAT signaling pathway, we first analyzed the Janus kinases (JAKs): IL-11 + VEGF primarily induced JAK2 phosphorylation while IL-11 + sIL-11Rα + VEGF additionally activated JAK1 and Tyk2 (Fig. 3A + Suppl. Fig. S2F). Further downstream in the signaling cascade, IL-11 + VEGF alone primarily induced the phosphorylation of STAT3 at the Tyr705 residue (Fig. 3B). Western blot analysis revealed that IL-11 + sIL-11Rα + VEGF had a stronger effect (Fig. 3B) and induced the phosphorylation of the STAT3 Ser727 site as well as other signaling pathways such as the STAT1, Akt and STAT5 pathways (Fig. 3B + C). Stimulation with sIL-11Rα alone did not appear to induce self-activation. The intracellular signaling pathways exhibited similar activation patterns in the group without VEGF stimulation (Suppl. Fig. S2G) as well as in HRMVECs (Fig. 3D + Suppl. Fig. S2H).

### The transcriptomic profile of IL-11 trans-signaling is distinct from that of IL-11 cis-signaling- and VEGF-induced transcriptomes

**Fig. 4:**
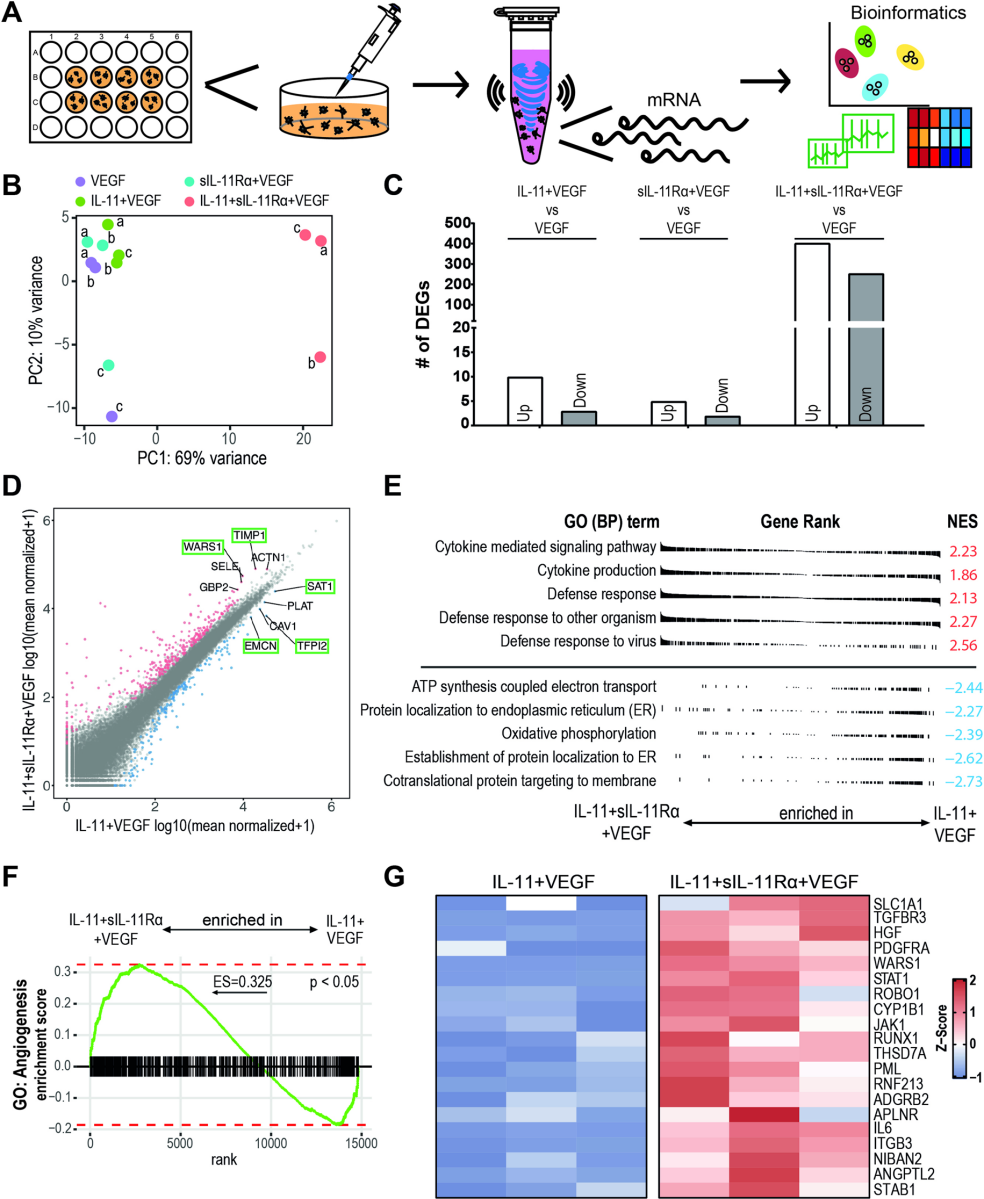
Transcriptional analysis of IL-11 + VEGF and IL-11 + sIL-11Rα + VEGF. (**A**) Experimental setup for RNA Seq analysis from 3D cell culture setting. *N* = 3 independent experiments using HUVECs with 14-22 spheroids per group and experiment. (**B**) PCA analysis of all samples. (**C**) Total number of up- and downregulated DEGs compared to VEGF (control). (**D**) Scatter blot visualizing all genes with at least one count when comparing IL-11 + sIL-11Rα + VEGF against IL-11 + VEGF. Differentially expressed genes (DEGs) were defined by padj <0.05 and abs(log2 foldchange) >1. The five most expressed up- and downregulated DEGs are labeled. All following analyzes were conducted by comparing IL-11 + sIL-11Rα + VEGF against IL-11 + VEGF. (**E**) The five most significantly enriched and depleted biological processes according to Gene Ontology (GO) terms. Positive normalized enrichment score (NES) refers to enrichment in the IL-11 + sIL-11Rα + VEGF treatment and negative NES to depletion and consequently enrichment in IL-11+VEGF. (**F**) Gene Set Enrichment Analysis (GSEA) for the GO term “angiogenesis” (GO:0048514). (**G**) Heatmap illustrating the 20 leading edge genes of the GSEA from F

To further elucidate the differences between IL-11 cis- and trans-signaling, an unsupervised analysis of transcriptomic changes in response to cytokine stimulation was performed. For this purpose, we used a novel technique to extract RNA from the 3D spheroid-sprouting assay (Fig. 4A) as described in our previous publication [37]. We performed a principal component analysis (PCA) to assess the variance between the experimental groups and biological replicates (Fig. 4B). In comparison to a stimulation with VEGF only, spheroids stimulated with IL-11 + VEGF and sIL-11Rα + VEGF showed only a small shift suggesting that the transcriptomes of these groups exhibit many similarities. In contrast, the IL-11 + sIL-11Rα + VEGF gene set was clearly distinct from those of all the other groups (Fig. 4B). Compared with the control group, the IL-11 + sIL-11Rα + VEGF group had 404 upregulated and 254 downregulated DEGs. In contrast, the comparison between IL-11 + VEGF and VEGF revealed 10 upregulated and 3 downregulated DEGs. For sIL-11Rα + VEGF compared to VEGF, only 5 DEGs were upregulated and 2 DEGs were downregulated (Fig. 4C).

To obtain a better insight into the differences between cis- and trans-signaling, we compared the group of spheroids stimulated with IL-11 + VEGF to the group stimulated with IL-11 + sIL-11Rα + VEGF. The scatter plot visualizes all genes with at least one count labeling the five most strongly expressed up- and downregulated DEGs. In the IL-11 + sIL-11Rα + VEGF group, the genes *WARS1* and *TIMP1* were the most strongly upregulated while *EMCN*, *SAT1* and *TFPI2* were the most strongly downregulated genes (Fig. 4D). Gene Ontology (GO) terms such as “cytokine-mediated signaling pathway”, “cytokine production” or “defense responses” were highly enriched in the transcriptome of IL-11 + sIL-11Rα + VEGF-treated spheroids (Fig. 4E). In contrast, IL-11 + VEGF treatment affected metabolic mechanisms such as “ATP synthesis coupled electron transport”, “protein localization” or “oxidative phosphorylation” (Fig. 4E). Based on the observed angiomodulatory effects of cis- and trans-signaling, a gene set enrichment analysis (GSEA) for the GO term “angiogenesis” was subsequently performed, revealing a significant enrichment of 0.325 (Fig. 4F). The 20 leading edge genes of the GSEA from Fig. 4F are displayed in a heatmap (Fig. 4G). A significant upregulation of proangiogenic genes such as *PDGFRA*, *APLNR*, *HGF*, *ANGPTL2* or *IL6* was observed in the group of IL-11 + sIL-11Rα + VEGF, consistent with the proangiogenic effect observed in the angiogenesis assays (Fig. 2A). Interestingly, pathway genes such as *JAK1* or *STAT1* were also upregulated in the IL-11 + sIL-11Rα + VEGF group which is consistent with the Western blot data showing that IL-11 + sIL-11Rα + VEGF activates both, the JAK1 and the STAT1 pathway (Fig. 3).

### A precise balance between the STAT3 and STAT1 pathways regulates angiogenesis

**Fig. 5:**
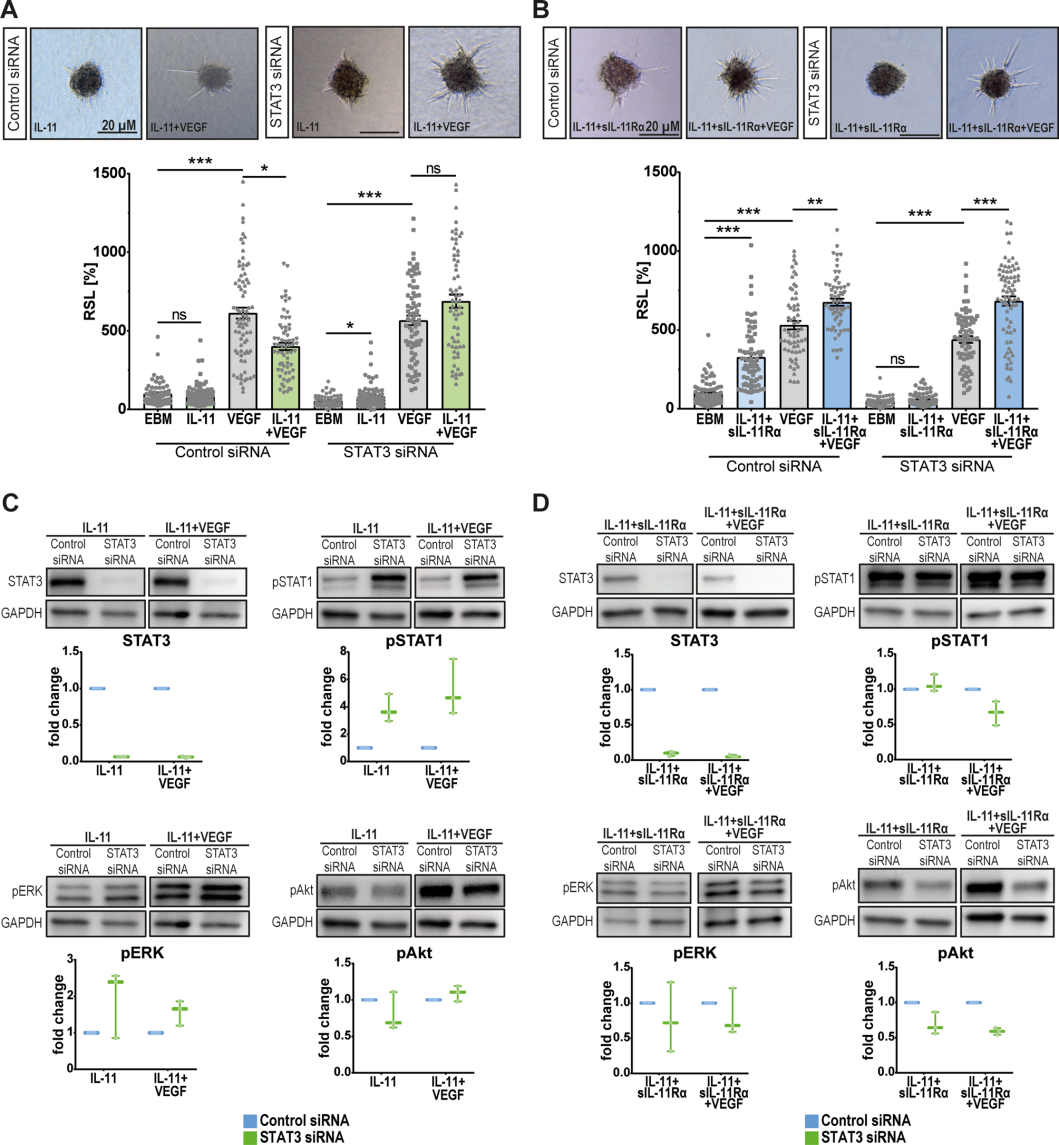
Angiogenic effects and altered signaling patterns of IL-11 cis- and trans-signaling following STAT3 knockdown. (**A**) Spheroid sprouting assay using HUVECs: Cells transfected with control siRNA or STAT3 siRNA were treated under following conditions: EBM (negative control), IL-11, VEGF (positive control), IL-11 + VEGF. RSL = Relative Sprouting Length. *N* = 4 independent experiments each consisting of 12-23 spheroids per group and experiment, statistical testing: Kruskal-Wallis Test adjusted for multiple testing, *p<0.05. (**B**) Spheroid sprouting assay using HUVECs: Cells transfected with control siRNA or STAT3 siRNA were treated under following conditions: EBM (negative control), IL-11 + sIL-11Rα, VEGF (positive control), IL-11 + sIL-11Rα + VEGF. RSL = Relative Sprouting Length. *N* = 4 independent experiments each consisting of 9-22 spheroids per group and experiment, statistical testing: Kruskal-Wallis Test adjusted for multiple testing, *p<0.01. (**C**) Semi-quantitative Western blot analysis of signaling molecules in HUVECs after STAT3 knockdown and treatment under the conditions mentioned in (**A**) for 15 min. Interleaved box and whiskers: the whiskers represent the minimum and maximum values while the line in between represents the median value. *N* = 3 independent experiments. (**D**) Semi-quantitative Western blot analysis of signaling molecules in HUVECs after STAT3 knockdown and treatment under the conditions mentioned in (**B**) for 15 min. Interleaved box and whiskers: the whiskers represent the minimum and maximum values while the line in between represents the median value. *N* = 3 independent experiments

To evaluate the role of the STAT3 pathway in mediating the angio-modulatory effect of IL-11 cis- and trans-signaling, we next performed STAT3 knockdown experiments (Fig. 5). Compared with VEGF stimulation alone, IL-11 + VEGF enhanced vascular endothelial sprouting. In the scrambled siRNA control group, there was less sprouting in the IL-11 + VEGF group than in the VEGF group (Fig. 5A). In contrast, STAT3 knockdown did not affect the previously observed enhanced sprouting effect of IL-11 + sIL-11Rα + VEGF (Fig. 5B).

We then extracted protein from STAT3 knockdown and control cells to test for compensatory activation of other pathways (Fig. 5C + Suppl. Fig. S4A). Western blot data indicated that STAT1 and ERK signaling was enhanced in IL-11-stimulated knockdown cells (Fig. 5C). In contrast, STAT1 activation in STAT3-knockdown cells was slightly lower in the IL-11 + sIL-11Rα + VEGF group than in the control group or in the IL-11 + sIL-11Rα group (Fig. 5D). Furthermore, STAT3 knockdown reduced Akt activation in all groups except for IL-11 + VEGF-stimulated cells (Fig. 5C + D). Supplemental figure S4B summarizes the observed compensatory signaling patterns in the case of STAT3 loss of function.

**Fig. 6:**
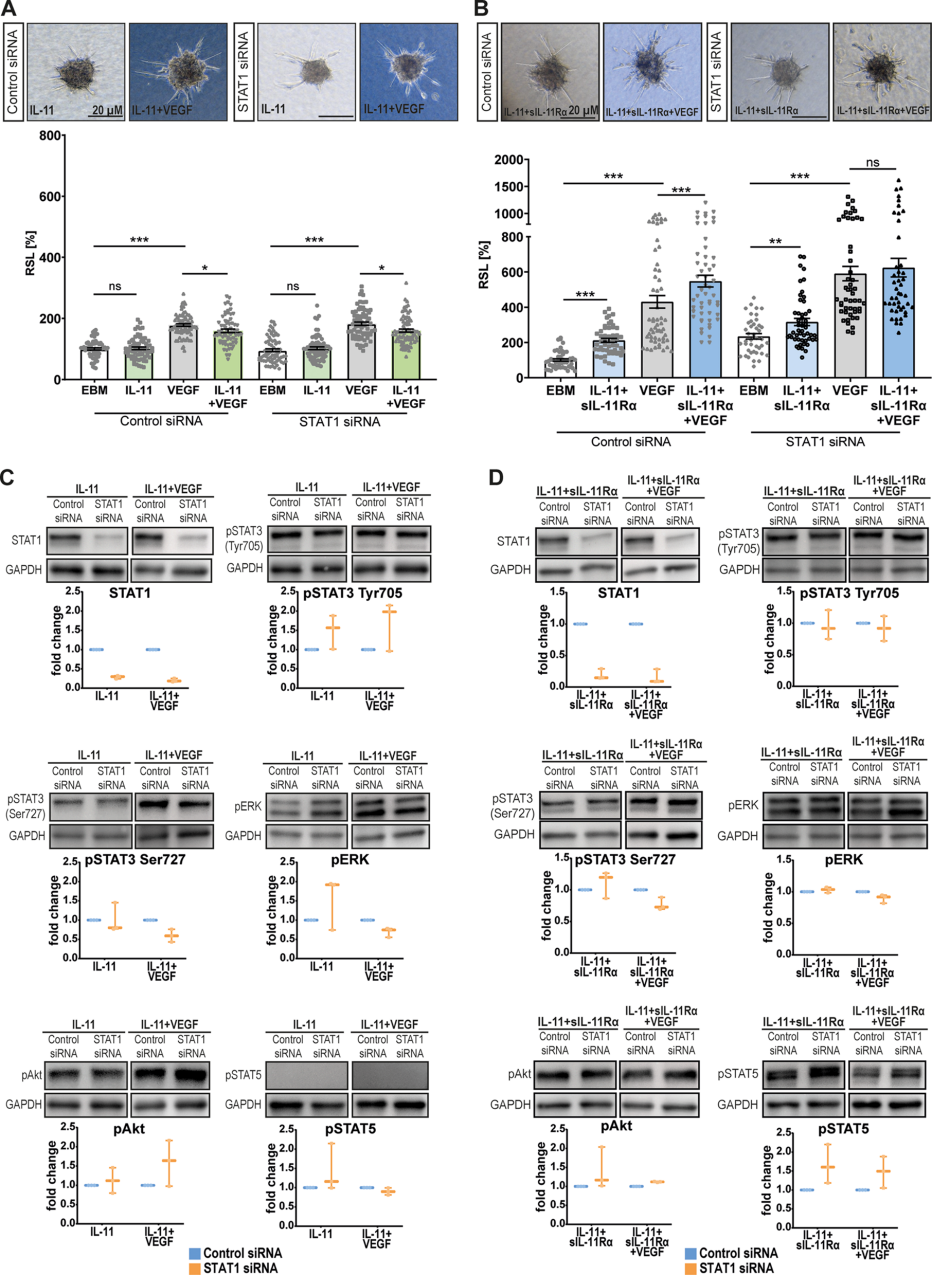
Characterization of angiogenic effects and compensatory changes of signaling pathways after STAT1 knockdown. (**A**) Spheroid sprouting assay using HUVECs: Cells transfected with control siRNA or STAT1 siRNA were treated under following conditions: EBM (negative control), IL-11, VEGF (positive control), IL-11+VEGF. RSL = Relative Sprouting Length. *N* = 4 independent experiments each consisting of 14-22 spheroids per group and experiment, statistical testing: Kruskal-Wallis Test adjusted for multiple testing, *p<0.05. Scale bar 20 µM. (**B**) Spheroid sprouting assay using HUVECs: Cells transfected with control siRNA or STAT1 siRNA were treated under following conditions: EBM (negative control), IL-11+sIL-11Rα, VEGF (positive control), IL-11+sIL-11Rα+VEGF. RSL = Relative Sprouting Length. *N* = 3 independent experiments each consisting of 11-20 spheroids per group and experiment, statistical testing: Kruskal-Wallis Test adjusted for multiple testing, *p<0.01. Scale bar 20 µM. (**C**) Semi-quantitative Western blot analysis of signaling molecules in HUVECs after STAT1 knockdown and treatment under the conditions mentioned in (**A**) for 15 min. Interleaved box and whiskers: the whiskers represent the minimum and maximum values while the line in between represents the median value. *N* = 3 independent experiments. (**D**) Semi-quantitative Western blot analysis of signaling molecules in HUVECs after STAT1 knockdown and treatment under the conditions mentioned in (**B**) for 15 min. Interleaved box and whiskers: the whiskers represent the minimum and maximum values while the line in between represents the median value. *N* = 3 independent experiments

Since Western blot data and RNA-Seq data revealed that STAT1 may strongly influence the angio-modulatory effect of IL-11 cis- and trans-signaling, we wanted to further evaluate its role. In the presence of VEGF, STAT1 knockdown did not affect the original anti-angiogenic effect of IL-11 (Fig. 6A) but did reduce the additive proangiogenic effect of IL-11 + sIL-11Rα (control siRNA ∆= +27%, STAT1 siRNA ∆= +6%) (Fig. 6B).

Western blot data revealed no changes in intracellular signaling patterns in IL-11-stimulated cells following STAT1 knockdown (Fig. 6C) while a compensatory slight upregulation of pSTAT5 was detected in IL-11 + sIL-11Rα-stimulated STAT1 knockdown cells (Fig. 6D). Supplemental figure S4C shows a summary of the STAT1 knockdown results.

### IL-11 decreases retinal neovascularization in vivo

**Fig. 7:**
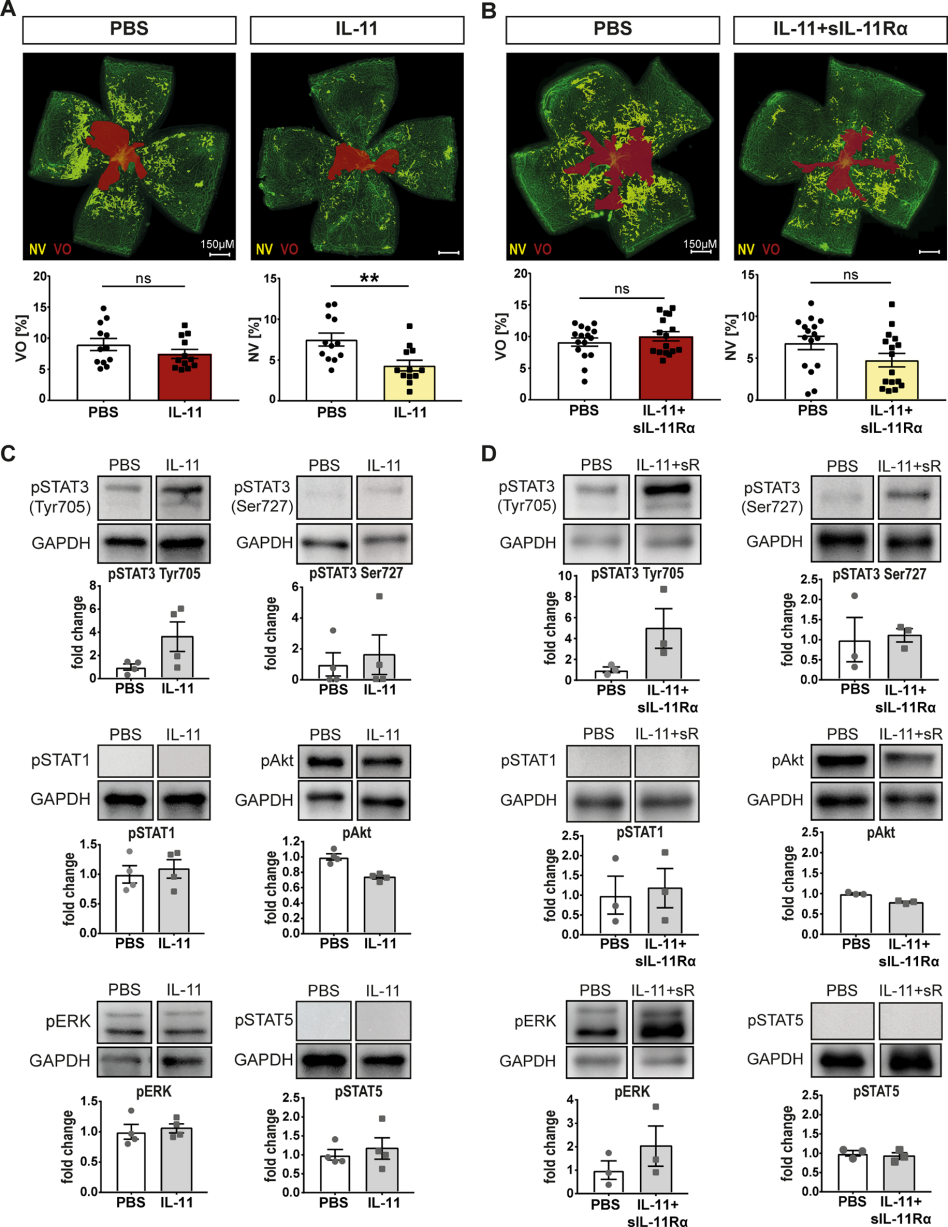
IL-11 and IL-11 + sIL-11Rα decrease the retinal neovascularization in vivo. (**A**) Representative images of retinal flatmounts with quantification of NV and VO in OIR P17 mice after intravitreal IL-11 or PBS control injection. *N* = 12 mice per condition from three independent OIR experiments, statistical testing: Mann–Whitney Test, *p<0.01. (**B**) Representative images of retinal flatmounts with quantification of NV and VO in OIR P17 mice following intravitreal IL-11+sIL-11Rα or PBS control injection. *N* = 16 mice per condition from three independent OIR experiments, statistical testing: Mann–Whitney Test. (**C**) Western blot of whole retina lysates 12 h after intravitreal injection of IL-11 or PBS control. *N* = 4 biological replicates representing four mice. (**D**) Western blot of whole retina lysates 12 h after intravitreal injection of IL-11+sIL-11Rα or PBS control. *N* = 3 biological replicates representing three mice

To further explore the role of IL-11 signaling in retinal neovascularization and capillary regrowth in vivo, we used a murine OIR model. Intravitreal injection of mIL-11 led to a significant decrease of neovascularization (NV) at P17 (mean ∆ = -45.6%) but had no statistically significant effect on capillary regrowth (VO) (Fig. 7A). Combined treatment with IL-11 + sIL-11Rα elicited a somewhat weaker effect (mean ∆ = -42.7%, not significant) while VO formation was not affected (Fig. 7B). Analysis of IL-11 and IL-11Rα levels under normoxia and after OIR (Suppl. Fig. S5) revealed no significant differences between those groups suggesting that the effect seen is most probable not caused by endogenously generated sIL-11Rα.

Next, we analyzed the activation pattern of IL-11-induced cis- and trans-signaling pathways in vivo using whole retina lysates to discover possible differences between in vivo- and in vitro- activation.

Similar to the in vitro data (Fig. 3B), mIL-11 strongly enhanced pSTAT3 Tyr705 in vivo (Fig. 7C) with a concomitant slight decrease in Akt phosphorylation (Fig. 7C).

Intravitreal injection of IL-11 + sIL-11Rα also induced a strong pSTAT3 Tyr705 signal (Fig. 7D) as well as a decrease in Akt phosphorylation. Additionally, an increase in pERK was detected (Fig. 7D). As in eyes injected with IL-11 (Fig. 7C), IL-11 + sIL-11Rα did not induce phosphorylation of STAT5 or STAT3 (Ser727) (Fig. 7D).

**Fig. 8:**
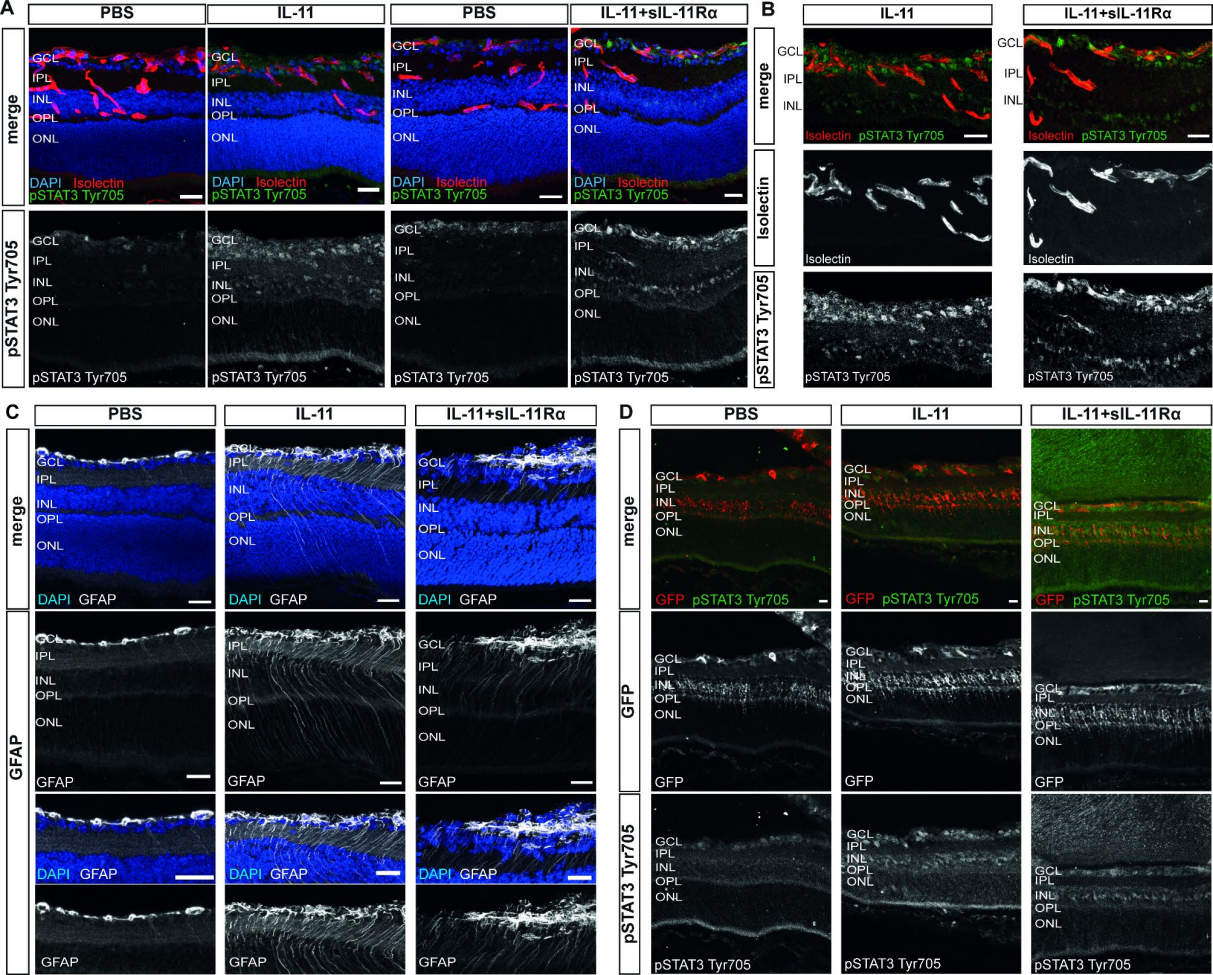
IL-11 and IL-11 + sIL-11Rα activate Müller cells in vivo. (**A**) Representative images of retinal cryosections of C57BL/6J mice 12 h after injection with IL-11, IL-11+sIL-11Rα or PBS control at OIR P12. Scale bar: 25 μm. (**B**) Representative higher magnification images of the ganglion cell layer, inner plexiform layer and inner nuclear layer after injection with IL-11 or IL-11+sIL-11Rα for better visualization of pSTAT3 Tyr705 and Isolectin co-staining. Scale bar: 25 μm. (**C**) Representative images retinal cryosections and of higher magnification images of C57BL/6J mice for GFAP 12 h after injection with IL-11, IL-11+sIL-11Rα or PBS control at OIR P12. Scale bar: 25 μm. (**D**) Representative images of retinal cryosections of ALDH1L1-GFP^+^ transgenic mice 12 h after injection with IL-11, IL-11+sIL-11Rα or PBS control at OIR P12. GCL = ganglion cell layer, IPL = inner plexiform layer, INL = inner nuclear layer, OPL = outer plexiform layer, ONL = outer nuclear layer. Scale bar: 25 μm

Taken together, these data suggest that cis- and trans-signaling have similar angiomodulatory effects in the retina which corresponds to similar signaling patterns in whole retina lysates. We next wanted to identify the cells that were responsive to mIL-11- or mIL-11 + sIL-11Rα- induced effects in the retina. Based on our western blot results showing a strong increase in pSTAT3 Tyr705 levels in response to mIL-11 as well as mIL-11 + sIL-11Rα, we used pSTAT3 Tyr705 as an immunohistochemical marker in retinal cryosections to identify mIL-11- and mIL-11 + sIL-11Rα- responsive cells. Figure 8A and B (+ Suppl. Fig. S6A + B) indicate that mIL-11 and mIL-11 + sIL-11Rα induce similar patterns of pSTAT3 Tyr705 staining in the superficial vascular plexus/ ganglion cell layer (GCL) as well as in the inner nuclear layer (INL). To rule out the source of signal coming from immune cells, we performed IBA1 staining and observed no significant differences in terms of quantity or intensity between the control and treatment group (Suppl. Fig. S6C). Furthermore, the expression of GFAP, a marker of astrocytes as well as activated Müller cells, was strongly enhanced throughout all retinal layers suggesting a strong Müller cell activation in response to mIL-11 as well as mIL-11 + sIL-11Rα (Fig. 8C). To validate our results, we injected IL-11 and IL-11 + sIL-11Rα intravitreally into ALDH1L1-GFP^+^ transgenic mice in which the Müller glial cell marker ALDH1L1 is labeled with green fluorescent protein (GFP) [38]. In the eyes injected with IL-11 or IL-11 + sIL-11Rα, we observed a strong pSTAT3 Tyr705 signal in the ganglion cell layer (GCL) as well as in the inner nuclear layer (INL) where the Müller cell nuclei are located. Notably, the pSTAT3 Tyr705 signal colocalized with the GFP signal from the INL of the ALDH1L1-GFP^+^ transgenic mice suggesting that IL-11 and IL-11 + sIL-11Rα activated Müller cells (Fig. 8D).

### In Müller cells, IL-11 cis- and trans-signaling induce similar intracellular signaling patterns

**Fig. 9:**
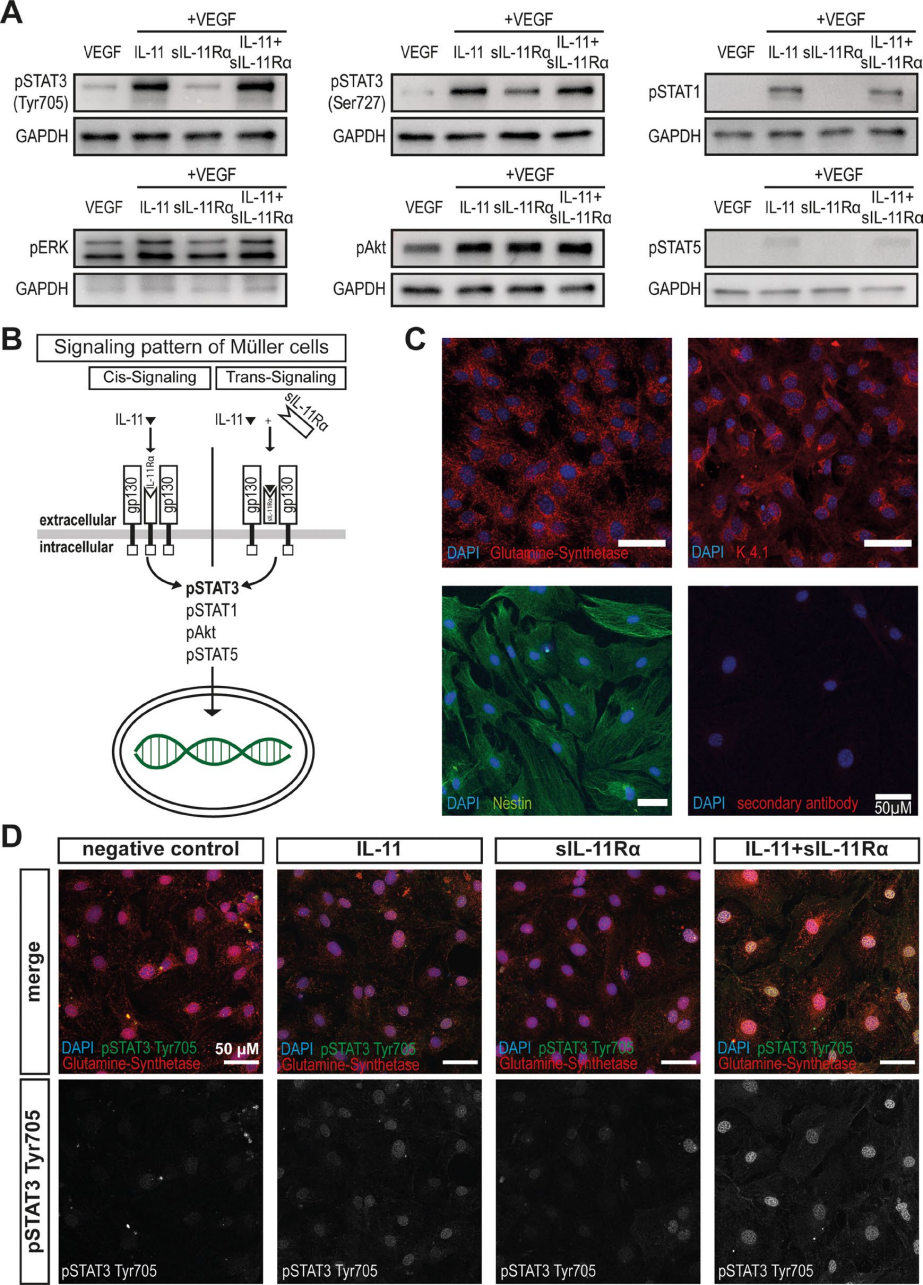
In Müller cells, IL-11 cis- and trans-signaling activate similar intracellular signaling pathways in vitro. (**A**) Western blot analysis for activated intracellular signaling molecules in Müller cells stimulated with VEGF, mIL-11+VEGF, sIL-11Rα+VEGF or mIL-11+sIL-11Rα+VEGF for 15 min. *N* = 4 independent experiments. (**B**) Graphical summary of the activated pathways by IL-11 and IL-11+sIL-11Rα in Müller cells. (**C**) Immunocytochemistry of primary Müller cells expressing the Müller glia-specific markers glutamine synthetase, K_ir_4.1 and nestin. *N* = 3 independent experiments, scale bar: 50 μm. (**D**) Immunocytochemistry of primary Müller cells after stimulation with control medium (Diff), mIL-11, sIL-11Rα or mIL-11+sIL-11Rα for 15 min. *N*=3 independent experiments, scale bar: 50 μm

As the cryosections suggested a strong IL-11-induced Müller cell activation in vivo (Fig. 8C), we further investigated the responses of Müller cells to IL-11 cis- and trans-signaling using cultivated murine primary Müller cells. Western blot analysis revealed that both IL-11 cis- and trans-signaling strongly induced the phosphorylation of STAT3 at Tyr705 as well as of STAT1 in Müller cells (Fig. 9A + Suppl. Fig. S7). Furthermore, a slight concomitant activation of pSTAT3 Ser727, pAkt and pSTAT5 was detected. Figure 9B summarizes these findings. Immunocytochemistry revealed the expression of Müller glia-specific markers including glutamine synthetase, K_ir_4.1 and nestin in our primary Müller cell culture (Fig. 9C). Furthermore, nuclear accumulation of pSTAT3 Tyr705 was visualized in mIL-11- and mIL-11 + sIL-11Rα- treated Müller cells which is consistent with the Western blot data (Fig. 9D).

## Discussion

Our study revealed that the opposite angiomodulatory effects of IL-11 on vascular endothelial cells depend on the availability of the soluble IL-11 receptor. In vitro, cis-signaling is anti-angiogenic, while trans-signaling has proangiogenic and pro-migratory effects on vascular endothelial cells. However, in vivo, both IL-11 cis- and trans-signaling reduced retinal angiogenesis at OIR P17 which was correlated with strong Müller cell activation. This highlights the complex interactions of vascular and glial cells in the retina in vivo.

In human vitreous samples, we detected highly elevated levels of IL-11Rα, but not IL-11 in the vitreous of PDR patients compared to those in the vitreous of control patients (Fig. 1B). This led to our hypothesis that the proangiogenic response to IL-11 in the eye is caused by the presence of IL-11Rα. However, the specific cellular origin of IL-11 or IL-11Rα in the eye, particularly in the retina, has yet to be definitively determined. Our findings contrast with a previous study reporting a significant upregulation of IL-11 in PDR [35], possibly due to interindividual biological variations or differences in clinical criteria. We defined non-diabetic patients with macular pucker as the control group. The impact of variations in the collection, handling, and storage of vitreous samples on cytokine stability also deserves consideration [39]. Our findings on vitreal IL-11Rα levels in PDR patients measured by ELISA are in line with Western blot data reported by Abu El-Asrar et al. [35].

Currently, there are limited data on the angiomodulatory potential of IL-11. Our in vitro data revealed that IL-11 trans-signaling had pro-angiogenic effects on vascular endothelial cells while IL-11 alone had an antiangiogenic effect (Fig. 2). Unlike HUVECs, HRMVECs do not express IL-11Rα (Suppl. Fig. S3). However, we observed similar contrasting effects of IL-11 cis- and trans-signaling in both cell types (Fig. 2B + Suppl. Fig. S2B). This finding suggests that the opposing angiomodulatory effects are primarily attributable to trans-signaling induced by IL-11 + sIL-11Rα. Previous studies have shown varying effects of the angiomodulatory effect of IL-11 on different diseases, such as rheumatoid arthritis [40] and lung cancer [41]. The discrepancy between these findings and our results supports the assumption that the effects of cis- and trans-signaling mediated by IL-11 may be context-dependent and disease-specific [42].

Our RNA-sequencing data revealed enrichment of metabolic transcripts such as ATP synthesis and oxidative phosphorylation following IL-11 + VEGF stimulation of vascular endothelial cell spheroids. Consequently, IL-11 + sIL-11Rα + VEGF led to a depletion of these metabolic transcripts. Subsequent Seahorse experiments confirmed metabolic differences between cis- and trans-signaling but revealed increased mitochondrial metabolism with trans-signaling compared to cis-signaling. These discrepancies may be attributed to post-transcriptional regulation since RNA-sequencing only measures mRNA levels or from adaptive cellular responses over time that are not evident in the RNA-seq data. Furthermore, the Seahorse assay is a 2D assay while we used spheroids from the 3D assay for RNA-Seq. Our group previously demonstrated that cells behave differently in 2D versus 3D culture systems with 3D assays better reflecting in vivo processes [37].

The signaling pathways STAT1 and STAT3 are known to play opposing roles in angiogenesis, inflammation and tumor growth [43]. Our results revealed that STAT3 knockdown slightly decreased the endothelial sprouting rate in the control groups (EBM and VEGF) consistent with its known role in promoting cell proliferation, motility and immune tolerance [17, 43–45]. Surprisingly, the sprouting rate of STAT3 knockdown cells stimulated with IL-11 + VEGF or IL-11 + sIL-11Rα + VEGF increased (Fig. 5A + B) contradicting the established proangiogenic role of STAT3. Our findings in this study are, however, supported by our previous work on CNTF and OSM [17] as well as other studies [43, 46, 47]. The delayed yet long-lasting compensatory activation of other pro-angiogenic signaling pathways, such as the ERK and AKT pathways [43, 46], as shown for OSM [16], may explain these observations.

Similar patterns were observed in our STAT1 knockdown experiments: loss of STAT1 attenuated the pro-angiogenic effect of IL-11 trans-signaling by IL-11 + sIL-11Rα + VEGF (Fig. 6B). This finding supports our hypothesis that STAT1 mediates proangiogenic effects in this setting. Previous studies have reported the tumor-promoting activities of STAT1 in breast cancer and leukemia among others [48]. The pro-apoptotic trait of STAT1 can be overcome by its constitutive overexpression [49].

The knockdown experiments strongly suggested that STAT1 and STAT3 have, at least partially, opposing roles in IL-11 cis- and trans-signaling and keep themselves in check. If one of these pathways is inactive, alternative pathways may compensate for and activate factors essential for cell growth or cell death [50].

In vivo, intravitreal injections of IL-11 led to a strong reduction in retinal neovascularization while IL-11 + sIL-11Rα had a weaker effect (Fig. 7A + B). This effect seems to be due to the common activation of Müller cells since both, IL-11 and IL-11 + sIL-11Rα, induced a strong GFAP signal in the immunohistochemical staining which colocalized with a strong pSTAT3 signal in the INL (Fig. 8C + D). This finding aligns with previous studies showing that CNTF or OSM injections primarily activated Müller cells rather than vascular endothelial cells [14, 51]. Many studies have shown that the interaction between neuronal, glial and vascular endothelial cells in the retina is crucial for the development of retinal neovascular disease [14]. Müller cell gliosis in response to activation can exert both neuroprotective and neurodegenerative effects. The gliotic reaction of Müller cells consists of a non-specific response that occur regardless of the initiating stimulus, and of a specific response tailored to the underlying pathogenic factor or mechanism [52]. Upregulation of glial fibrillary acidic protein (GFAP) represents a sensitive, non-specific response that serves as an early biomarker for retinal injury and reactivity. The observed gliosis following IL-11 or IL-11 + sIL11Rα administration, coupled with attenuated neovascularization in the OIR model, suggests a potentially protective role for Müller cells. However, further investigations are needed to elucidate the precise mechanisms by which Müller cell activation in response to IL-11 signaling modulates endothelial cell behavior and vascular remodeling. Müller cells have been described to exert proangiogenic as well as antiangiogenic effects on vascular endothelial cells in the retina. For a proangiogenic effect, Müller cells can produce HIF-1α [53] and VEGF leading to ischemia-induced retinal neovascularization and vascular leakage [54]. In terms of antiangiogenic effects, Müller cells may also inhibit proliferation and retinal neovascularization by secreting different factors, such as CXCL10 [55], PEDF [56] or TGF-β2 [57], or by inducing the anti-angiogenic *PAI-1* and suppressing the pro-angiogenic *Id2* [58].

Our study suggests that IL-11 cis-signaling may have a neuroprotective role in the retina, similar to a study examining the role of IL-6 cis-signaling in Müller cells in vivo [59]. Furthermore, only the injection of IL-11 + sIL-11Rα but not IL-11 led to slight activation of the ERK pathway which is known for its proangiogenic effects [60]. This might explain why the injections of IL-11 + sIL-11Rα led to a slightly reduced NV formation (Fig. 7B).

Interestingly, in vitro experiments with Müller cells revealed no difference in the activation patterns of cis- and trans-signaling pathways including the STAT3 (Tyr705), STAT1, Akt and STAT5 signaling pathways (Fig. 9).

Limitations of this study apply to the scope of cellular models investigated and the complexity of IL-11 signaling mechanisms. The current research suggests that vascular endothelial cells can respond to IL-11. One key limitation is the use of mostly one endothelial cell type (primarily HUVEC) with confirmatory experiments using HRMVECs given the fact that IL-11Rα expression levels differ between both cell lines underlining the diversity of vascular endothelial cell biology. Furthermore, additional studies addressing IL-11 signaling in Müller glia and utilizing co-culture systems that combine multiple retinal cell types would provide a more comprehensive understanding of the neuro-vascular interaction and how they are impacted in disease states. Likewise, the dual nature of IL-11 signaling, requiring both cis- and trans-activation, presents challenges in studying this pathway and determining its precise mechanisms of action. Future work will need to carefully dissect these intricacies of IL-11 biology to develop a clearer picture of its function in retinal angiogenesis and vascular homeostasis. Addressing these limitations will be crucial for translating these findings into potential therapeutic strategies targeting the retinal vasculature.

In conclusion, our study revealed different angiogenic effects of cis- and trans-signaling on IL-11-mediated angiogenesis which can be explained by individual signaling responses leading to unique associated transcriptomic shifts and metabolic changes. The presence of soluble IL-11 receptors may have a significant, yet often overlooked, impact on diseases characterized by abnormal angiogenesis. Characterizing the expression status of soluble interleukin receptors could provide an important foundation for developing targeted therapeutic interventions, potentially opening new avenues for the treatment of angiogenesis-related disorders.

## Methods

### Patient samples

To evaluate the role of IL-11 and IL-11Rα in the pathogenesis of PDR, undiluted vitreous samples from patients with PDR or macular pucker (as a clinical control cohort) were collected at the beginning of pars plana vitrectomy, as were blood samples. A diagnosis of diabetes mellitus was an exclusion criterion in the control group. The key clinical characteristics of the patients in the PDR and control groups are detailed in Tables 1 and 2. Samples were centrifuged (vitreous: 500 x g for 20 min, blood: 3000 x g for 15 min) at 4 °C and vitreous and plasma supernatants were aliquoted and frozen at − 80 °C until the ELISA experiments were performed (Fig. 1A).

### Cell culture

Human umbilical vein endothelial cells (HUVECs, #2519A; Lonza) were cultured in endothelial growth medium-2 (EGM, #CC-3162; Lonza), stored frozen in liquid nitrogen at passage 3 (P3) and used up to P6.

Human retinal microvascular endothelial cells (HRMVECs, #ACBRI 181, Cell Systems) were cultivated in HRMVEC growth medium (#PB-MH-100-4090, PELOBiotech) supplemented with 10% FBS. HRMVECs at P9 were used for all experiments.

Primary Müller cells were obtained from the retinas of 12-day-old C57/Bl6J mice according to a previously published protocol [55]. In brief, both isolated retinas from each animal were transferred to a mixture of papain, DNase and ovomucoid (Papain Dissociation system, #LK003150; Worthington Biochemical Corporation), triturated and centrifuged at 4 °C. The pellet was resuspended in growth medium containing human endothelial growth factor (100 ng/mL, #PHG0311; Thermo Fisher Scientific), Neurobasal-A medium (#10888022; Thermo Fisher Scientific), N2 supplement (#17502048; Thermo Fisher Scientific), 10% FBS superior (#S0615; Bio&SELL), L-glutamine Q8 (2 mM, #25030081; Thermo Fisher Scientific) and penicillin-streptomycin (#P4333; Sigma-Aldrich). Müller cells were incubated in growth medium until confluency. After the 2nd split and reaching approximately 80% confluence, the Müller cells were transferred to differentiation medium supplemented with Neurobasal-A medium, 1% FBS, N2 supplement, L-glutamine (2 mM), Pen/Strep and B27 supplement (50x, #17504044; Thermo Fisher Scientific) for one week.

In all the following experiments, the cells were stimulated with or without VEGF (25 ng/mL, #100 − 20; Peprotech) under the following conditions unless otherwise indicated. In HUVECs, we used human IL-11 (IL-11, 100 ng/mL, #200 − 11; Peprotech), soluble human IL-11Rα (sIL-11R, 400 ng/mL, #8895-MR-050; R&D Systems) or a combination of IL-11 + sIL-11Rα. Murine Müller cells were stimulated with murine IL-11 (mIL-11, 100 ng/mL, #220 − 11, Peprotech), soluble mIL-11Rα (600 ng/mL, #7405-MR-050, R&D Systems) or mIL-11 + sIL-11Rα.

### Spheroid sprouting assay

We performed an endothelial spheroid sprouting assay as previously published [17, 55]. In brief, endothelial spheroids were formed in a hanging drop consisting of 200.000 HUVECs or HRMVECs resuspended in 80% EGM and 20% methylcellulose. The next day, spheroids were harvested, embedded in a collagen matrix (Collagen I, Rat Tail Cat#: 354236, Corning) and stimulated with cytokines for 17 h. Phase-contrast micrographs of individual spheroids were taken (at least 10 spheroids/well) with an inverted microscope (Zeiss Axio Vert. A1, Jena, Germany) at 10x magnification. For quantification, each sprout of the spheroid was manually labeled using the “straight-line” tool of the program “ImageJ-Fiji”. The total sprouting length of each stimulation was normalized to that of the control (EBM or VEGF) using GraphPad Prism, resulting in the relative sprouting length (RSL).

### Wound healing assay

The wound healing assay was performed as previously described [37]. HUVECs (20000 per well) were seeded in 0.1 mL EGM in 96-well IncuCyte ImageLock plates (#BA-04856, Sartorius), allowed to settle for 8 h and then starved overnight. After creating a wound using the IncuCyte WoundMaker (IncuCyte^®^ Cell Migration Kit, #4493, Sartorius) and stimulating the cells with cytokines, images of each well were taken every hour for 24 h using an IncuCyte S3 Live-Cell Analysis System (#4647, Sartorius). The relative wound density (RWD) was analyzed using the IncuCyte software (Integrated Cell Migration analysis module, #9600-0012, Sartorius).

### STAT3 and STAT1 knockdown using siRNA

For a transient siRNA knockdown, HUVECs were transfected with OptiMEM (#31985062, Thermo Fisher Scientific) containing either control siRNA (15 nM, #12935300, Thermo Fisher Scientific), STAT3 siRNA (15 nM, #1299001, Thermo Fisher Scientific) or STAT1 siRNA (15 nM, #10620318, Thermo Fisher Scientific) and 0,4% RNAiMAX Lipofectamine reagent (#13778030, Thermo Fisher Scientific). Transfection efficiency was assessed by Western blot analysis. Cells were harvested 48 h post transfection for Western blot experiments while the analysis of the spheroid sprouting assay was conducted 72 h after transfection.

### Protein analysis

Western blot: For protein analysis, HUVECs, HRMVECs and single retinas from mice were lysed with T-Per buffer (#78510; Thermo Fisher Scientific) containing 1% phosphatase (#78420, Thermo Fisher Scientific) and proteinase inhibitors (#87786; Thermo Fisher Scientific). After denaturation, proteins were separated by gel electrophoresis, transferred to a membrane (Immobilon-P Membrane, #IPVH00010, Merck Millipore), blocked and incubated overnight at 4 °C using the following primary antibodies:

Phospho-Jak Family Antibody Sampler Kit (#97999, Cell Signaling Technology), STAT3 (79D7) rabbit mAb (#4904, Cell-Signaling), pSTAT3 (Tyr705) rabbit mAb (#9145, Cell Signaling Technology), pSTAT3 (Ser727) rabbit mAb (#9134, Cell Signaling Technology), STAT1 rabbit Antibody (#9172, Cell Signaling Technology), pSTAT1 rabbit mAb (#9167, Cell Signaling Technology), pERK p44/42 MAPK (T202/Y204) rabbit mAb (#4370, Cell Signaling Technology), pAkt (Ser473) rabbit mAb (#9271, Cell Signaling Technology), pSTAT5 (#Y694, Cell Signaling Technology) rabbit mAb (#9359, Cell Signaling Technology), mouse GAPDH Ab (#MAB374, Merck), mouse monoclonal anti-α-Tubulin (#T-9026, Sigma-Aldrich).

On the second day, the membrane was incubated with the following secondary antibodies for one hour: Peroxidase AffiniPure Goat Anti-Rabbit IgG (H + L) (1:10000, #111-035-003, Jackson ImmunoResearch), Peroxidase AffiniPure Goat Anti-Mouse IgG (H + L) (1:10000, #115-035-003, Jackson ImmunoResearch).

Afterwards, the membrane was coated with ECL (Amersham ECL Western Blotting Detection Reagent, #RPN2109, Cytiva), and images of the bands were captured using the Evolution CaptEdge program (FUSION FX WB and Chemiluminescence Imaging System, Vilber).

The Western blot bands were semiquantified using the program „Fiji–ImageJ“. The protein of interest was first normalized to the loading control protein (GAPDH or α-tubulin) before calculating the relative fold of protein expression compared to either the negative control (EBM) or positive control (VEGF).

A summary of uncut blots can be found in the supplemental material.

ELISA: The cytokine levels in vitreous and plasma samples were determined using the human IL-11 ELISA Kit (#ab100551; Abcam) and the human IL-11Rα ELISA Kit (#MBS453817, MyBioSource) according to the manufacturer’s instructions.

### RNA-sequencing

In total 12 samples from three independent spheroid sprouting experiments of HUVECs were analyzed using RNA Sequencing (Fig. 4A). Total RNA was extracted from spheroids by the RNeasy Micro Kit (QIAGEN, Hilden, Germany) including a collagen digestion. The SMARTer Ultra Low Input RNA Kit for Sequencing v4 (Clontech Laboratories, Inc., Mountain View, CA, USA) was used to generate first strand cDNA from approximately 500 pg of total-RNA. Double-stranded cDNA was amplified by LD PCR (12 cycles) and purified via magnetic bead clean-up. Library preparation was carried out as described in the Illumina Nextera XT Sample Preparation Guide (Illumina, Inc., San Diego, CA, USA). Equimolar amounts of each library were sequenced on an Illumina NextSeq 2000 instrument using one 50 cycles P3 Flow Cell with the dual index, single-read (SR) run parameters. Image analysis and base calling were performed using by the Real Time Analysis Software (RTA) v3.7.17. The resulting .cbcl files were converted into .fastq files with the bcl2fastq v2.20 software.

RNA extraction, library preparation and RNA-seq were performed at the Genomics Core Facility “KFB - Center of Excellence for Fluorescent Bioanalytics” (University of Regensburg, Regensburg, Germany; www.kfb-regensburg.de).

### Statistics and bioinformatics

Raw .fastq files were uploaded to the galaxy.eu web platform [61] (https://usegalaxy.eu). FastQC software [62] (Galaxy Version 0.73) was used to evaluate the quality of all files. Raw reads were mapped to a human reference genome (GRCh38) and its corresponding gene annotation file provided by GENCODE (downloaded September 2021) with the STAR aligner [63] (Galaxy Version 2.7.8a) and assigned to genes by featureCounts [64] (Galaxy Version 2.0.1). For all software standard settings were used. Generated count tables were imported into R 4.0.2 (https://www.rproject.org) and detection of differentially expressed genes was performed with DESeq2 [65]. Using the biomaRt package [66], ENSEMBLE gene ID was linked to the HGNC system. After shrinkage of log2-fold changes by DESeq2 in standard settings Gene Set Enrichment Analysis (GSEA) was performed by fgsea [67] by sorting genes according to their log2-fold change and excluding genes with < 10 counts. Gene sets from the MSigDB database (http://www.gseamsigdb.org/gsea/msigdb, downloaded April 2021) were used for analysis. ComplexHeatmaps [68] and ggplot2 [69] were used to visualize the data. PCA was performed with DESeq2 without batch effect correction and no samples were excluded.

### Seahorse XFe-96 extracellular metabolic flux analysis

The Seahorse XFe96 Analyzer (Agilent Technologies, Santa Clara, CA, USA) was used to measure the oxygen consumption rate (OCR) and the extracellular acidification rate (ECAR) according to the manufacturer’s instructions and a previously published protocol [17]. Briefly, HUVECs were seeded at a density of 20.000 cells/ well in XF96-well cell culture plates (Seahorse XF96 V3 PS Cell Culture Microplates, #101085-004, Agilent Technologies) and incubated with the abovementioned cytokine combinations for 15 h. The medium was replaced with DMEM (#D5030, Sigma-Aldrich) containing 10 mM glucose, 2 mM glutamine and 10 mM HEPES (pH 7.4) one hour before the test and a standardized Seahorse XF Cell Mito Stress Test was run. Afterwards, the CyQUANT™ Cell Proliferation Assay (#C7026, Thermo Fisher Scientific) was used to normalize the data to the cell number in each well.

### Animal models

OIR model: Wild-type C57BL/6J mice (Charles River Laboratories, strain code 632) and ALDH1L1-GFP^+^ transgenic mice (B6;FVB-Tg(Aldh1l1-EGFP/Rpl10a)JD130Htz/J, The Jackson Laboratory, strain #030247 [70]), both of which are negative for the known pathogenic RD1 and RD8 mutations, were used for OIR experiments [71]. In brief, 7-day-old (P7) pups were exposed to hyperoxia (75% oxygen) for five days and transferred to room air at P12. 0.5 µL of mIL-11 (100 ng/mL) or the combination of mIL-11 and its soluble receptor (mIL-11R, 600 ng/mL) was injected intravitreally at P12. PBS injections in the contralateral eye served as controls. Eyes were harvested from mice weighing between 5 and 7 g [72] following cervical translocation at the time points outlined throughout the manuscript.

### Immunohistochemistry in vitro and in vivo

Primary Müller cells at a density of 8.000 cells per well were seeded onto fibronectin-precoated (#FC010, Millipore) coverslips in 24 well plates, stimulated and fixed in 100% methanol for 10 min on ice. Following permeabilization, cells were incubated overnight at 4 °C with the following primary antibodies: pStat3 (Tyr705) Mouse mAb (#4113, Cell Signaling), Anti-Glutamine Synthetase antibody (#ab73593, abcam), Anti-K_ir_4.1 antibody (#APC-035, alomone lab), Anti-Nestin antibody (#ab134017, abcam). Immunohistochemical analyses were performed as previously described [55]. Harvested bulbi were fixed in 4% PFA for 40 min on ice, dissected to retrieve the retina which was stained overnight in Isolectin Gs-Ib4 1:100 (#FL-1201; Vector Laboratories), and cut to generate flatmount slides which were subsequently imaged with a slide scanner (NanoZoomer S60 Digital slide scanner, #C13210-01, Hamamatsu). To quantify the areas of vaso-obliteration (VO) and neovascularization (NV), we used established techniques [73]. VO and NV are presented as values normalized to the total flatmount area.

For retinal cryosections, eyes were fixed in 2% PFA for one hour 12 h after intravitreal injection and placed in 20% sucrose solution for 24 h. The slides were fixed in cold acetone, permeabilized with 0.3% Triton, blocked and incubated with the following primary antibodies overnight: pSTAT3 (Tyr705), chicken anti-mouse Gfap Ab (#ab4674; abcam), anti-GFP antibody (#ab13970, abcam).

### PCR

RNA isolation from HUVECs, HRMVECs, Müller cells and murine retinas was performed using 700 µL of ice-cold Qiazol. Retinal samples were collected 24 h post injection at P13. All samples were stored at -80 °C prior to processing.

RNA was extracted using the miRNAeasy Mini Kit (#217004; Qiagen) according to the manufacturer’s instructions and transcribed to cDNA using SuperScript™ IV Reverse Transcriptase (200 U/µL) (#18090-010, Thermo Fisher Scientific). cDNA was stored at -20 °C until further use.

For gel electrophoresis, a PCR master mix was prepared consisting of 15.5 µL nuclease-free water, 5 µL Green Buffer (#M7918, Promega), 0.4 µL dNTP 10mM (#R0192, Thermo Fisher Scientific), 0.1 µL GoTaq G2 Polymerase (#M7408, Promega) and 2 µL of primers (5 µM, Suppl. Table S1) per sample. PCR products or a DNA ladder (GeneRuler 50 bp DNA Ladder, #SM0373, Thermo Fisher Scientific) were mixed with Gel-Red (1:100, GelRed™ 10000x, #41003, Biotium, VWR) and run on a 2% agarose gel for 1.5 h at 110 V.

### qPCR

For qPCR, 2 µL of DNA was combined with 10 µL SYBR Green, 7.2 µL nuclease-free water, and 0.8 µL primer (10 µM, Suppl. Table S2) in a LightCycler 96-well plate (LightCycler 480 Multiwell Plate 96, white, #04729692001, Roche). The assay was run on a LightCycler 96 according to the LightCycler 480 system protocol. Two replicates of mouse retinas were used for each gene and experimental approach. The data were analyzed using the 2^−ΔΔCq^ method [74] with GAPDH as the endogenous control. The fold change was calculated using the negative logarithm to base 2 of the final value.

### Statistics

GraphPad Prism 7 was used for statistical analysis. Unless otherwise stated, the Mann-Whitney test or Kruskal-Wallis Test was used for statistical analysis. Multiple testing was adjusted by the Benjamini, Krieger and Yekutieli method. For all experiments, the graphs represent means ± SEM. P values < 0.05 were considered to indicate statistical significance and are marked with an asterisk. Two asterisks indicate P values < 0.01 and three asterisks indicate P values < 0.001.

## Electronic supplementary material

Below is the link to the electronic supplementary material.

Supplementary Material 1

Supplementary Material 2

Supplementary Material 3

Supplementary Material 4

## Data Availability

The RNA sequencing dataset supporting the conclusions of this article is available in the NCBI Gene Expression Omnibus with the accession number GSE234142. Full unedited blots supporting the western blot data can be found in the supplementary information files.

## Abbreviations

AMD: Age-related macular degeneration
(P)DR: (Proliferative) diabetic retinopathy
gp130: Glycoprotein130 receptor
STAT3: Signal transducer and activator of transcription 3
STAT1: Signal transducer and activator of transcription 1
ERK: Extracellular-signal regulated kinases
VEGF: Vascular endothelial growth factor
IL-6: Interleukin 6
IL-11: Interleukin 11
(s)IL-11Rα: (Soluble) Interleukin 11 receptor subunit alpha
CNTF: Ciliary neurotrophic factor
OSM: Oncostatin M
JAK: Janus kinase
HUVEC: Human umbilical vein endothelial cell
HRMVEC: Human retinal microvascular endothelial cell
EGM: Endothelial growth medium
EBM: Endothelial basal medium
Diff: Differential medium
GSEA: Gene Set Enrichment Analysis
DEG: Differentially expressed genes
NES: Normalized enrichment score
GO: Gene Ontology
TIMP1: TIMP metallopeptidase inhibitor 1
WARS1: Tryptophanyl-tRNA synthetase 1
EMCN: Endomucin
SAT1: Spermidine/spermine N1-acetyltransferase 1
TFPI2: Tissue factor pathway inhibitor 2
PDGFRA: Platelet derived growth factor receptor alpha
APLNR: Apelin receptor
HGF: Hepatocyte growth factor
ANGPTL2: Angiopoietin like 2
CXCL10: C-X-C motif chemokine ligand 10
PEDF: Pigment epithelium-derived factor
TGF-ß2: Transforming growth factor, beta 2
HIF-1α: Hypoxia inducible factor 1 subunit alpha
PAI-1: Plasminogen activator inhibitor-1
Id2: Inhibitor of differentiation 2
OIR: Oxygen in retinopathy
NV: Neovascularization
VO: Vasoobliteration
GFP: Green fluorescent protein
GFAP: Glial fibrillary acidic protein
siRNA: Small interfering RNA
PFA: Paraformaldehyde
ELISA: Enzyme-linked Immunosorbent Assay
