# Supplementary material for: Characterization of the angiomodulatory effects of Interleukin 11 cis- and trans-signaling in the retina
Source: J Neuroinflammation. 2024 Sep 18;21:230. doi: 10.1186/s12974-024-03223-3 (PMC11412048; doi:10.1186/s12974-024-03223-3)

Full unedited blot for Figure 3B + Suppl. Fig. S2F + Suppl. Fig. S3A   #1

pSTAT3 Tyr

79, 86 kDa

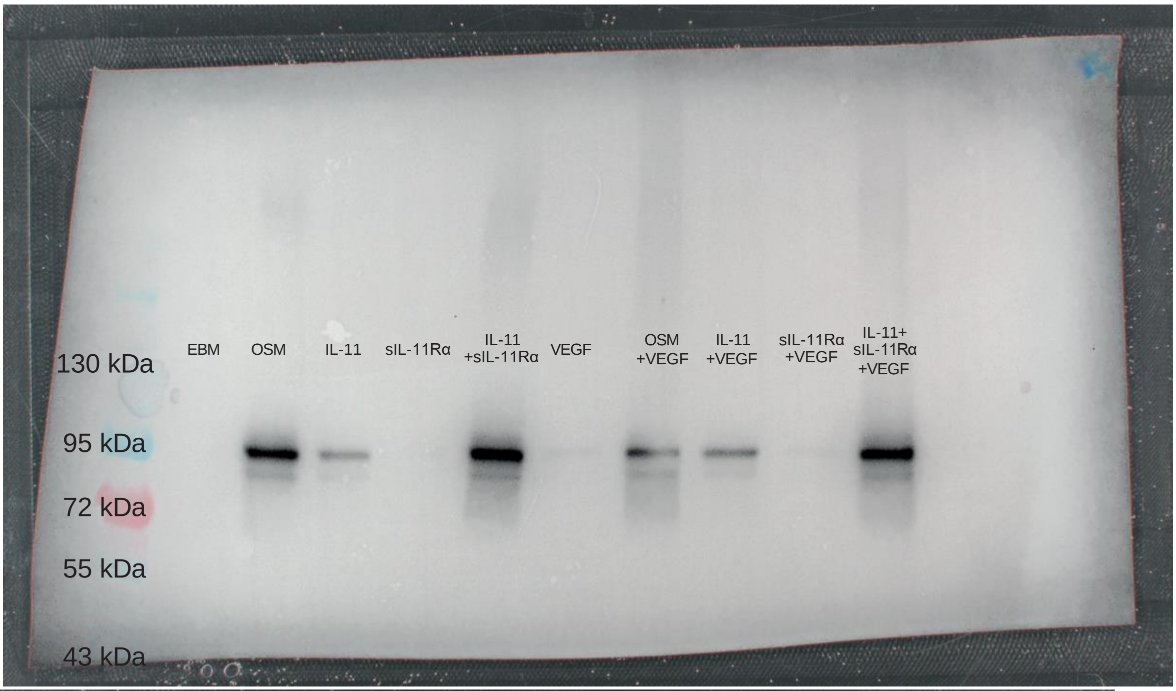

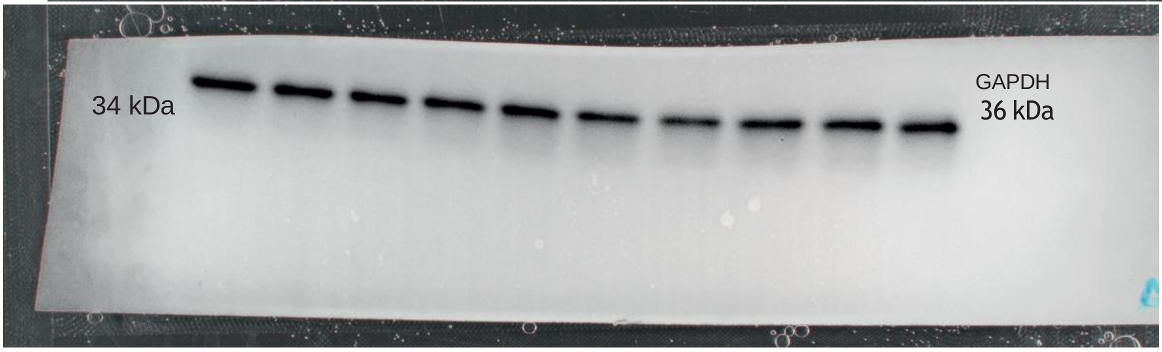

GAPDH

36 kDa

Full unedited blot for Figure 3B + Suppl. Fig. S2F + Suppl. Fig. S3A #1

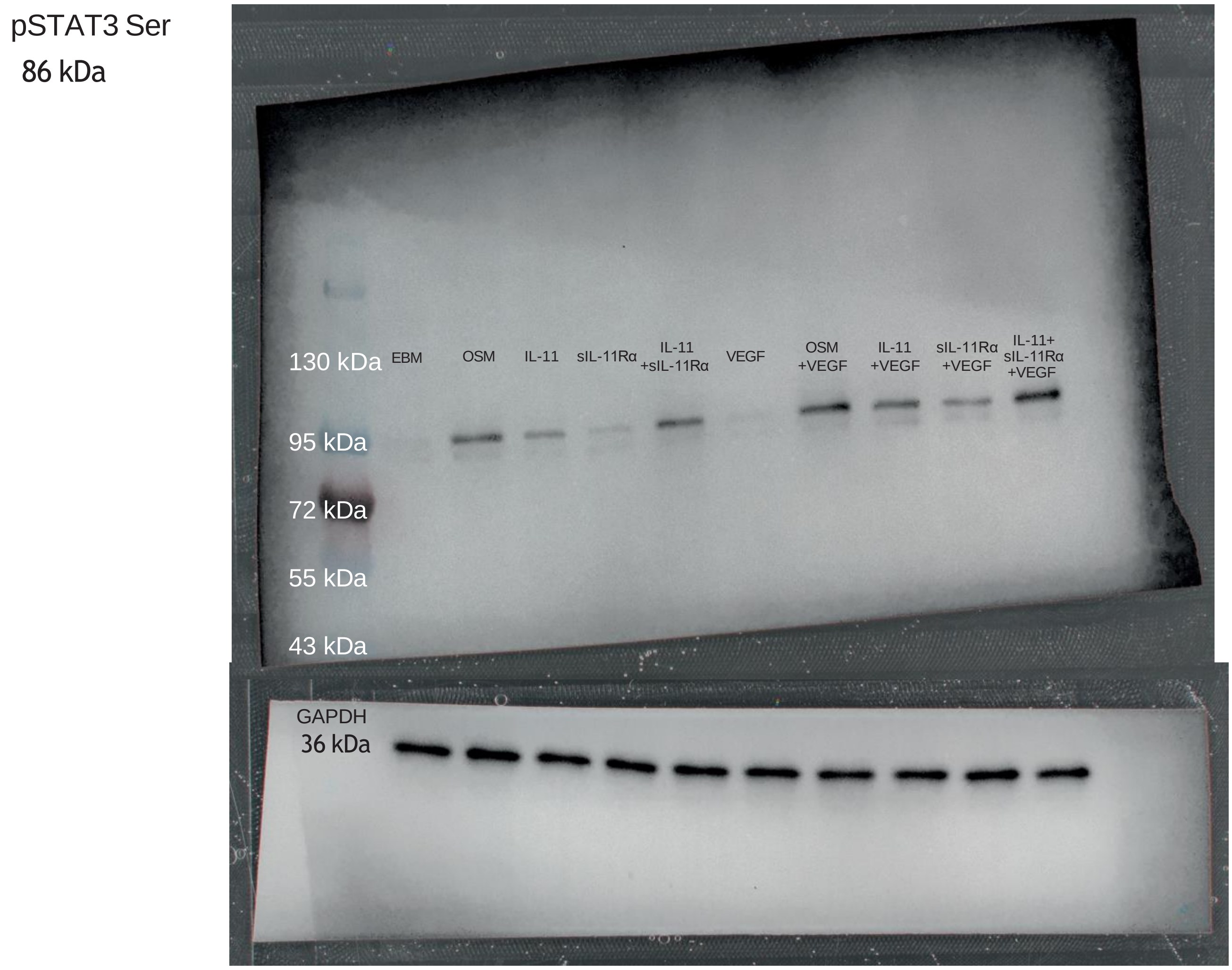

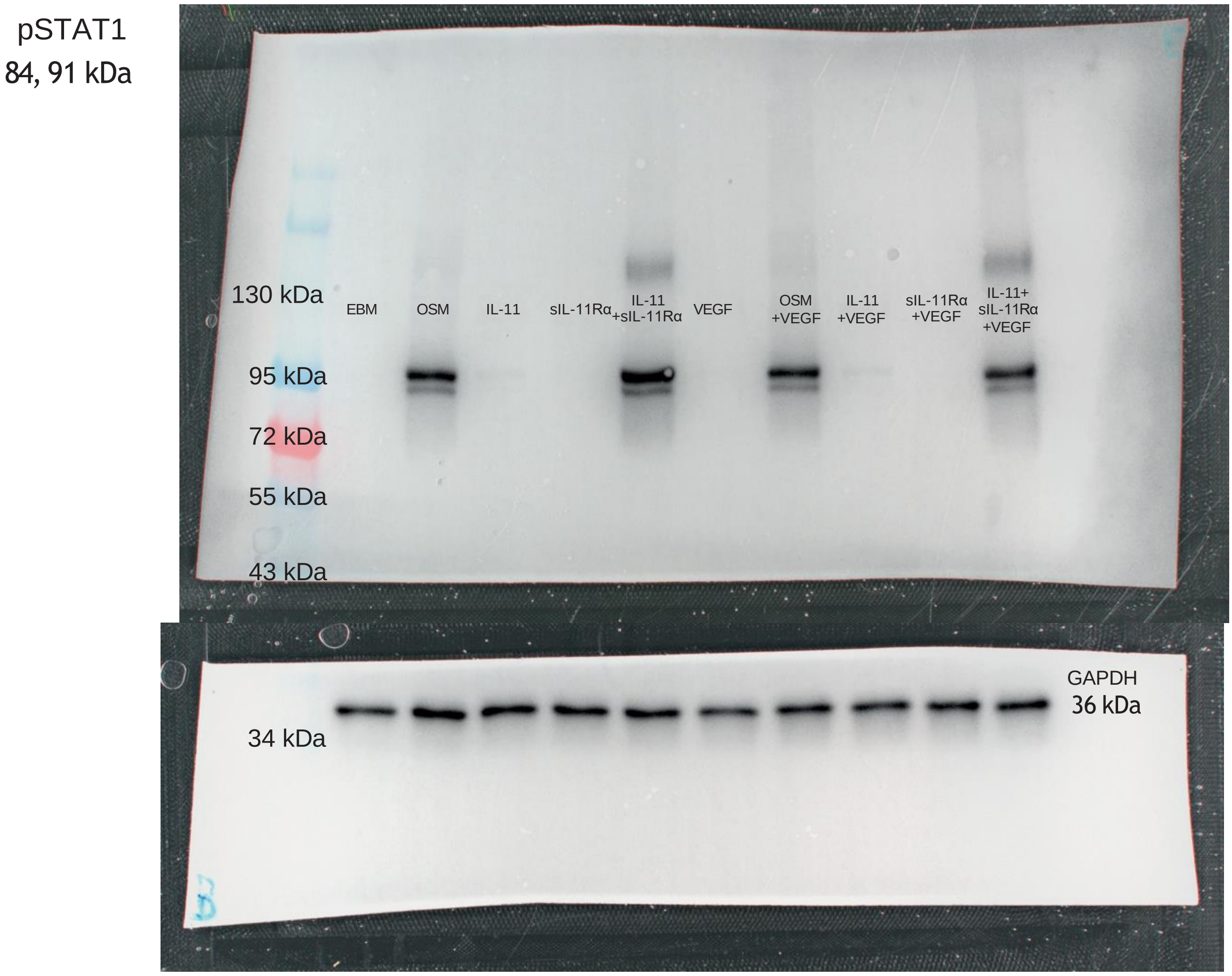

Full unedited blot for Figure 3B + Suppl. Fig. S2F + Suppl. Fig. S3A #1

pAkt

60 kDa

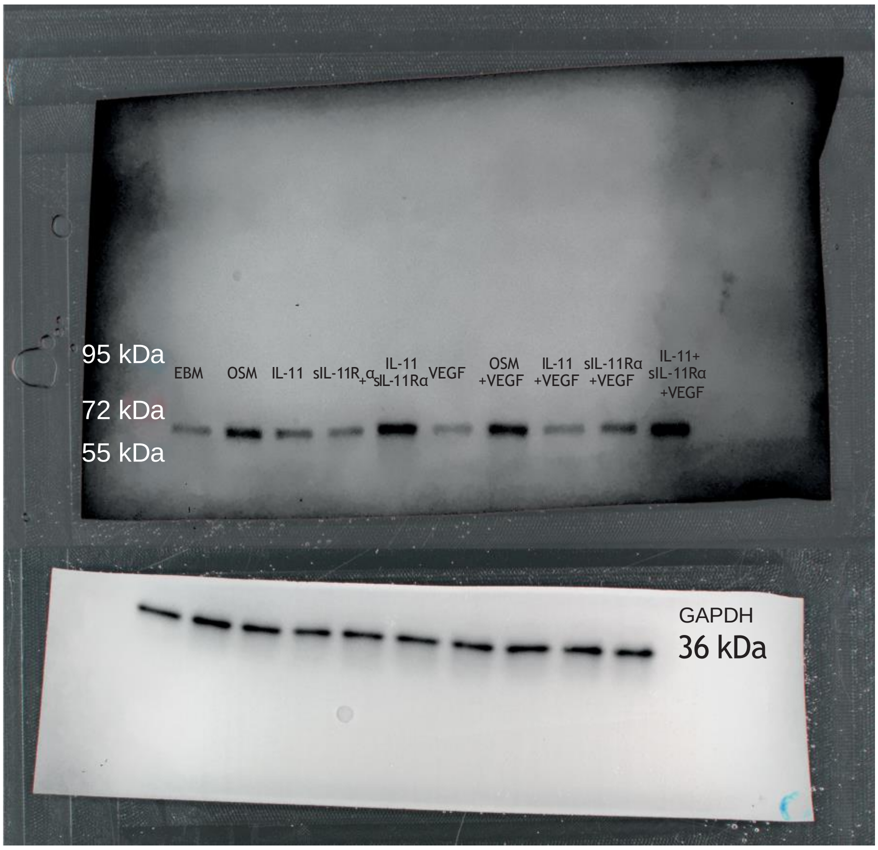

pERK 44, 42 kDa

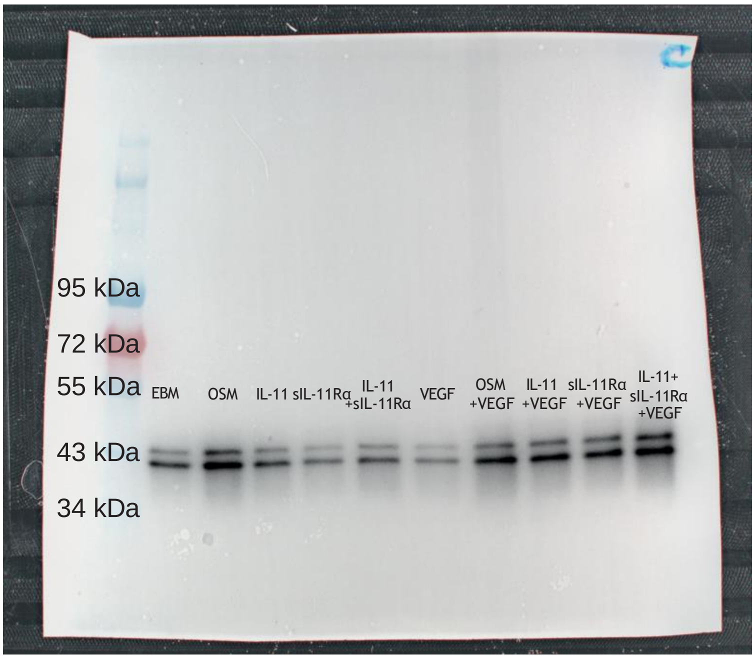

GAPDH

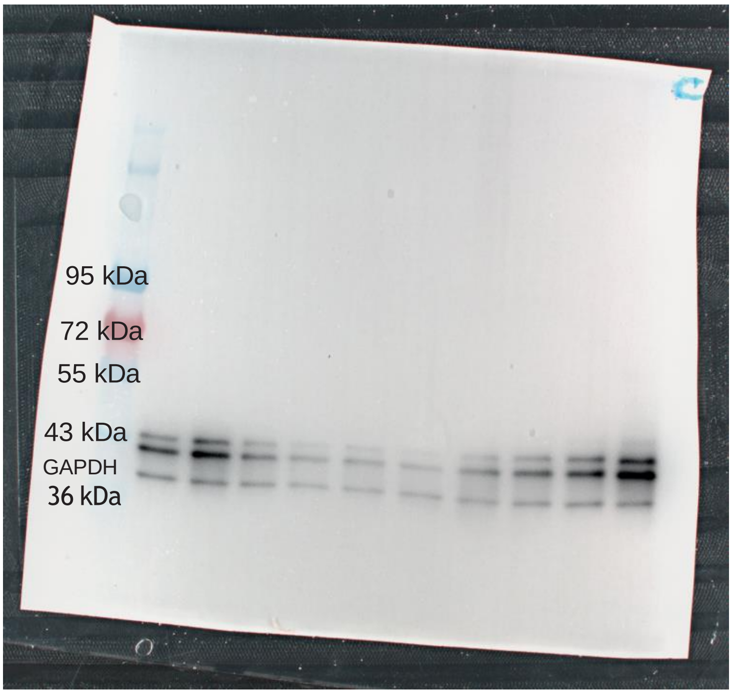

Full unedited blot for Figure 3B + Suppl. Fig. S2F + Suppl. Fig. S3A #2

pSTAT3 Tyr

79, 86 kDa

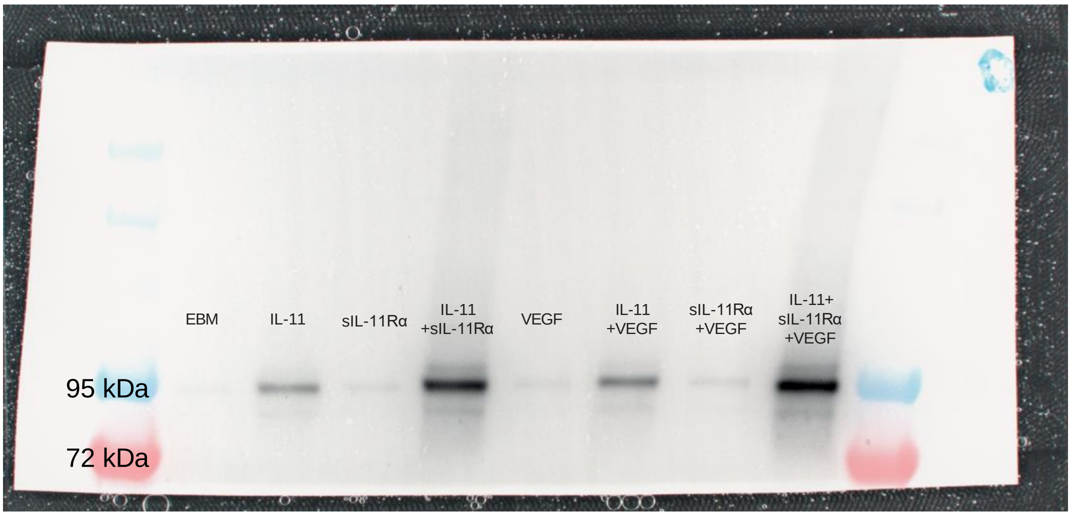

pERK

44, 42 kDa

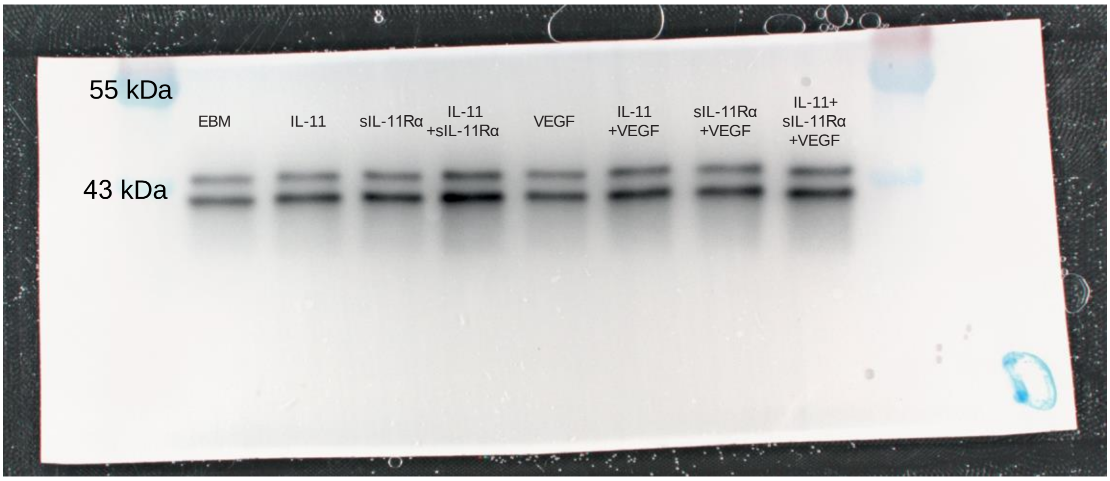

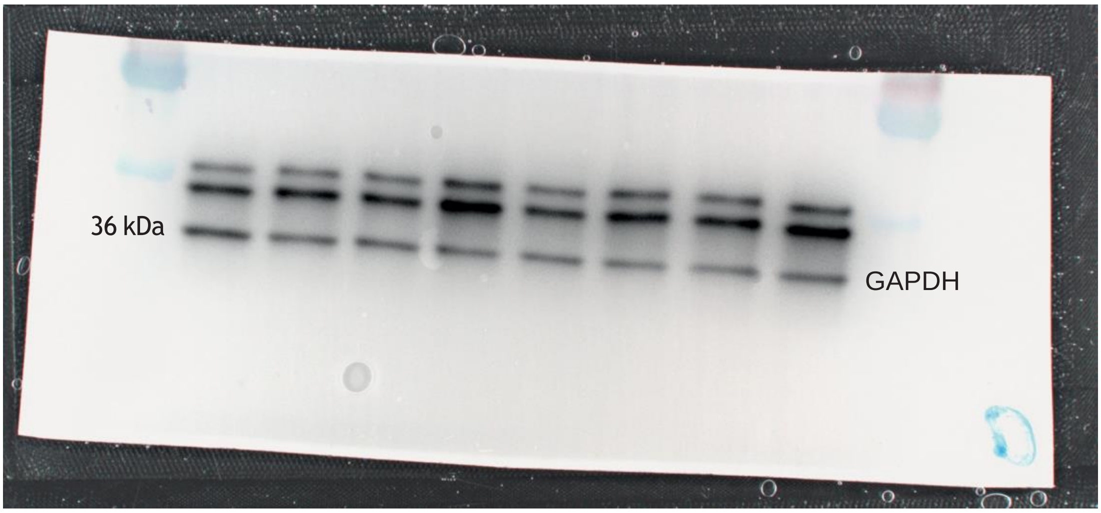

Full unedited blot for Figure 3B + Suppl. Fig. S2F + Suppl. Fig. S3A   #2

pSTAT1

84, 91 kDa

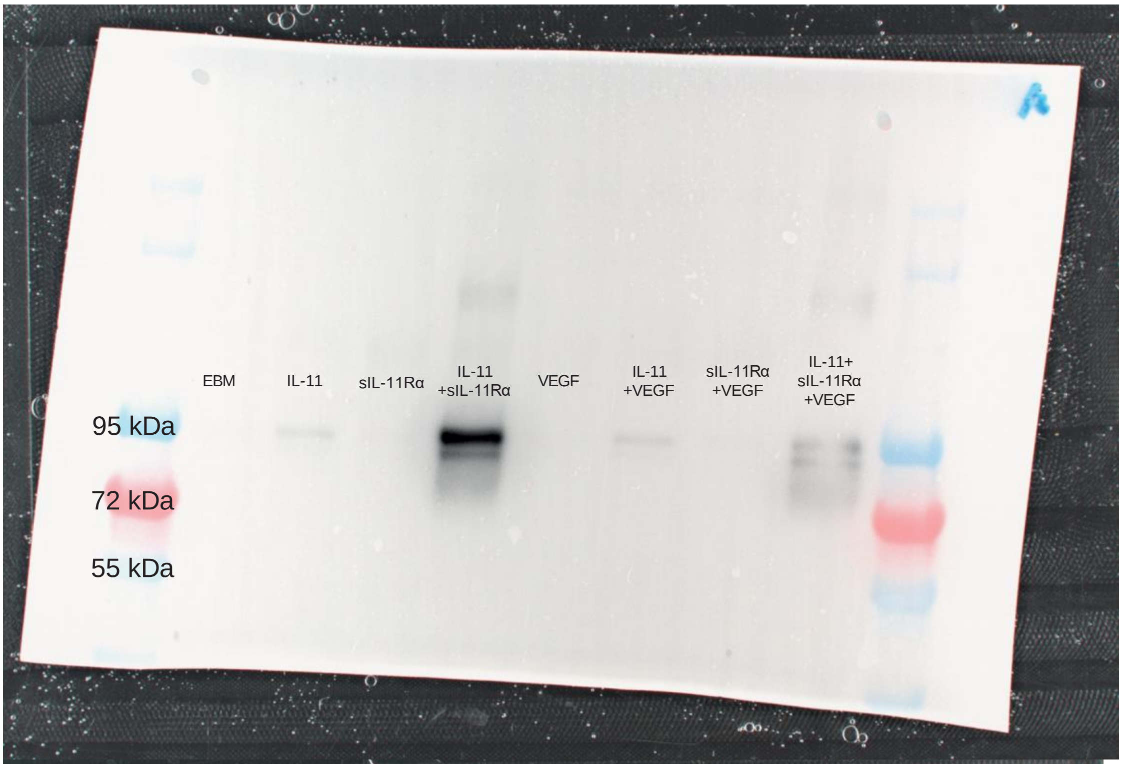

GAPDH

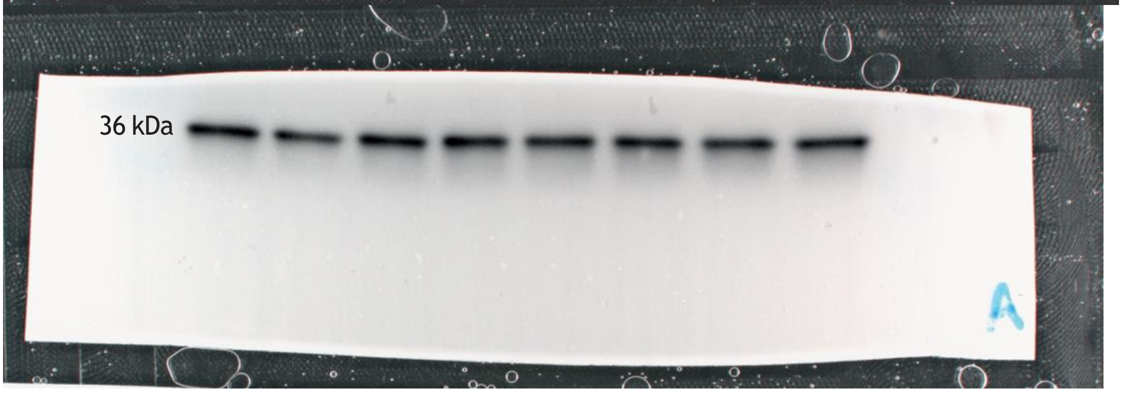

pSTAT3 Ser

86 kDa

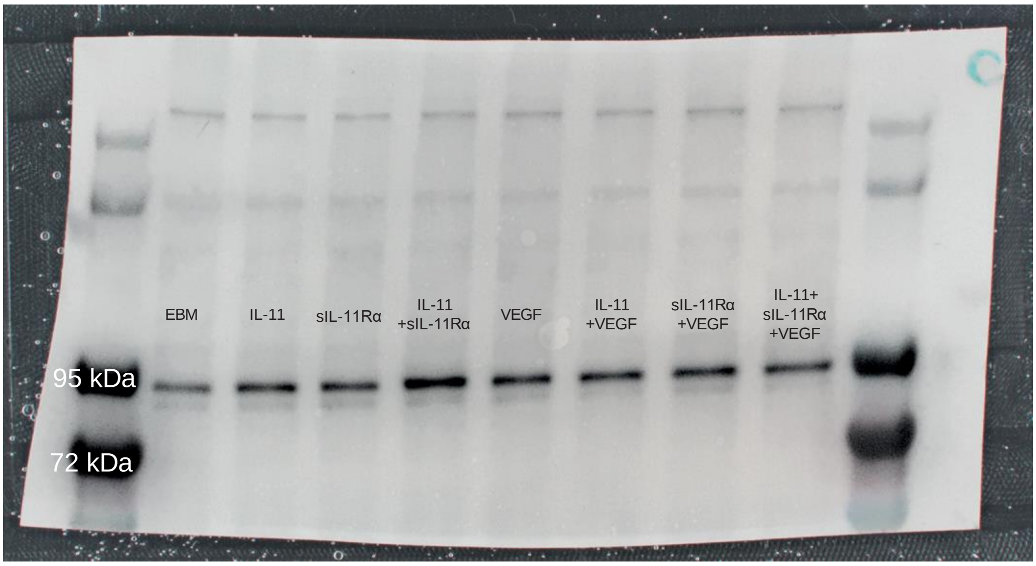

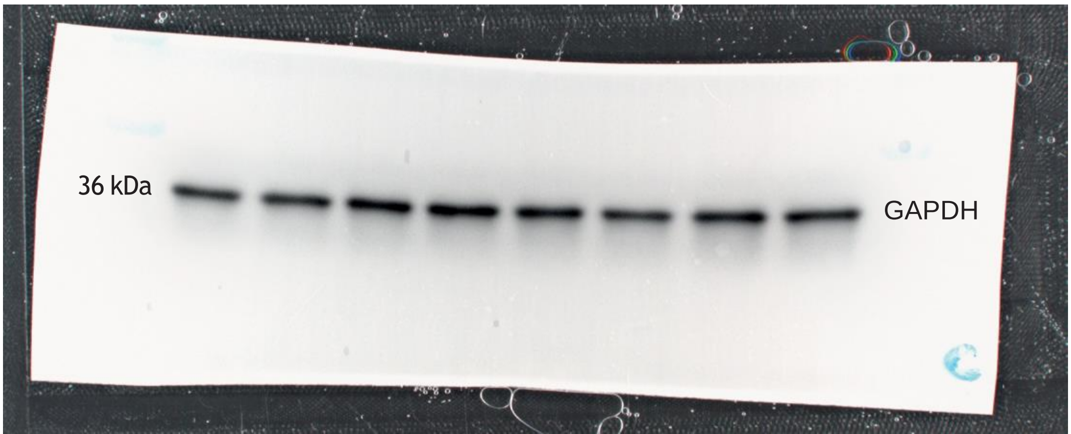

Full unedited blot for Figure 3B + Suppl. Fig. S2F + Suppl. Fig. S3A #2

pSTAT5

90 kDa

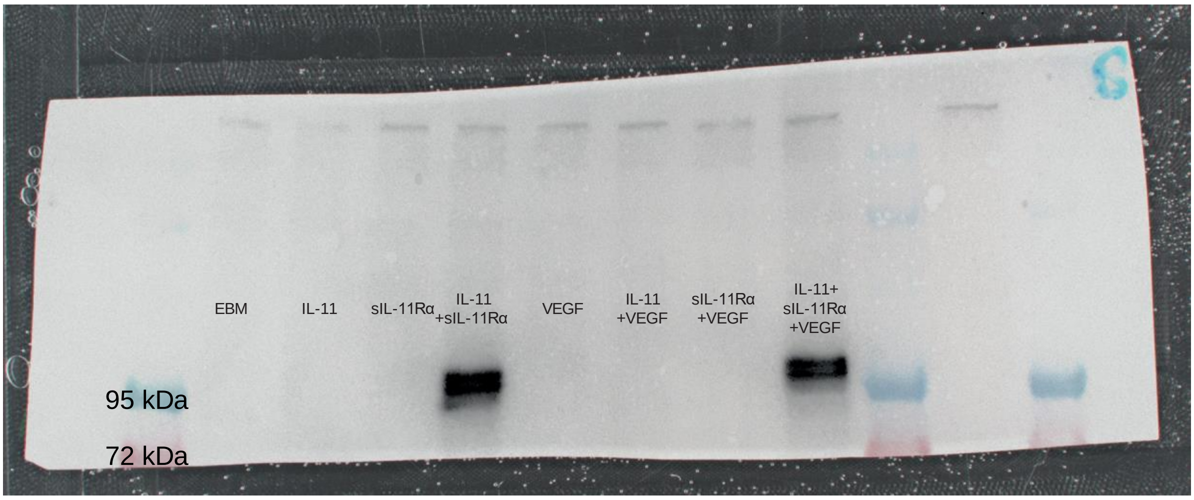

pAkt

60 kDa

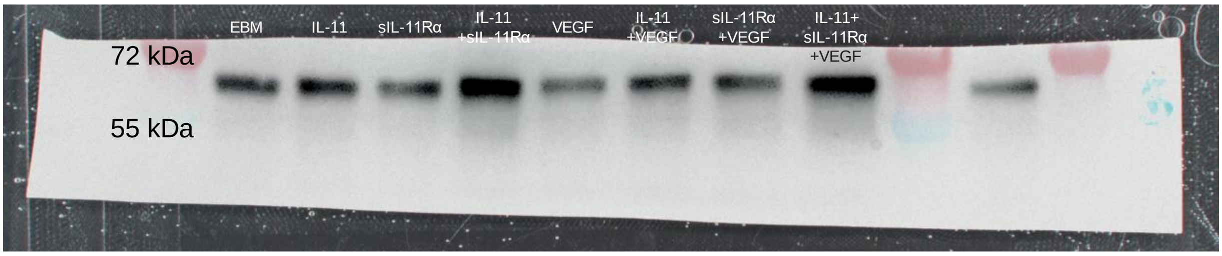

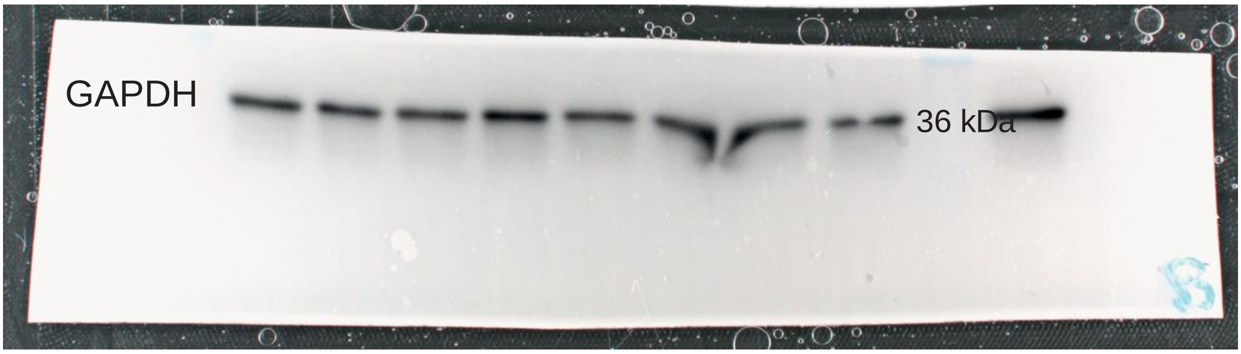

Full unedited blot for Figure 3B + Suppl. Fig. S2F + Suppl. Fig. S3A #3

pSTAT3 Tyr

79, 86 kDa

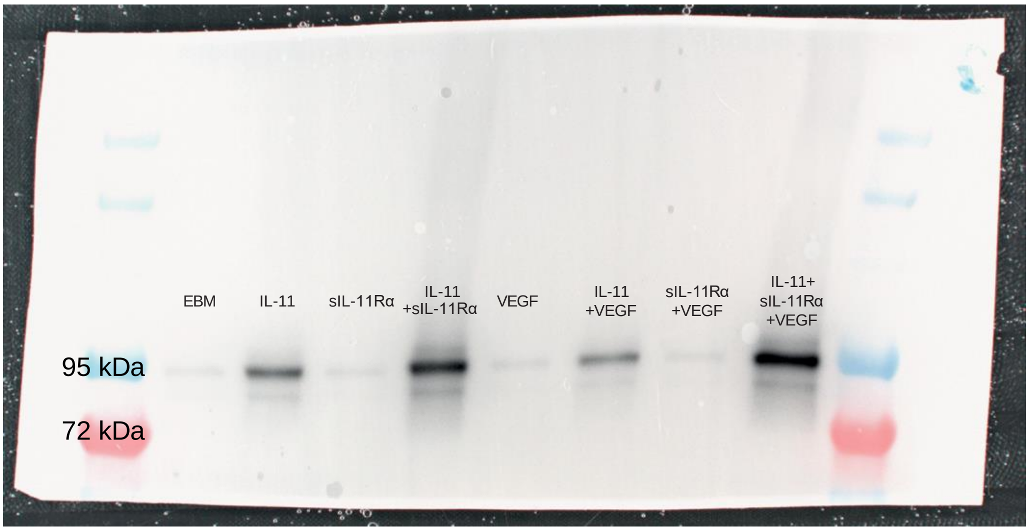

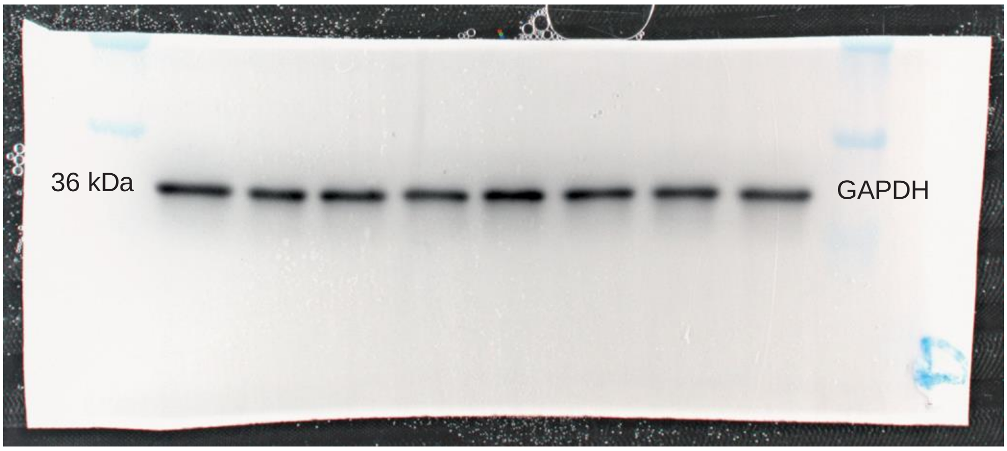

pSTAT3 Ser

86 kDa

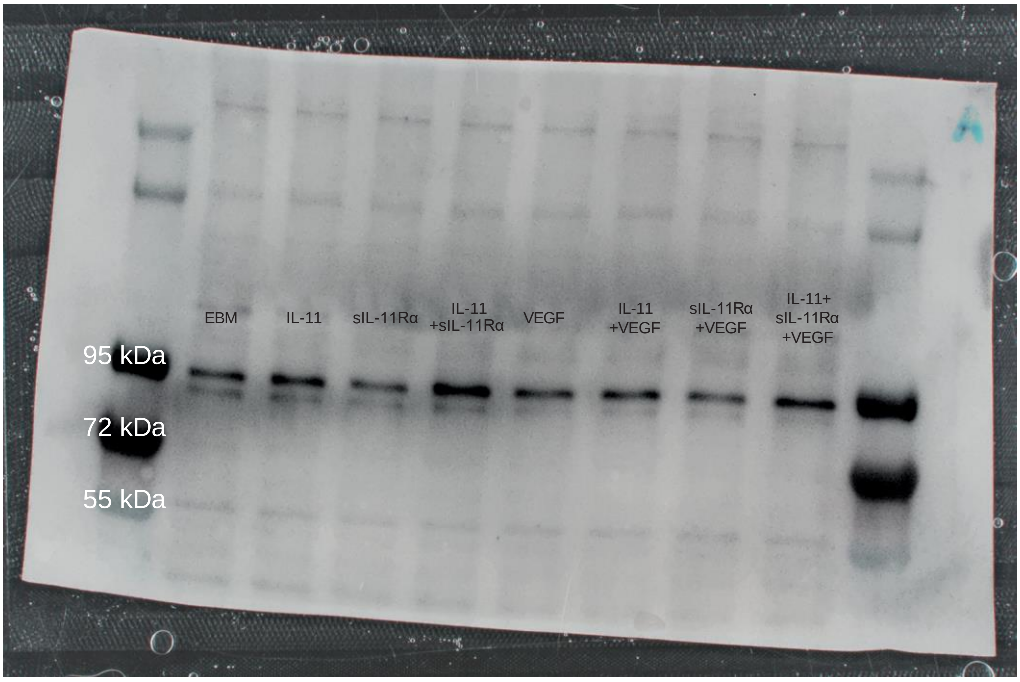

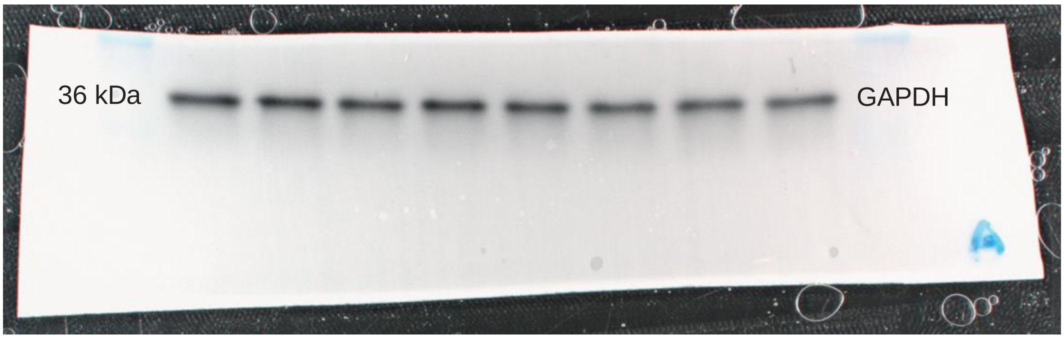

Full unedited blot for Figure 3B + Suppl. Fig. S2F + Suppl. Fig. S3A #3

pSTAT1

84, 91 kDa

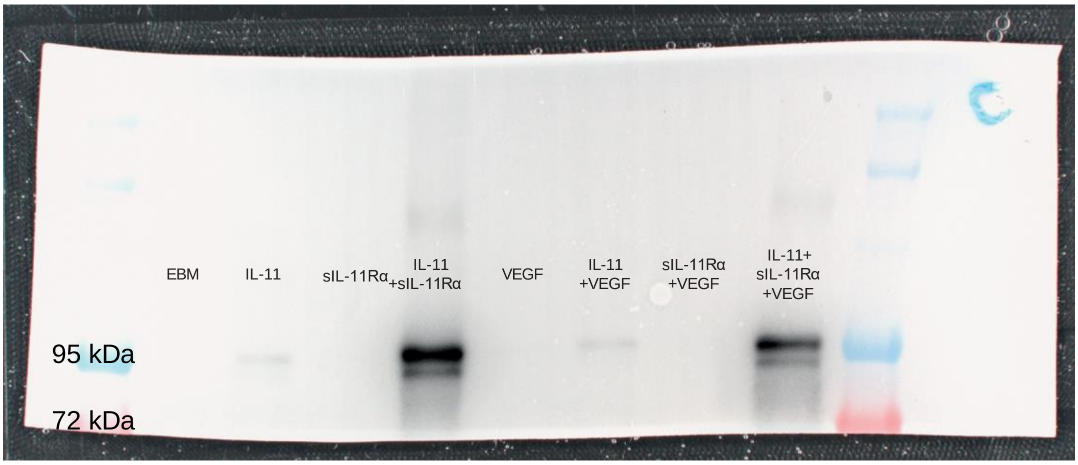

pERK

44, 42 kDa

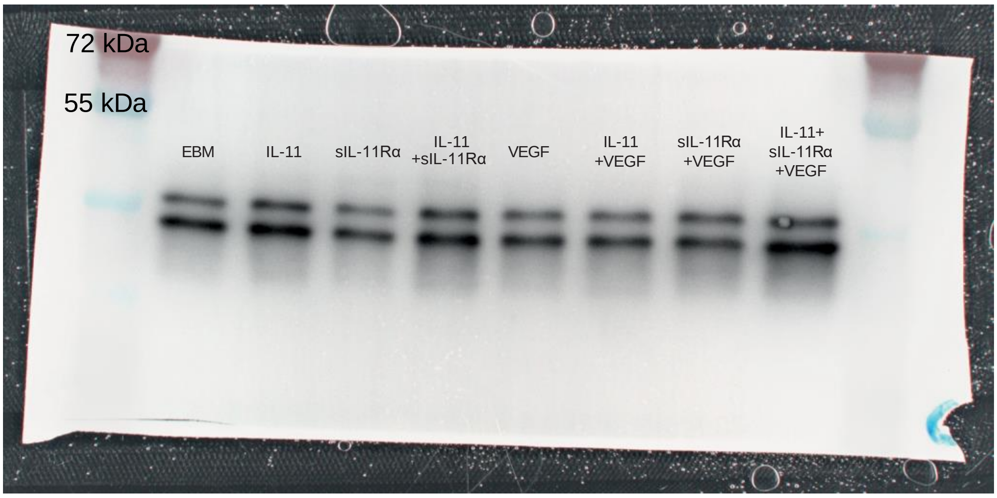

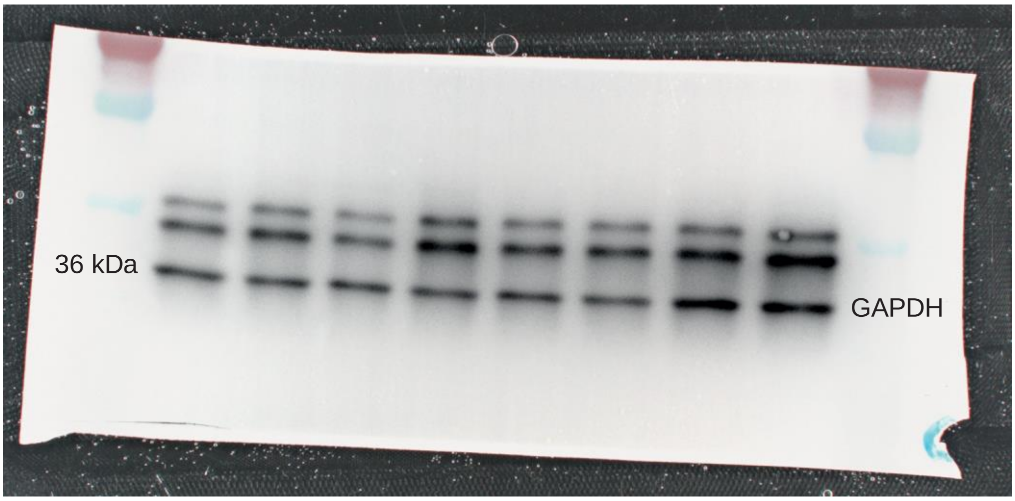

Full unedited blot for Figure 3B + Suppl. Fig. S2F + Suppl. Fig. S3A #3

pSTAT5

90 kDa

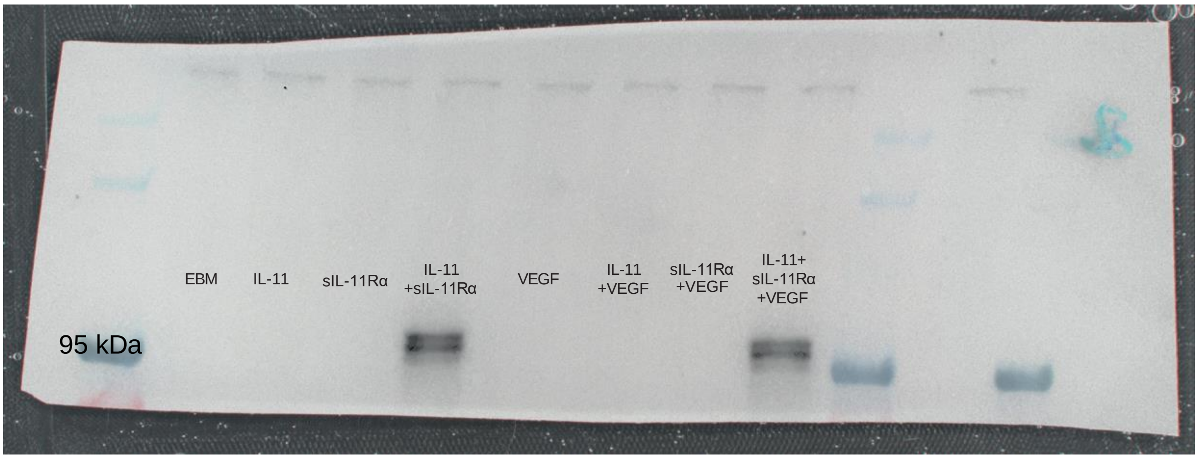

pAkt

60 kDa

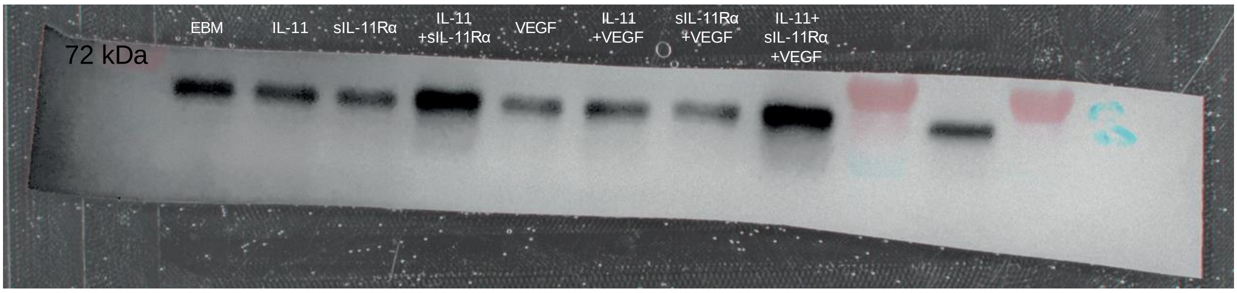

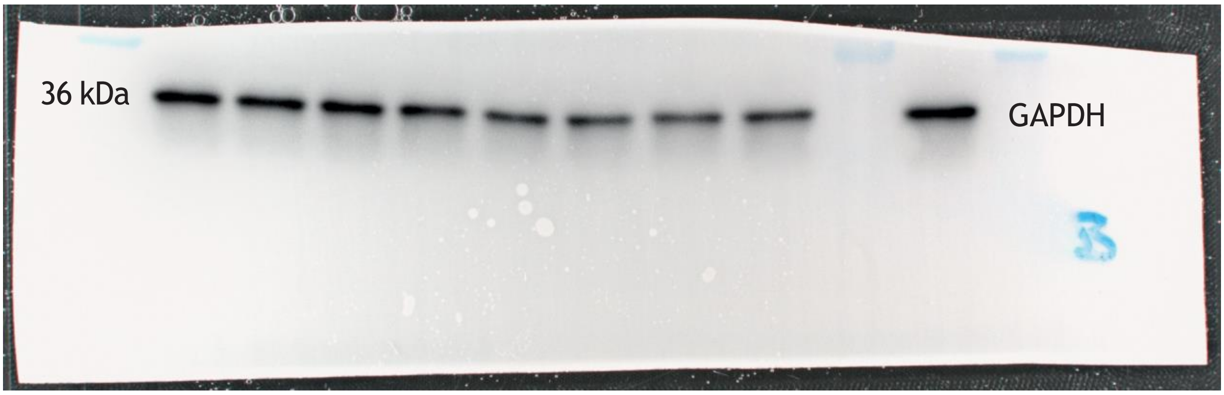

Full unedited blot for Figure 3B + Suppl. Fig. S2F + Suppl. Fig. S3A #4

pSTAT3 Tyr
79, 86 kDa

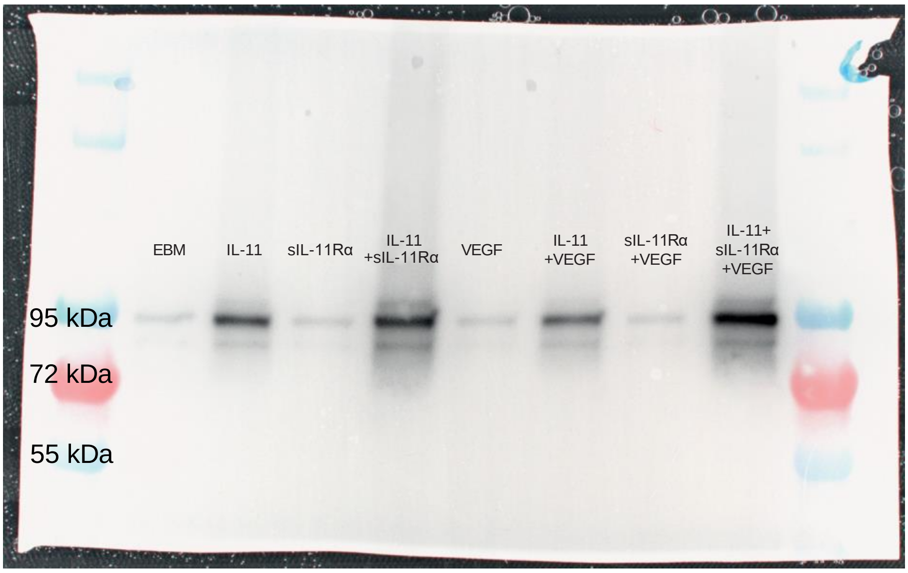

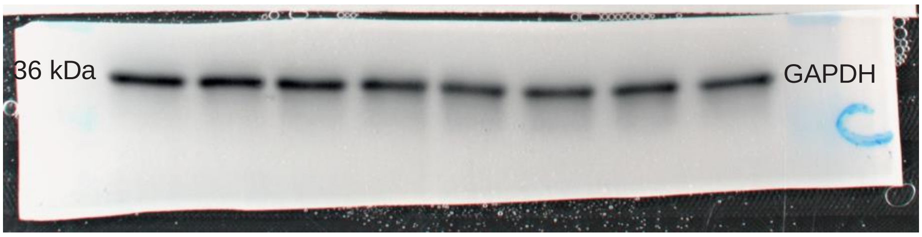

pSTAT3 Ser
86 kDa

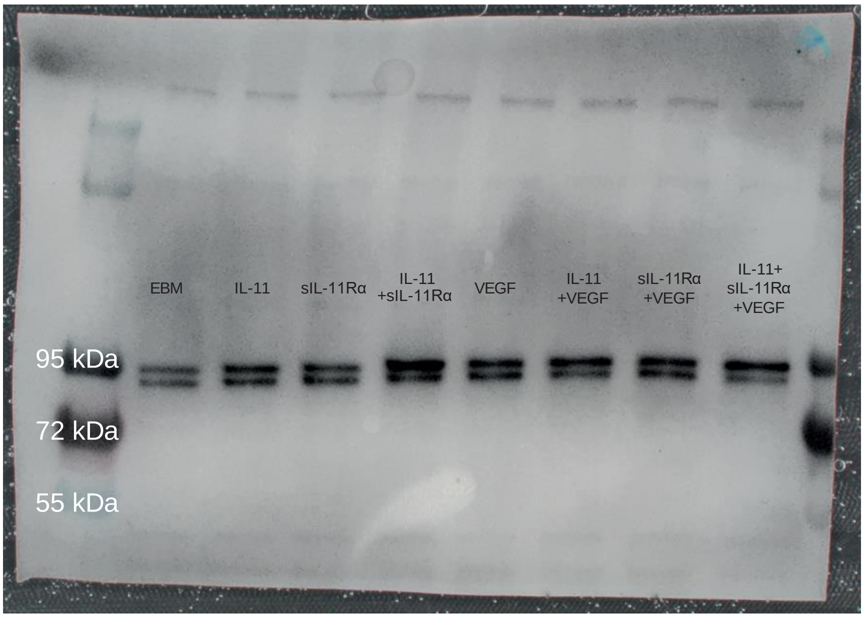

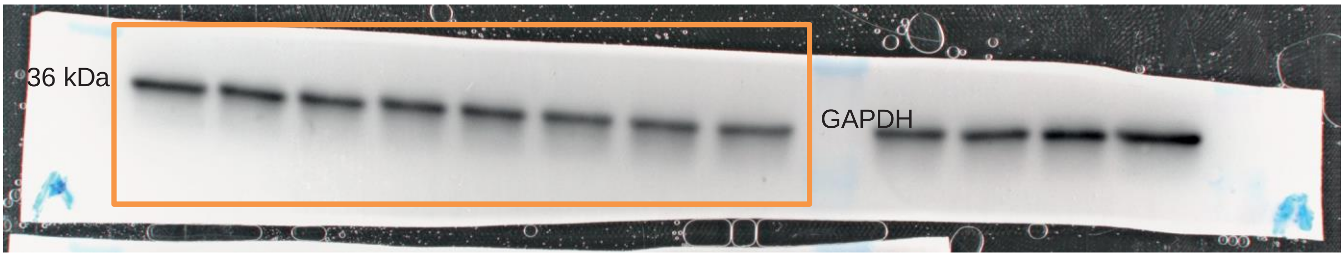

Full unedited blot for Figure 3B + Suppl. Fig. S2F + Suppl. Fig. S3A #4

pSTAT1

84, 91 kDa

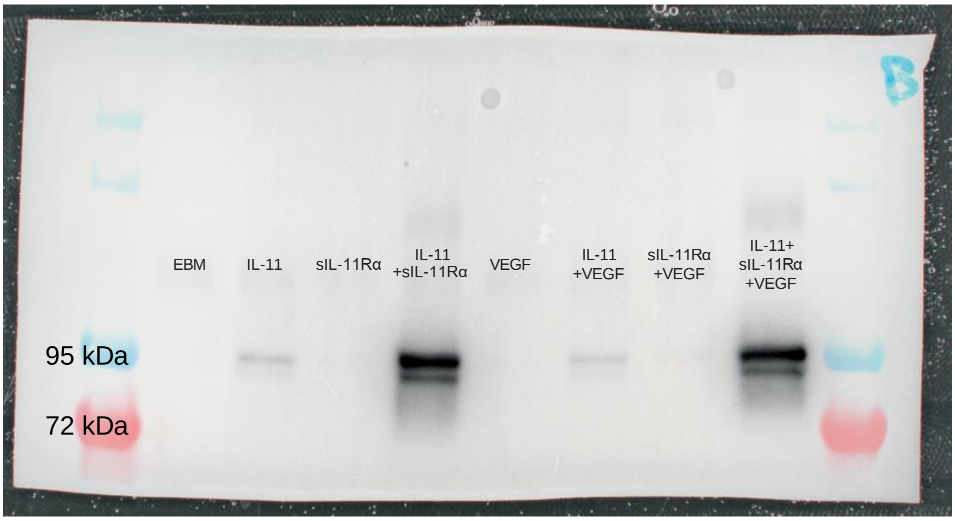

pERK

44, 42 kDa

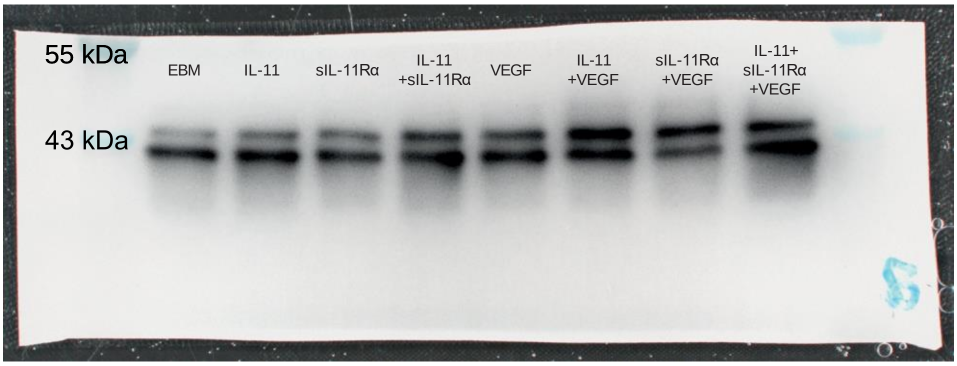

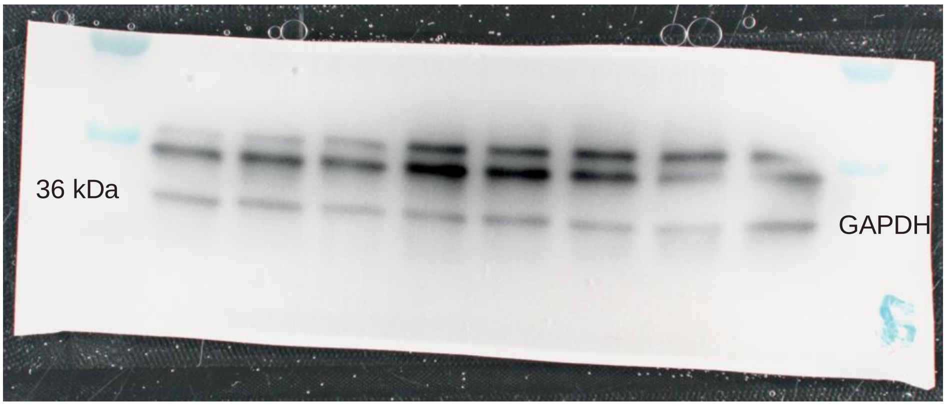

Full unedited blot for Figure 3B + Suppl. Fig. S2F + Suppl. Fig. S3A #4

pSTAT5
90 kDa

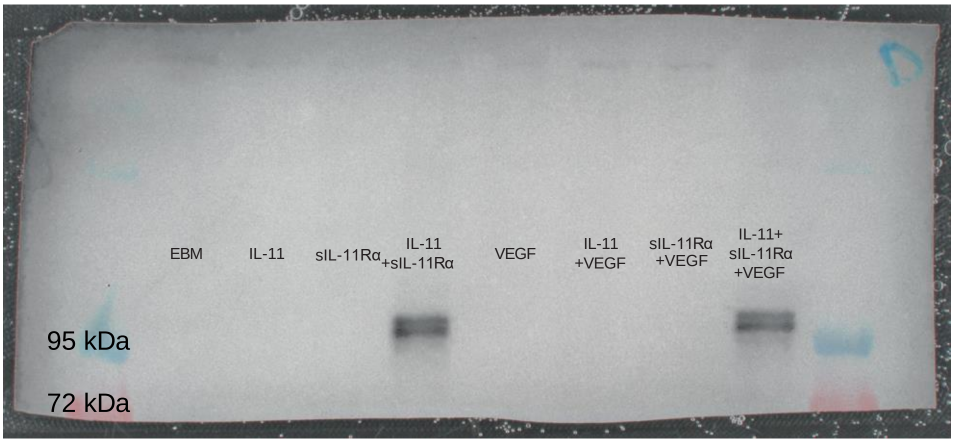

pAkt

60 kDa

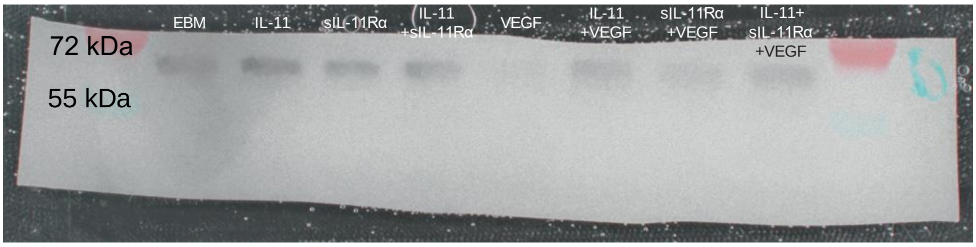

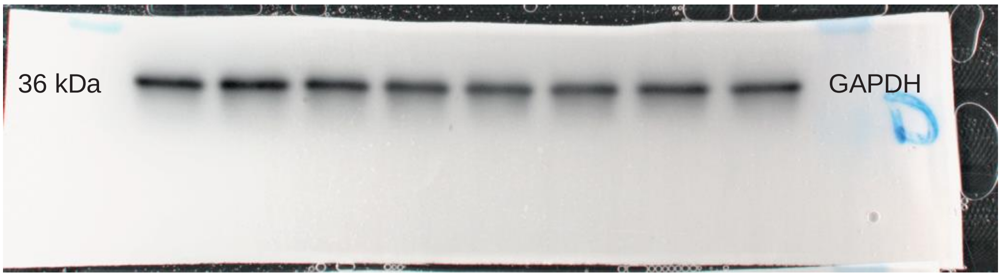

Full unedited blot for Figure 3A + Suppl. Fig. S2E #1

pJAK1

130 kDa

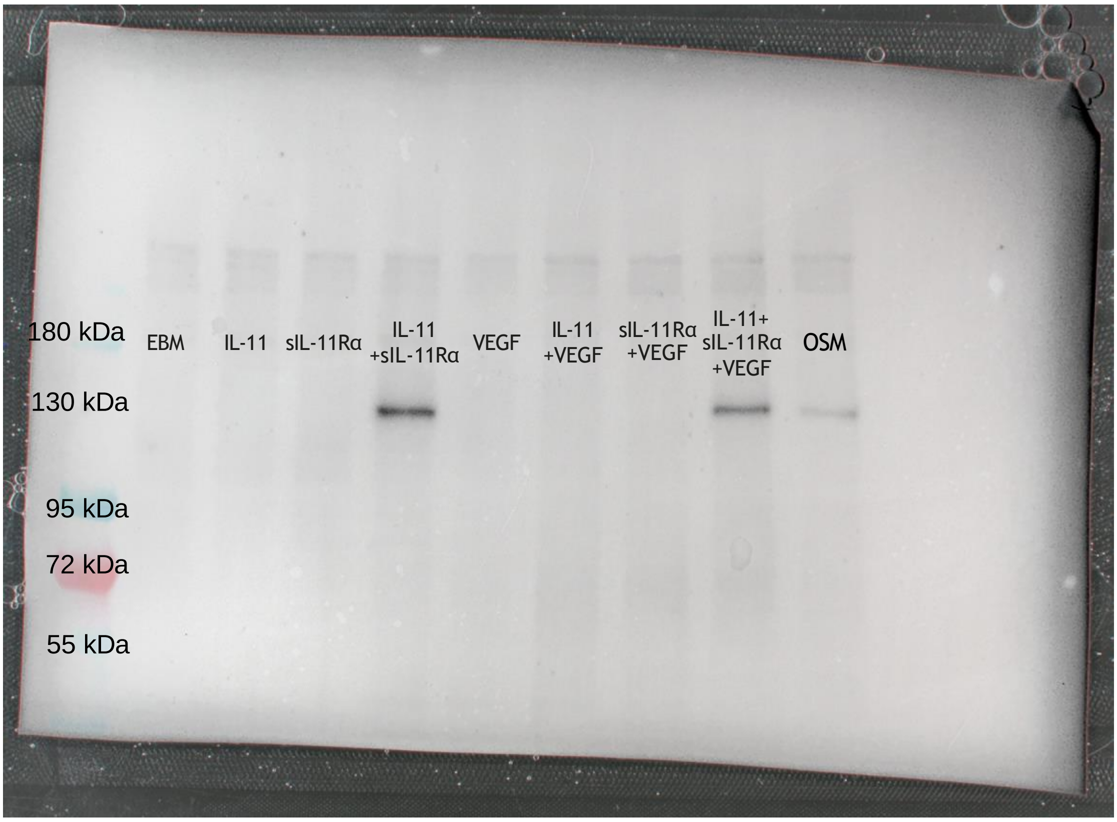

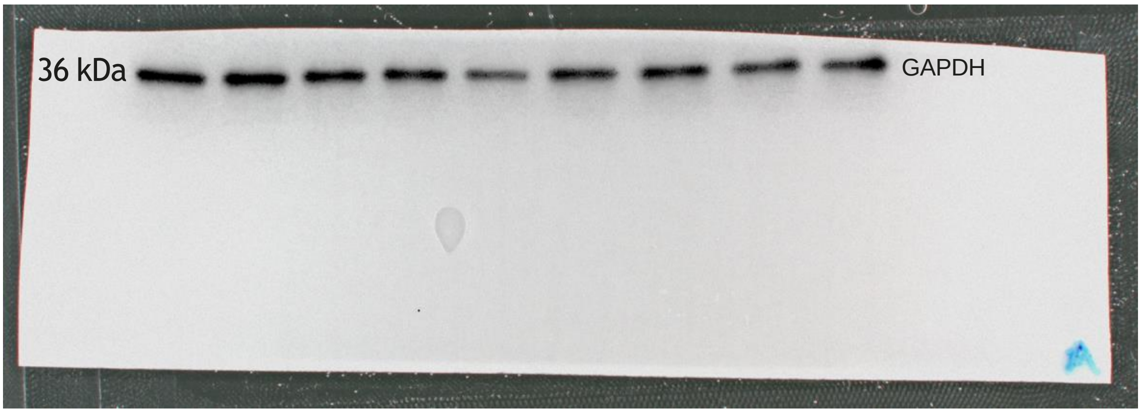

Full unedited blot for Figure 3A + Suppl. Fig. S2E #1

pJAK2
125 kDa

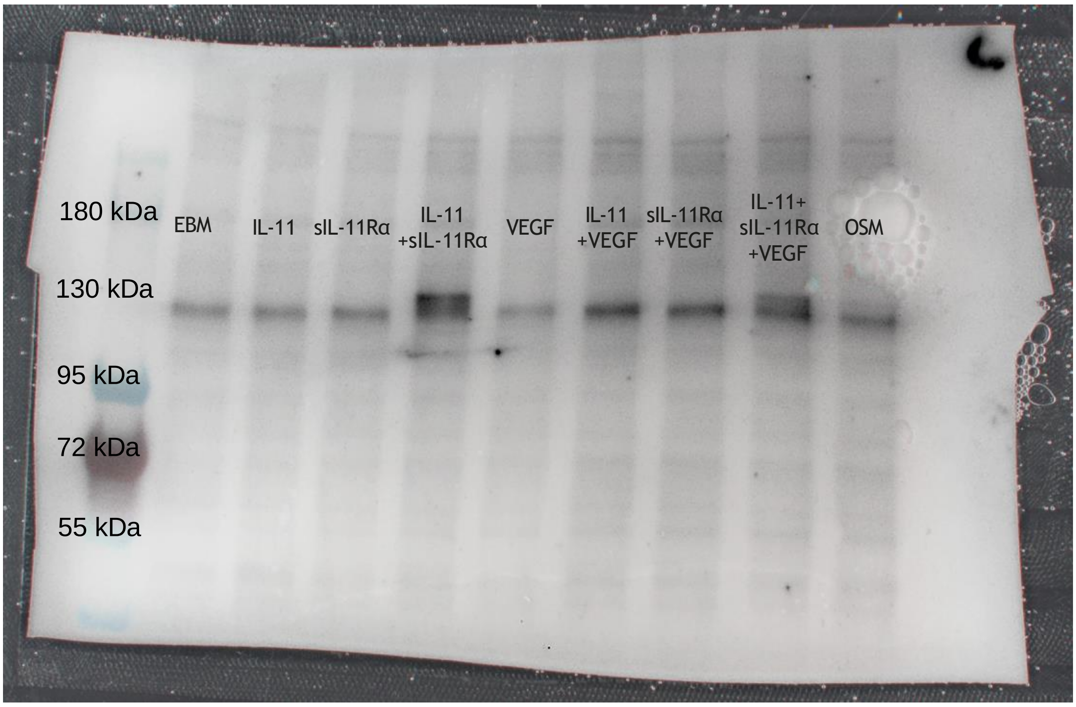

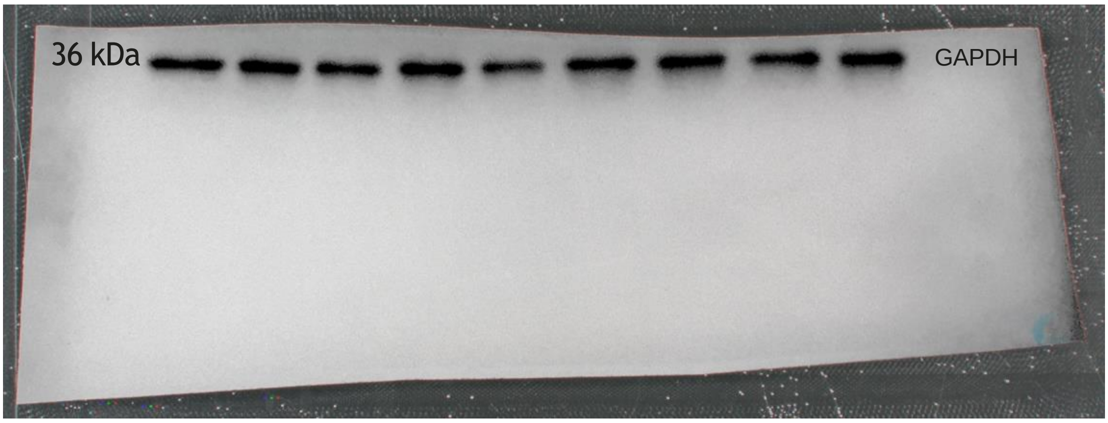

Full unedited blot for Figure 3A + Suppl. Fig. S2E   #1

pTyk2
134 kDa

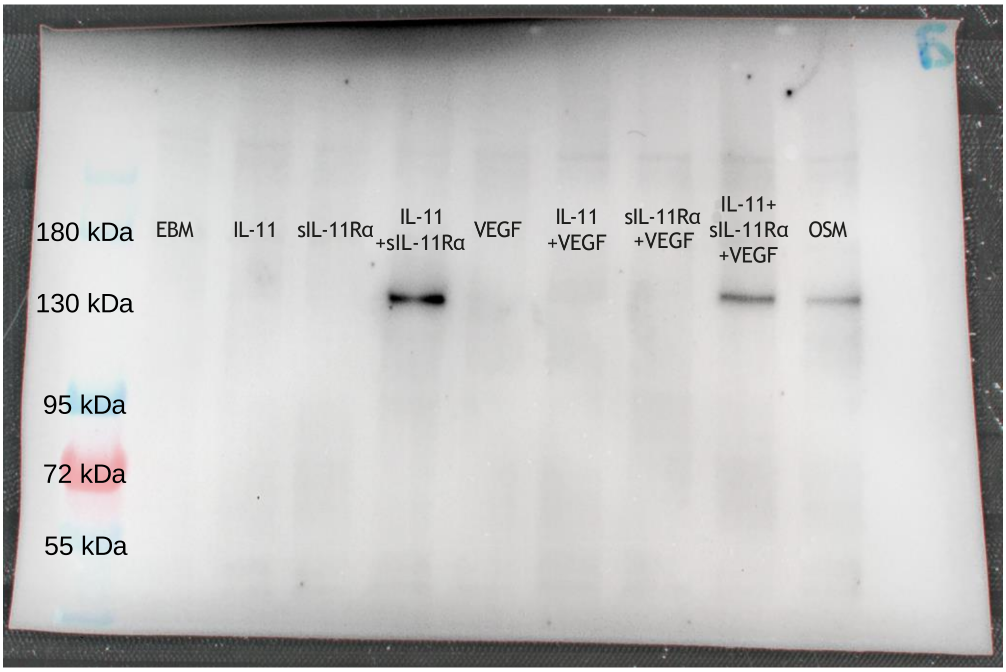

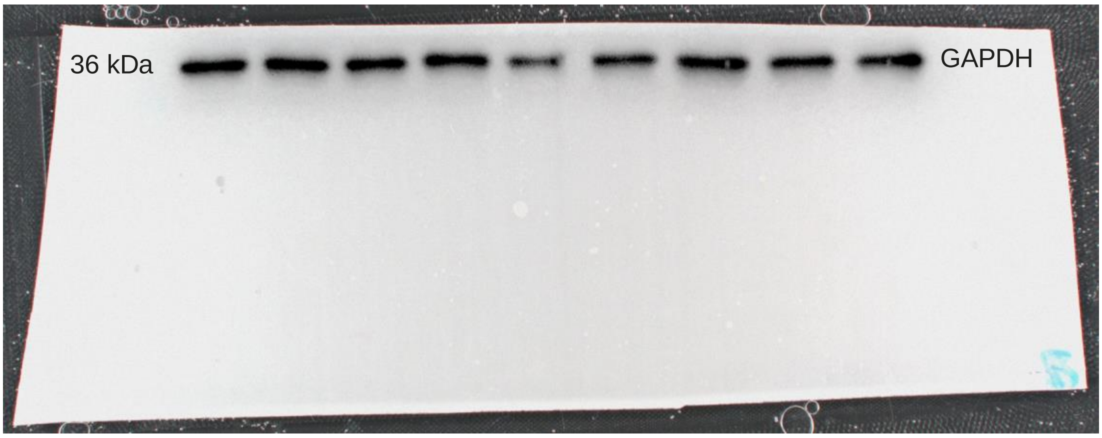

Full unedited blot for Figure 3A + Suppl. Fig. S2E #2

pJAK1

130 kDa

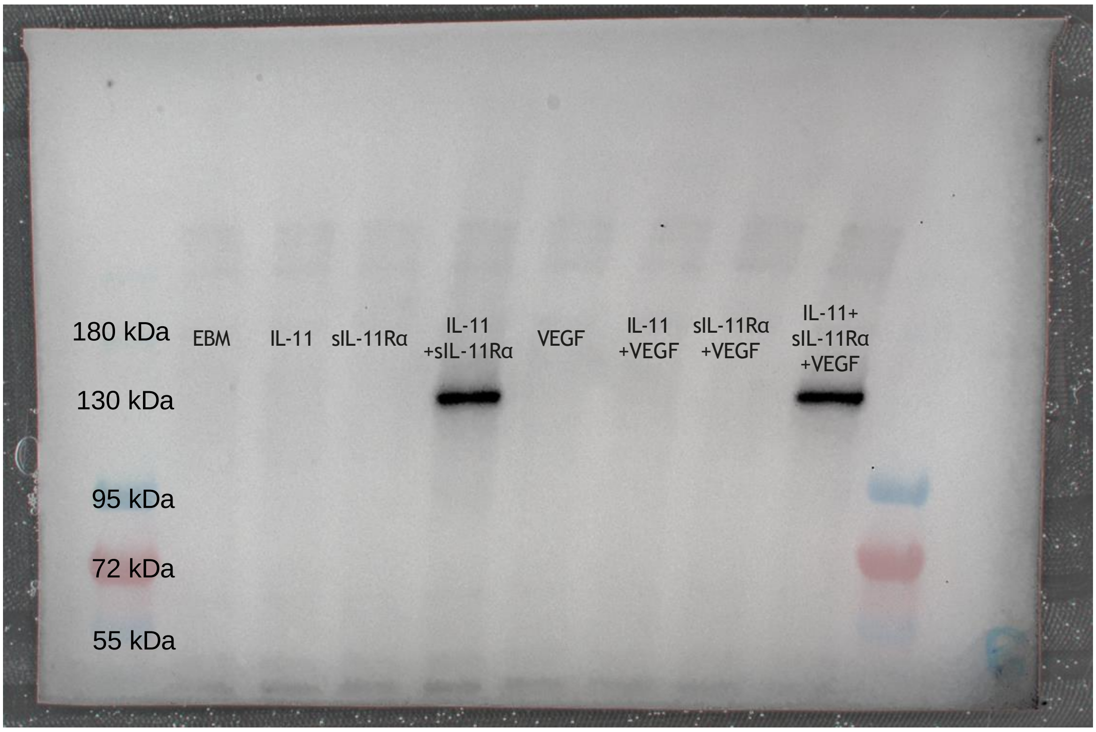

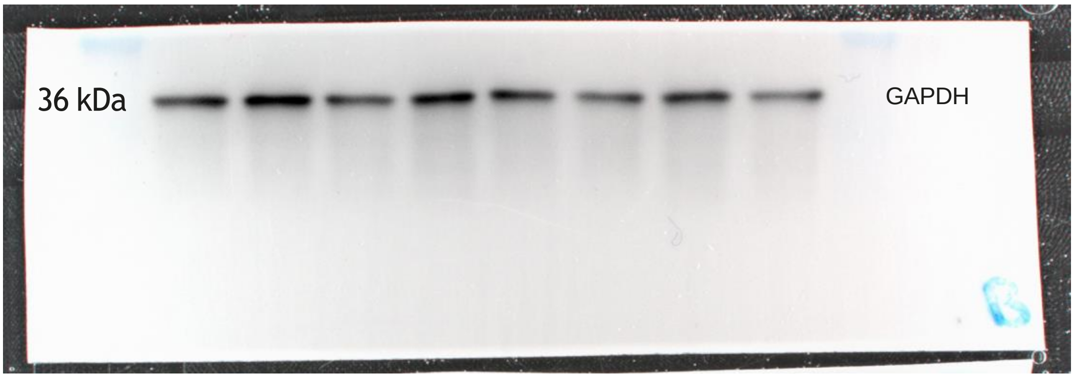

Full unedited blot for Figure 3A + Suppl. Fig. S2E #2

pJAK2

125 kDa

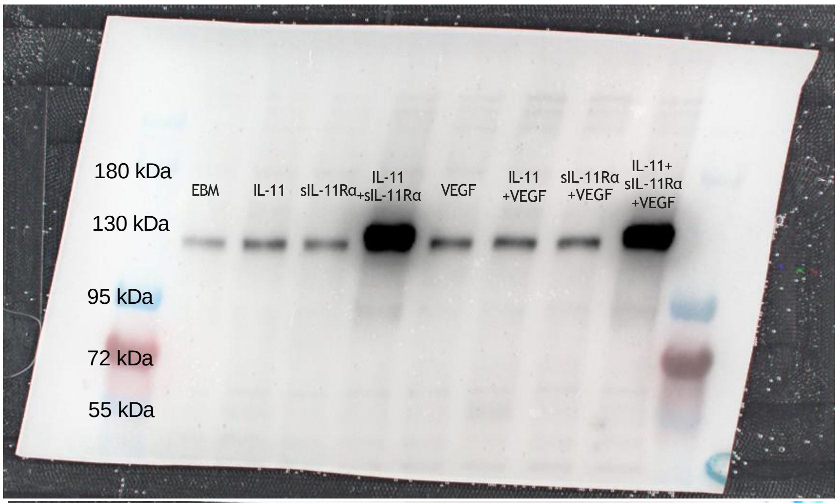

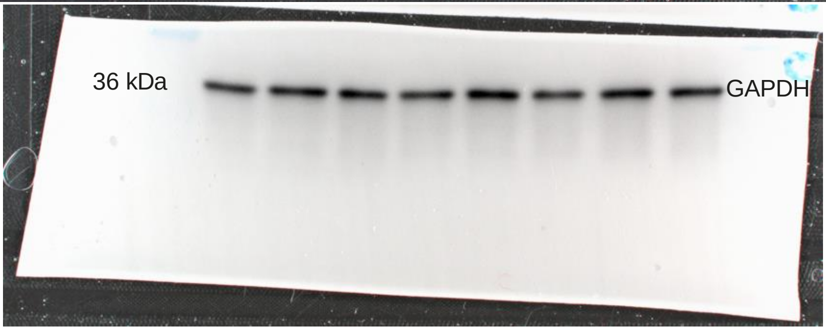

pTyk2

134 kDa

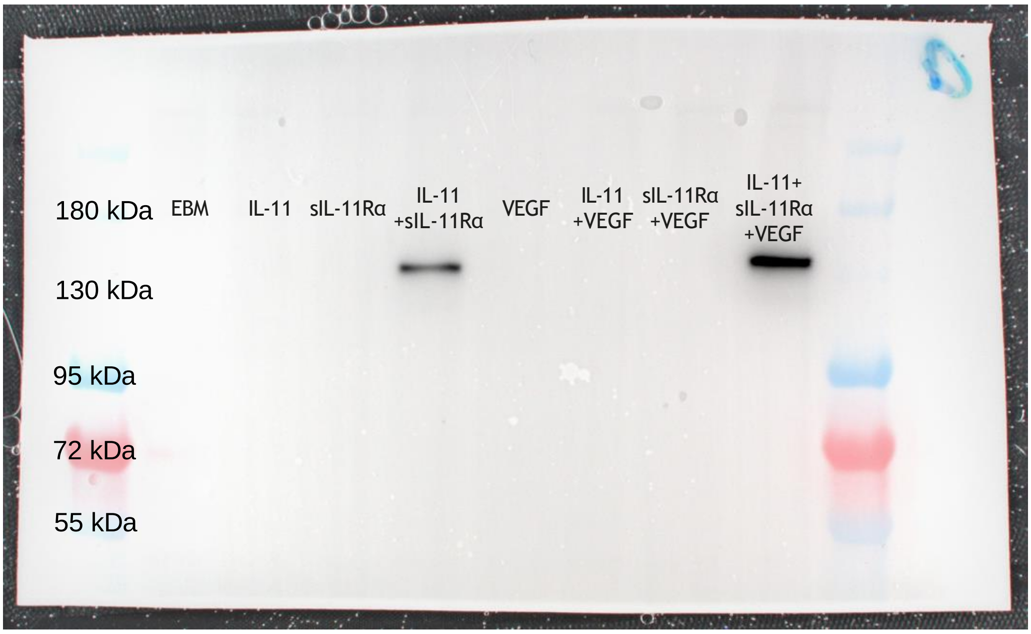

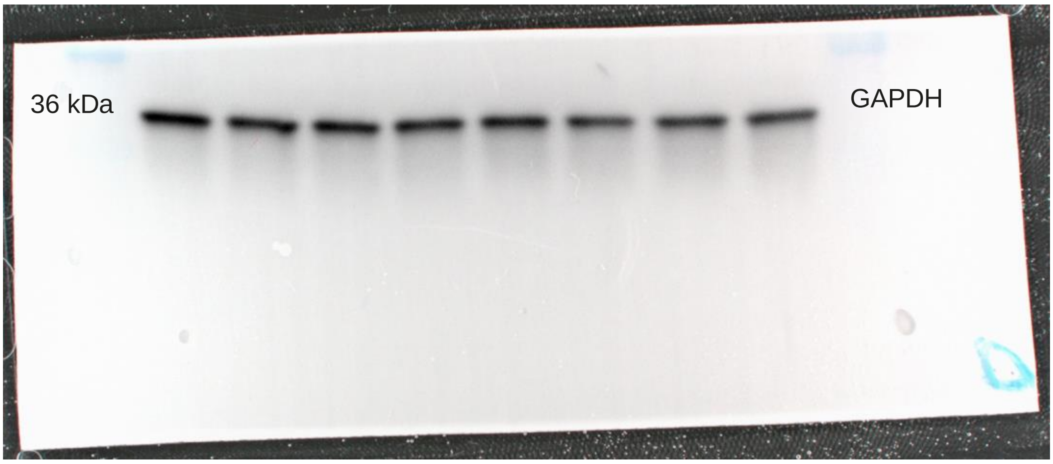

Full unedited blot for Figure 3A + Suppl. Fig. S2E #3

pJAK1

130 kDa

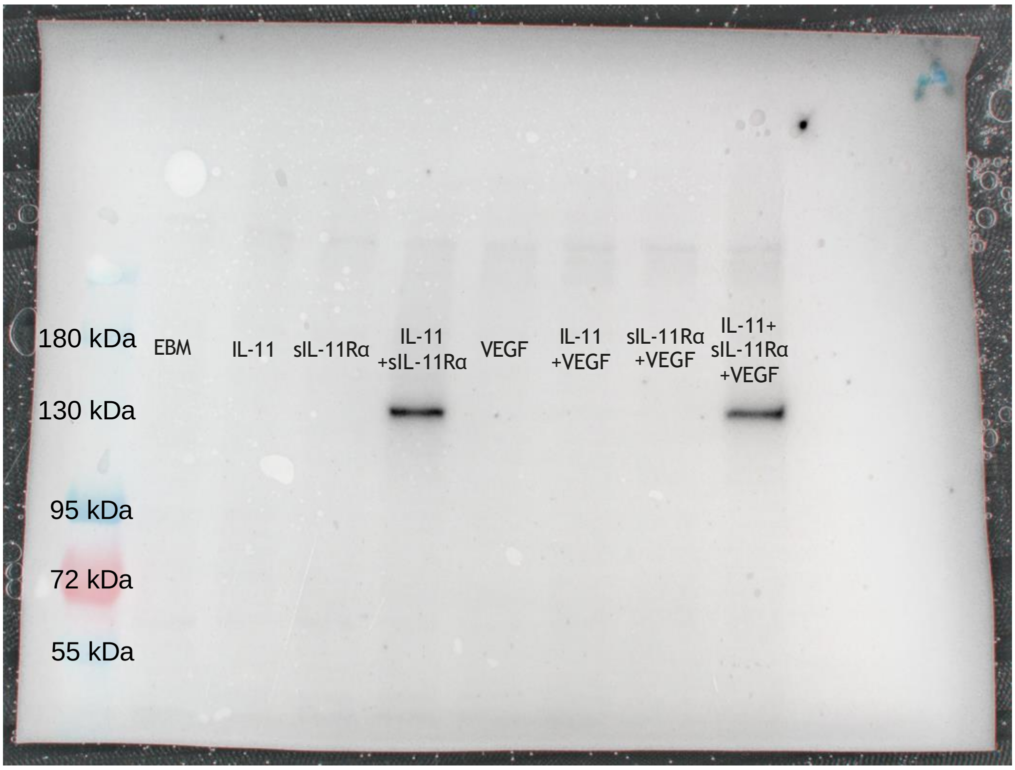

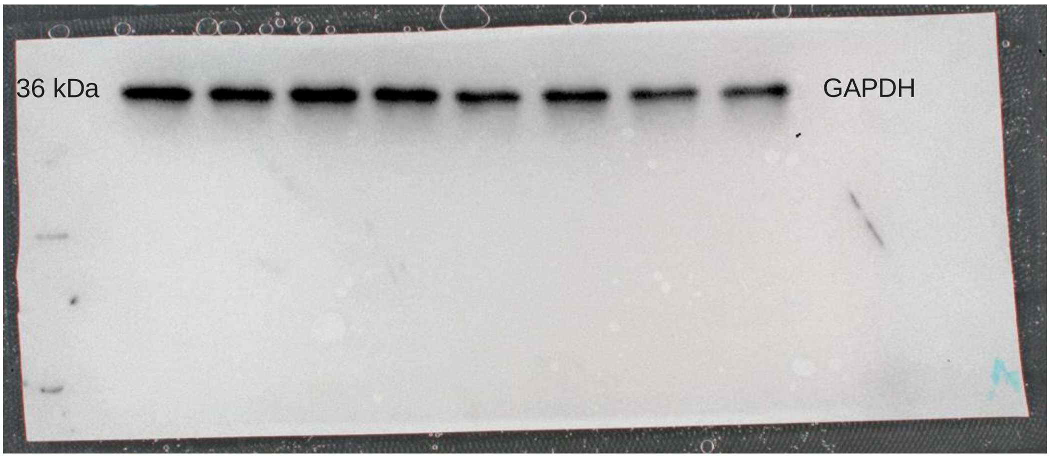

Full unedited blot for Figure 3A + Suppl. Fig. S2E #3

pJAK2

125 kDa

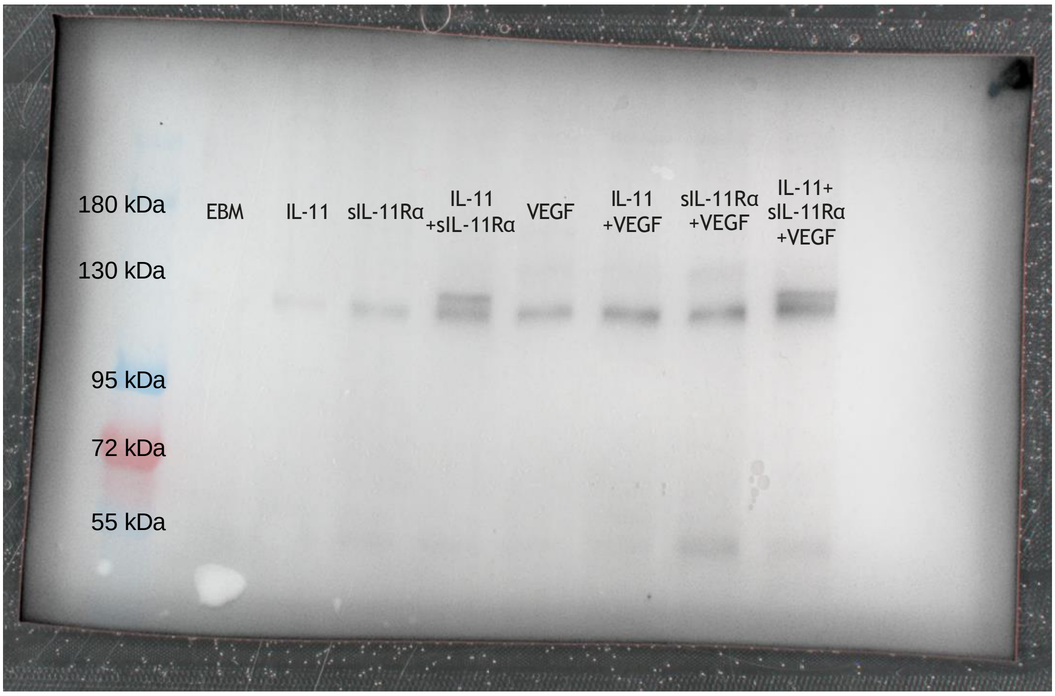

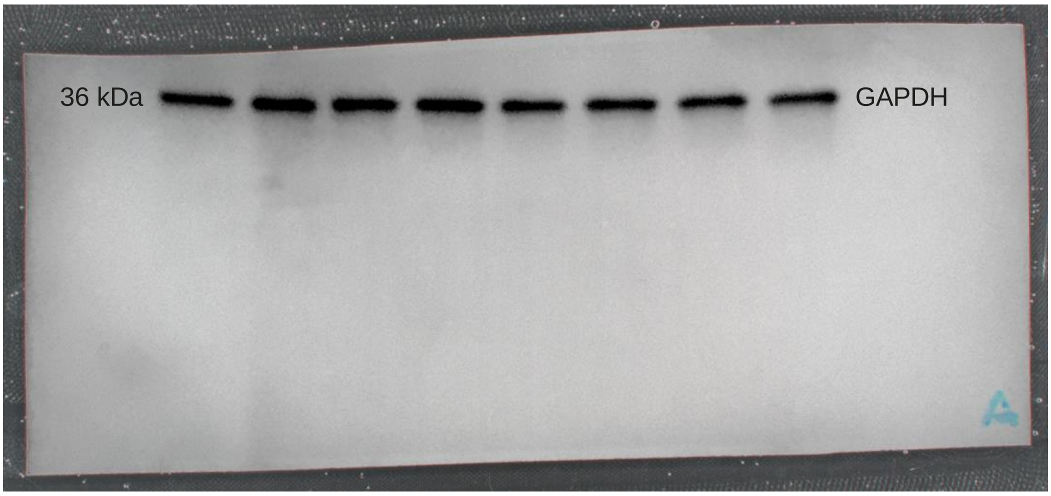

pTyk2

134 kDa

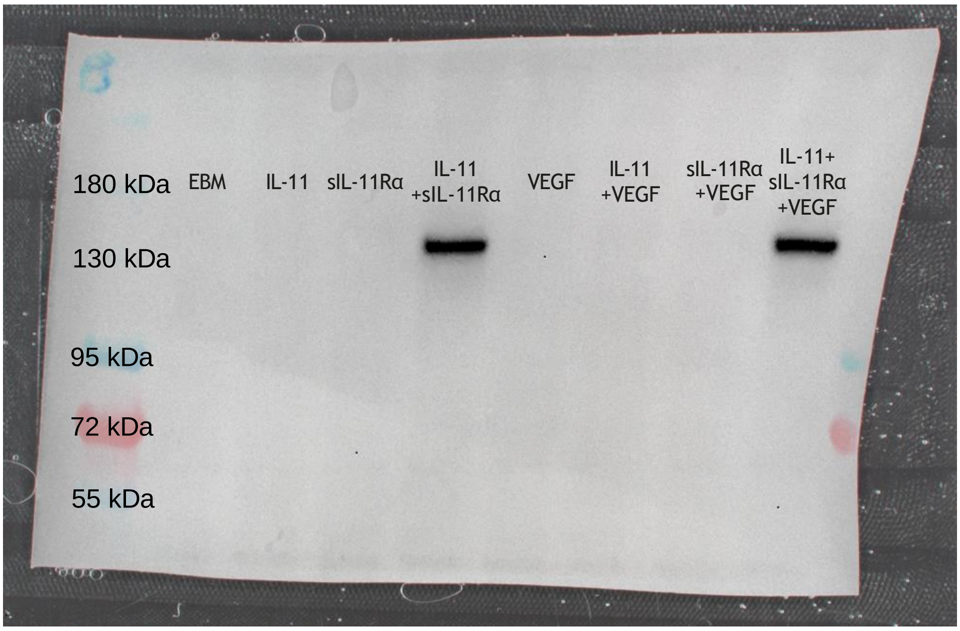

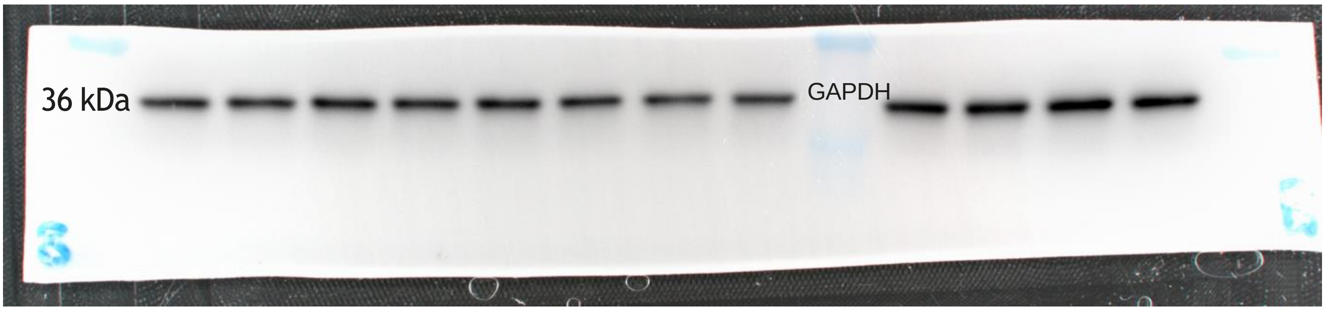

Full unedited blot for Figure 5C

#1

STAT3

79, 86 kDa

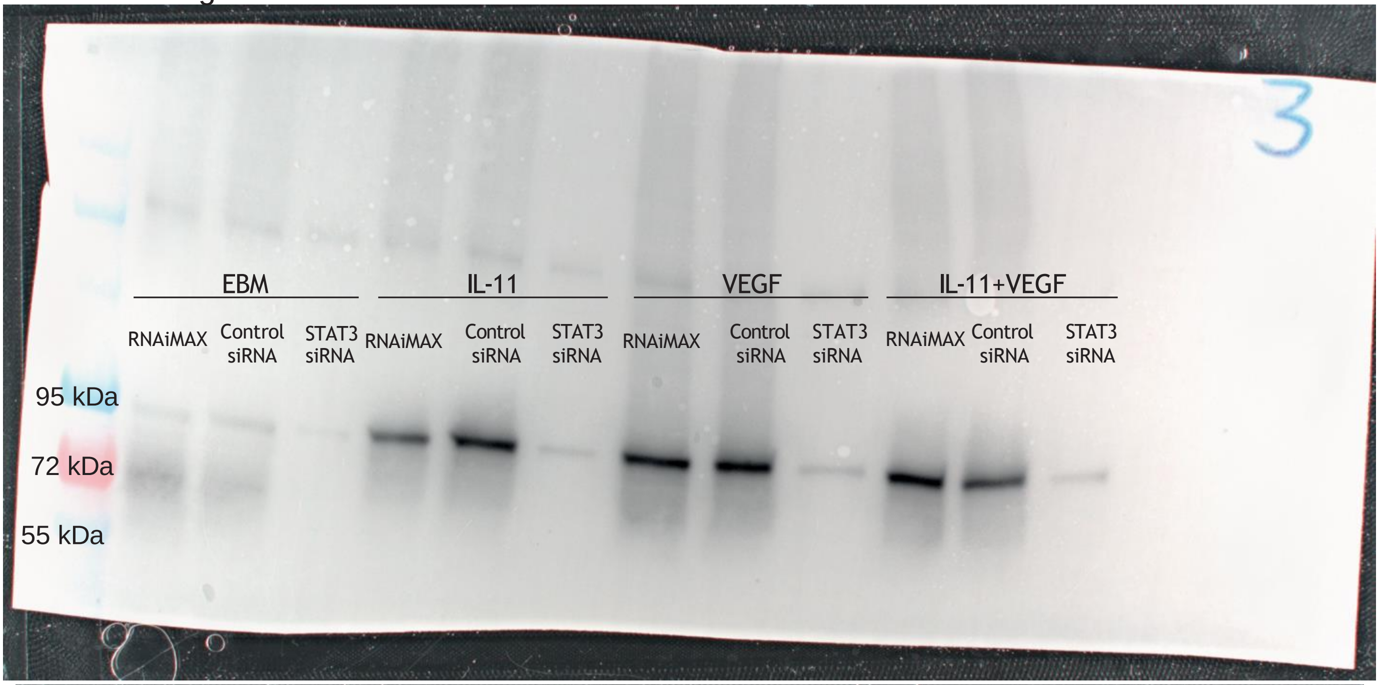

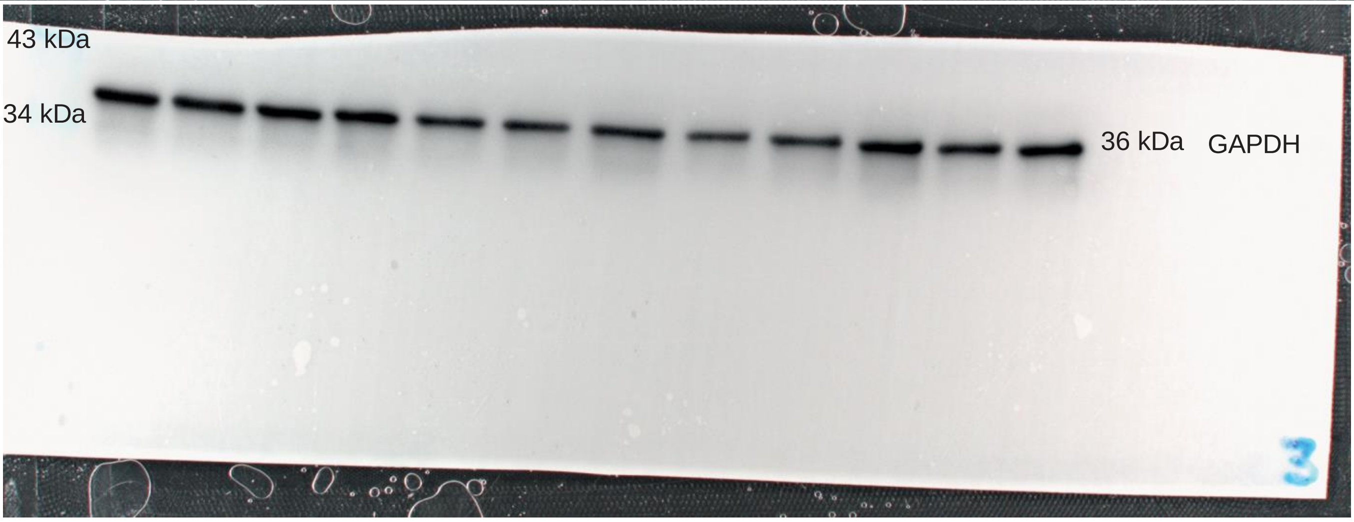

pSTAT1

84, 91 kDa

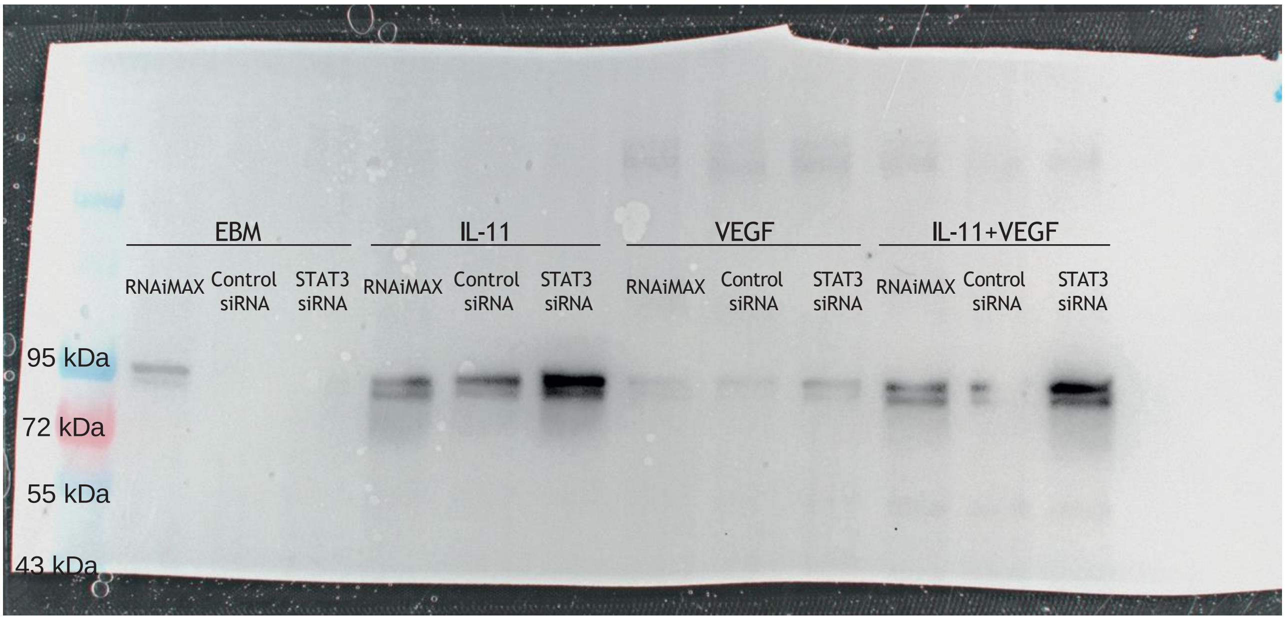

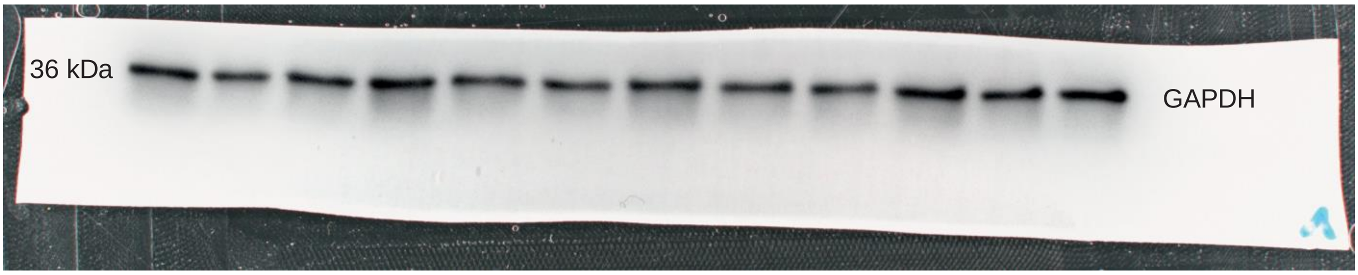

Full unedited blot for Figure 5C #1

pAkt

60 kDa

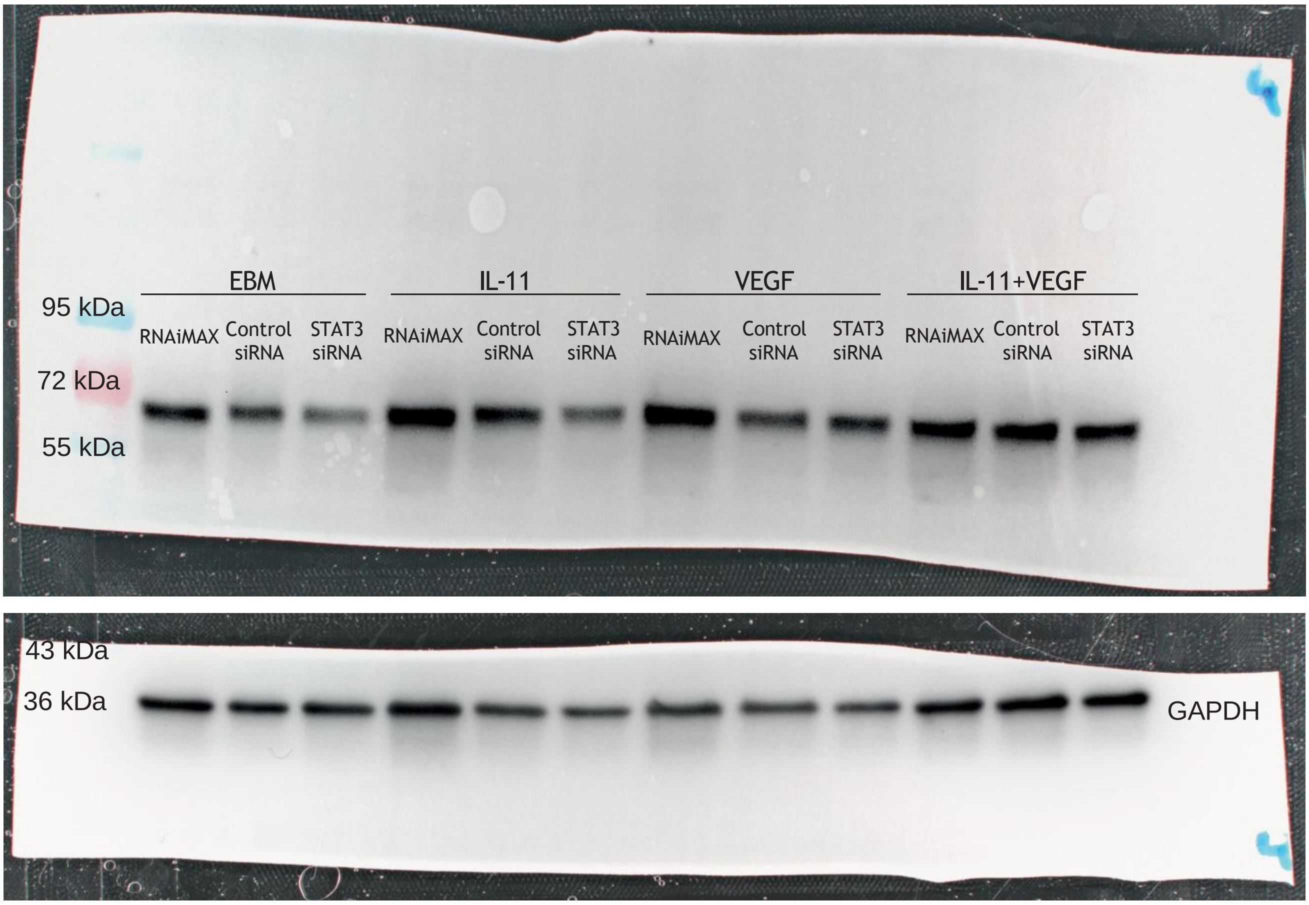

Full unedited blot for Figure 5C #1

pSTAT5

90 kDa

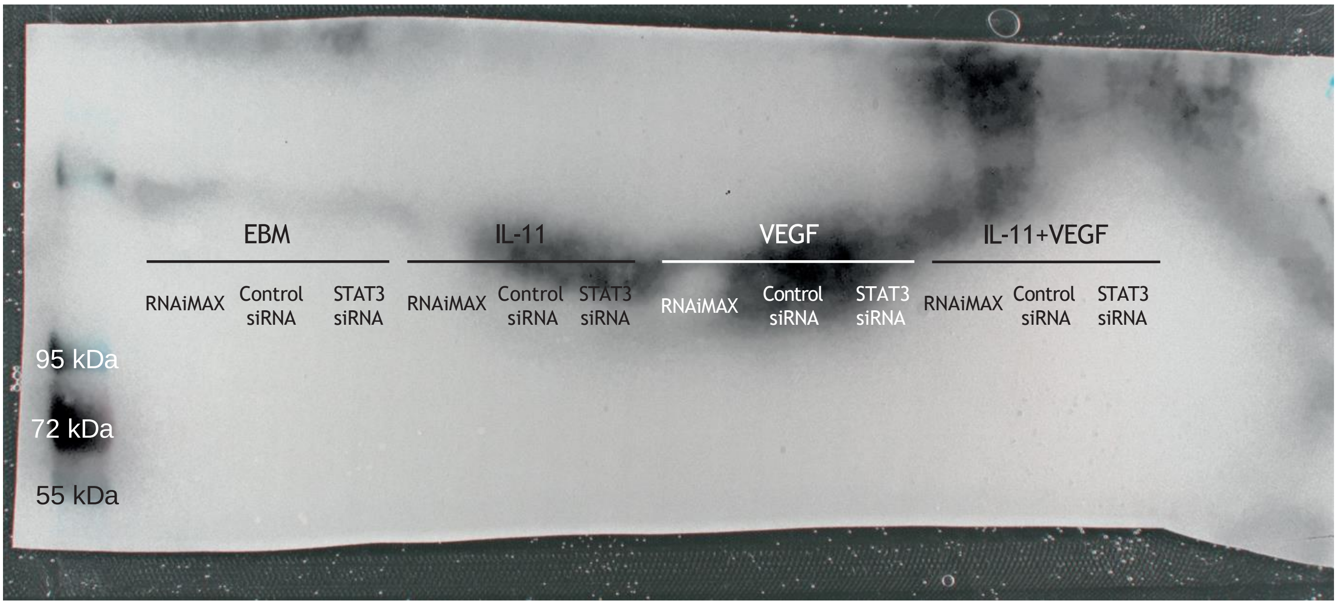

pERK

44, 42 kDa

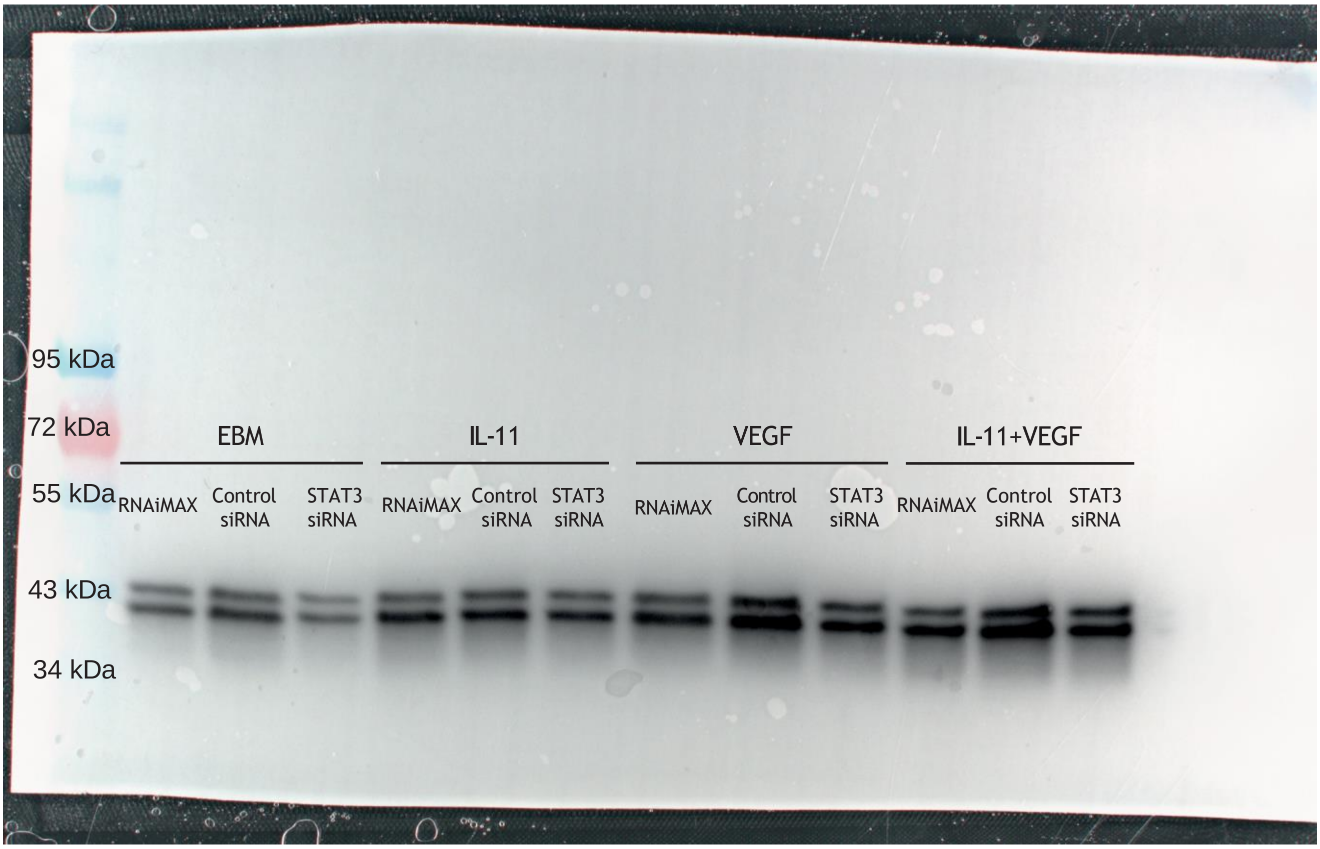

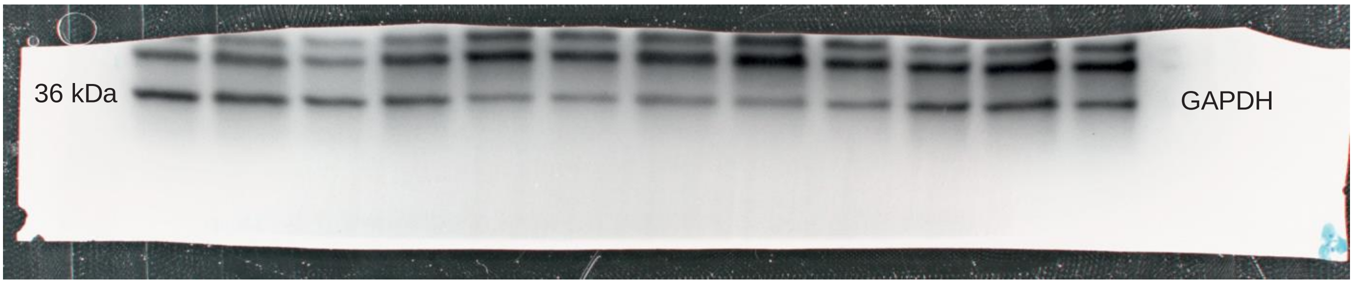

Full unedited blot for Figure 5C #2

STAT3

79, 86 kDa

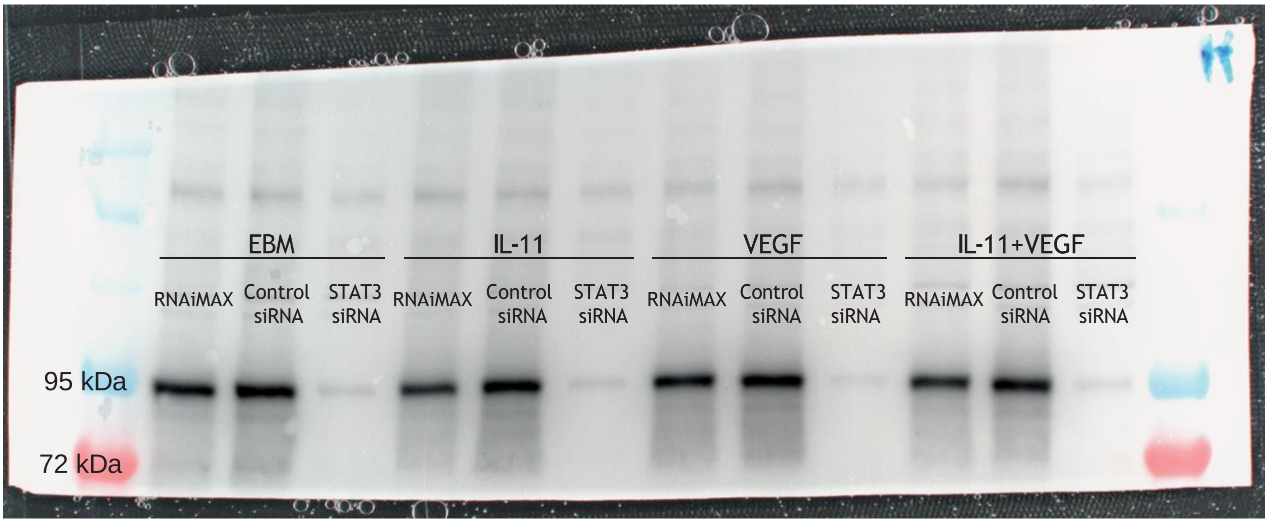

pERK

44, 42 kDa

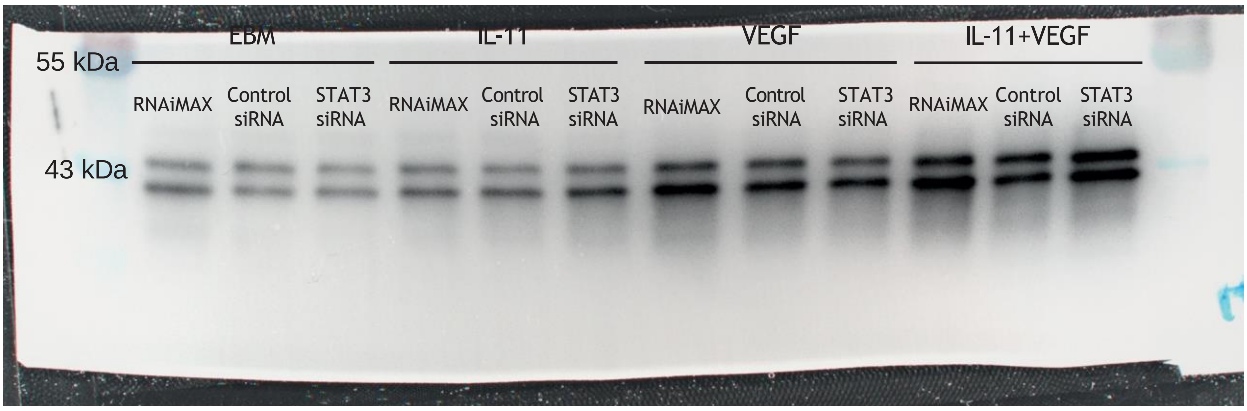

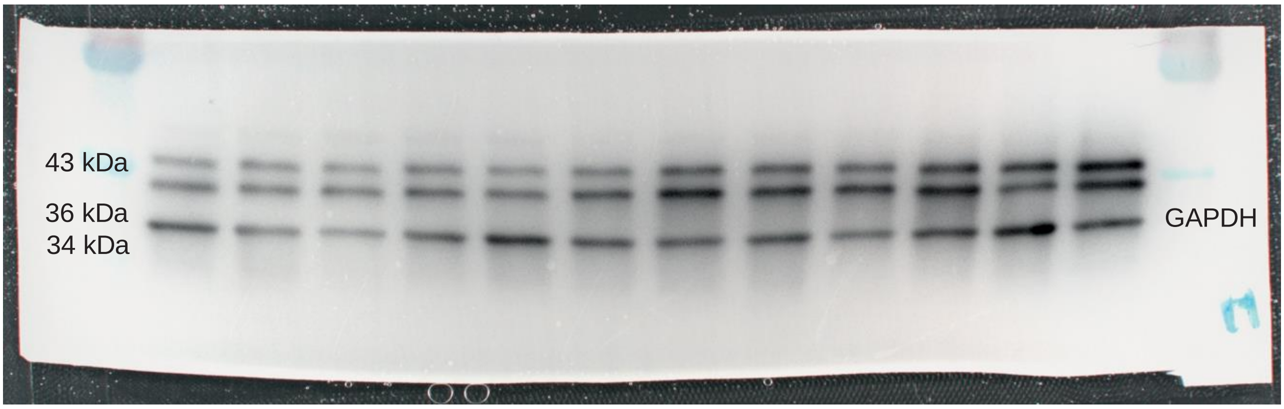

Full unedited blot for Figure 5C + Suppl. Fig. S3A #2

pSTAT1

84, 91 kDa

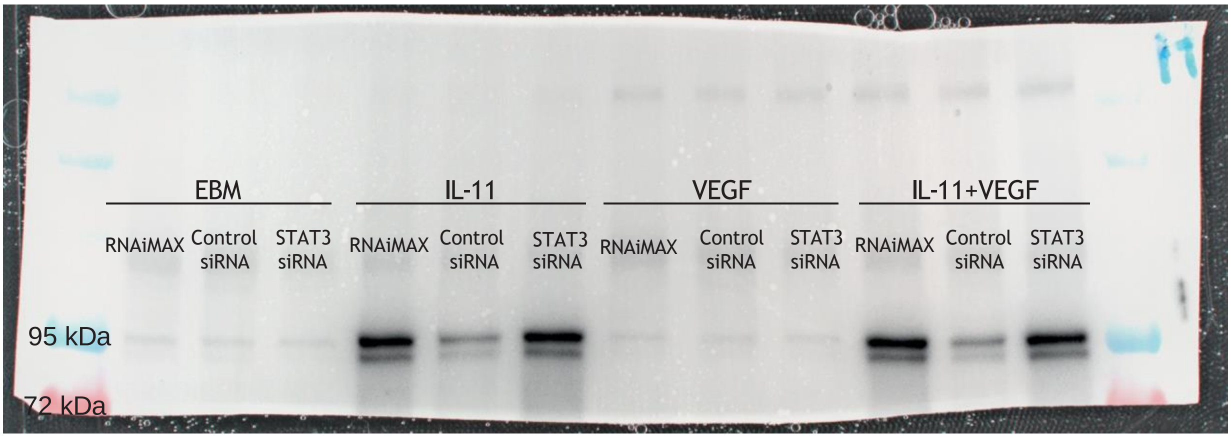

EBM | IL-11 | VEGF | IL-11+VEGF

RNAiMAX Control siRNA STAT3 siRNA RNAiMAX Control siRNA STAT3 siRNA RNAiMAX Control siRNA STAT3 siRNA RNAiMAX Control siRNA STAT3 siRNA

pAkt

60 kDa

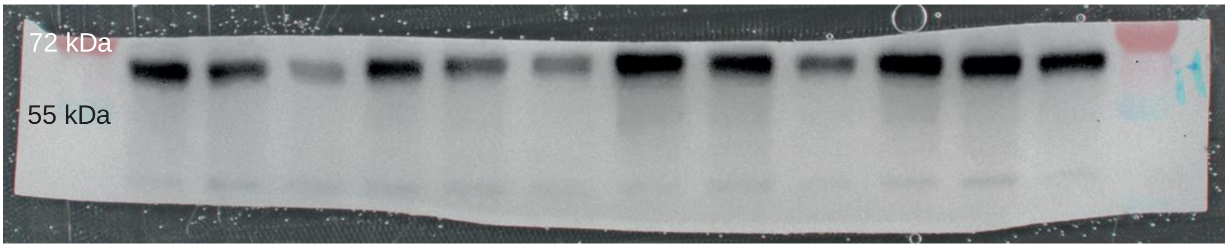

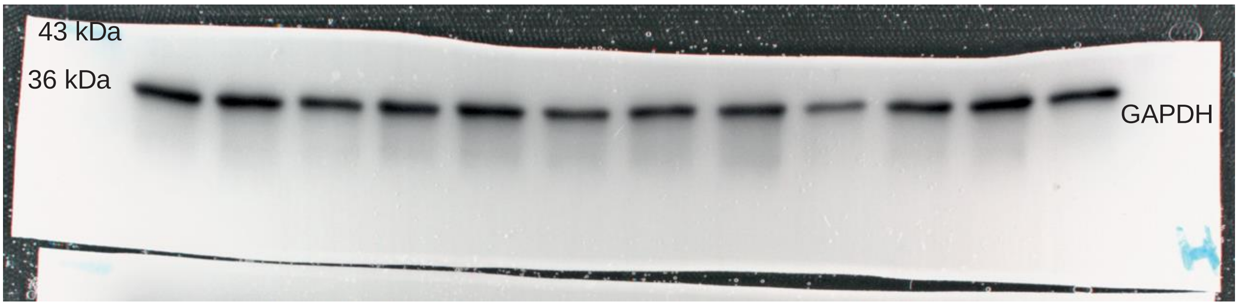

Full unedited blot for Figure 5C + Suppl. Fig. S3A #2

pSTAT5

90 kDa

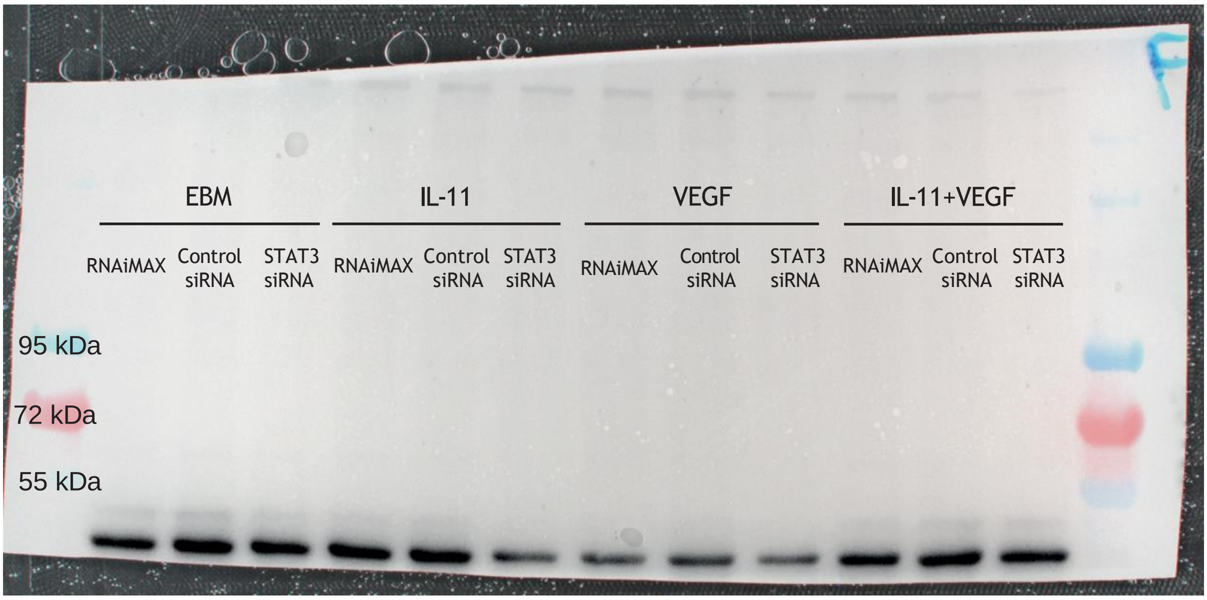

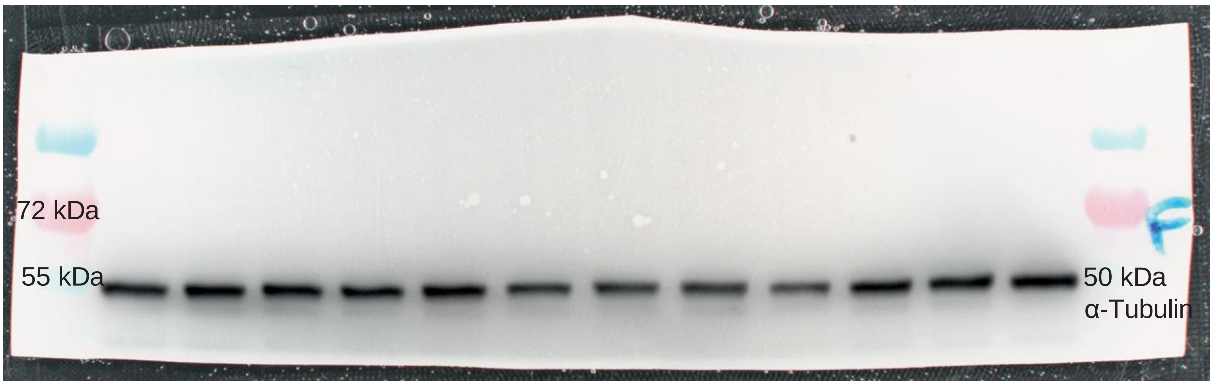

Full unedited blot for Figure 5C #3

STAT3

79, 86 kDa

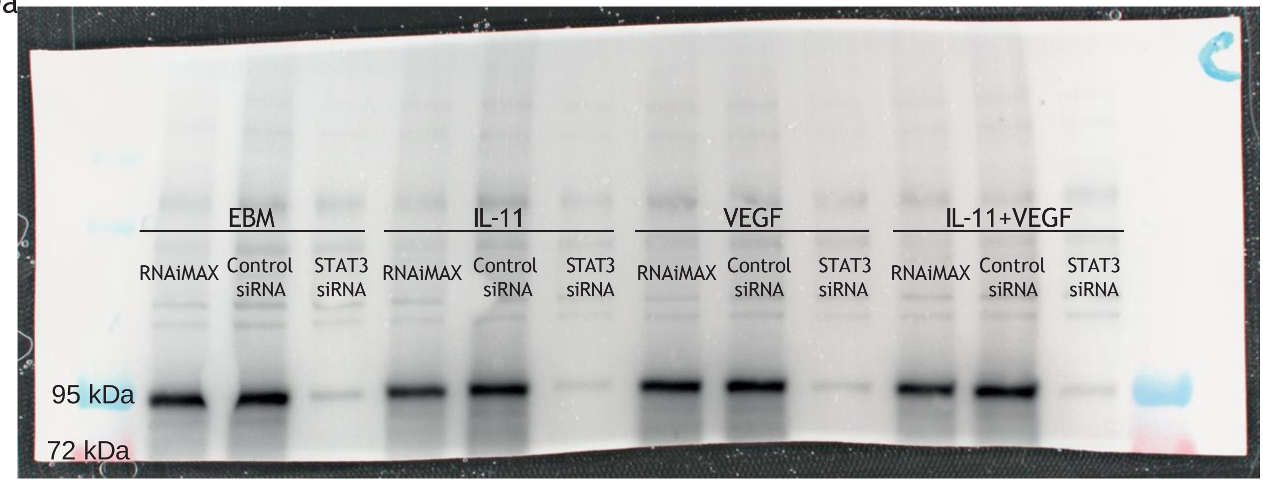

pAkt

60 kDa

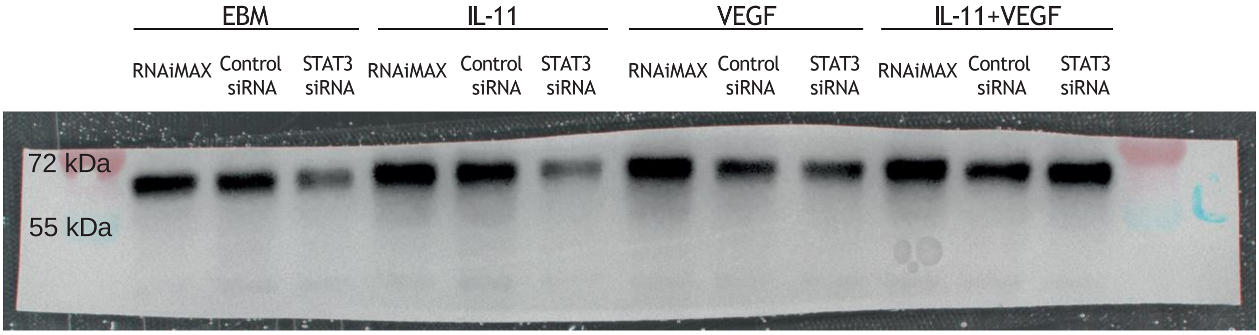

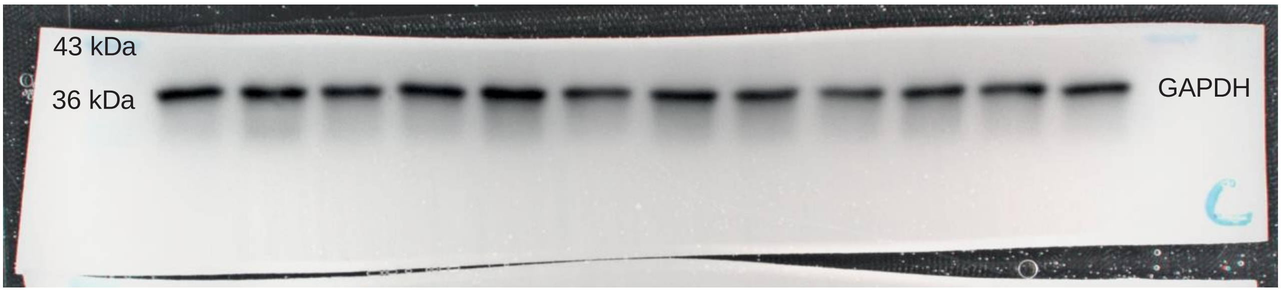

Full unedited blot for Figure 5C #3

pSTAT1

84, 91 kDa

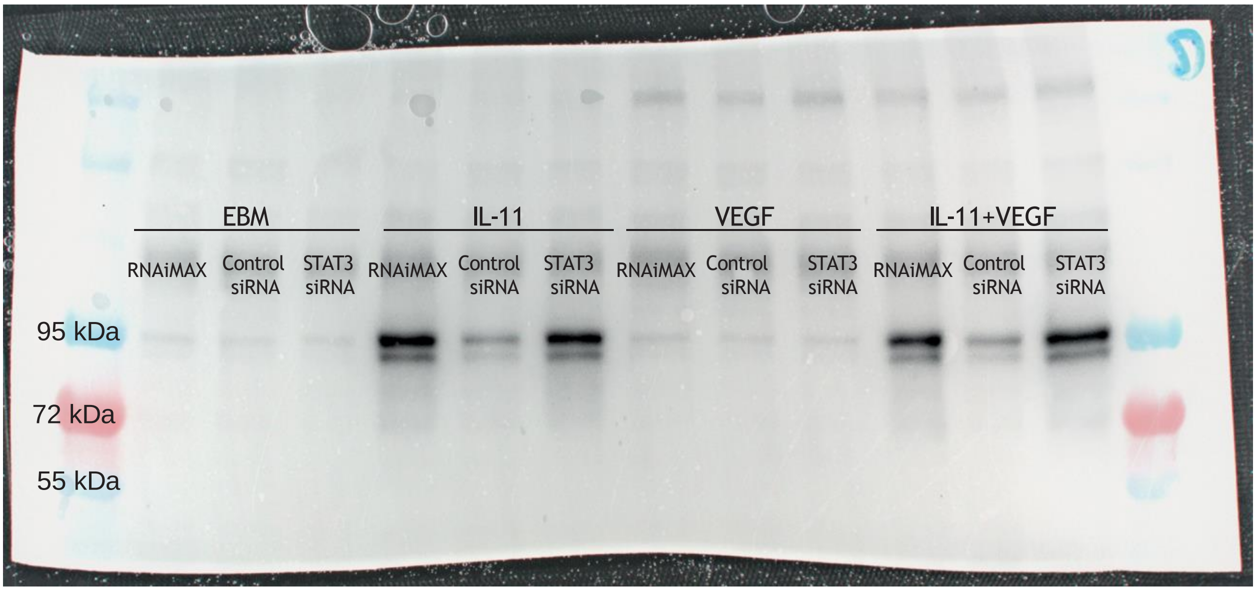

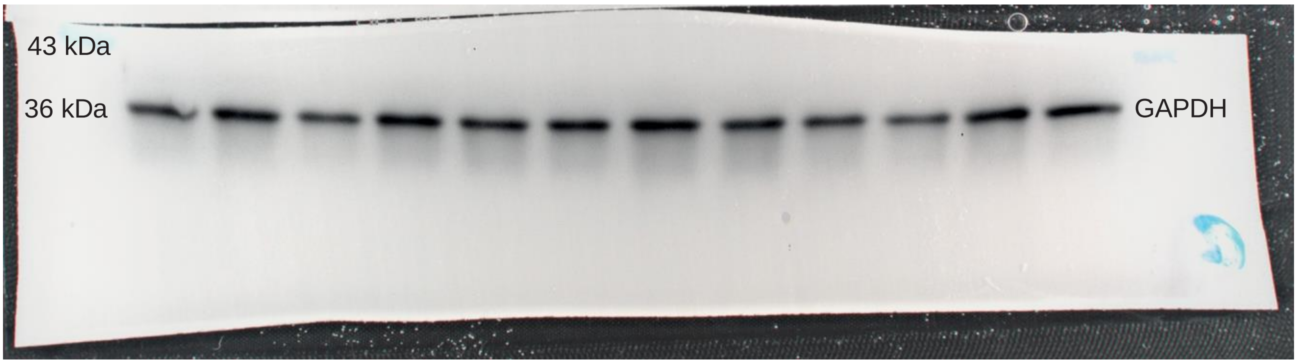

Full unedited blot for Figure 5C #3

pSTAT5

90 kDa

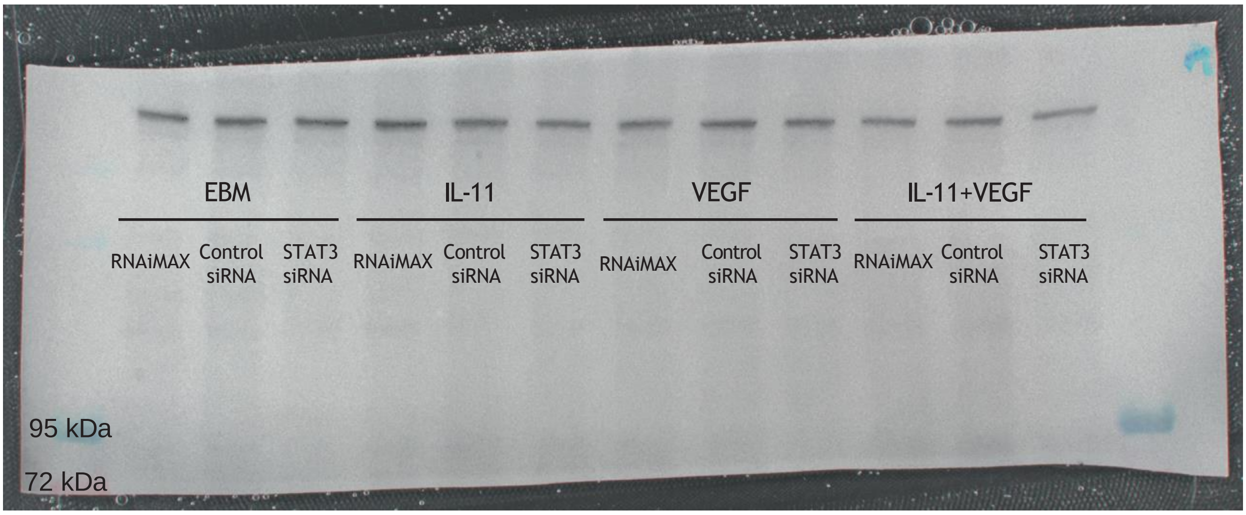

pERK

44, 42 kDa

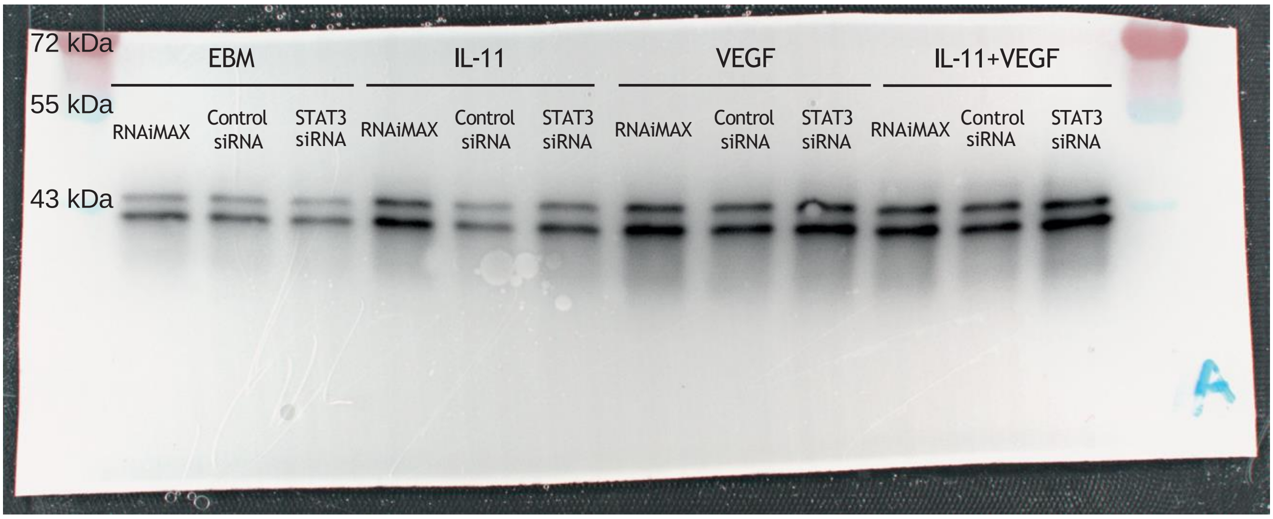

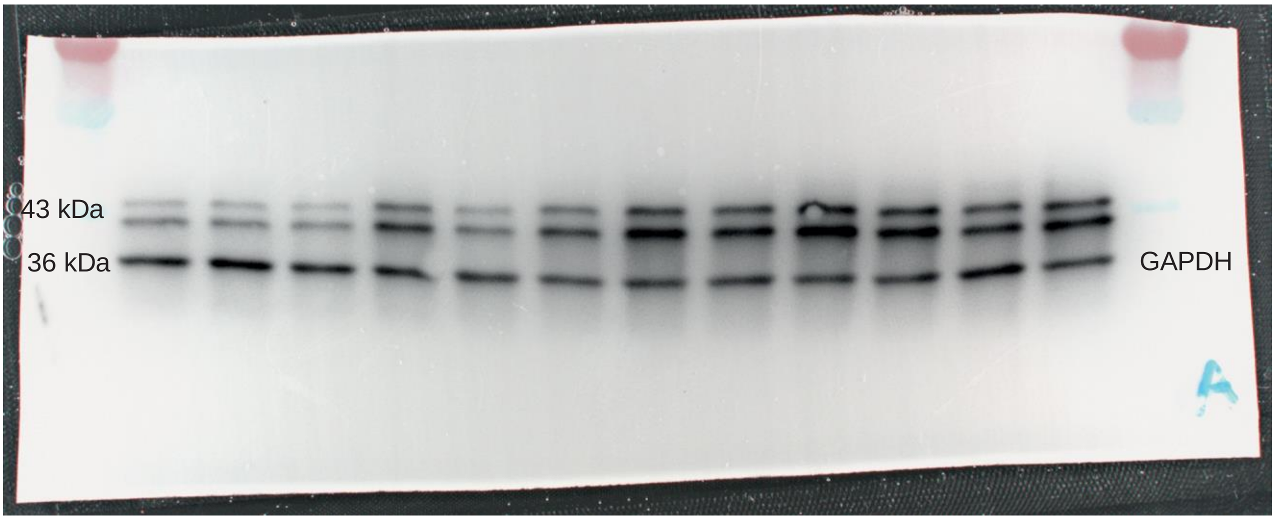

Full unedited blot for Figure 5D

#1

STAT3

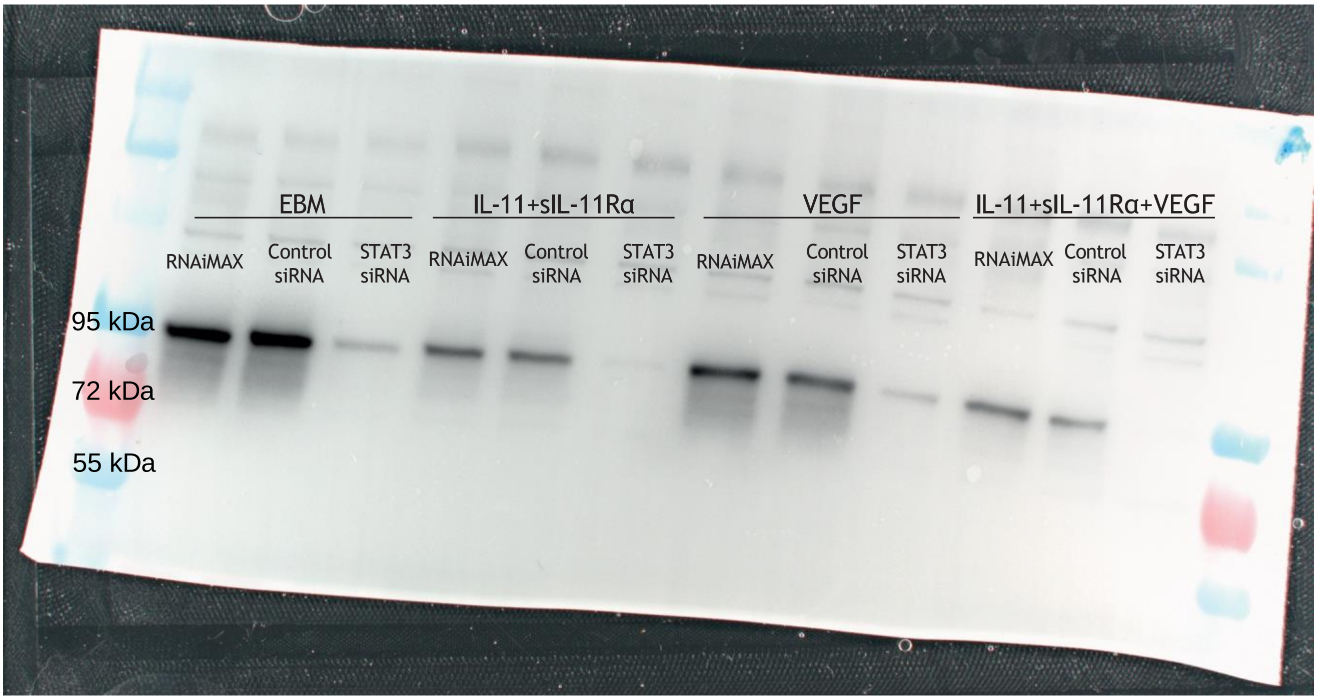

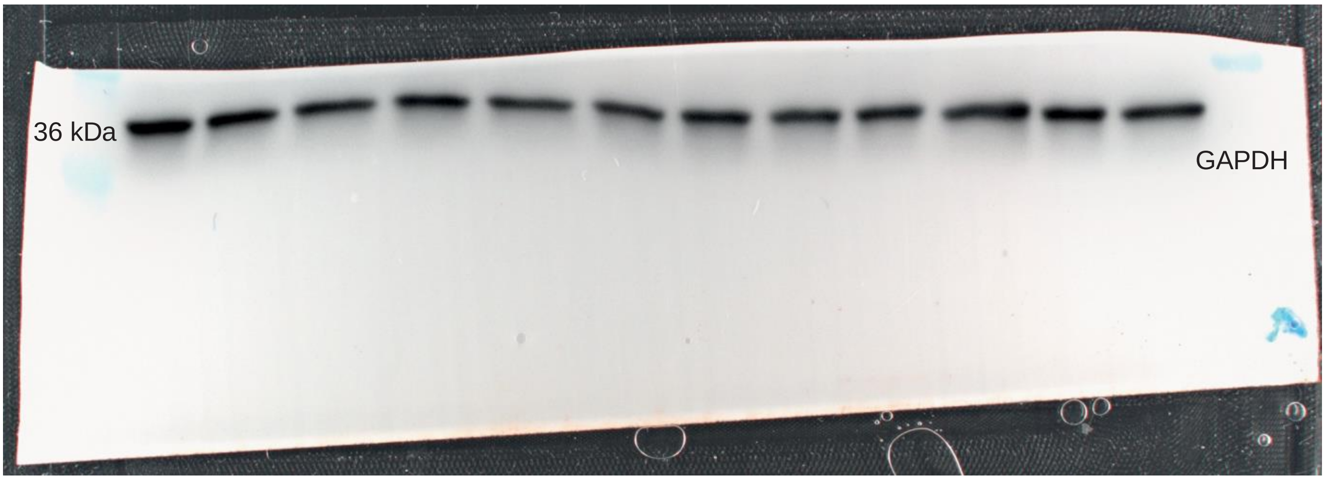

Full unedited blot for Figure 5D #1

pERK

44, 42 kDa

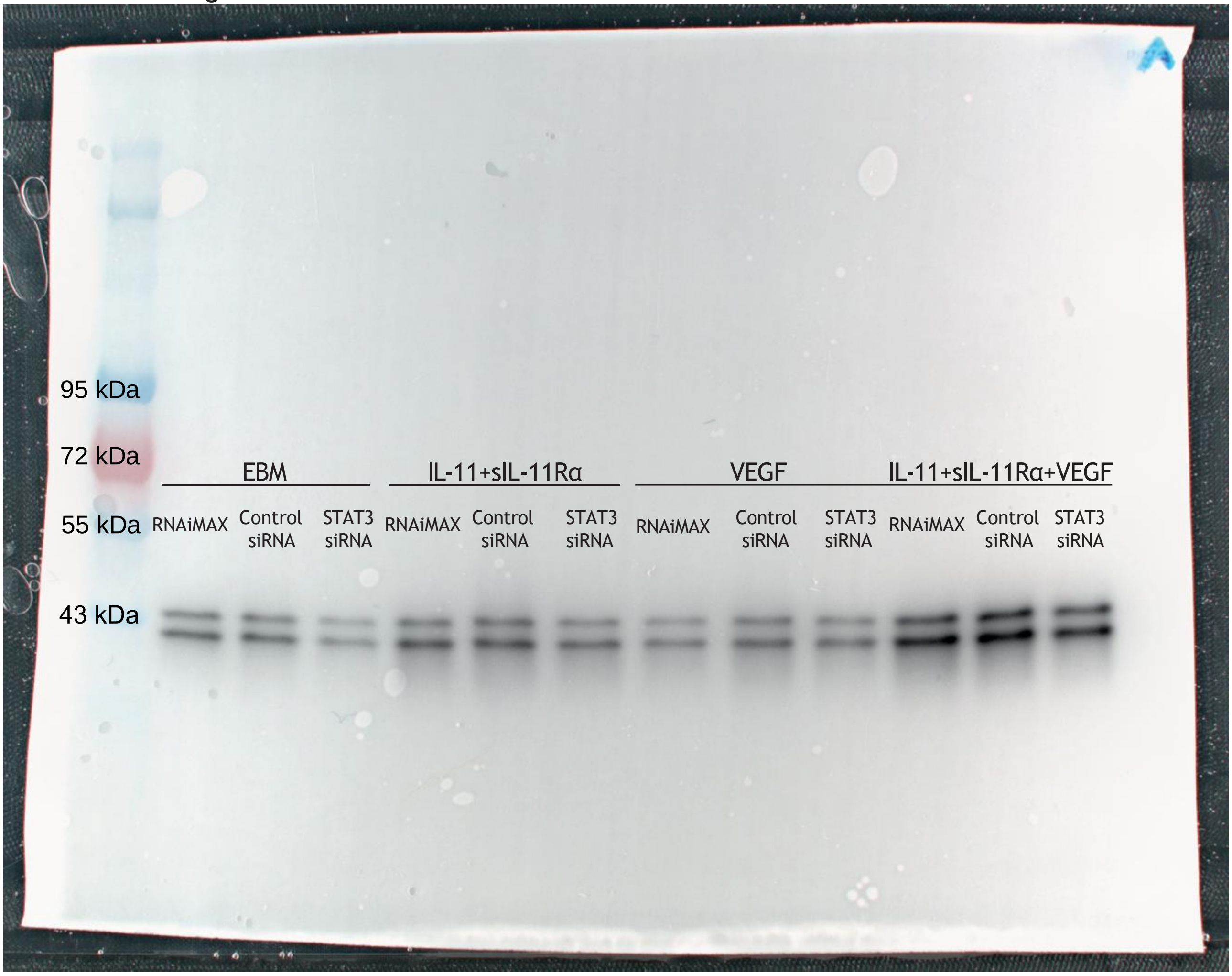

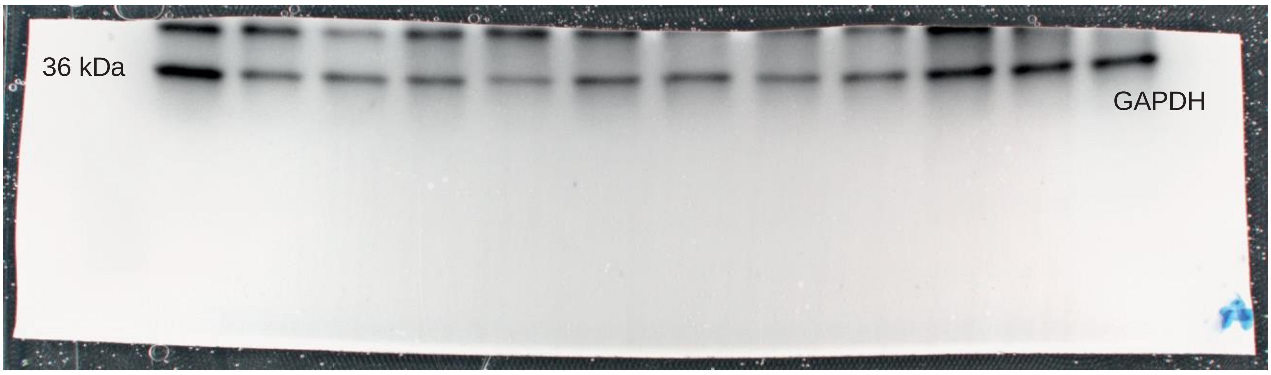

Full unedited blot for Figure 5D #1

pSTAT1

84, 91 kDa

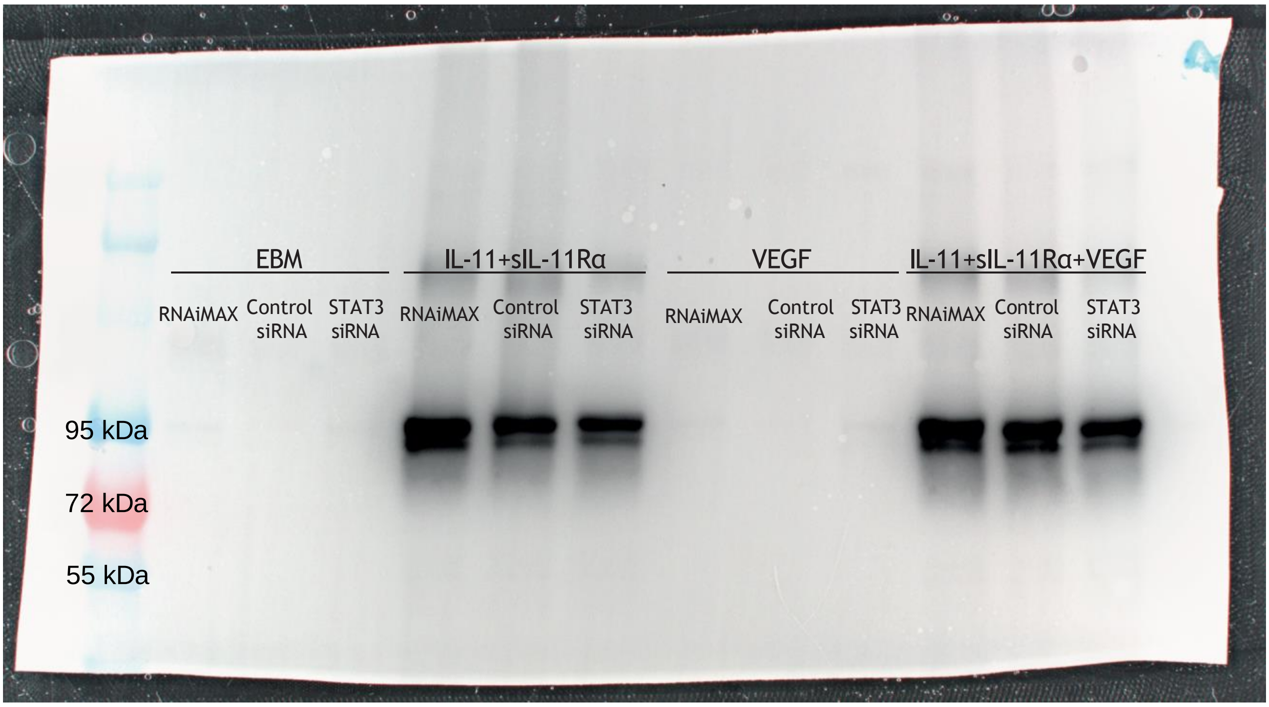

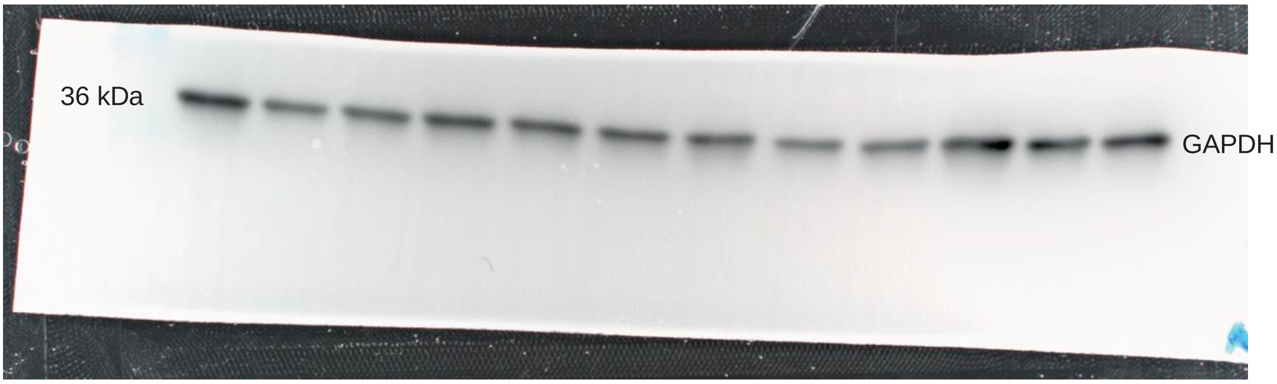

Full unedited blot for Figure 5D #1

pSTAT5

90 kDa

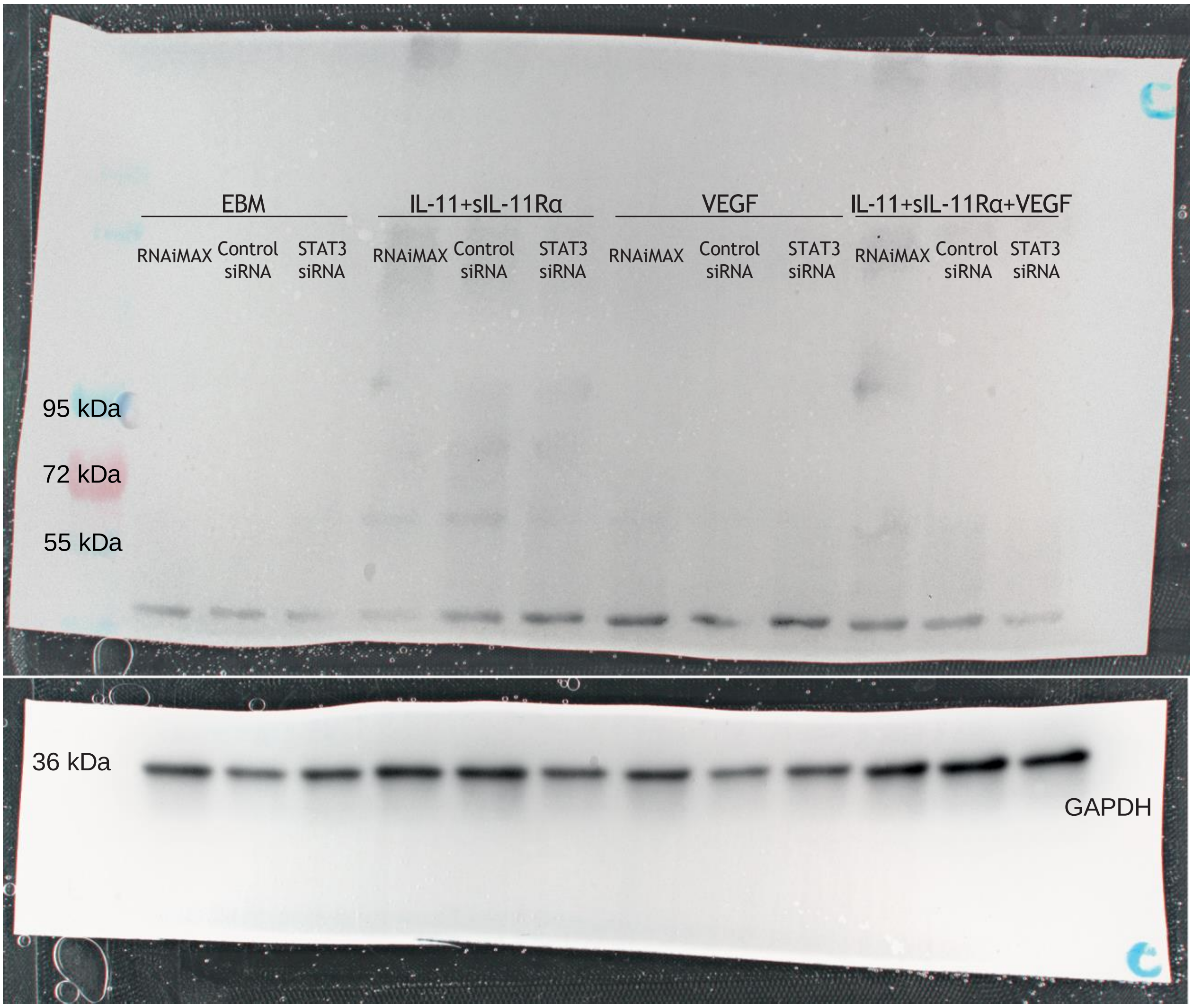

pAkt

60 kDa

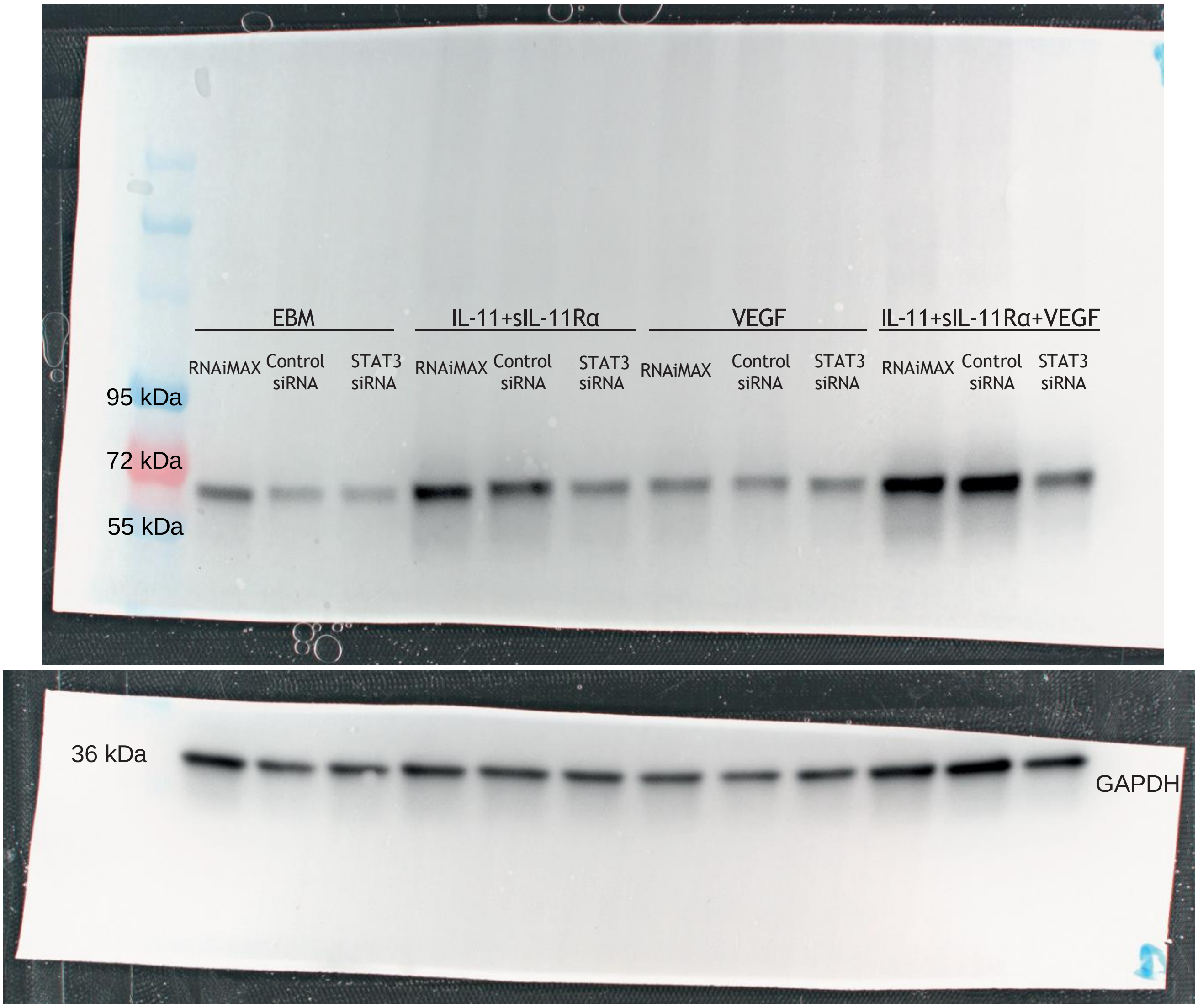

Full unedited blot for Figure 5D #2

STAT3

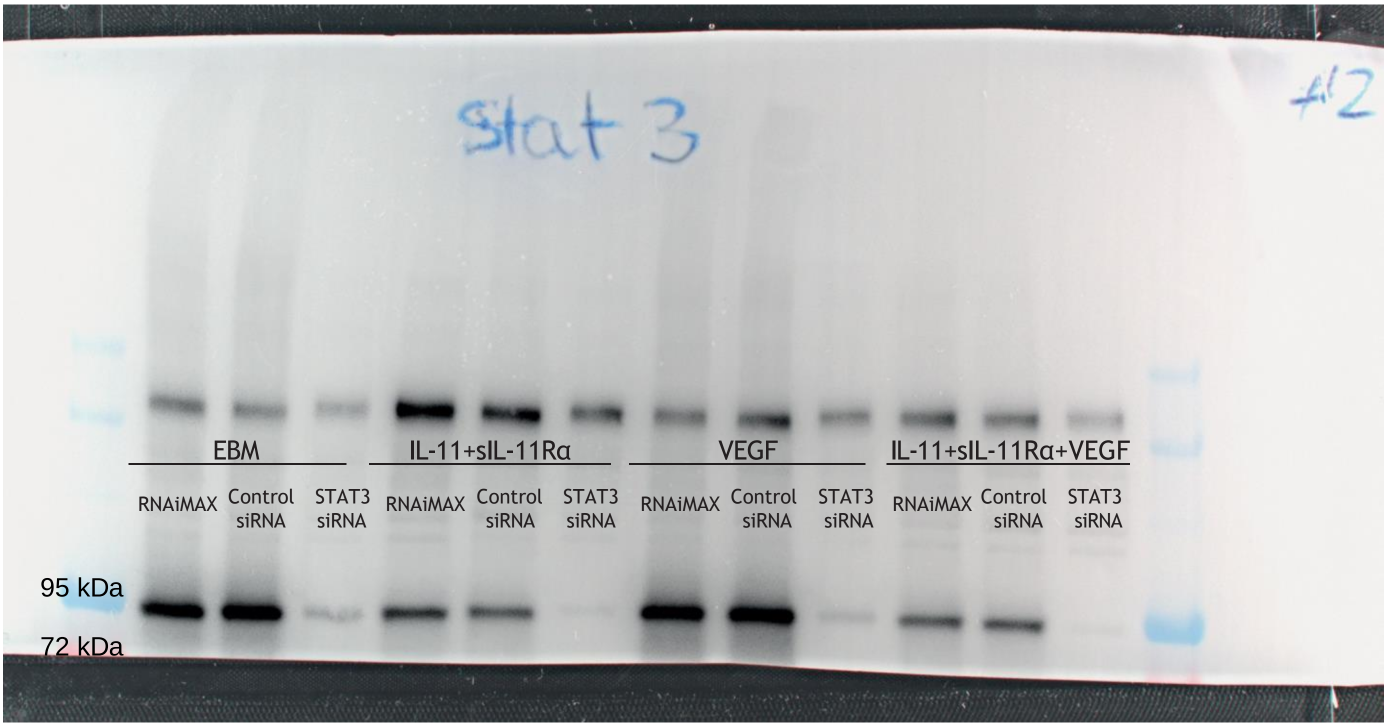

pAkt

60 kDa

EBM			IL-11+sIL-11Rα			VEGF			IL-11+sIL-11Rα+VEGF		
RNAiMAX	Control siRNA	STAT3 siRNA	RNAiMAX	Control siRNA	STAT3 siRNA	RNAiMAX	Control siRNA	STAT3 siRNA	RNAiMAX	Control siRNA	STAT3 siRNA

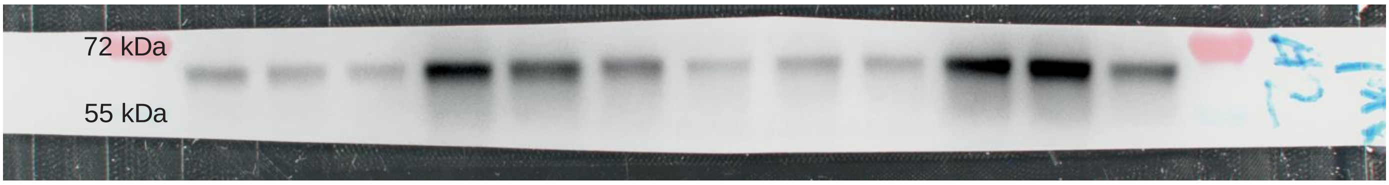

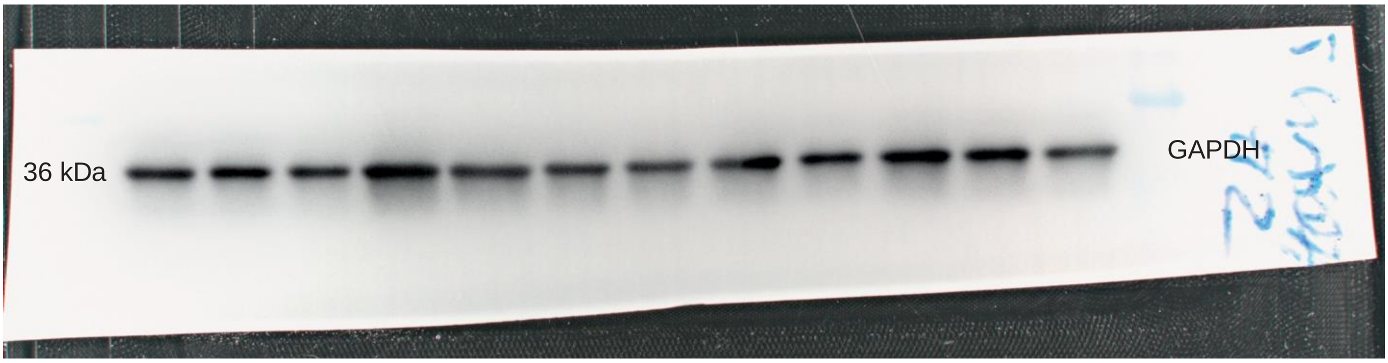

Full unedited blot for Figure 5D #2

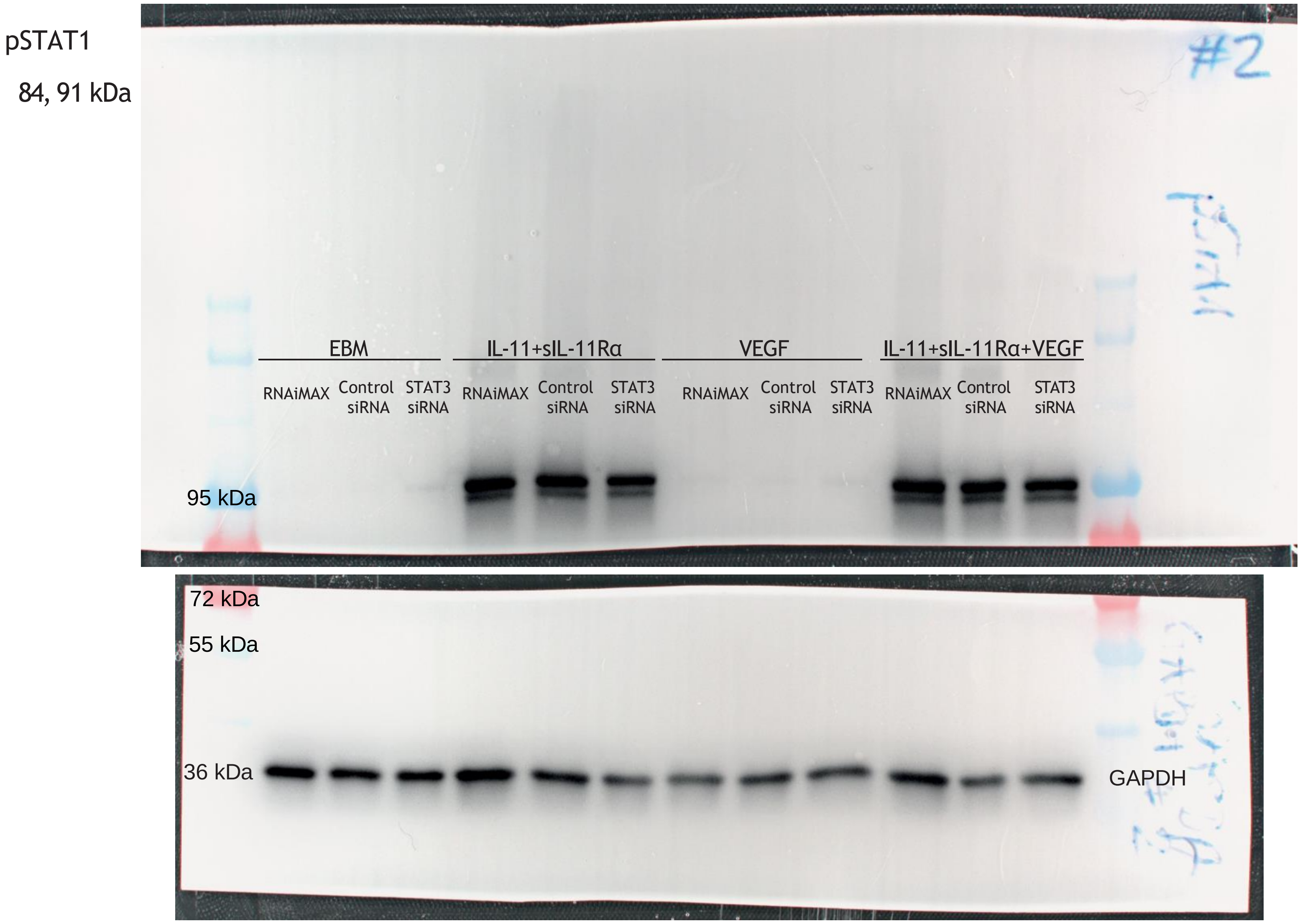

Full unedited blot for Figure 5D #2

pSTAT5

90 kDa

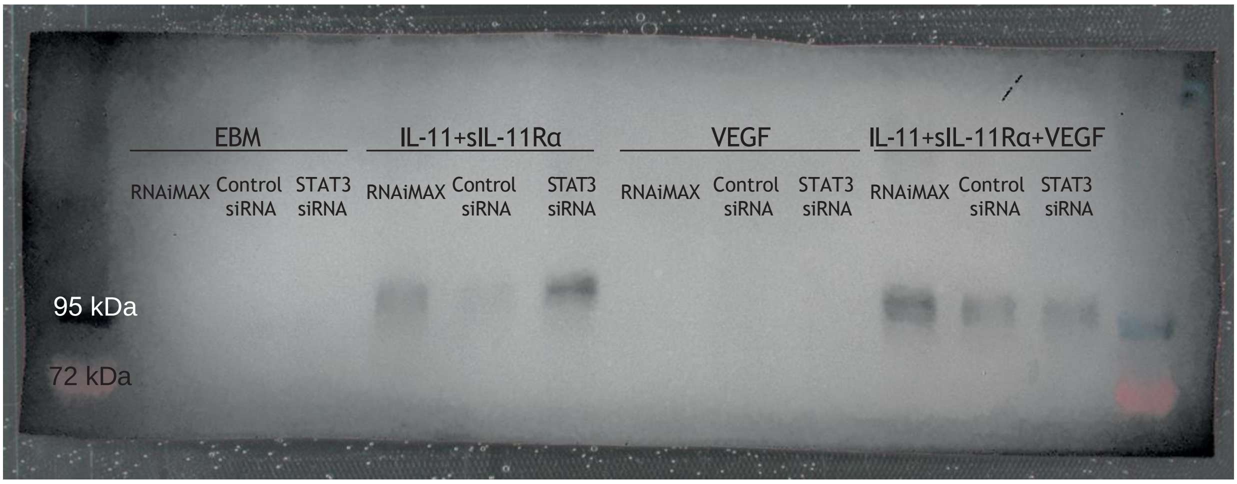

pERK

44, 42 kDa

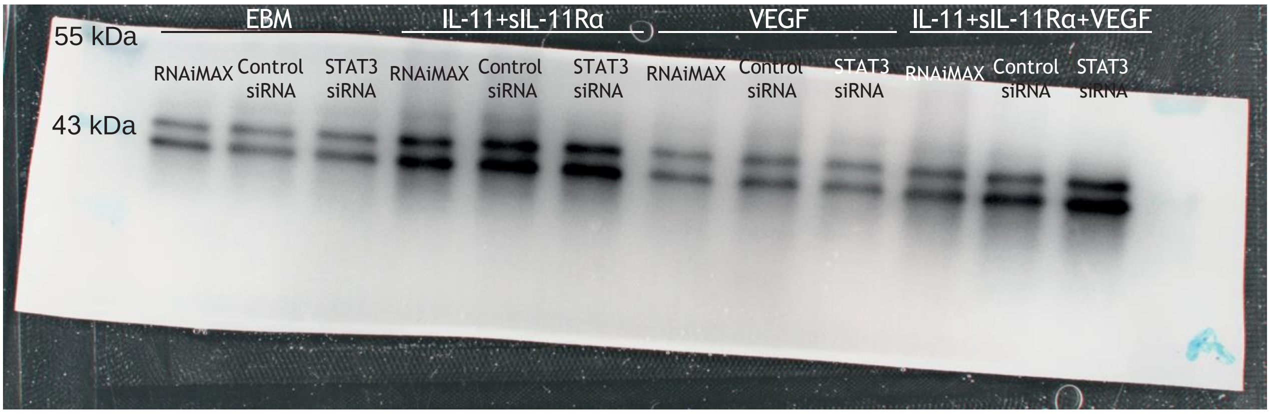

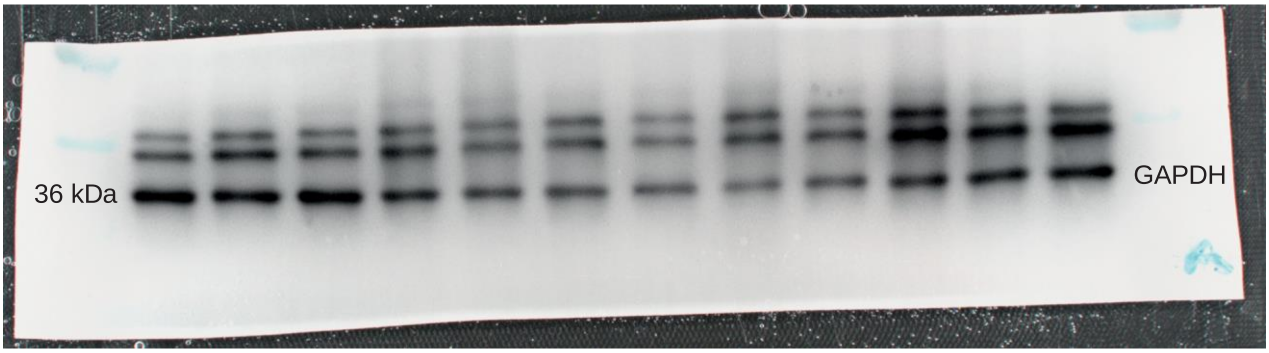

Full unedited blot for Figure 5D #3

STAT3

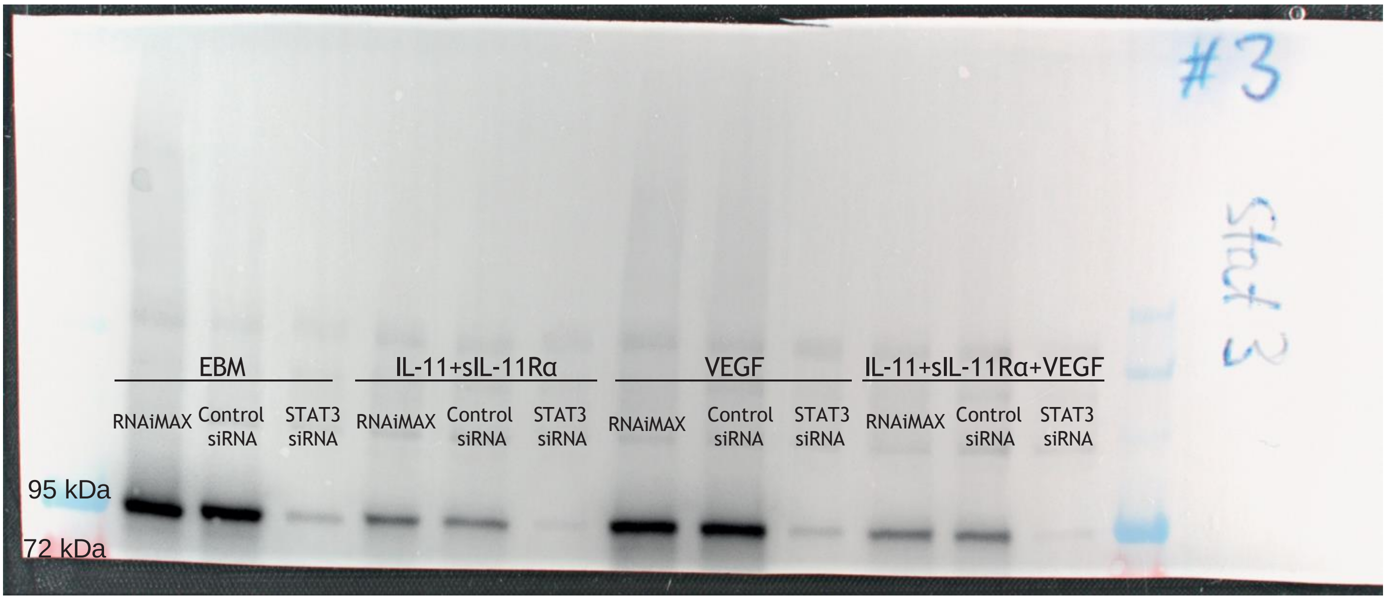

pAkt

60 kDa

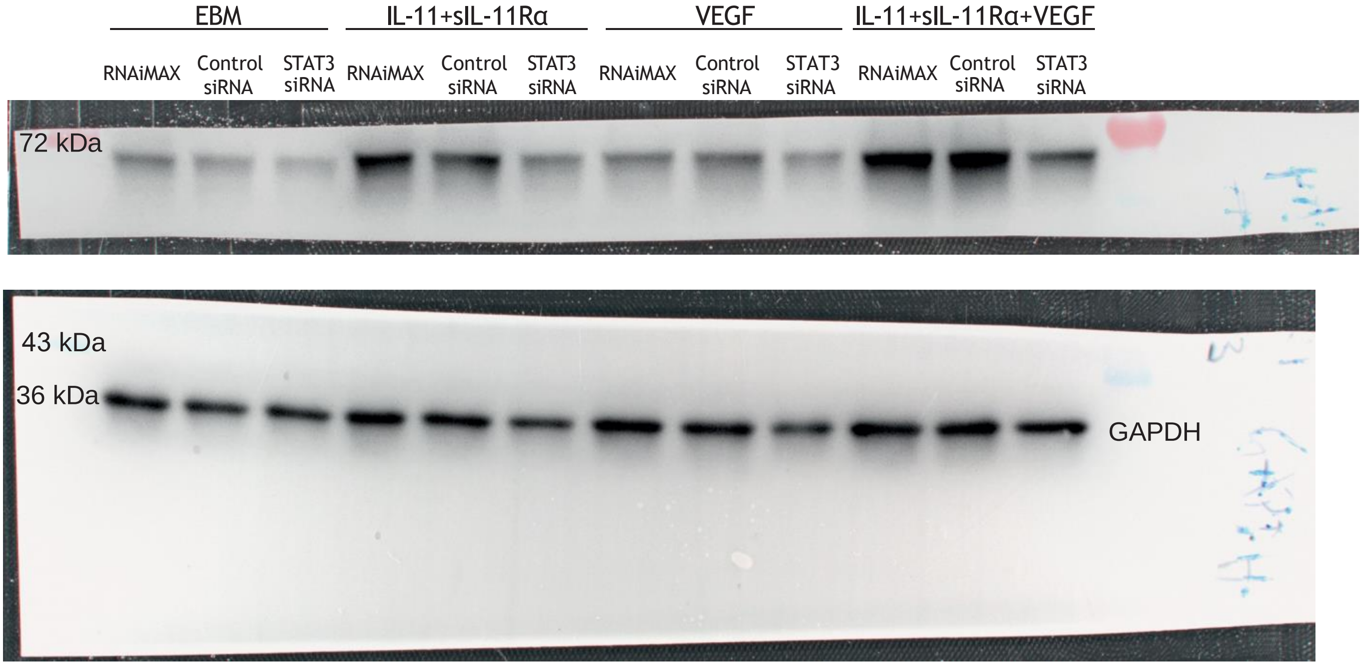

Full unedited blot for Figure 5D #3

pSTAT1
84, 91 kDa

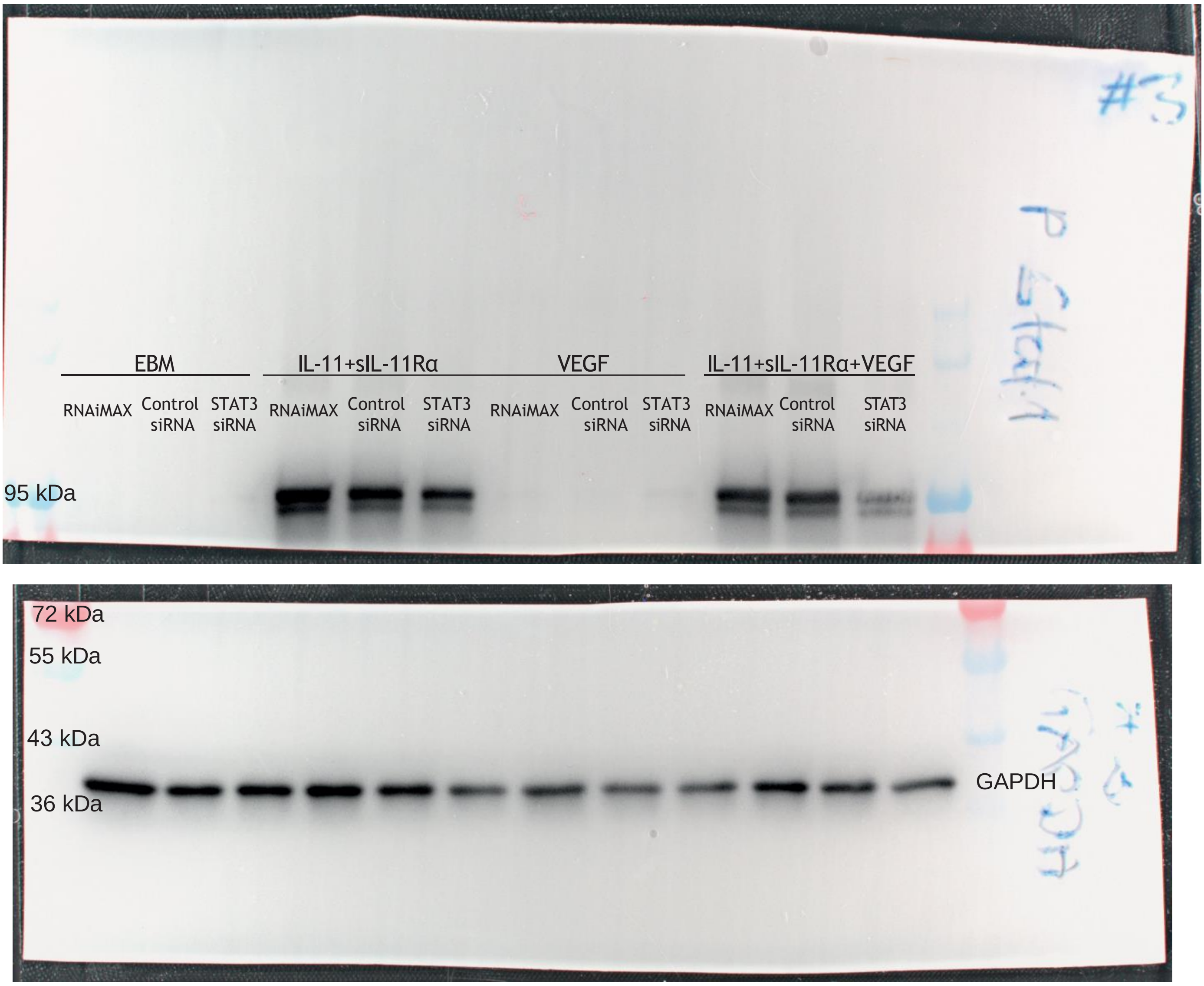

Full unedited blot for Figure 5D #3

pSTAT5

90 kDa

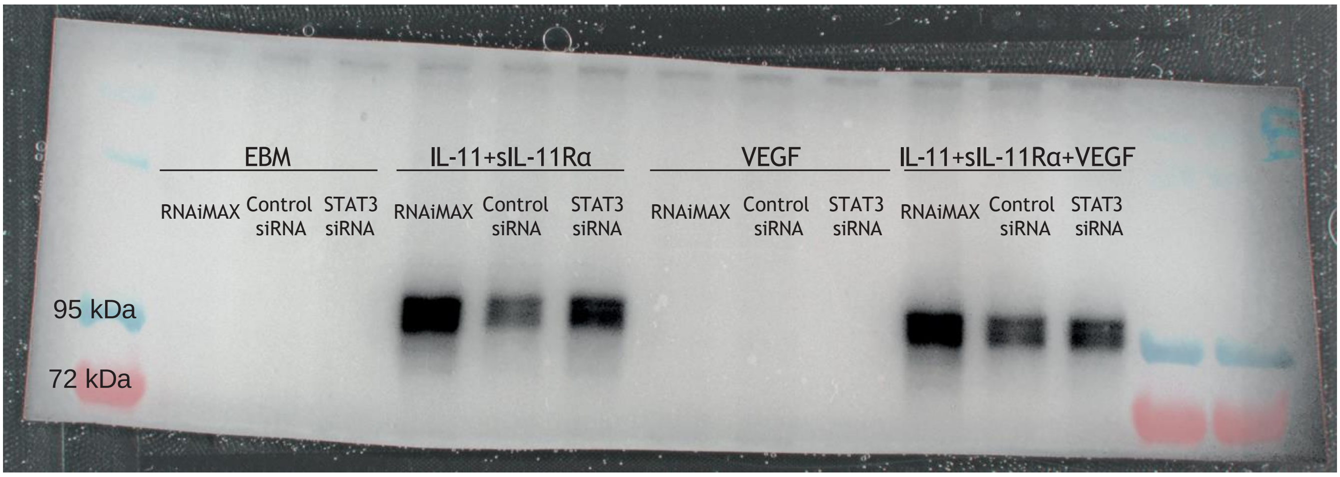

pERK

44, 42 kDa

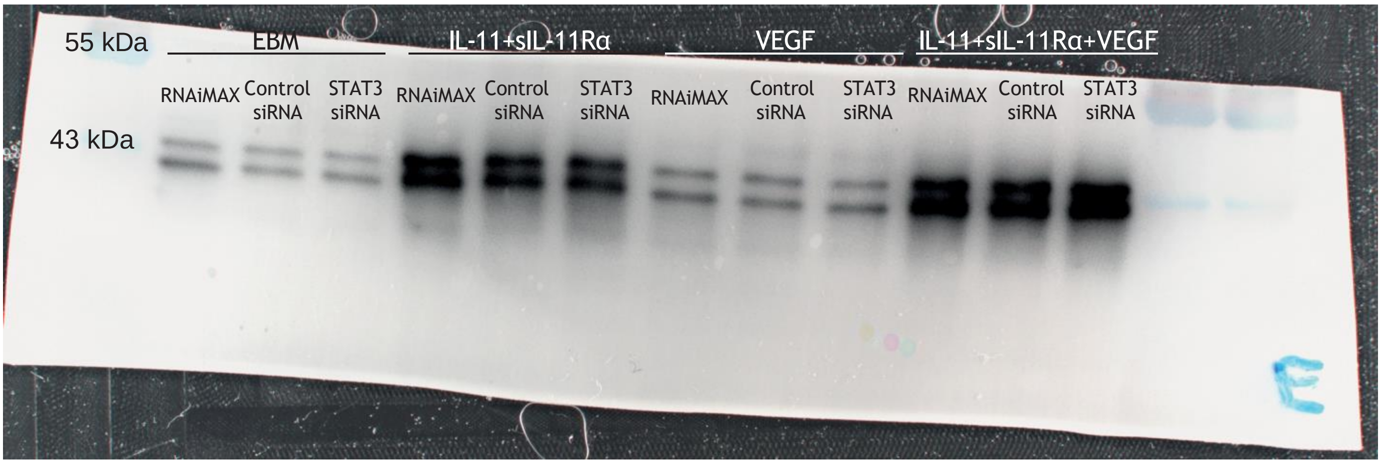

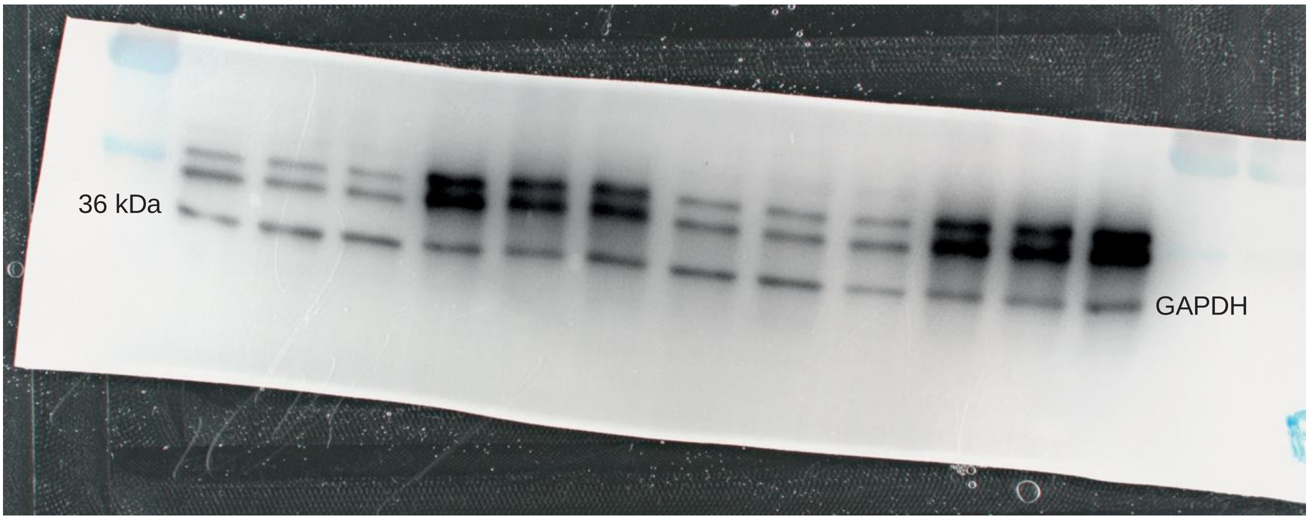

Full unedited blot for Figure 6C #1

STAT1

84, 91 kDa

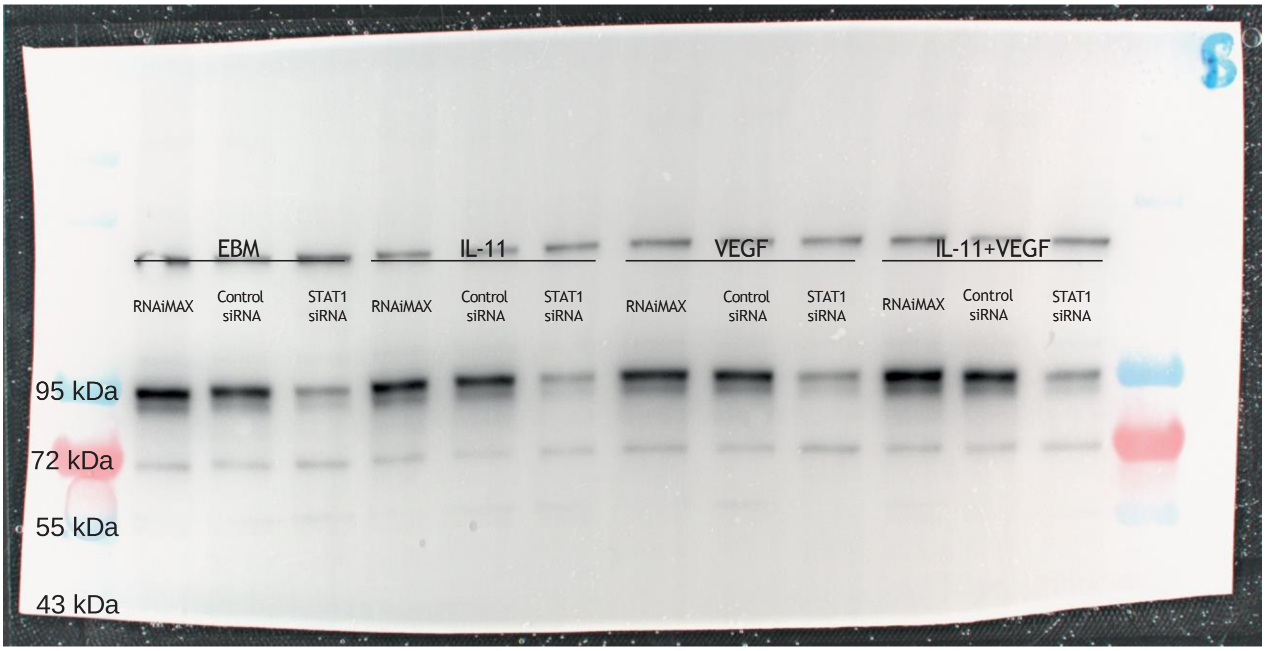

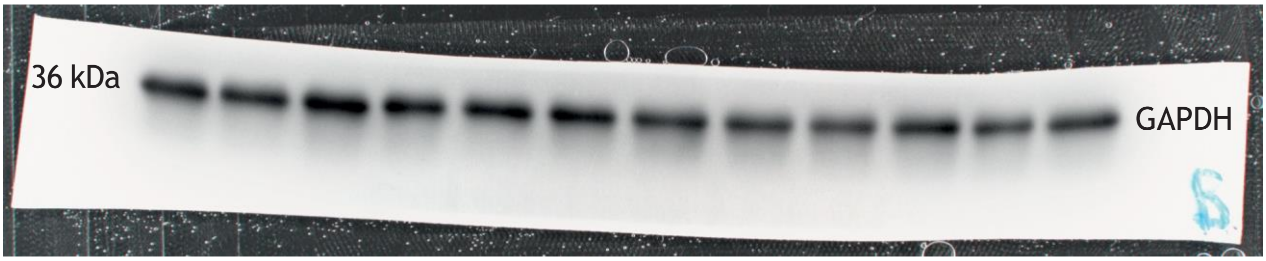

pSTAT3 Tyr

79, 86 kDa

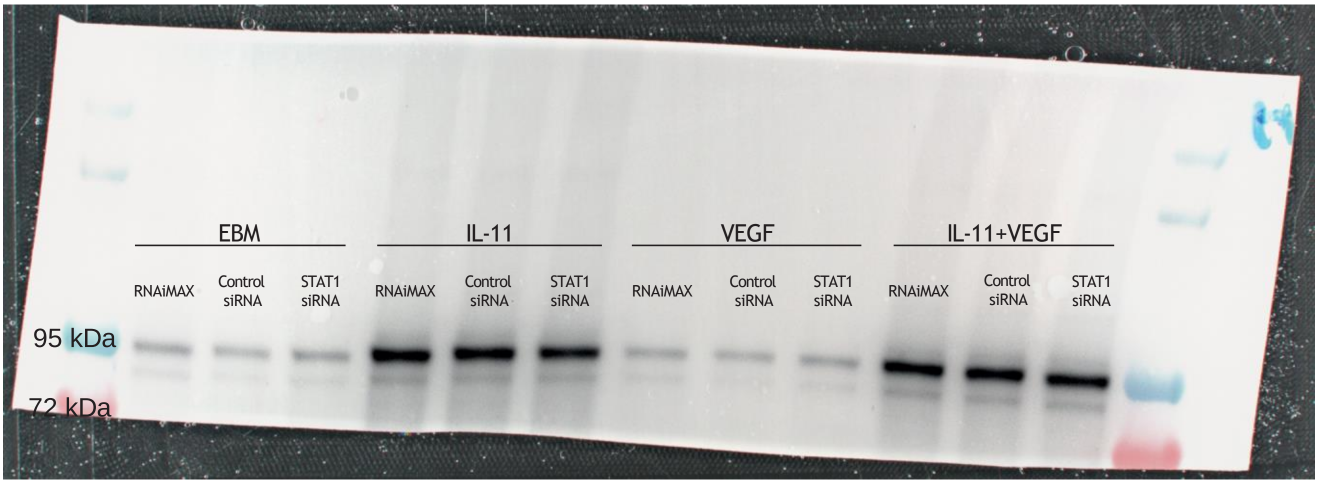

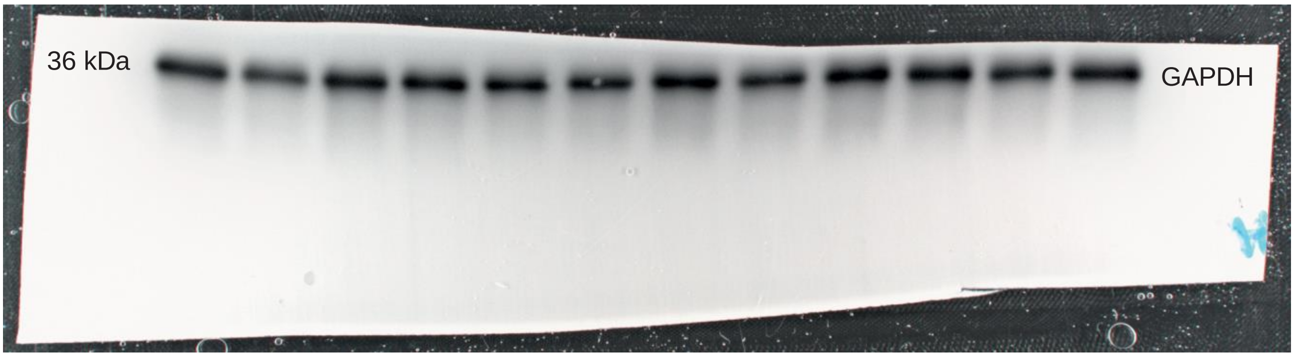

Full unedited blot for Figure 6C

#1

pSTAT3 Ser 86 kDa

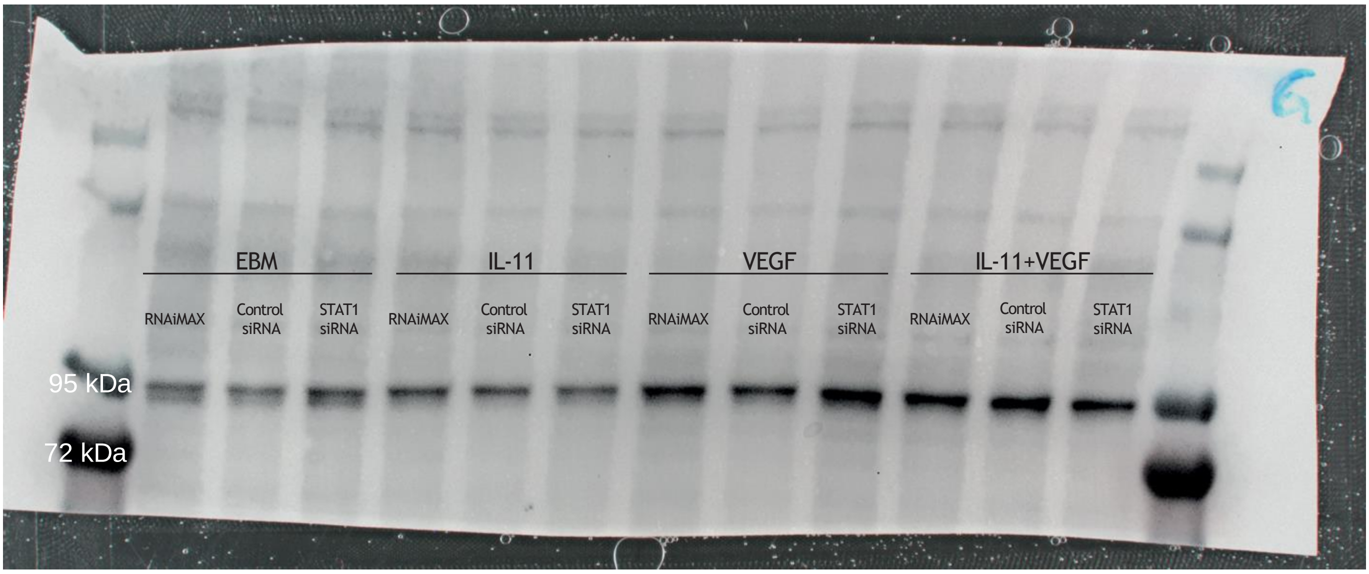

pERK 44, 42 kDa

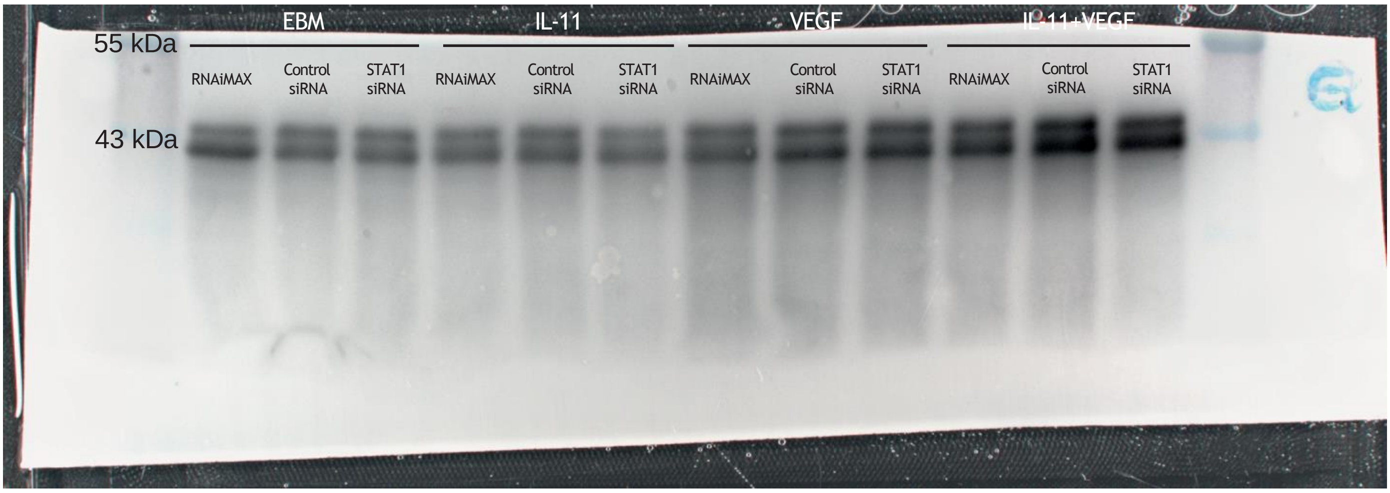

GAPDH 36 kDa

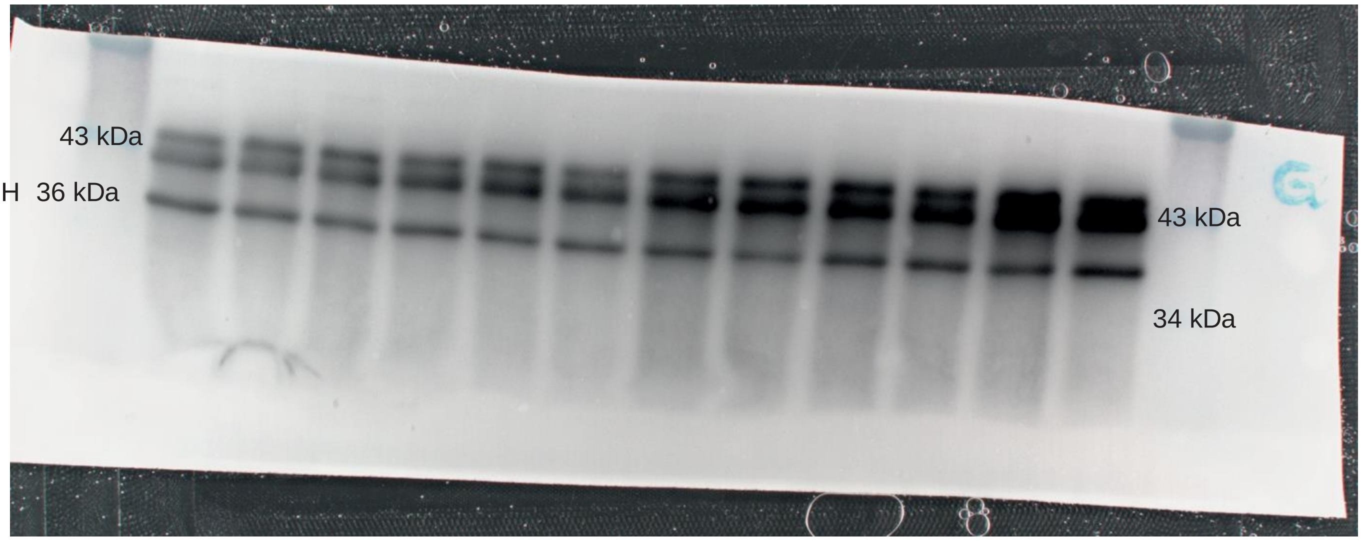

Full unedited blot for Figure 6C #1

pSTAT5
90 kDa

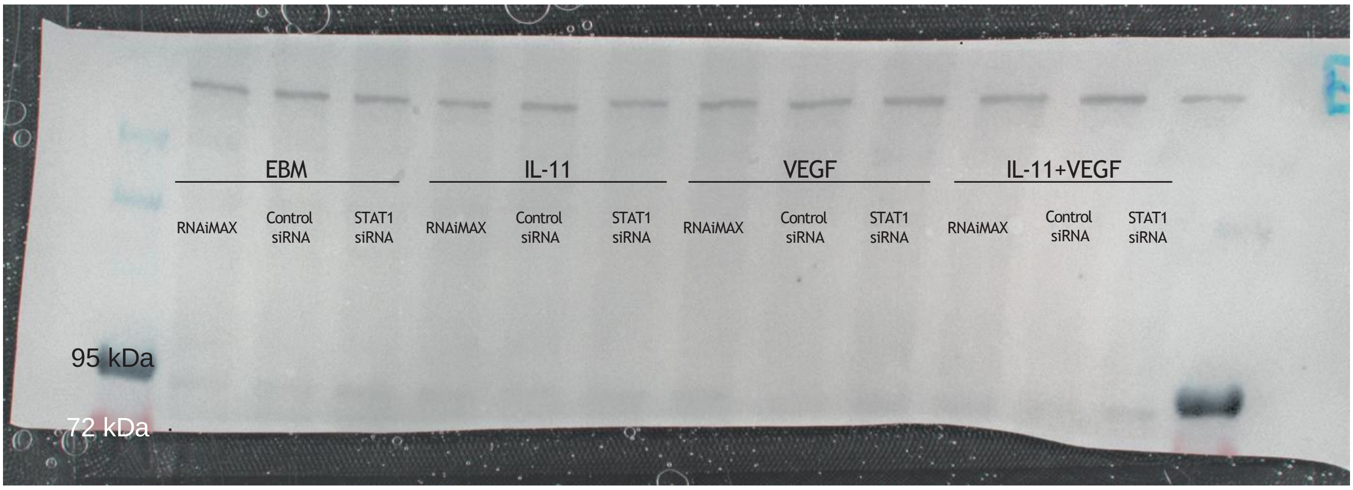

EBM | IL-11 | VEGF | IL-11+VEGF

RNAiMAX | Control siRNA | STAT1 siRNA | RNAiMAX | Control siRNA | STAT1 siRNA | RNAiMAX | Control siRNA | STAT1 siRNA | RNAiMAX | Control siRNA | STAT1 siRNA

pAkt

60 kDa

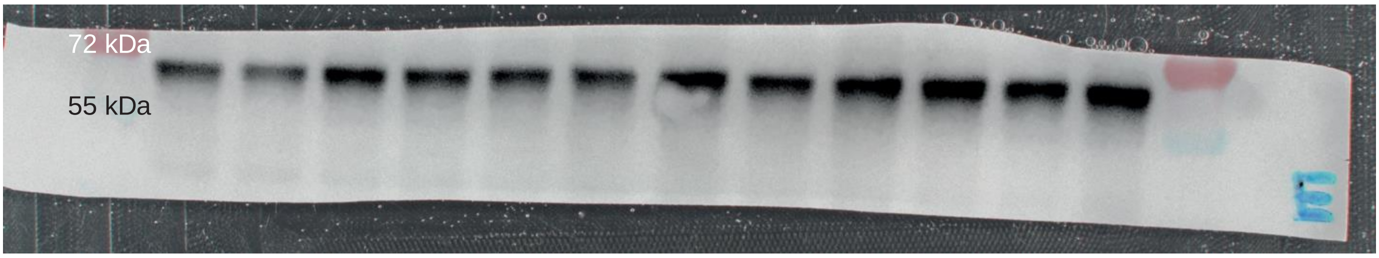

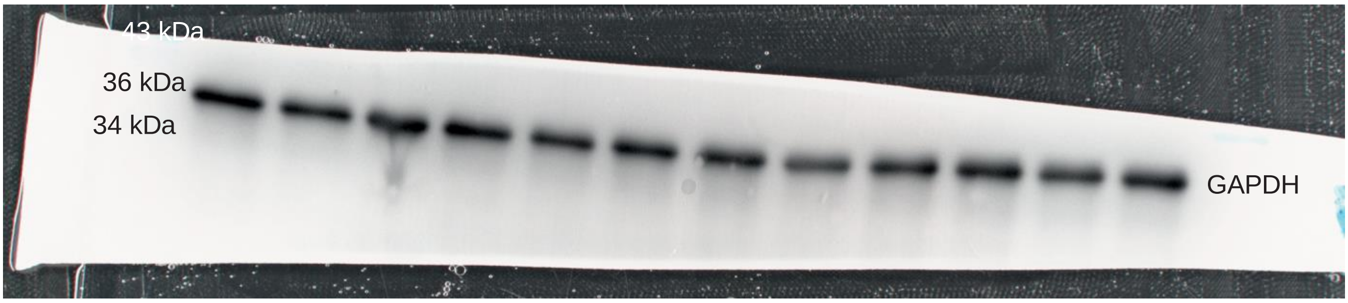

Full unedited blot for Figure 6C #2

STAT1
84, 91 kDa

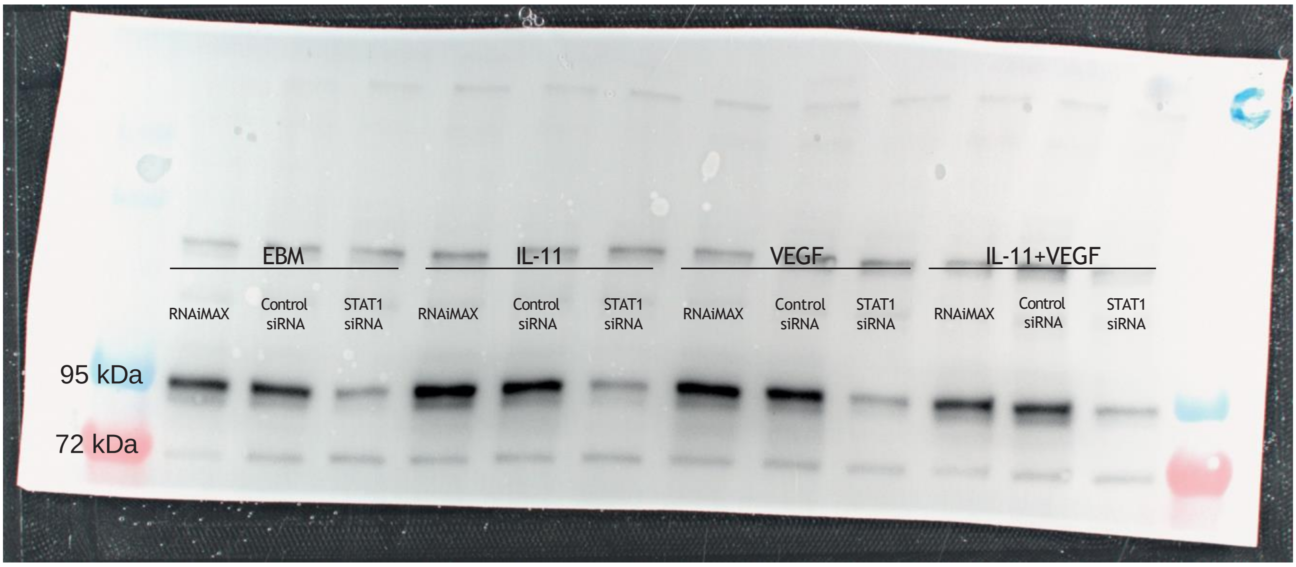

pERK
44, 42 kDa

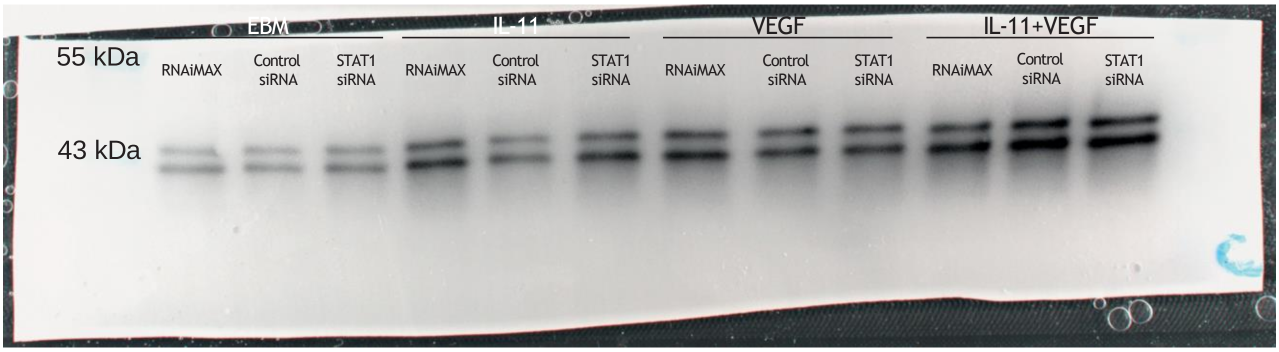

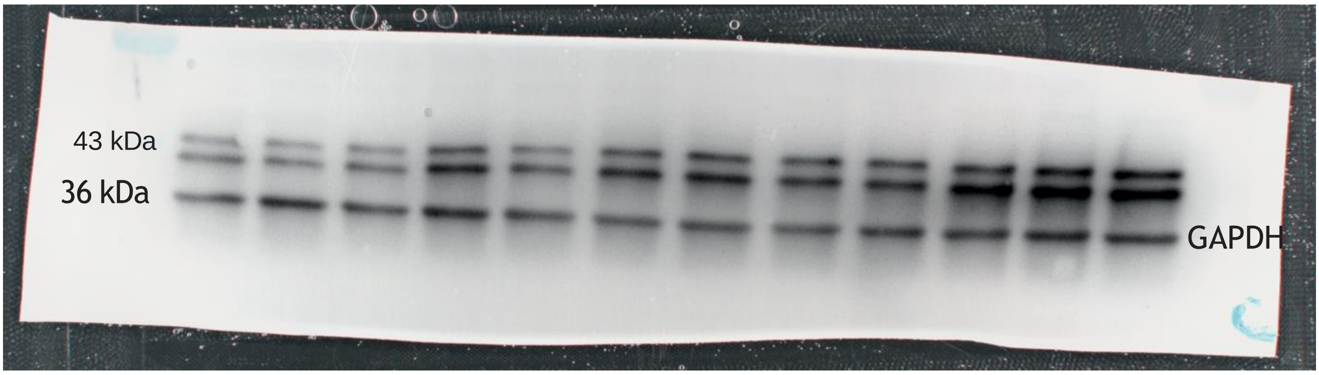

Full unedited blot for Figure 6C #2

pSTAT3 Tyr

79, 86 kDa

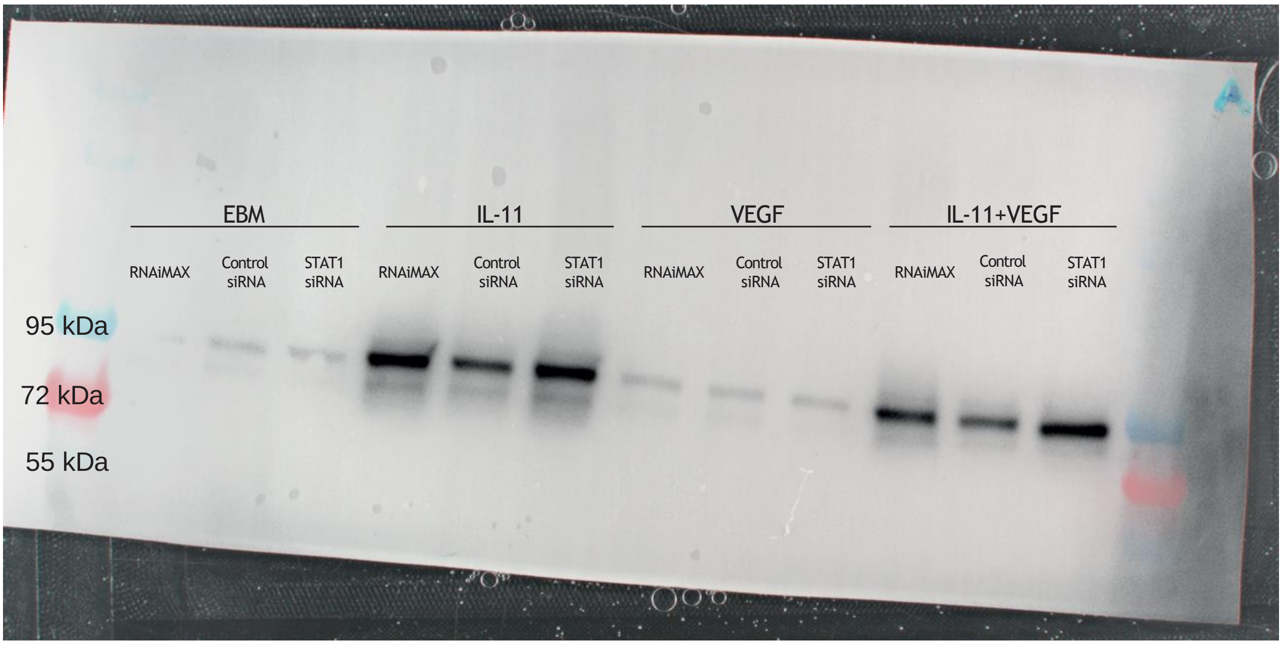

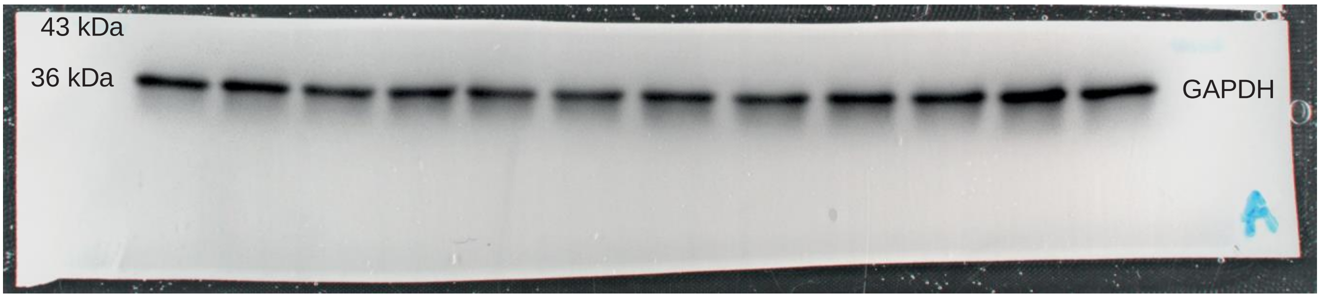

pSTAT3 Ser

86 kDa

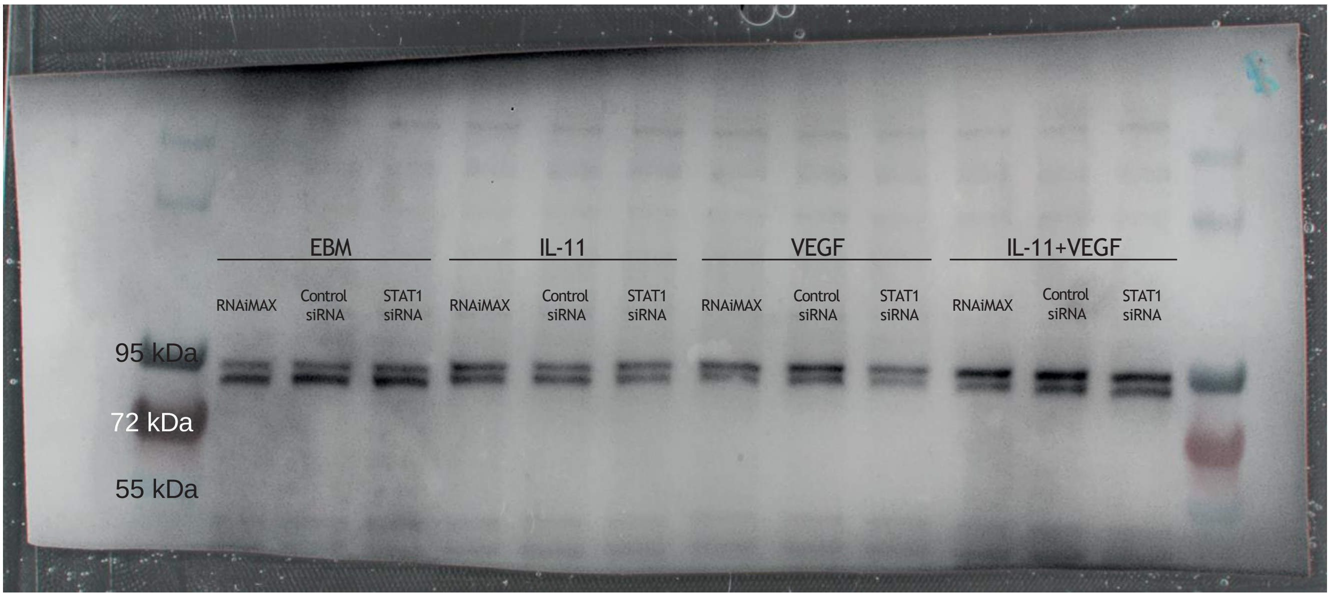

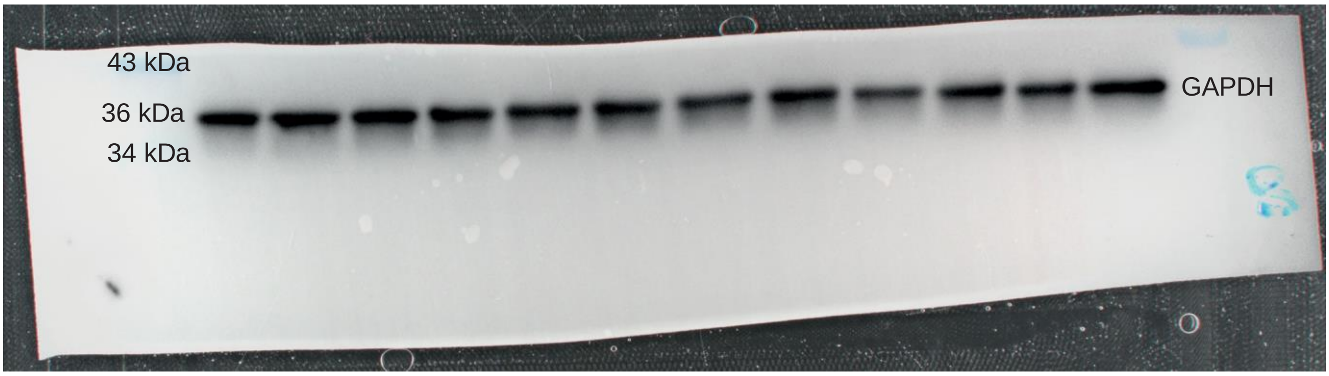

Full unedited blot for Figure 6C #2

pSTAT5

90 kDa

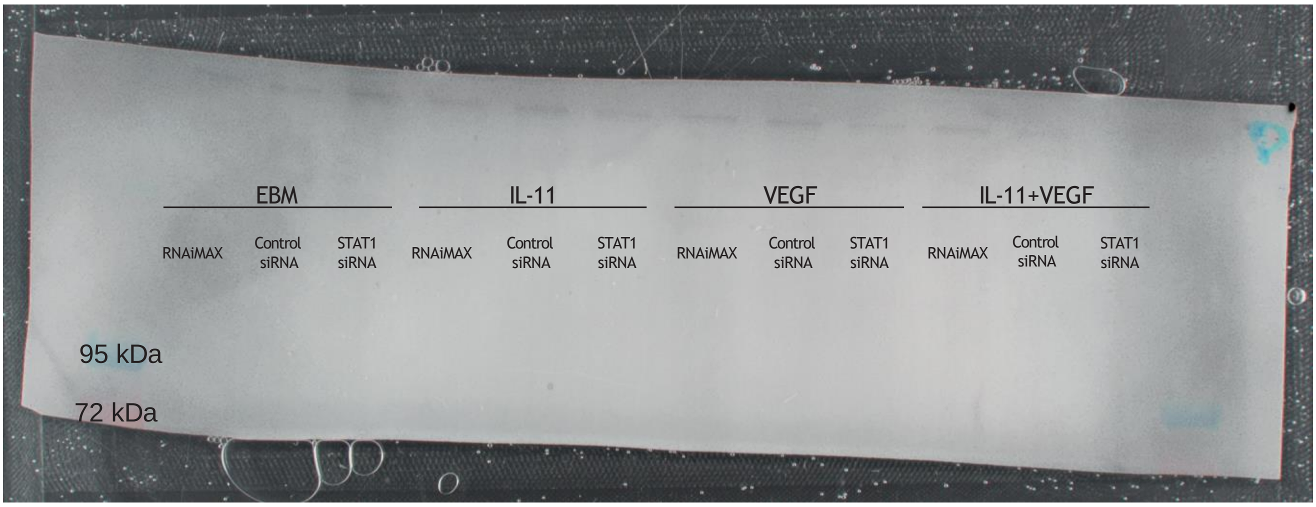

EBM IL-11 VEGF IL-11+VEGF

RNAiMAX Control siRNA STAT1 siRNA RNAiMAX Control siRNA STAT1 siRNA RNAiMAX Control siRNA STAT1 siRNA RNAiMAX Control siRNA STAT1 siRNA

pAkt

60 kDa

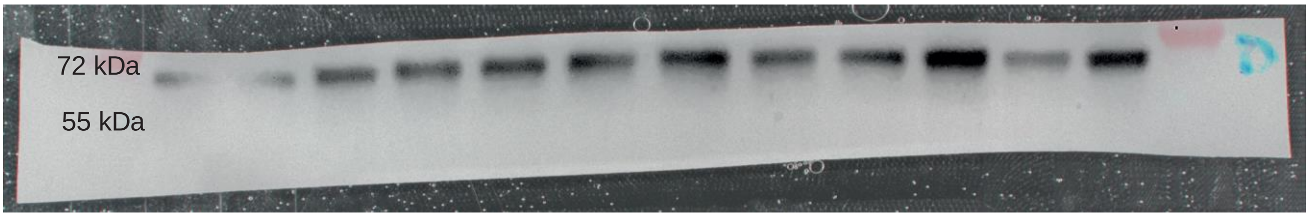

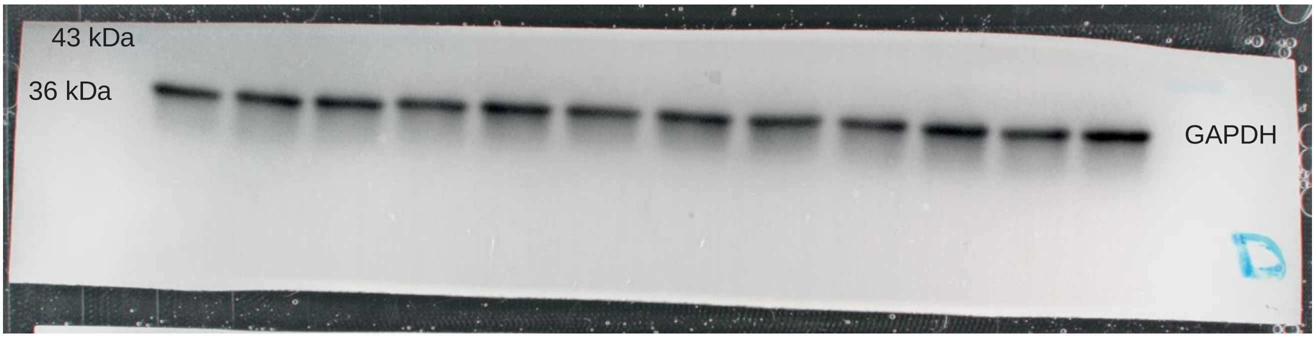

Full unedited blot for Figure 6C #3

STAT1
84, 91 kDa

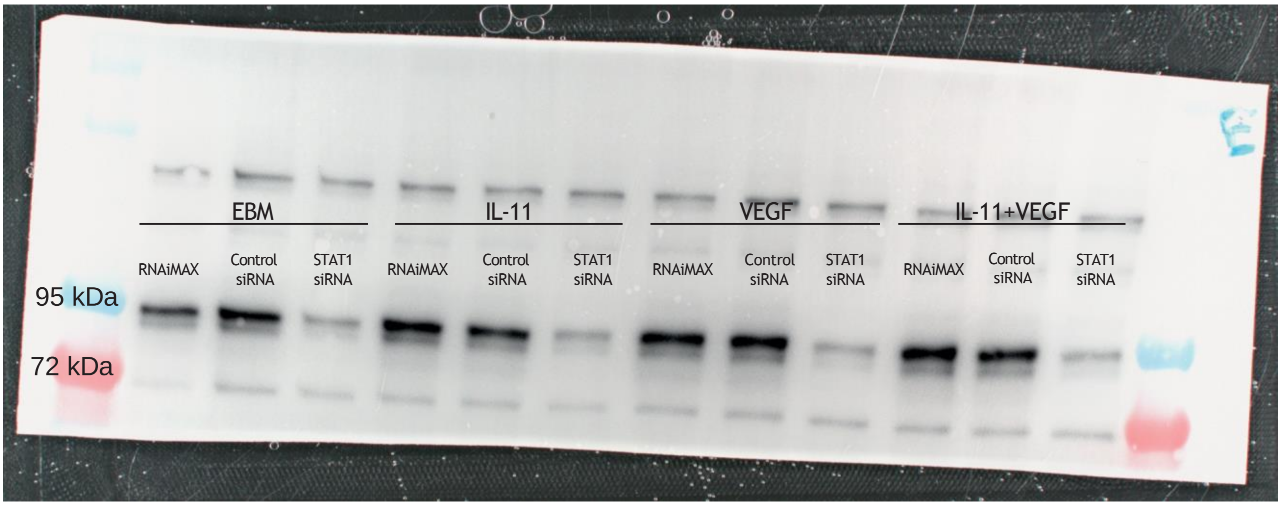

pERK
44, 42 kDa

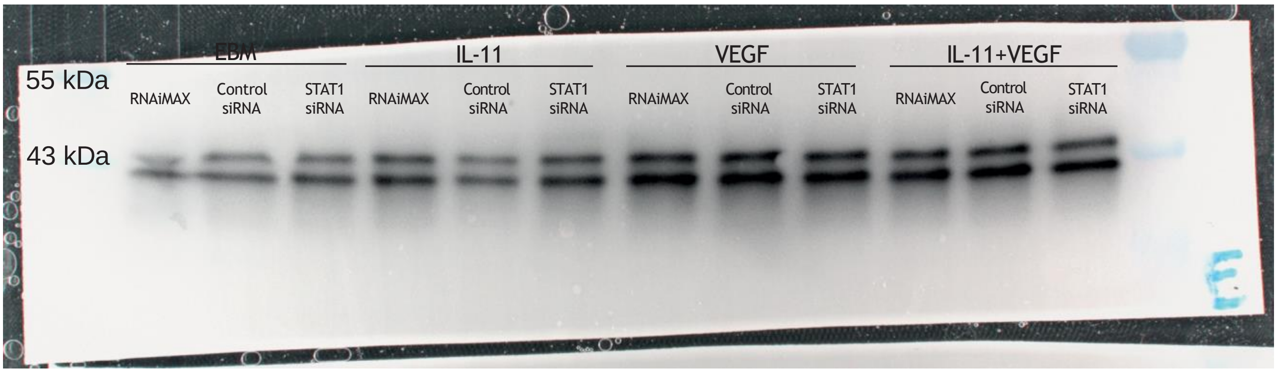

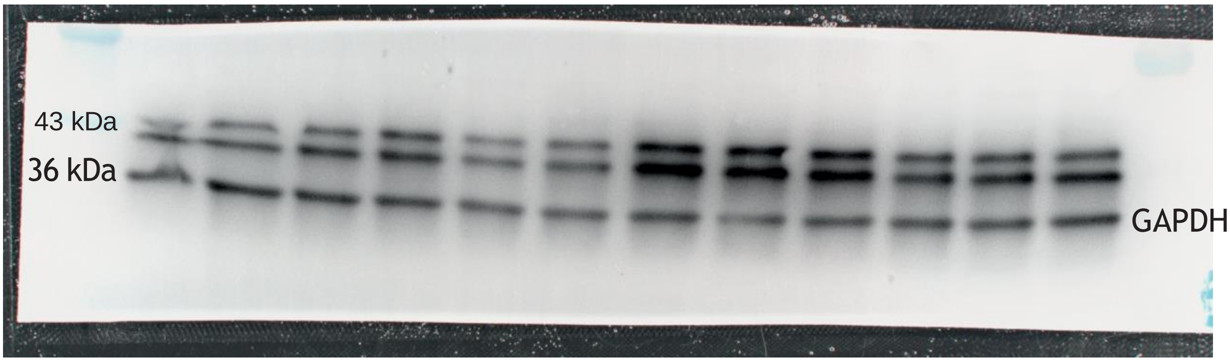

Full unedited blot for Figure 6C #3

pSTAT3 Tyr

79, 86 kDa

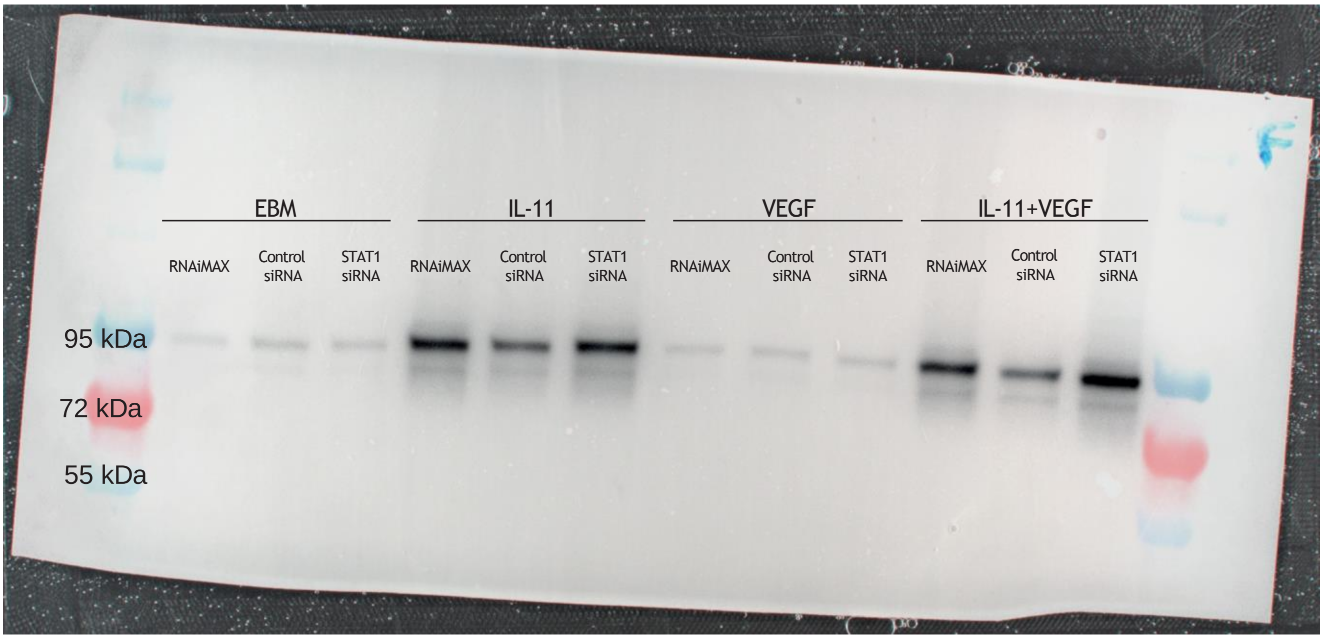

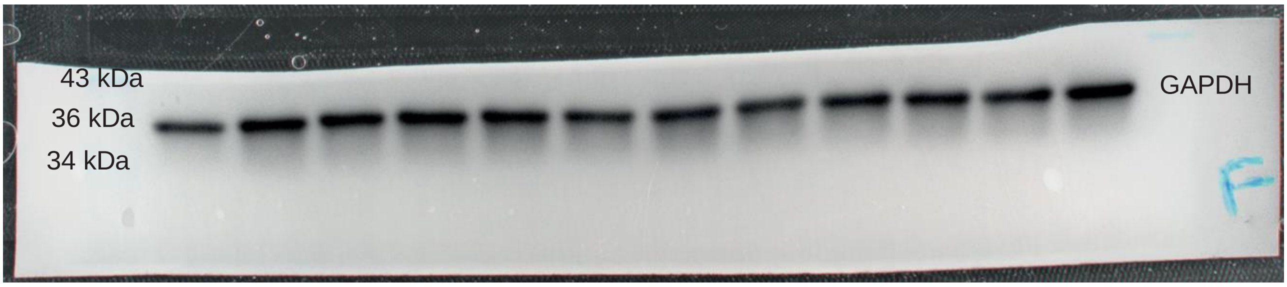

pSTAT3 Ser

86 kDa

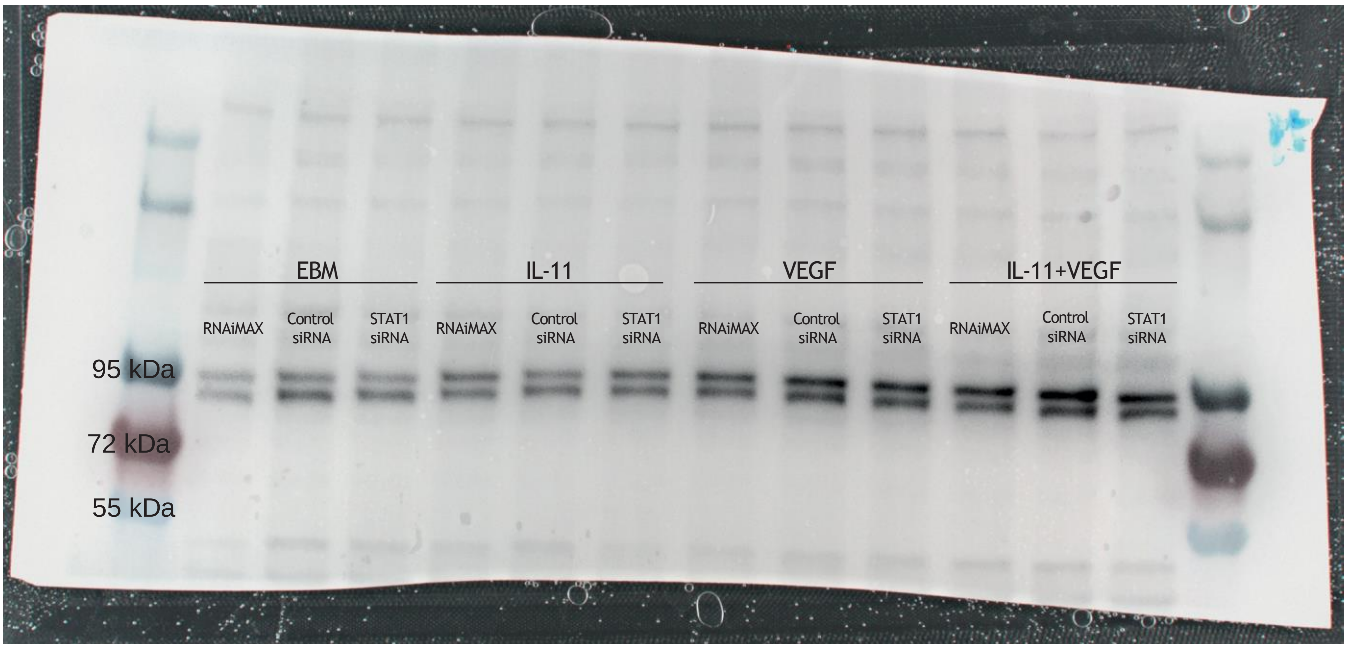

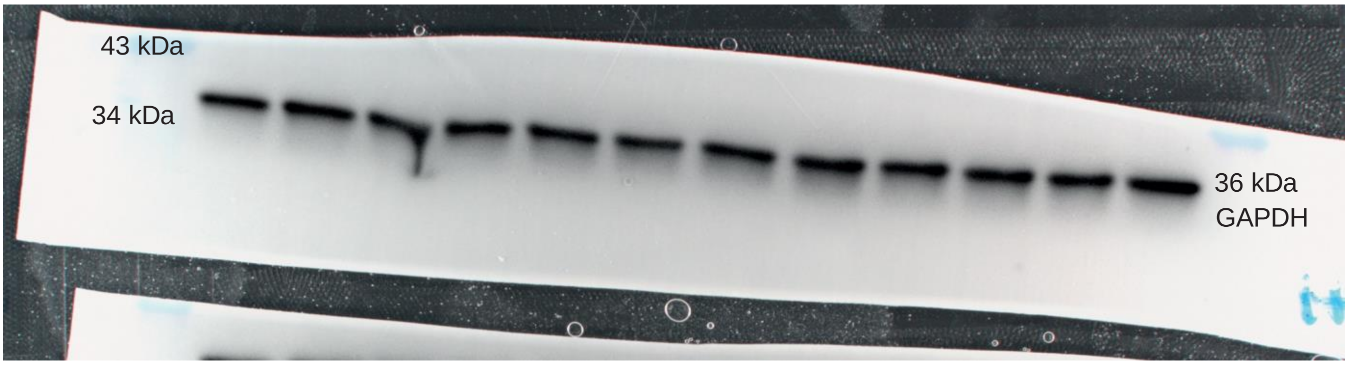

Full unedited blot for Figure 6C #3

pSTAT5

90 kDa

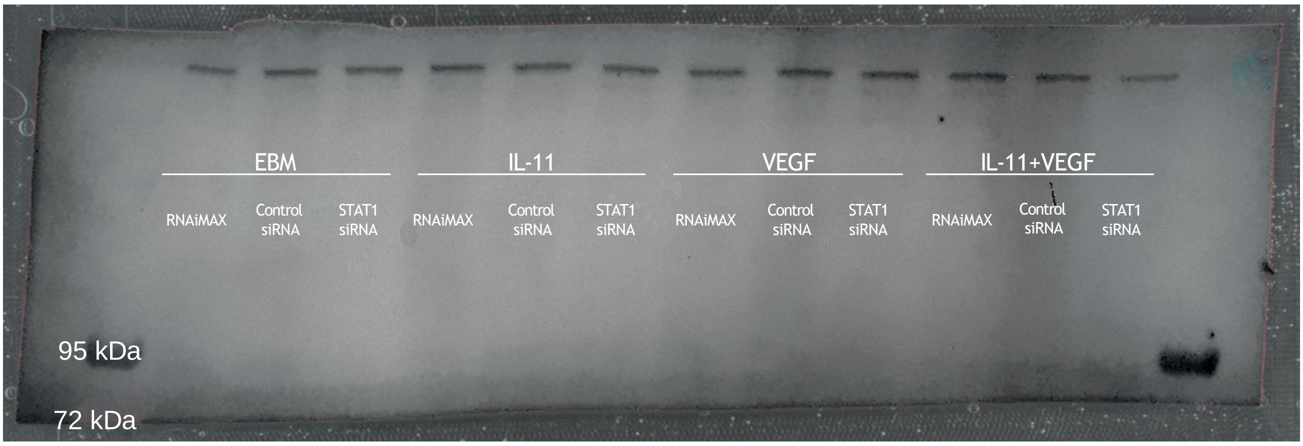

EBM IL-11 VEGF IL-11+VEGF

RNAiMAX Control siRNA STAT1 siRNA RNAiMAX Control siRNA STAT1 siRNA RNAiMAX Control siRNA STAT1 siRNA RNAiMAX Control siRNA STAT1 siRNA

pAkt

60 kDa

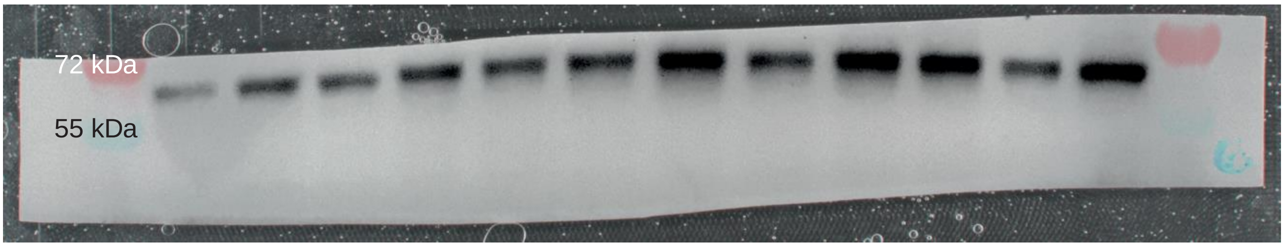

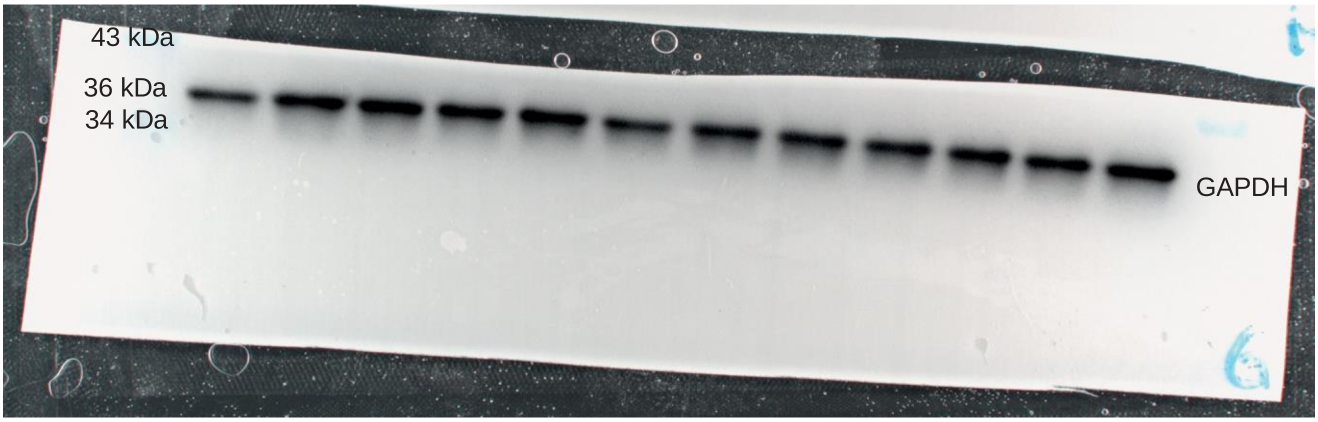

Full unedited blot for Figure 6D #1

STAT1

84, 91 kDa

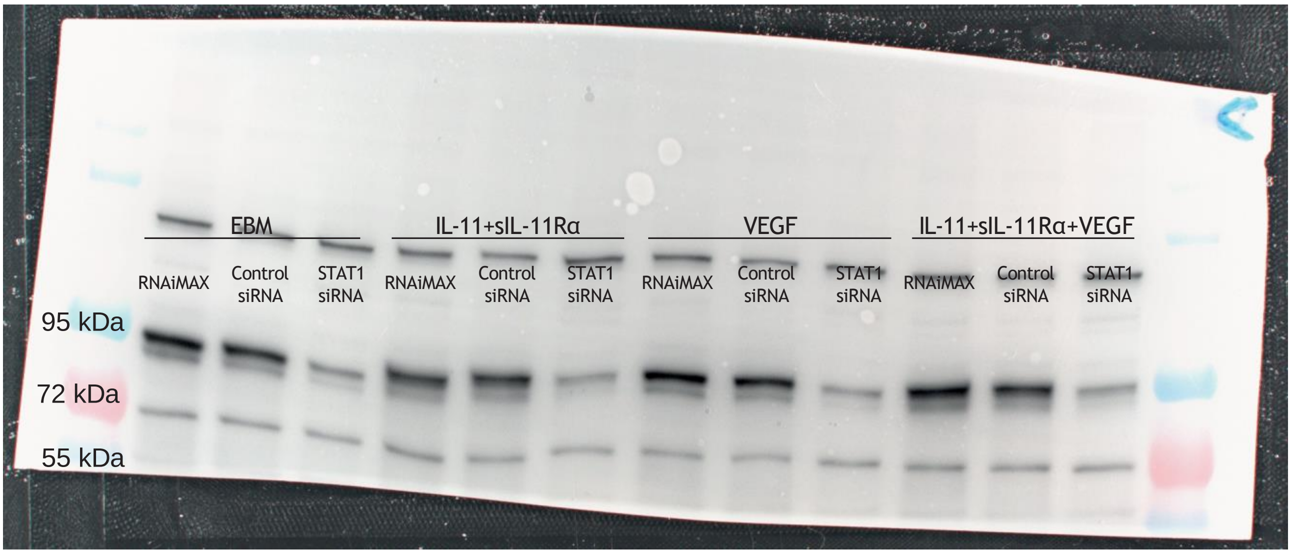

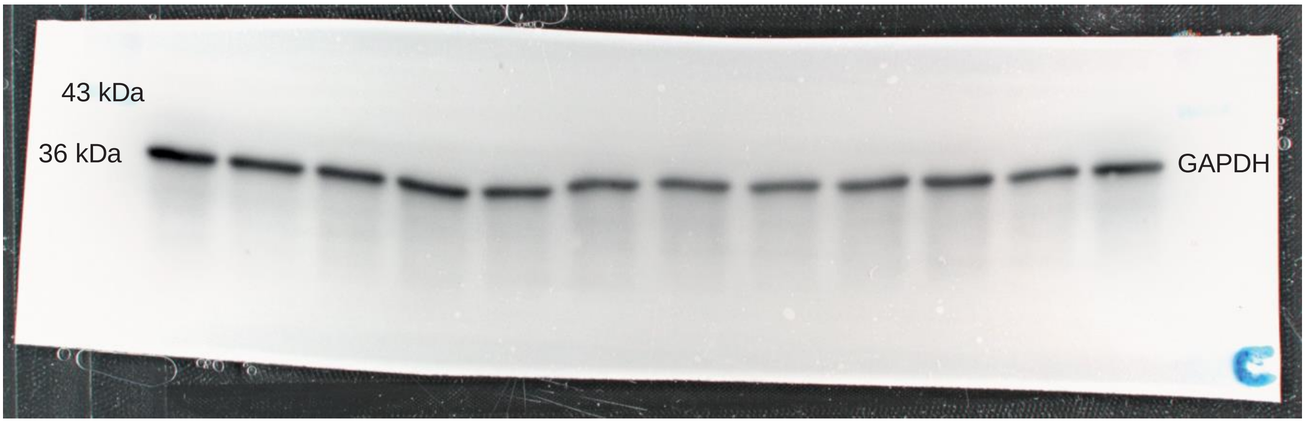

pSTAT3 Ser

86 kDa

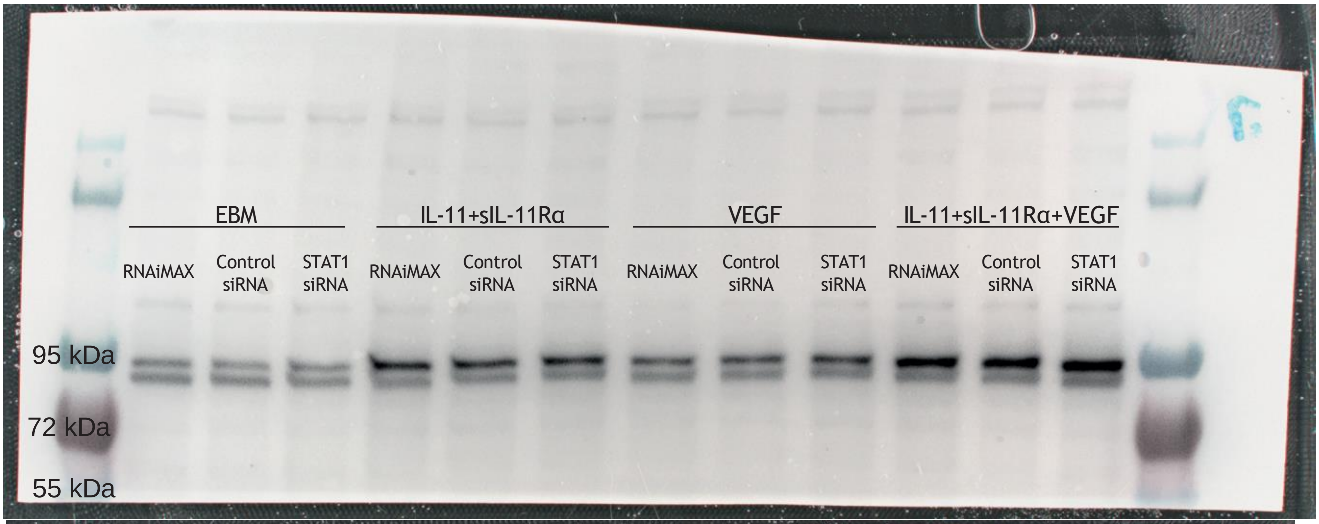

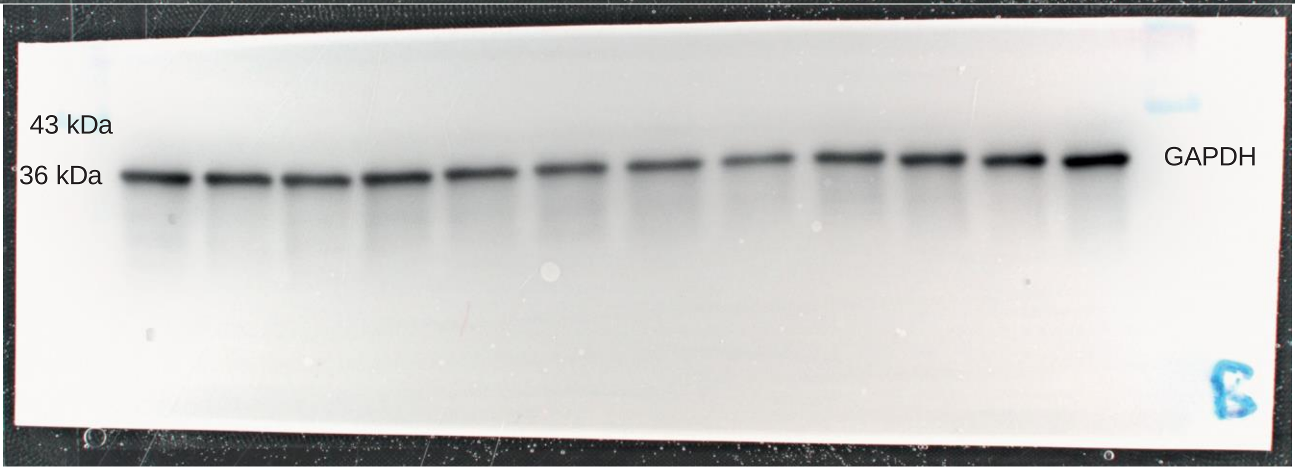

Full unedited blot for Figure 6D #1

pSTAT3 Tyr

79, 86 kDa

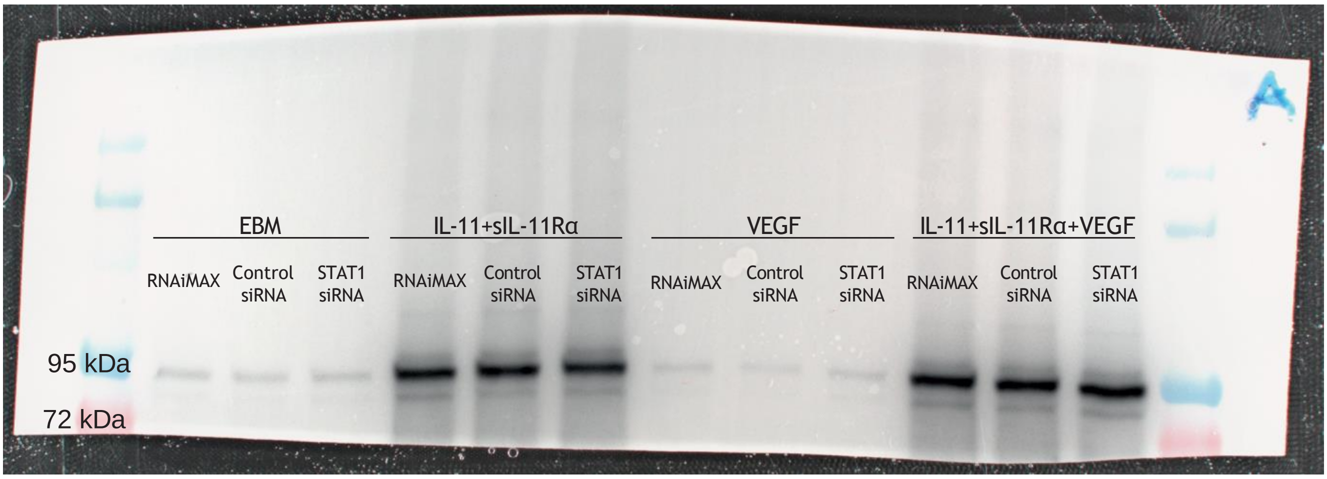

pERK

44, 42 kDa

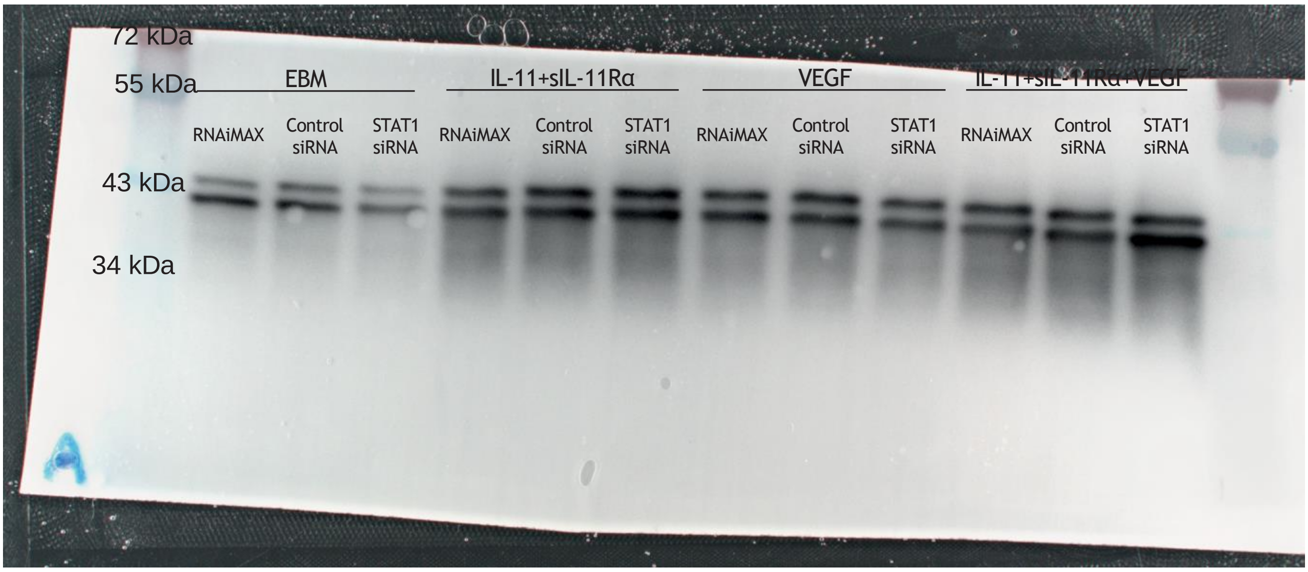

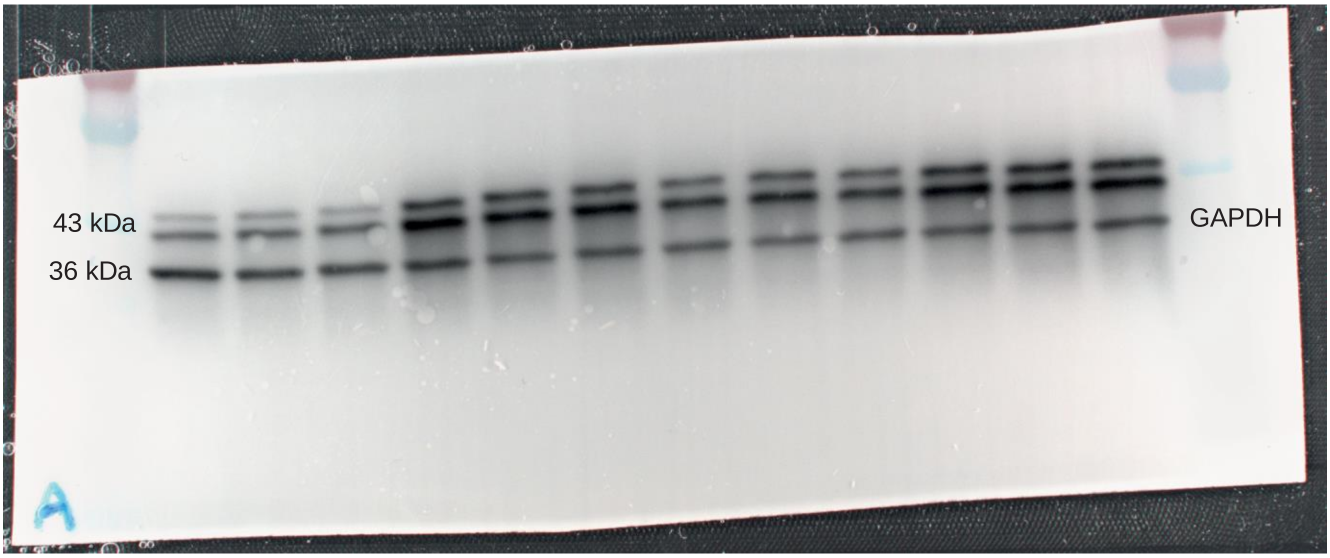

Full unedited blot for Figure 6D #1

pSTAT5

90 kDa

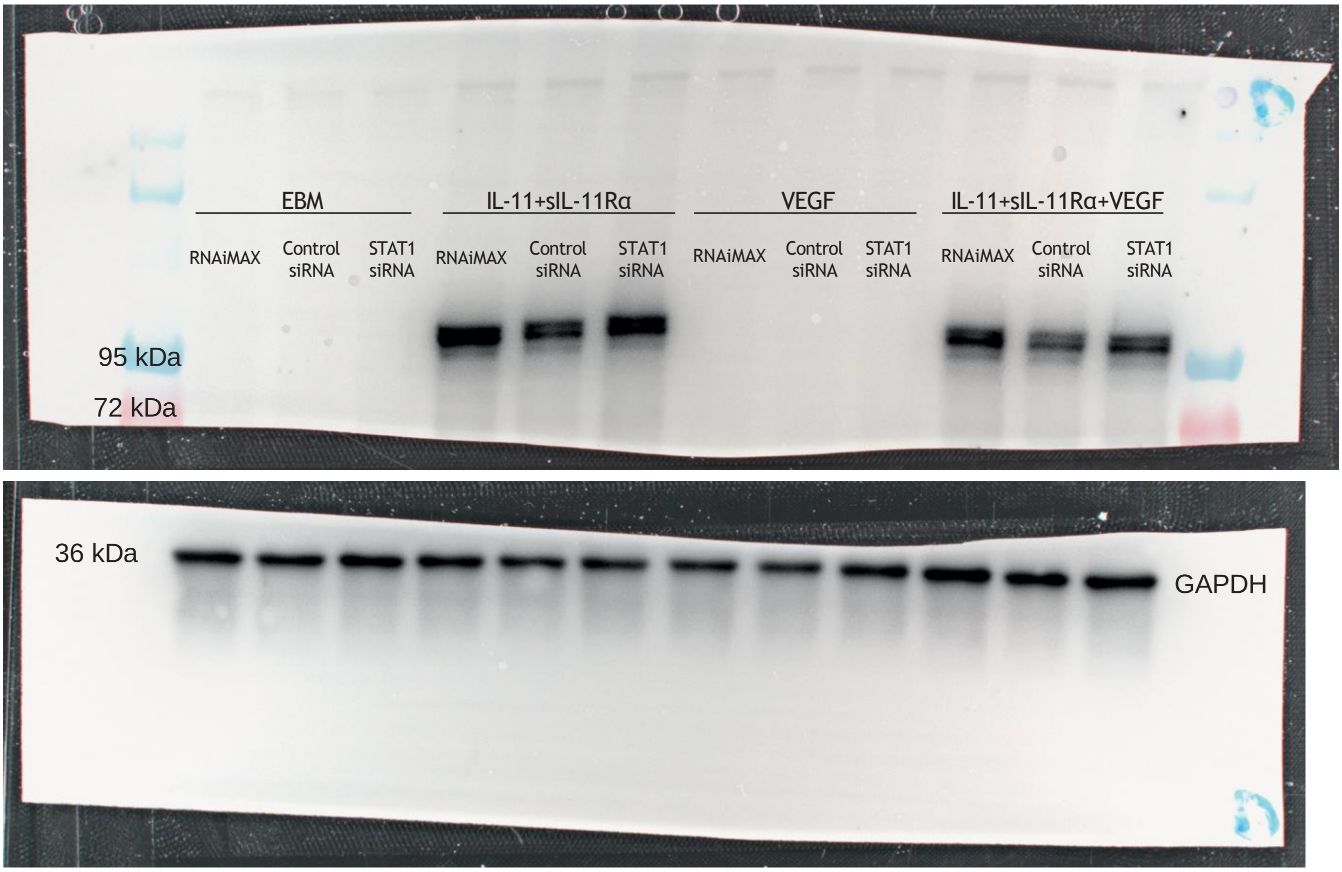

pAkt

60 kDa

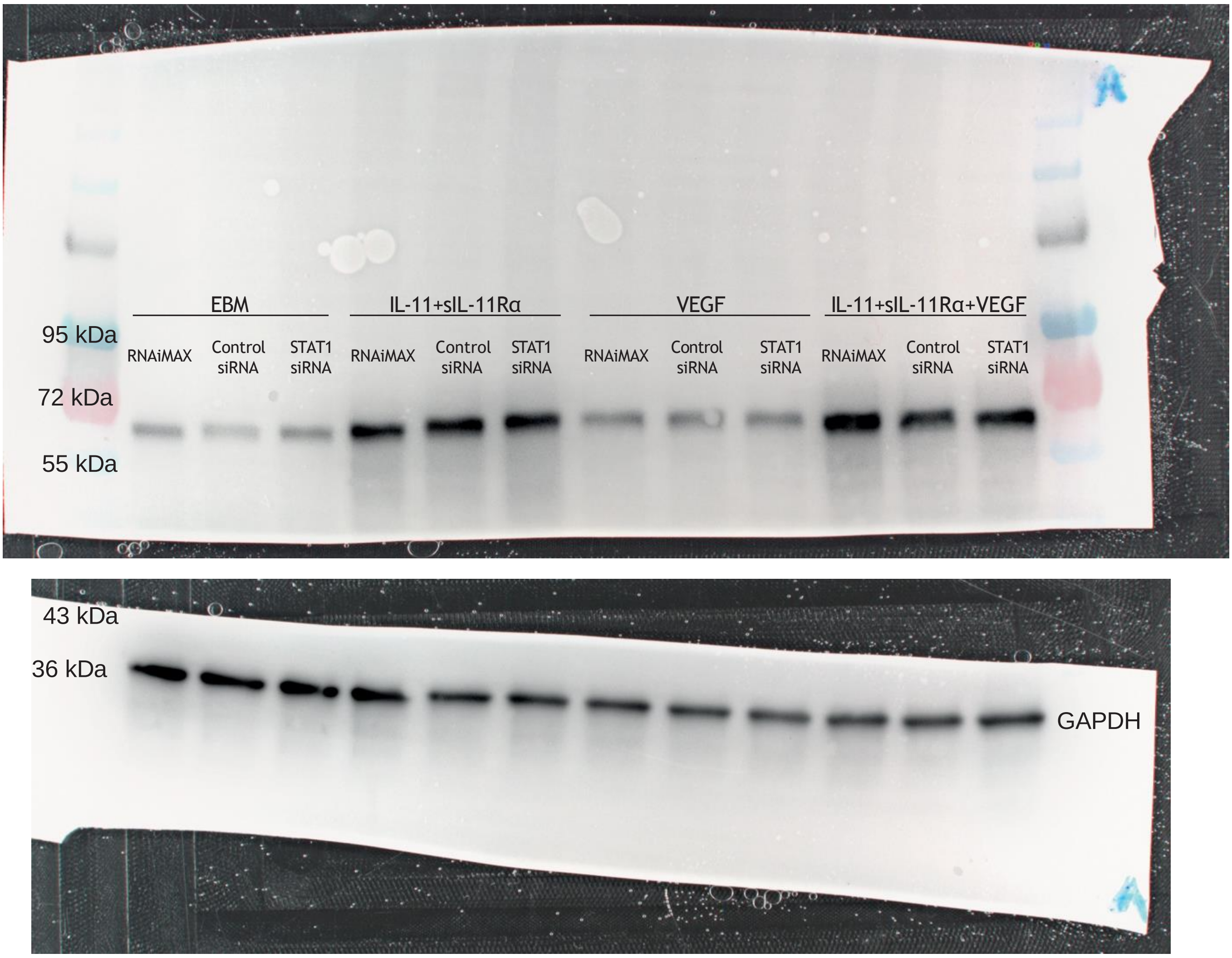

Full unedited blot for Figure 6D #2

STAT1

84, 91 kDa

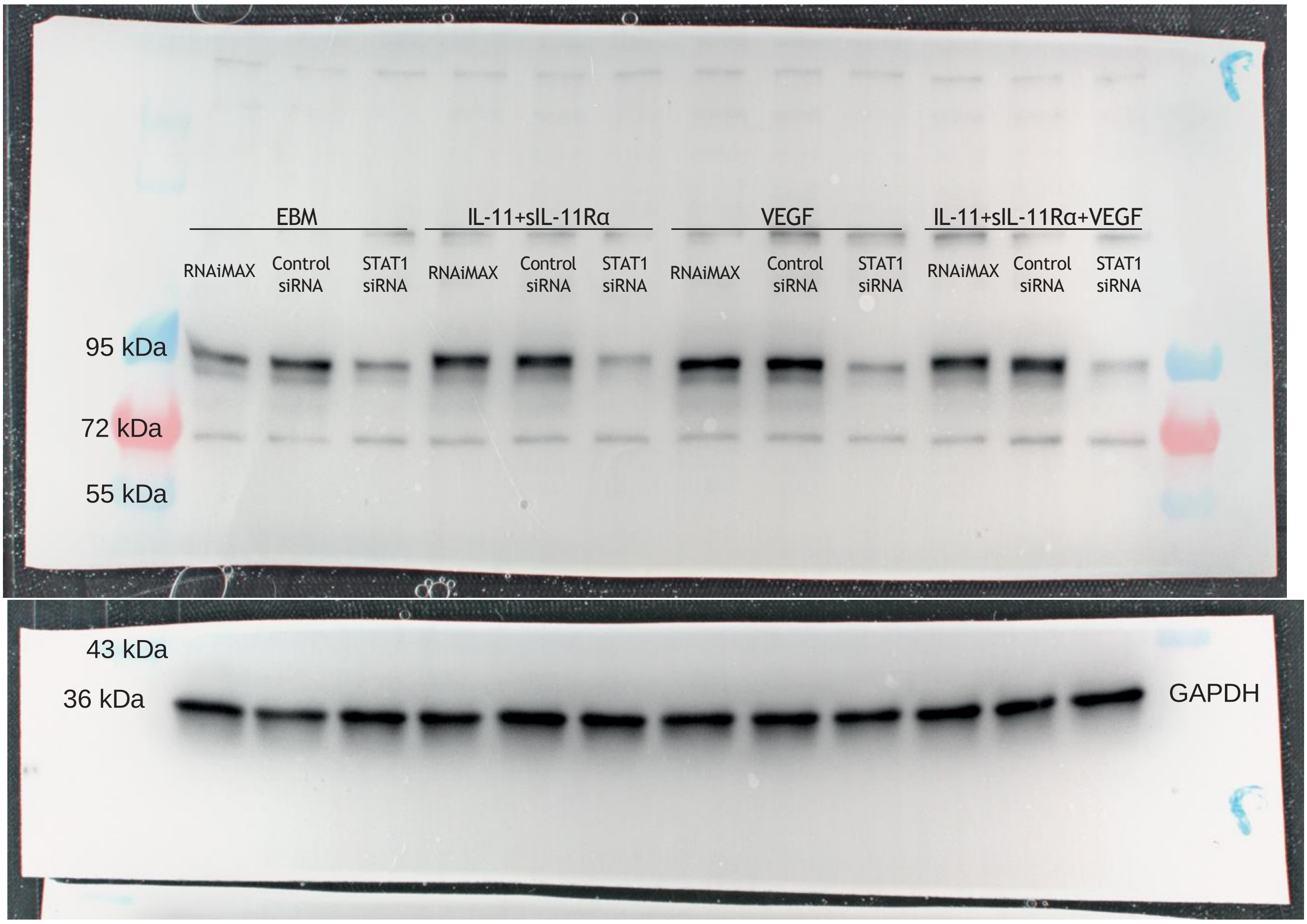

pSTAT3 Ser
86 kDa

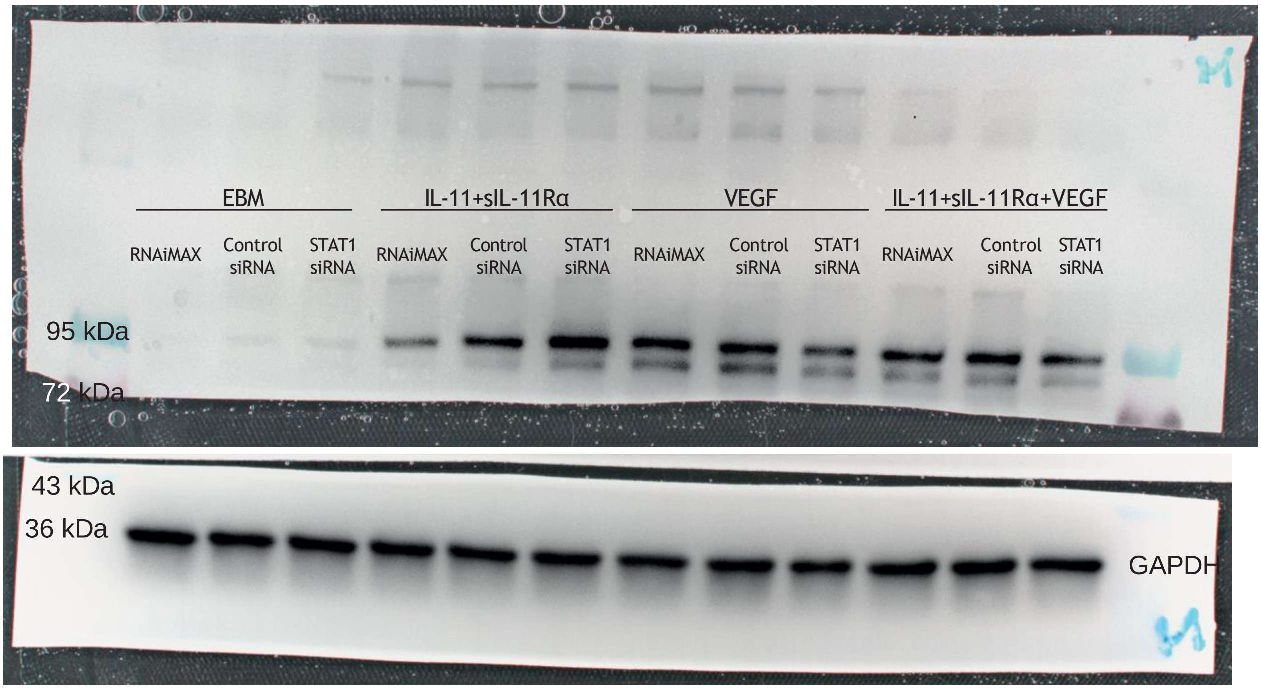

Full unedited blot for Figure 6D #2

pSTAT3 Tyr

79, 86 kDa

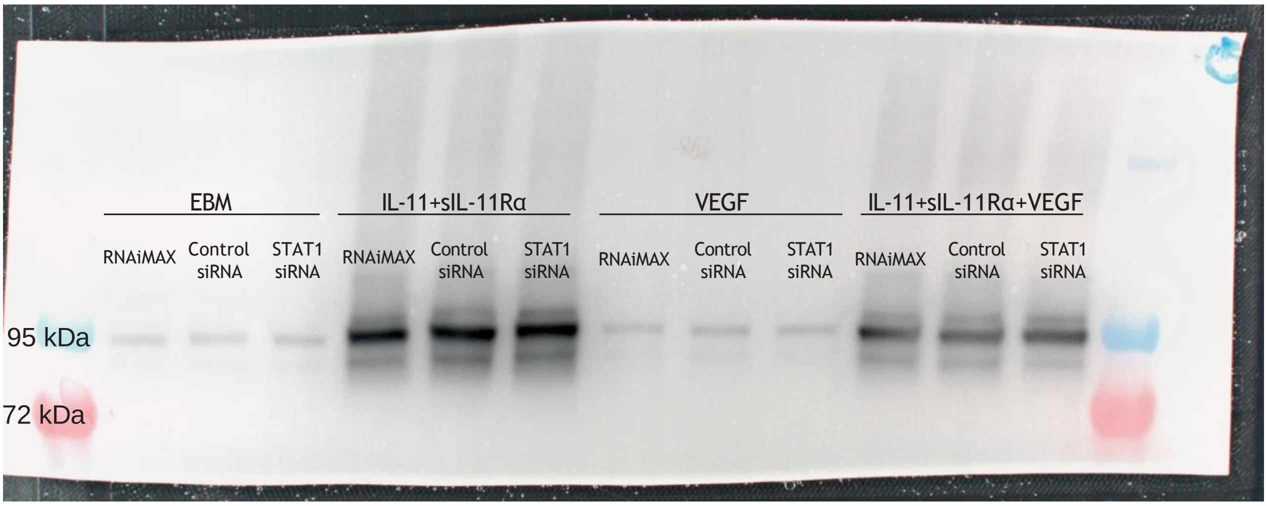

pERK

44, 42 kDa

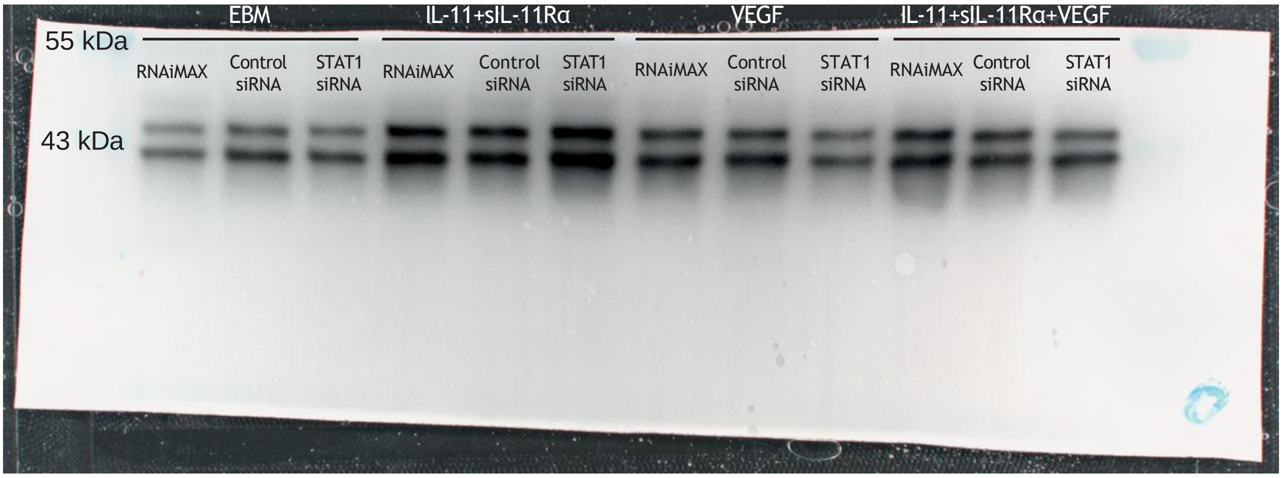

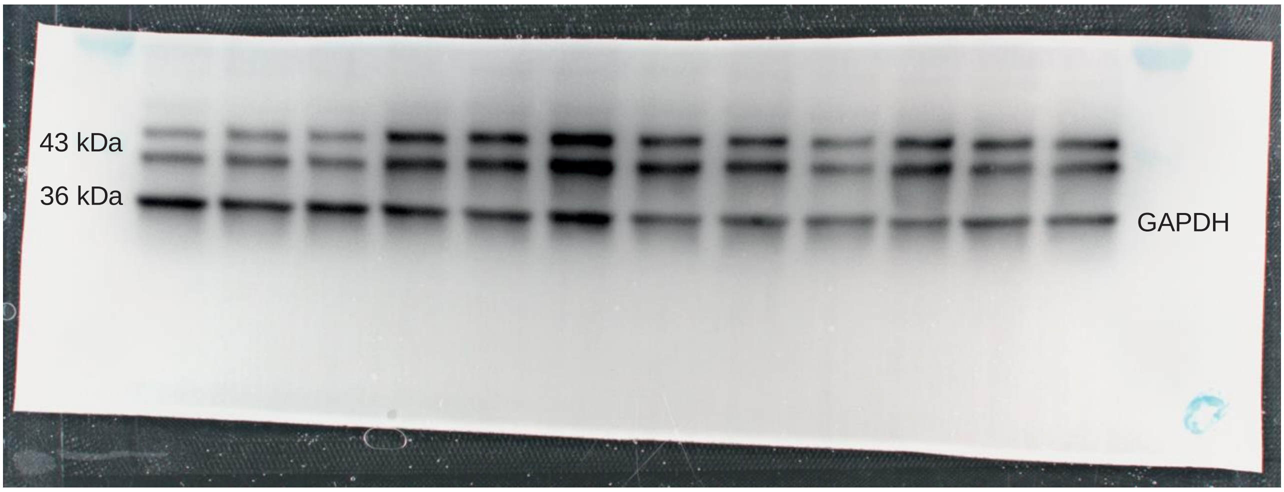

Full unedited blot for Figure 6D #2

pSTAT5

90 kDa

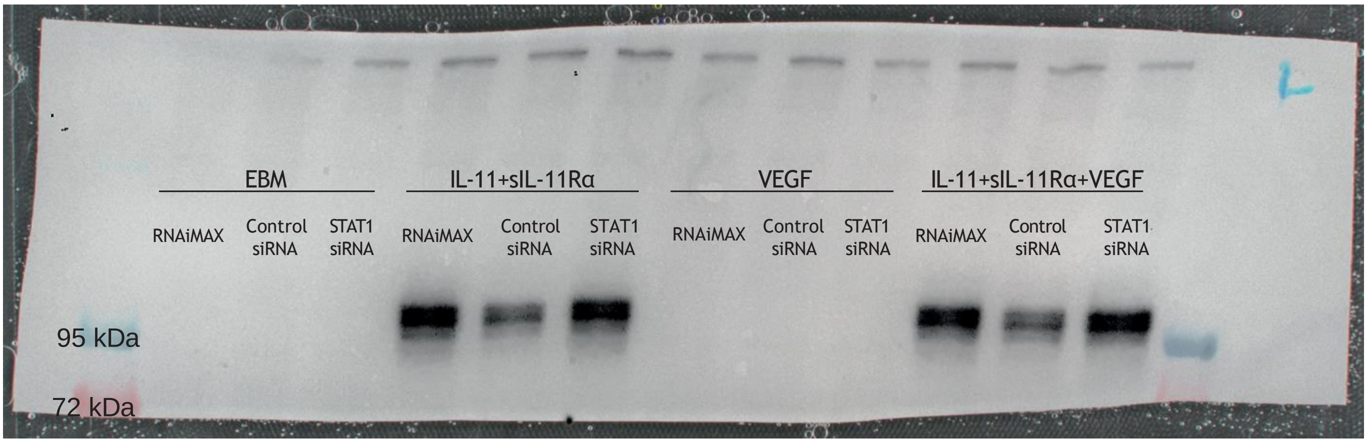

pAkt

60 kDa

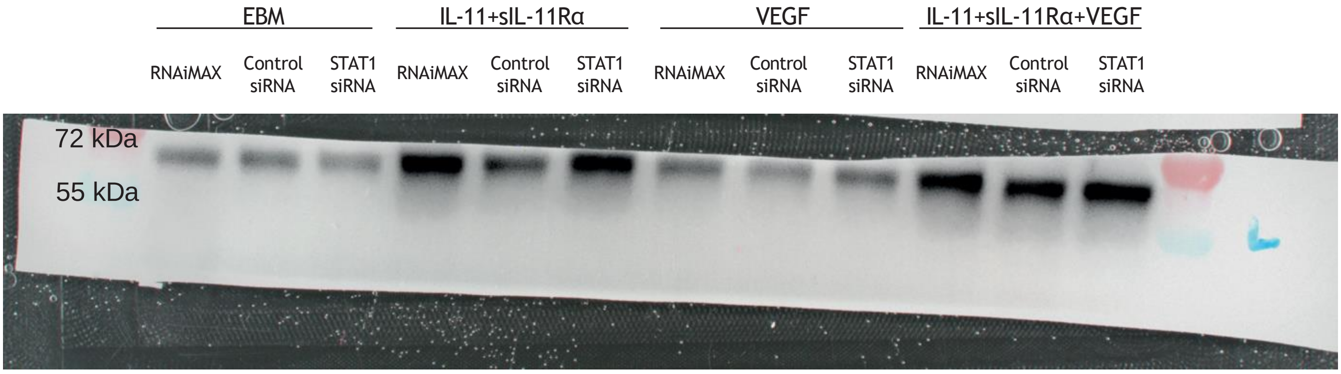

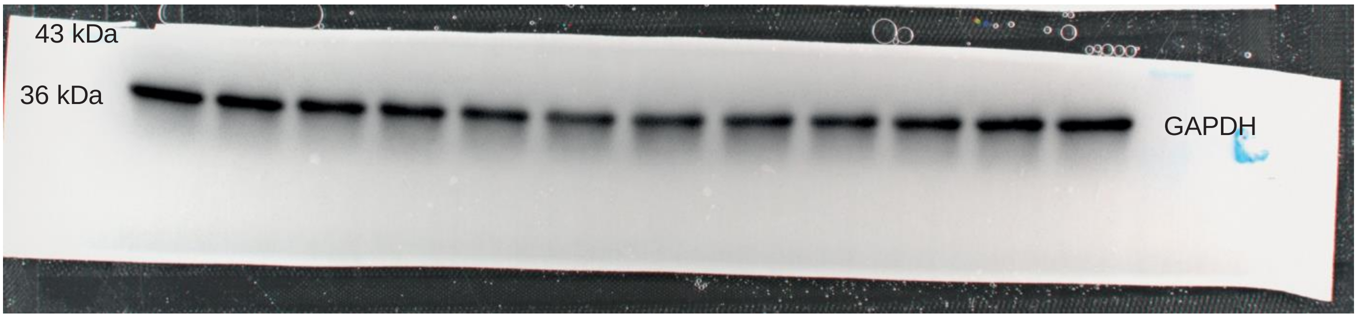

Full unedited blot for Figure 6D #3

STAT1

84, 91 kDa

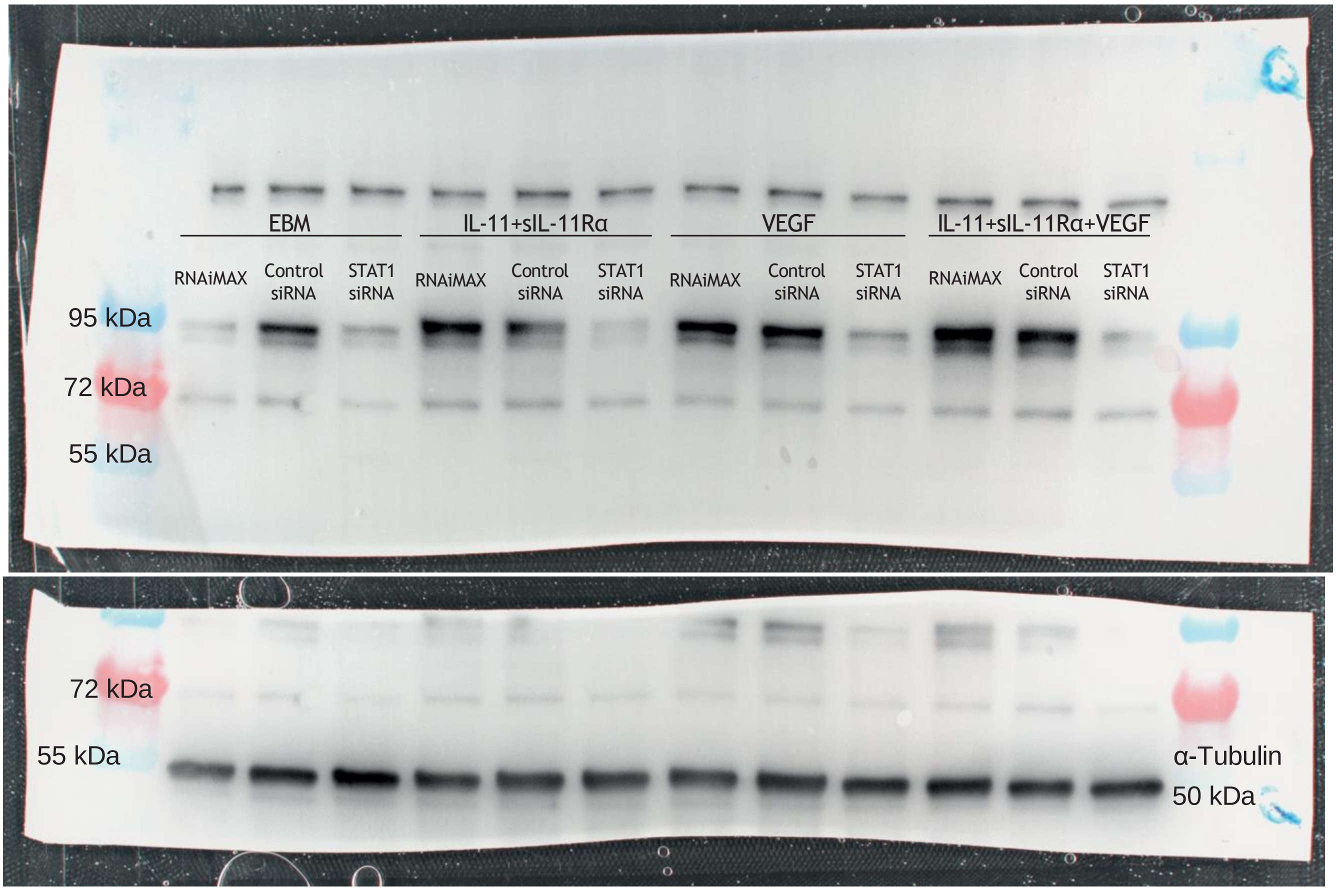

pSTAT3 Tyr

79, 86 kDa

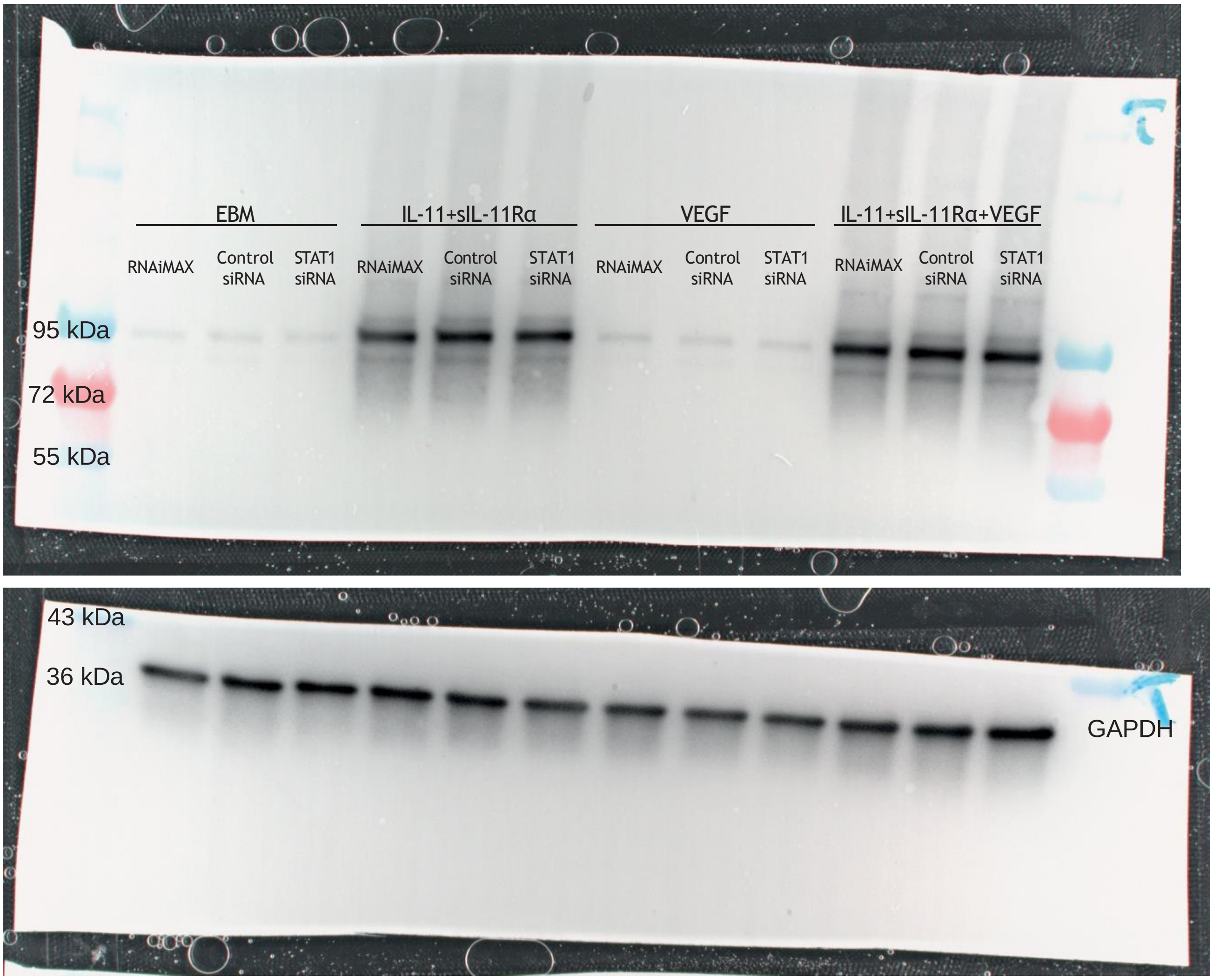

Full unedited blot for Figure 6D #3

pSTAT3 Ser
86 kDa

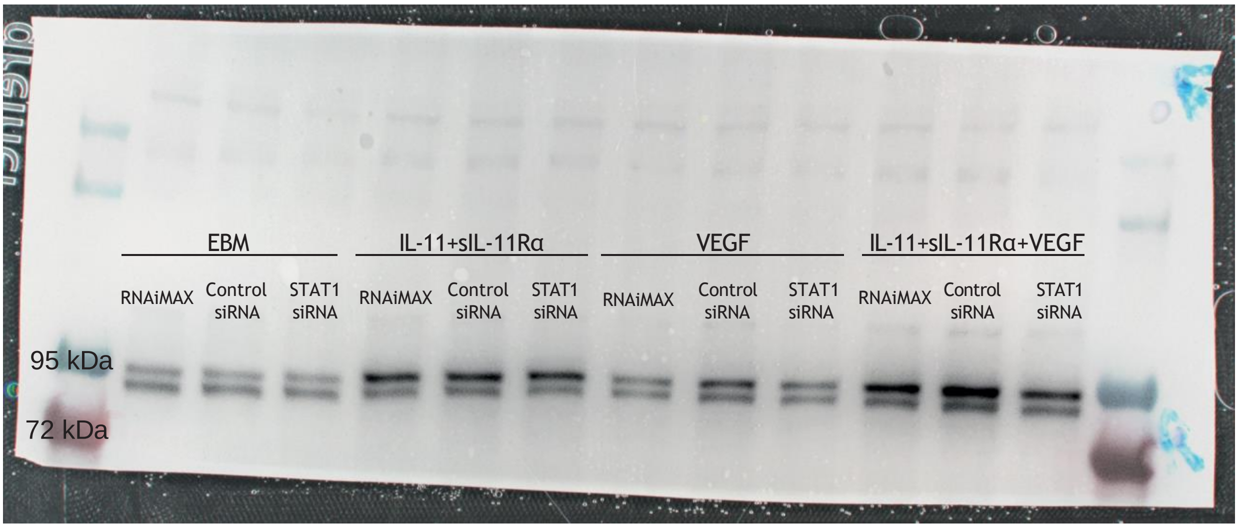

pERK
44, 42 kDa

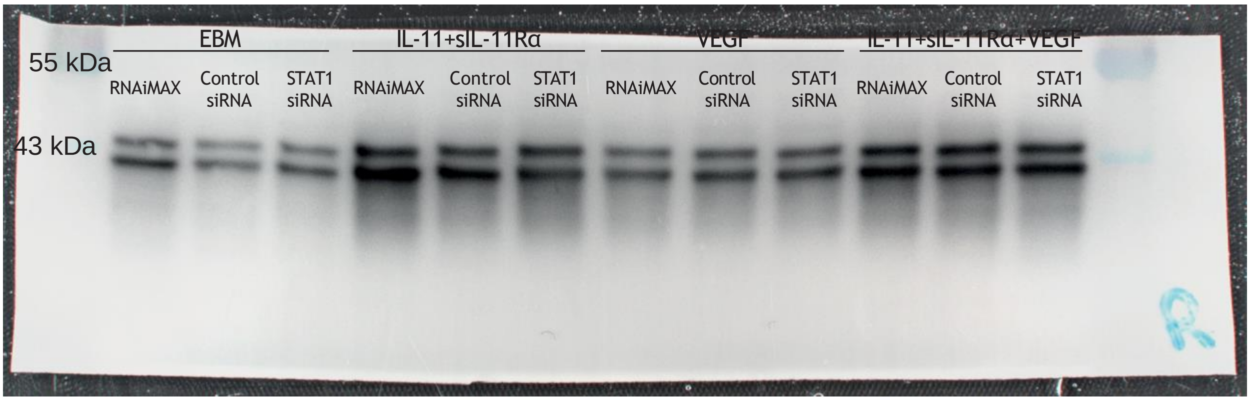

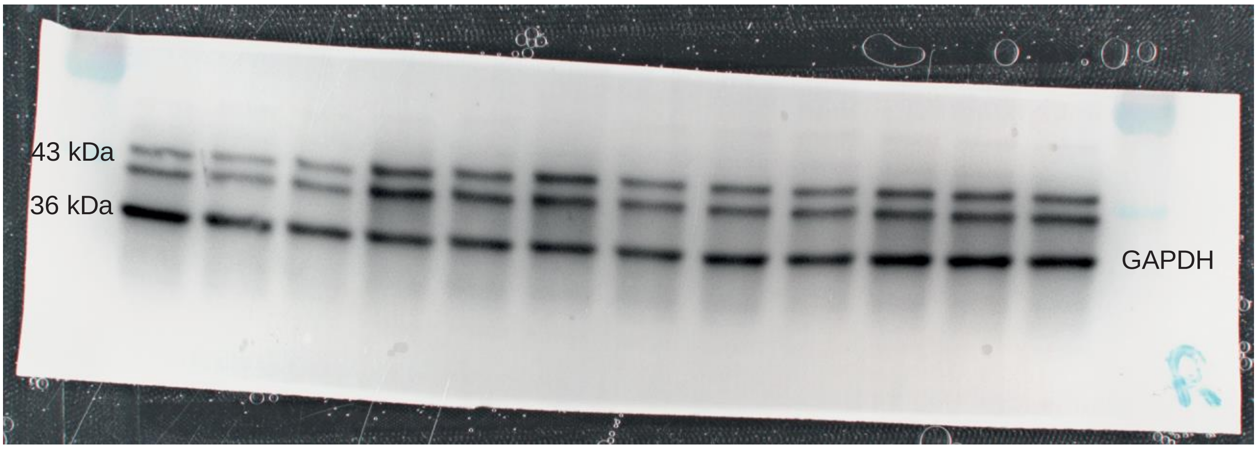

Full unedited blot for Figure 6D #3

pSTAT5

90 kDa

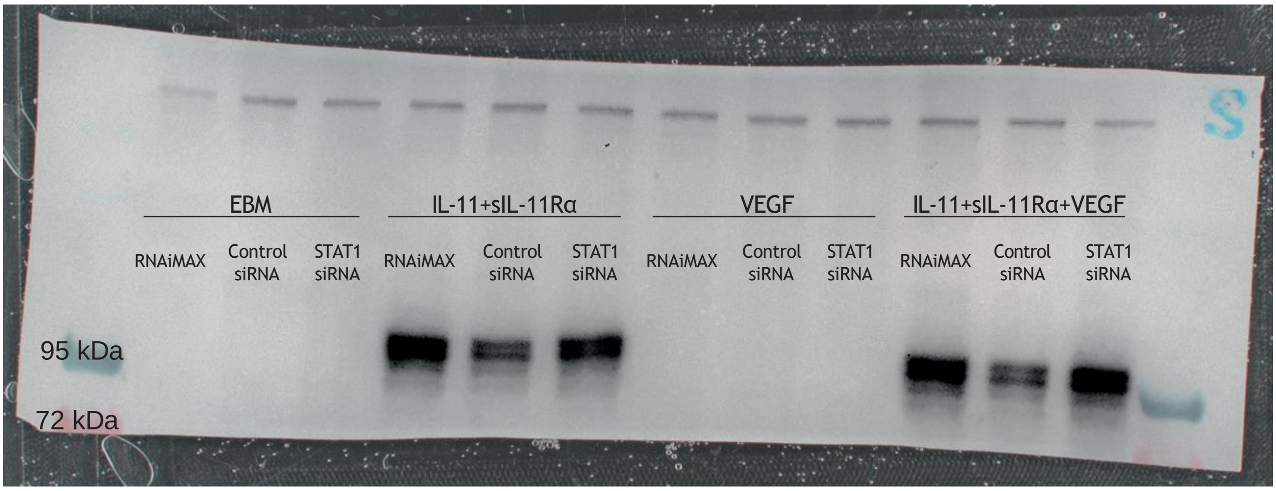

pAkt

60 kDa

EBM			IL-11+sIL-11Rα			VEGF			IL-11+sIL-11Rα+VEGF		
RNAiMAX	Control siRNA	STAT1 siRNA	RNAiMAX	Control siRNA	STAT1 siRNA	RNAiMAX	Control siRNA	STAT1 siRNA	RNAiMAX	Control siRNA	STAT1 siRNA

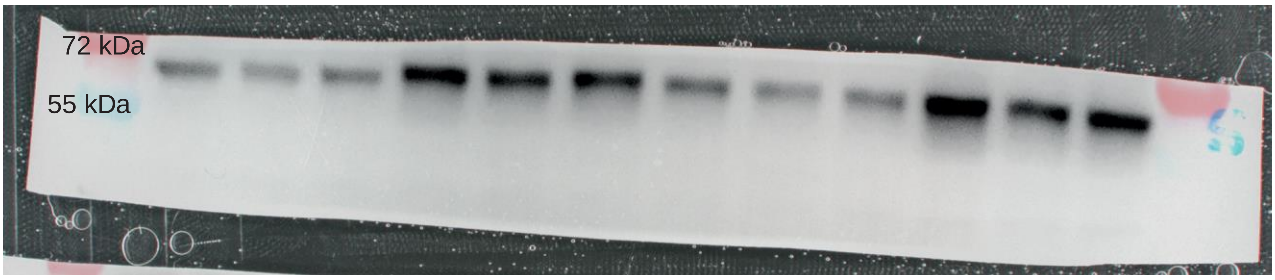

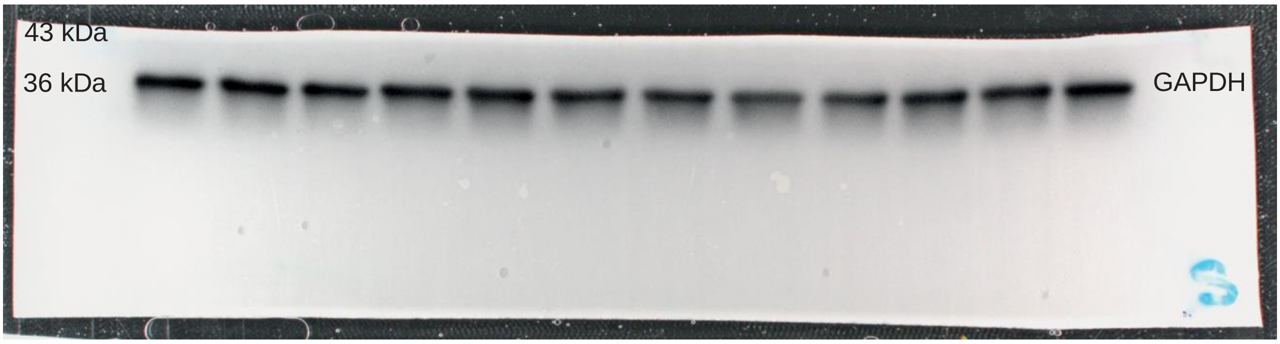

Full unedited blot for Figure 7C

pSTAT3 Tyr

79, 86 kDa

pSTAT3 Tyr

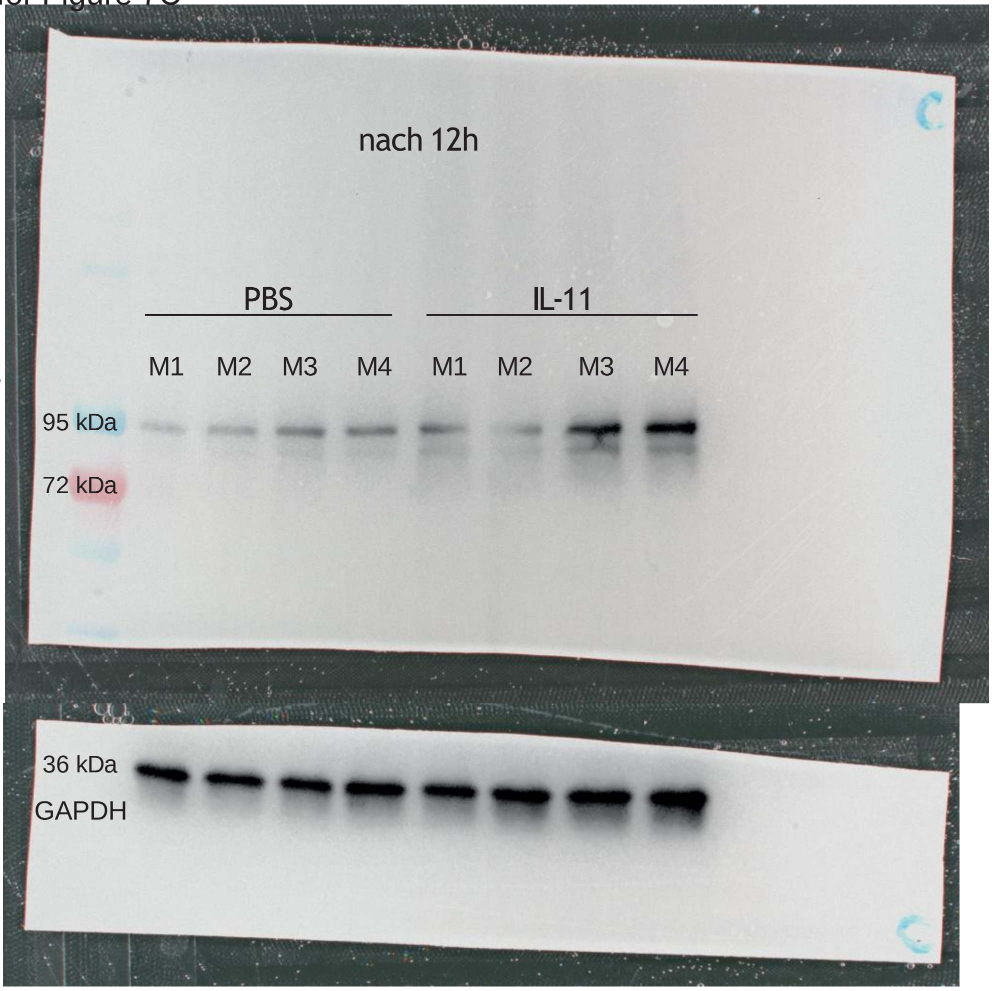

pSTAT1

84, 91 kDa

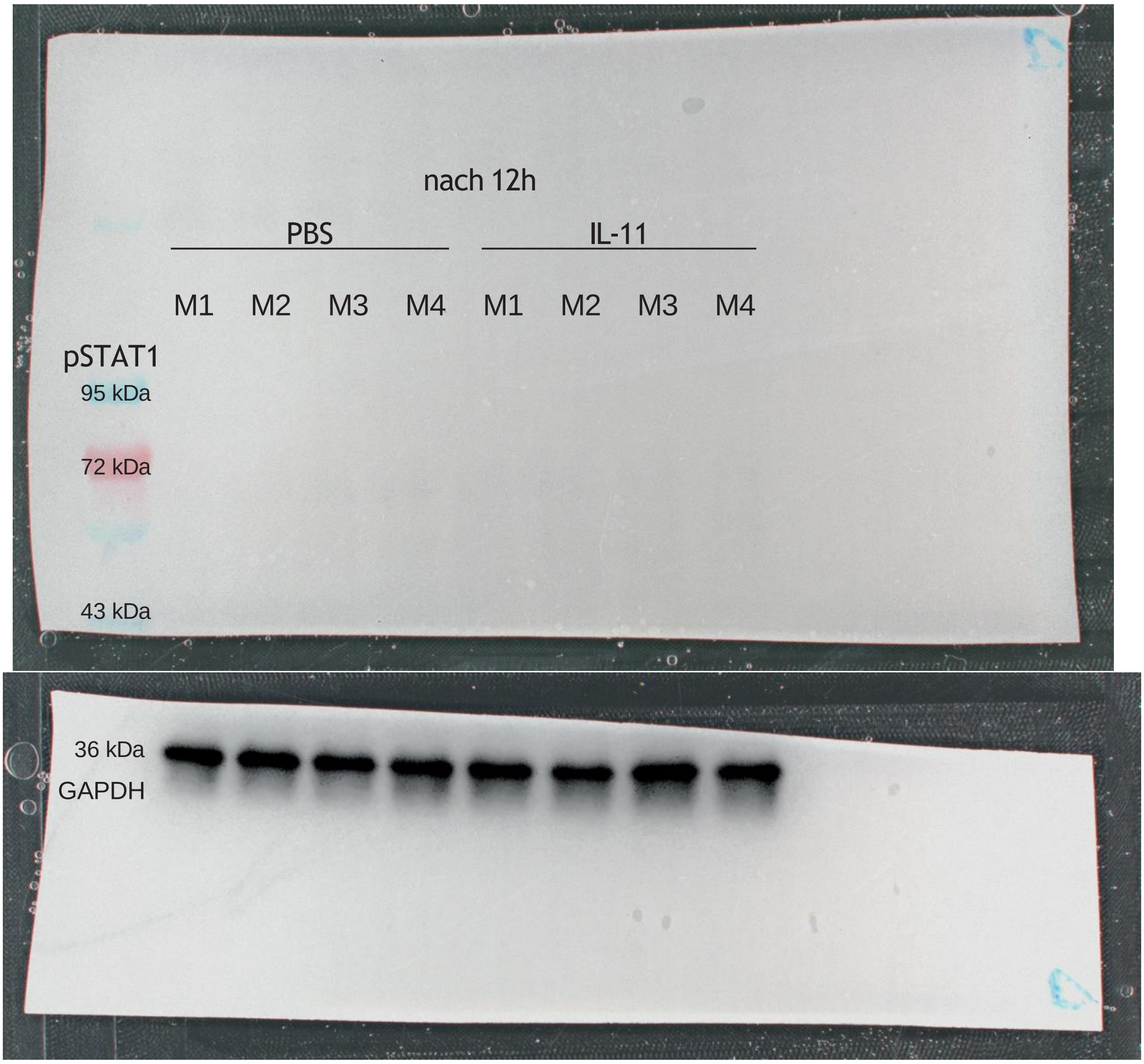

Full unedited blot for Figure 7C

pAkt

60 kDa

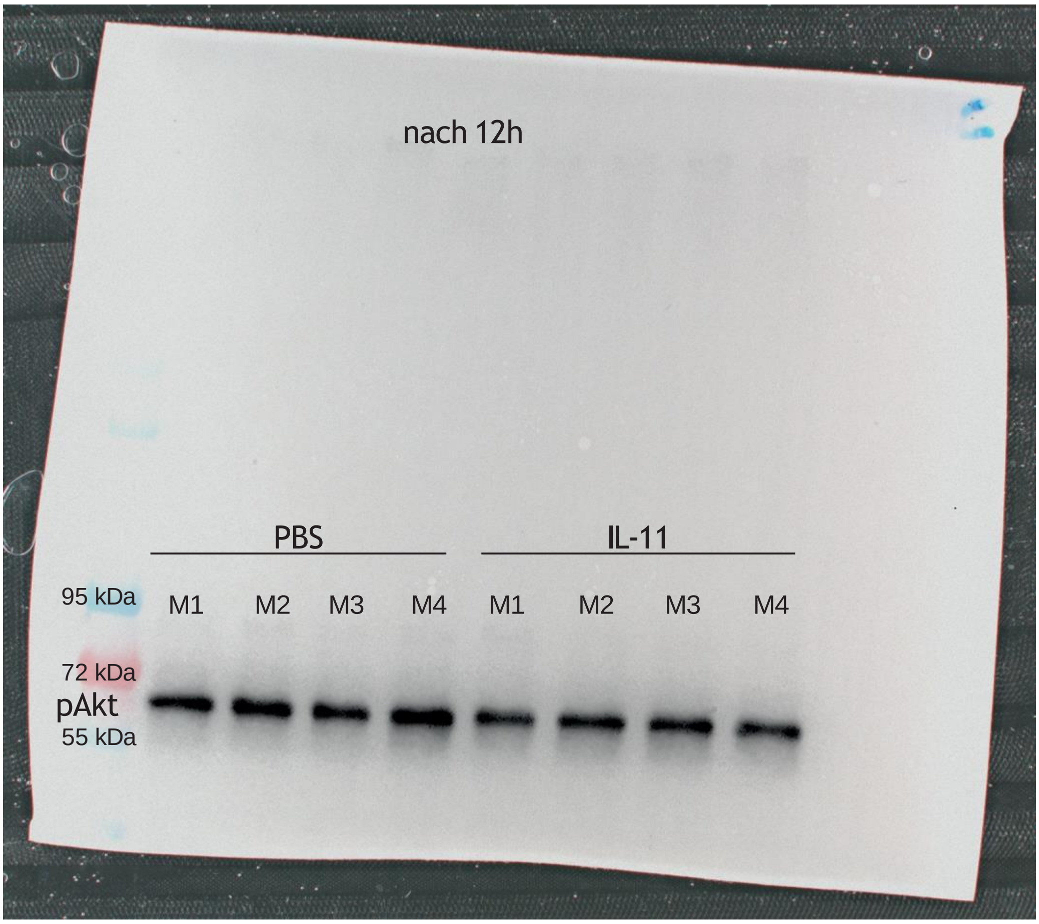

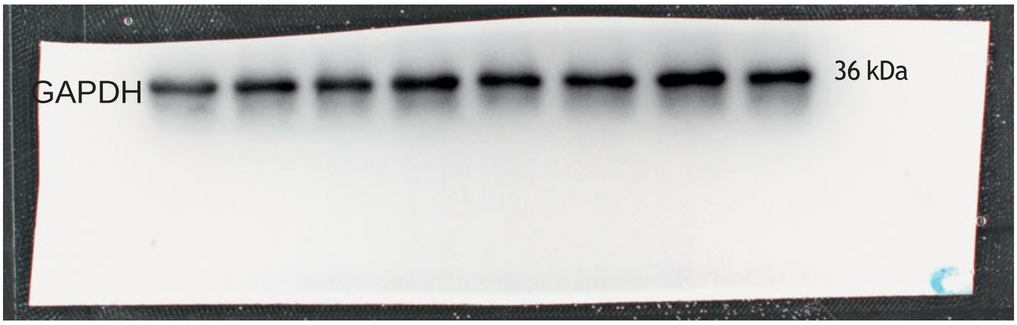

Full unedited blot for Figure 7C

pERK 44, 42 kDa

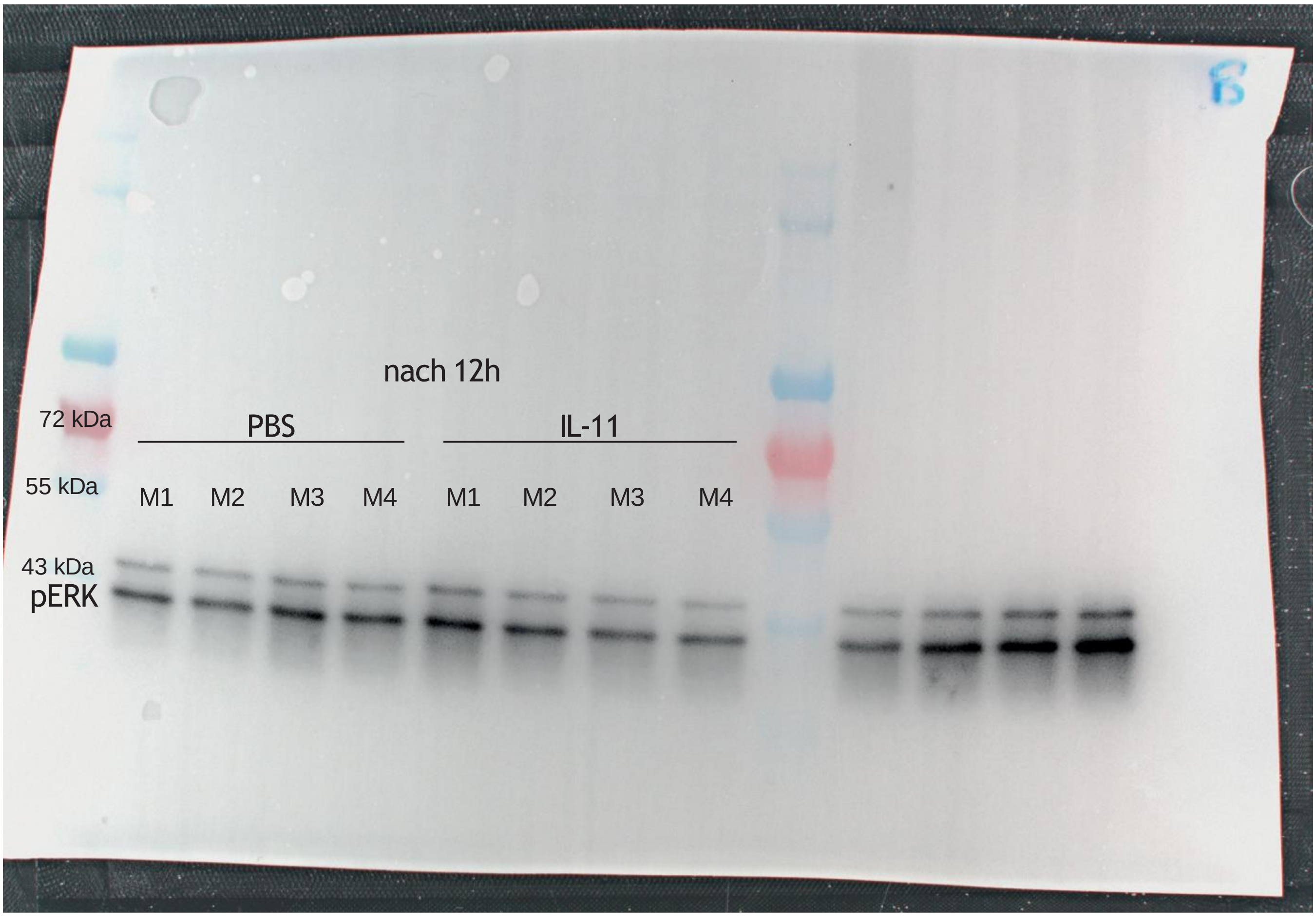

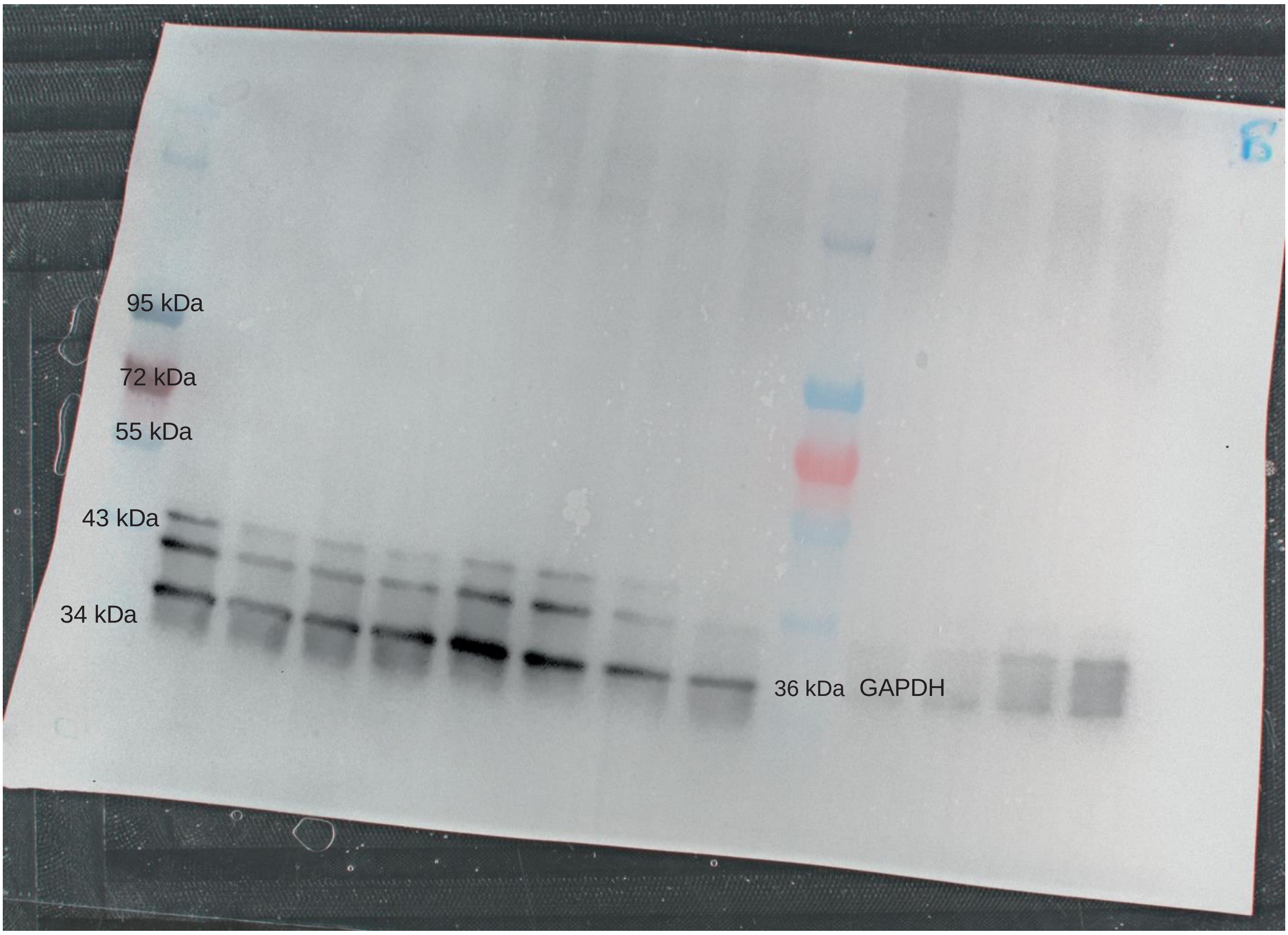

Full unedited blot for Figure 7C

pSTAT3 Ser
86 kDa

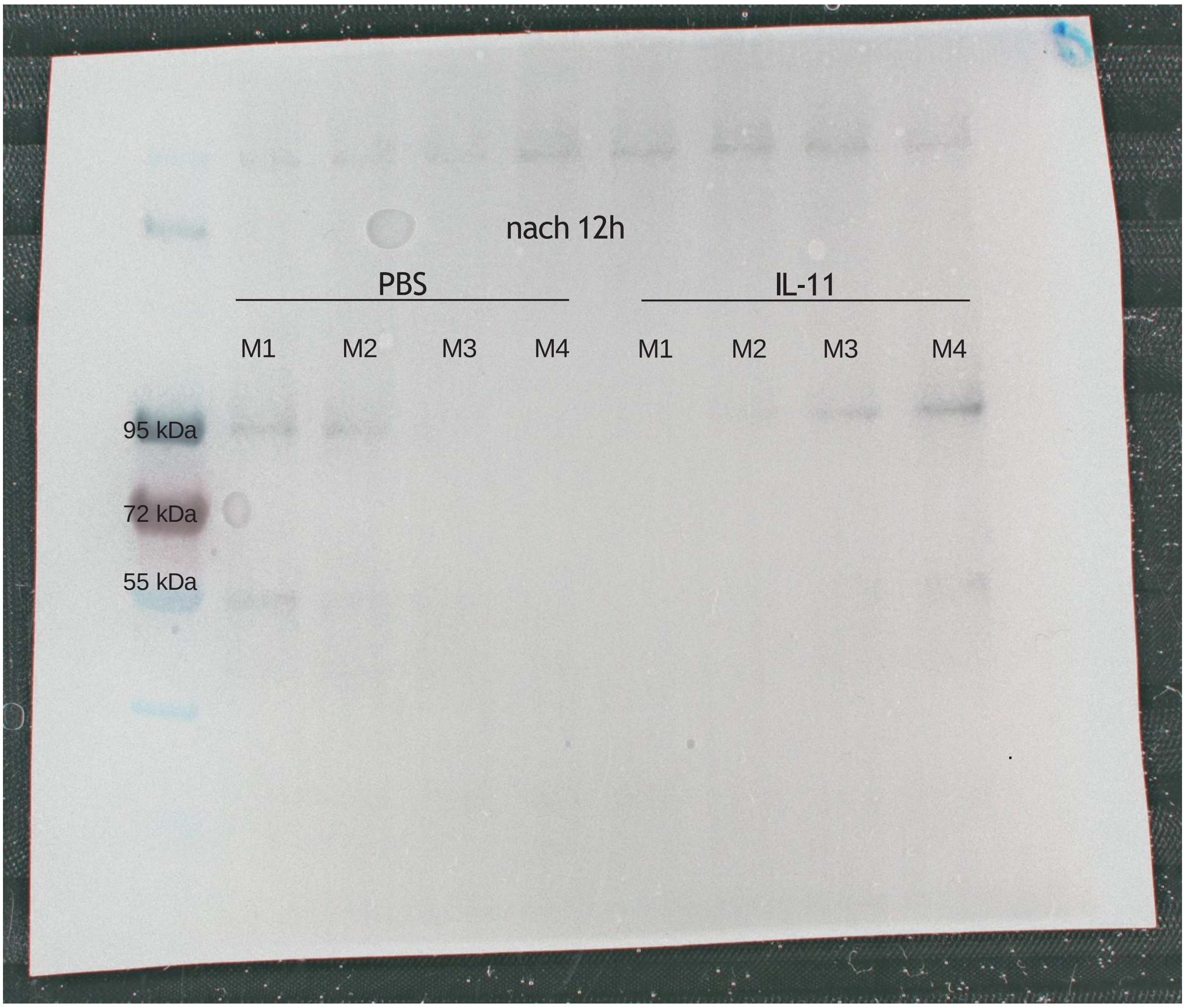

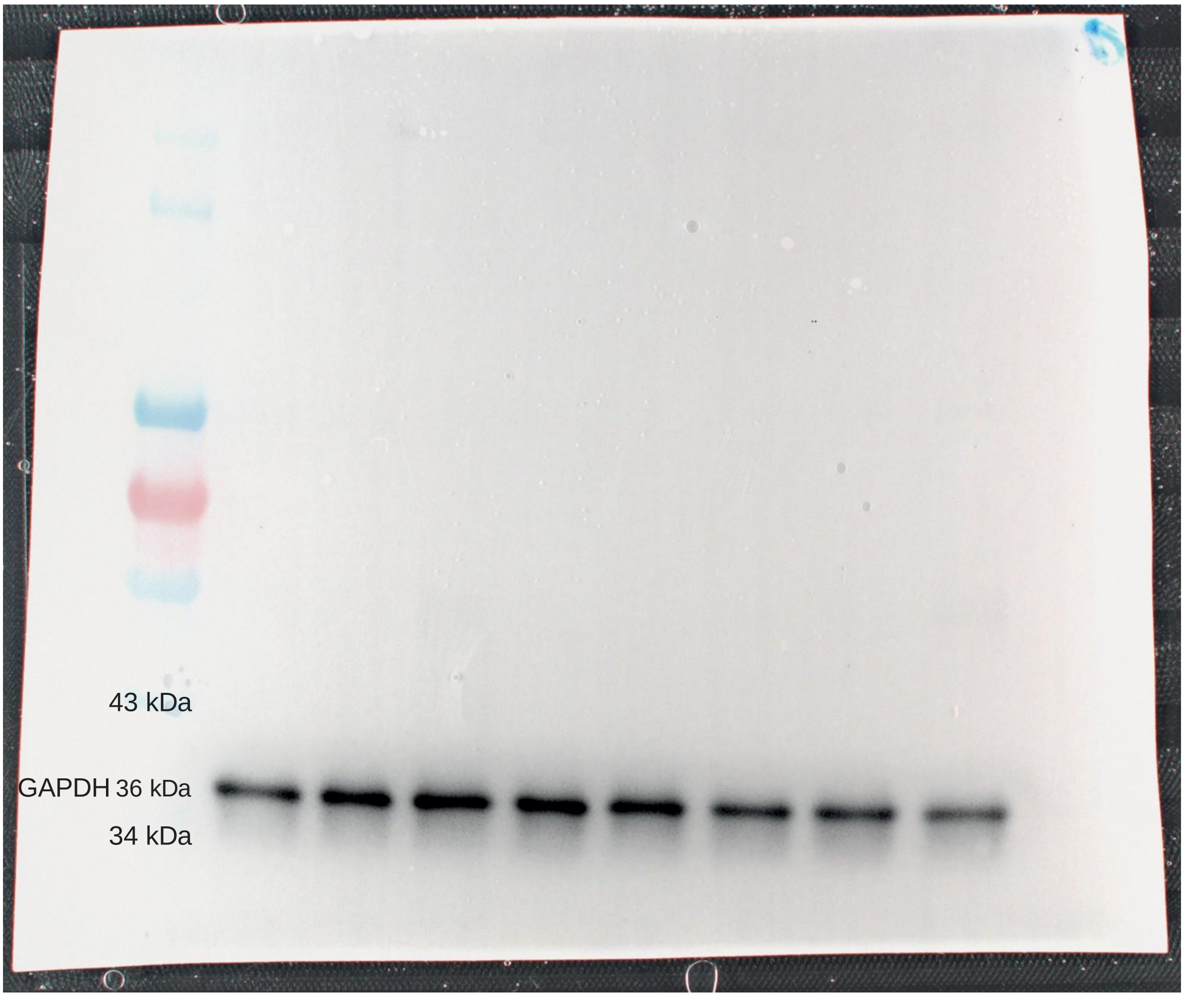

Full unedited blot for Figure 7C

pSTAT5

90 kDa

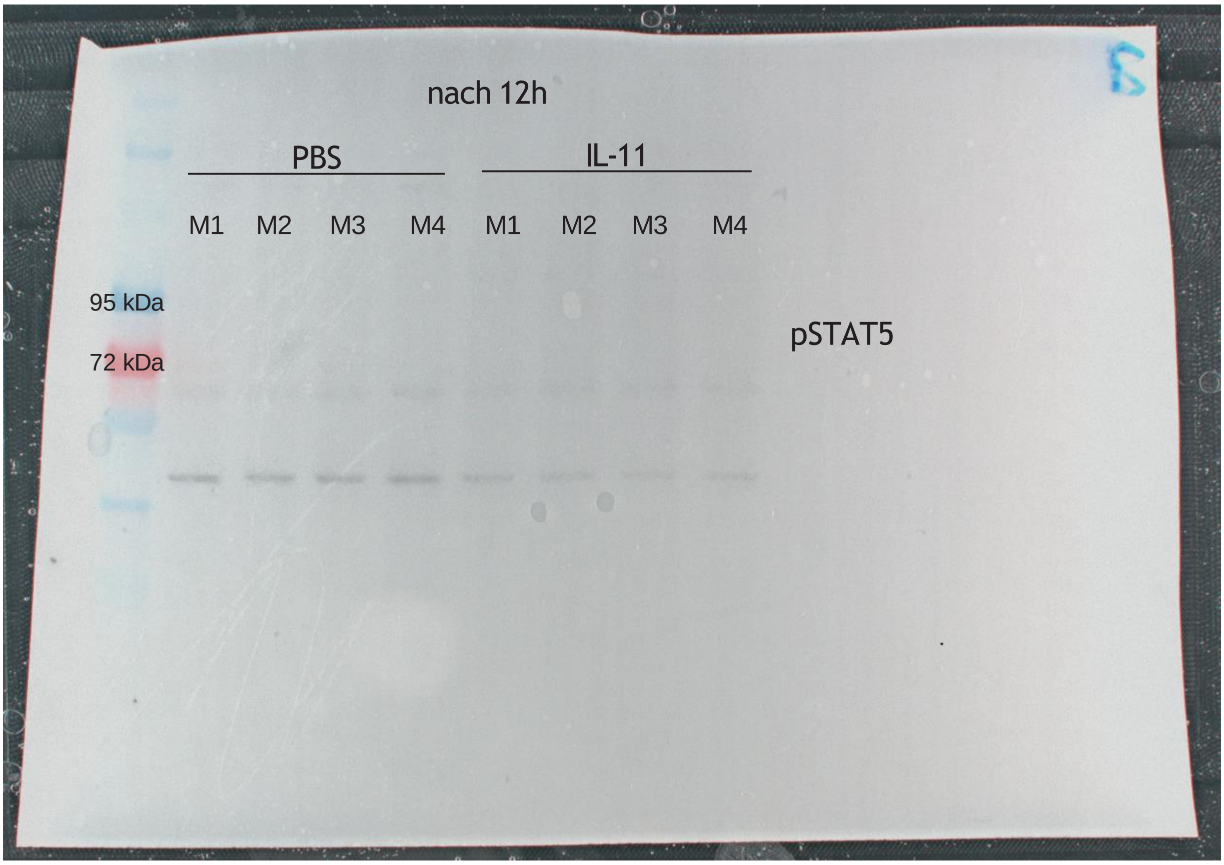

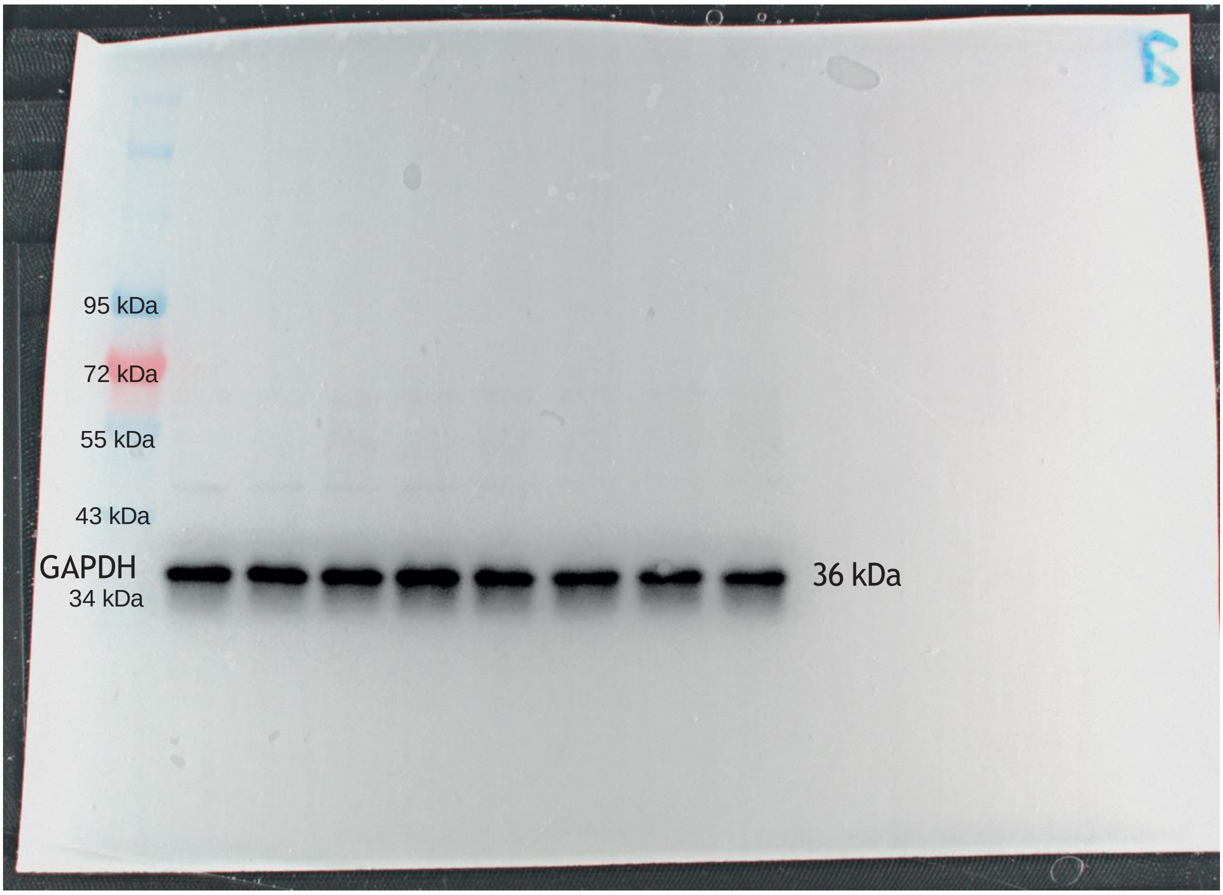

Full unedited blot for Figure 7D

pSTAT3 Tyr

79/ 86 kDa

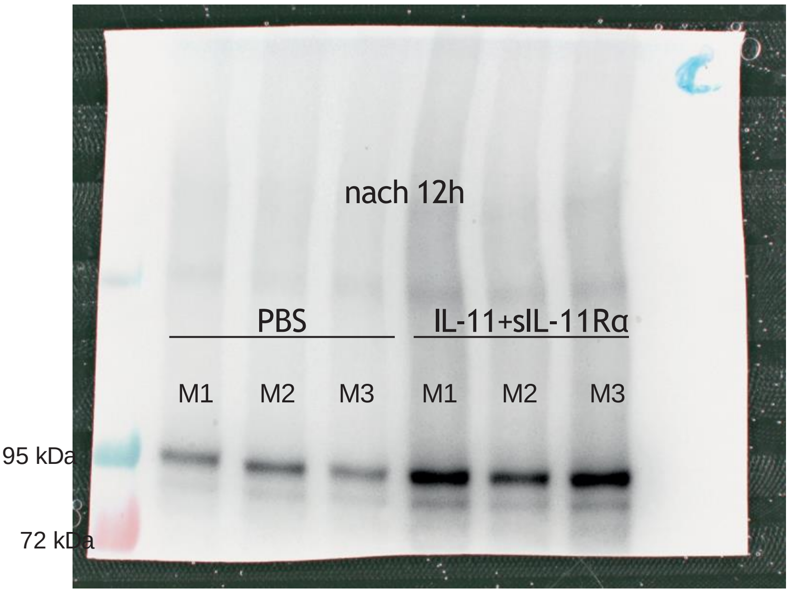

pERK

44/ 42 kDa

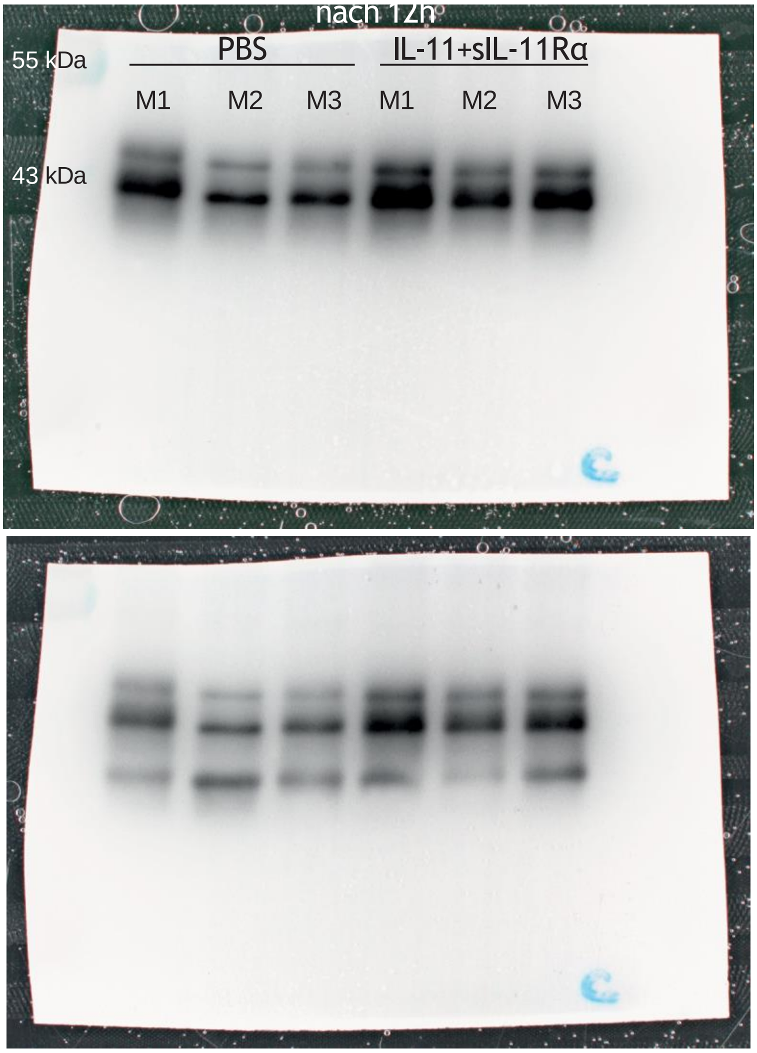

Full unedited blot for Figure 7D

pSTAT3 Ser

86 kDa

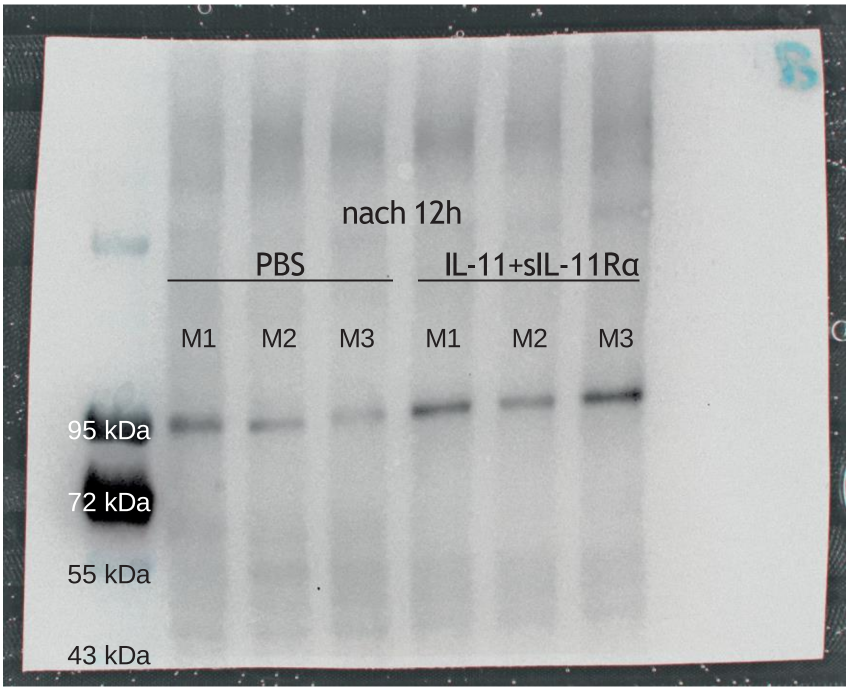

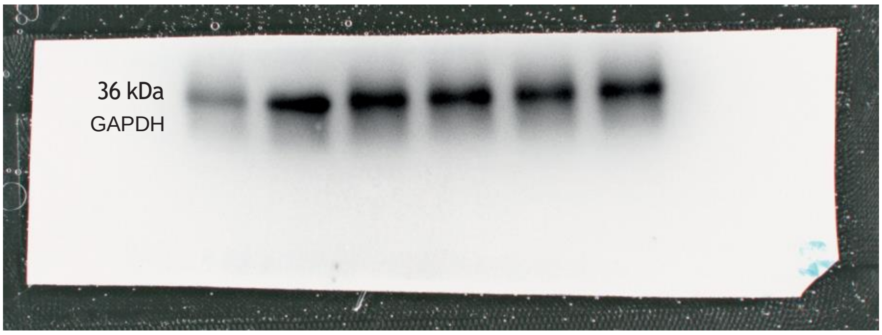

pSTAT5

90 kDa

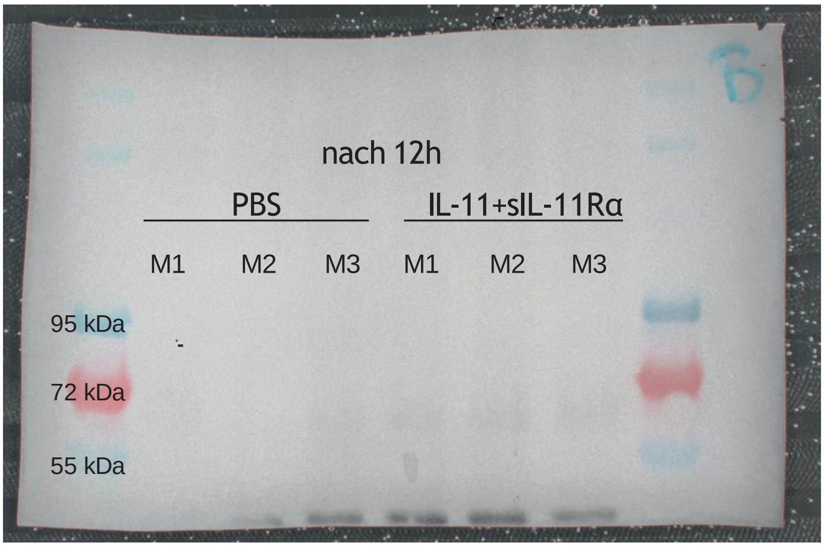

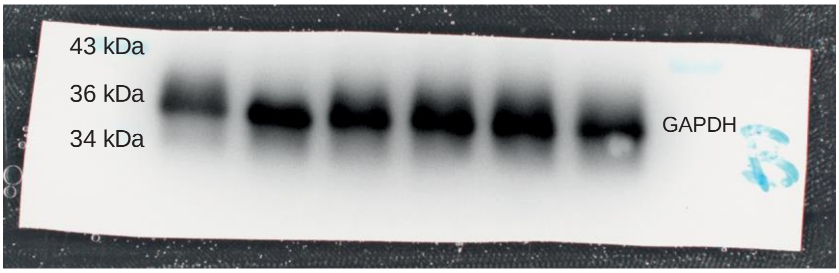

Full unedited blot for Figure 7D

pAkt
60 kDa

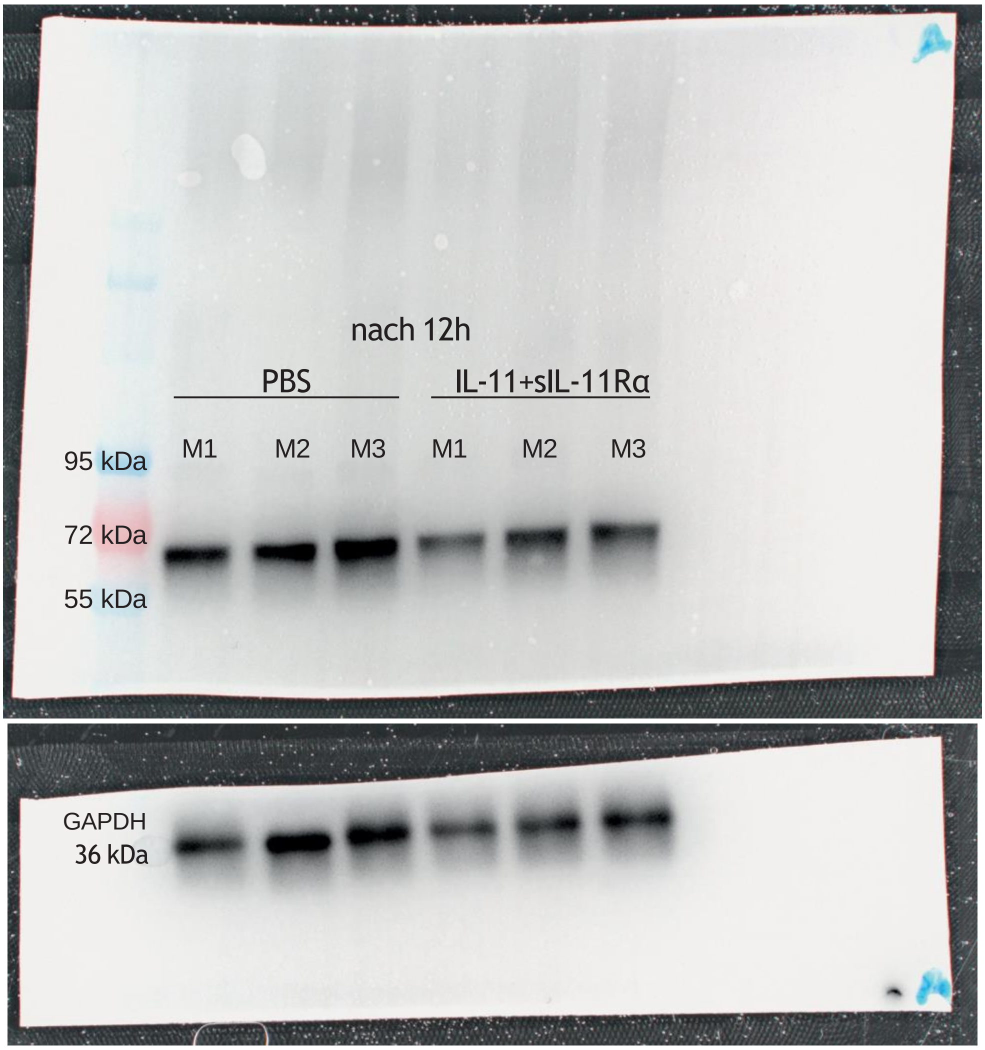

pSTAT1
84, 91 kDa

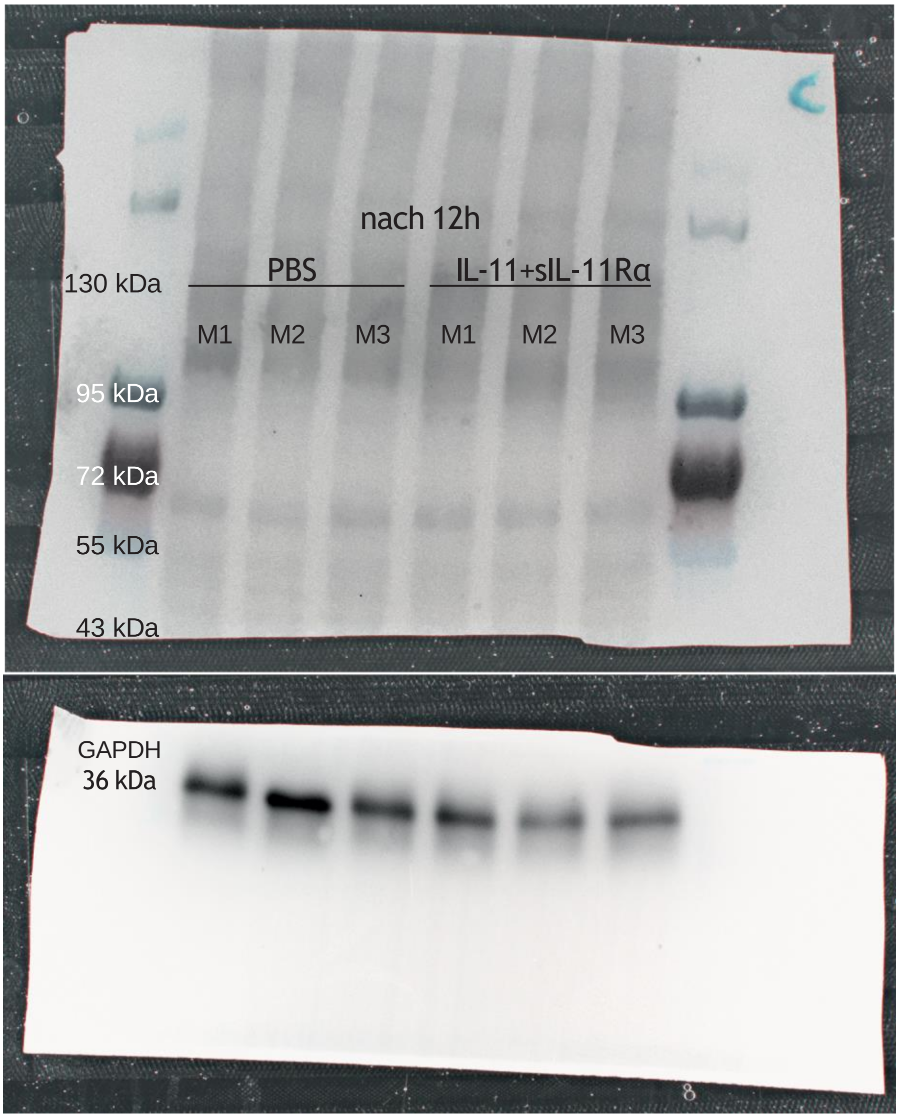

Full unedited blot for Figure 9A + Suppl. Fig. S7 #1

pAkt

60 kDa

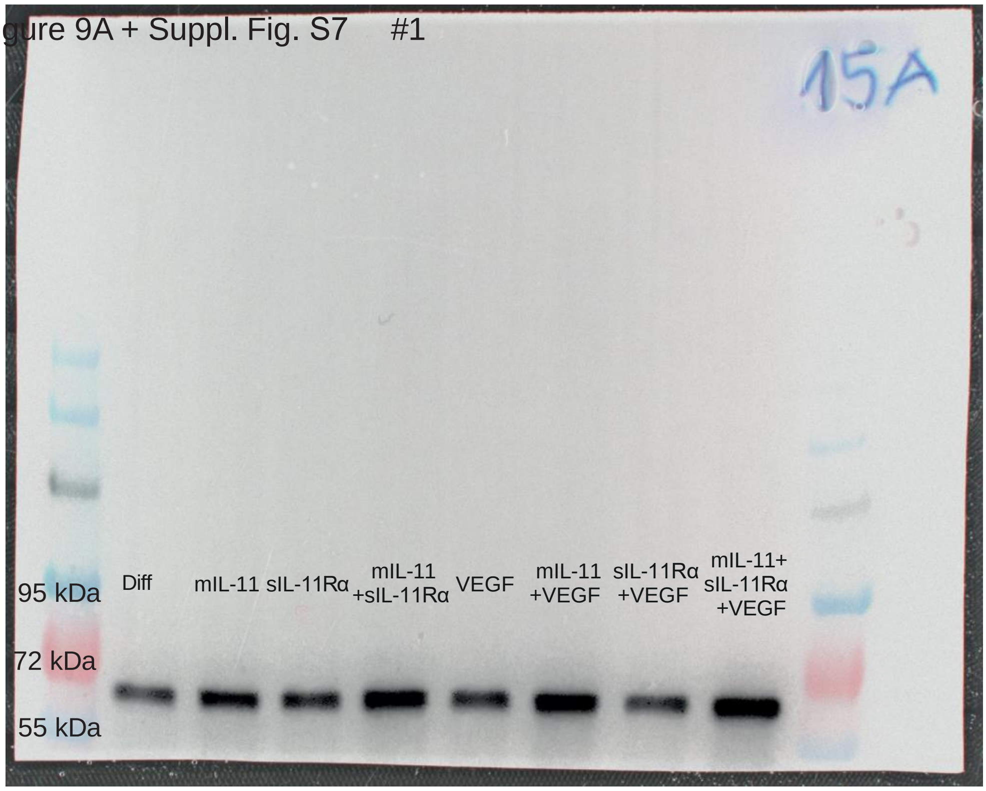

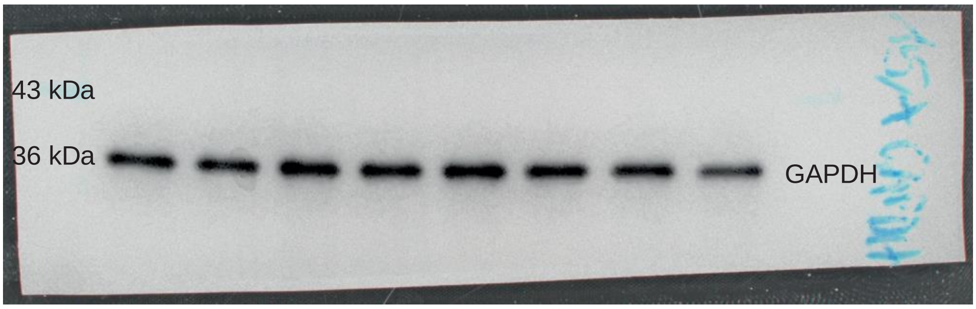

pSTAT3 Tyr

79, 86 kDa

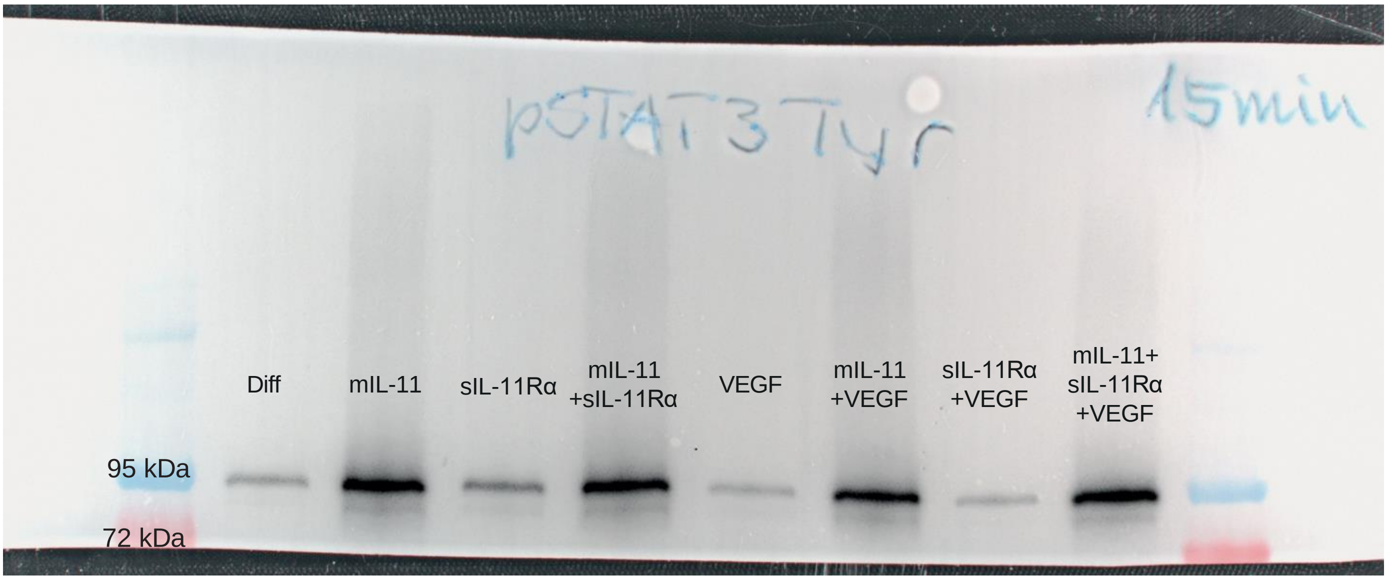

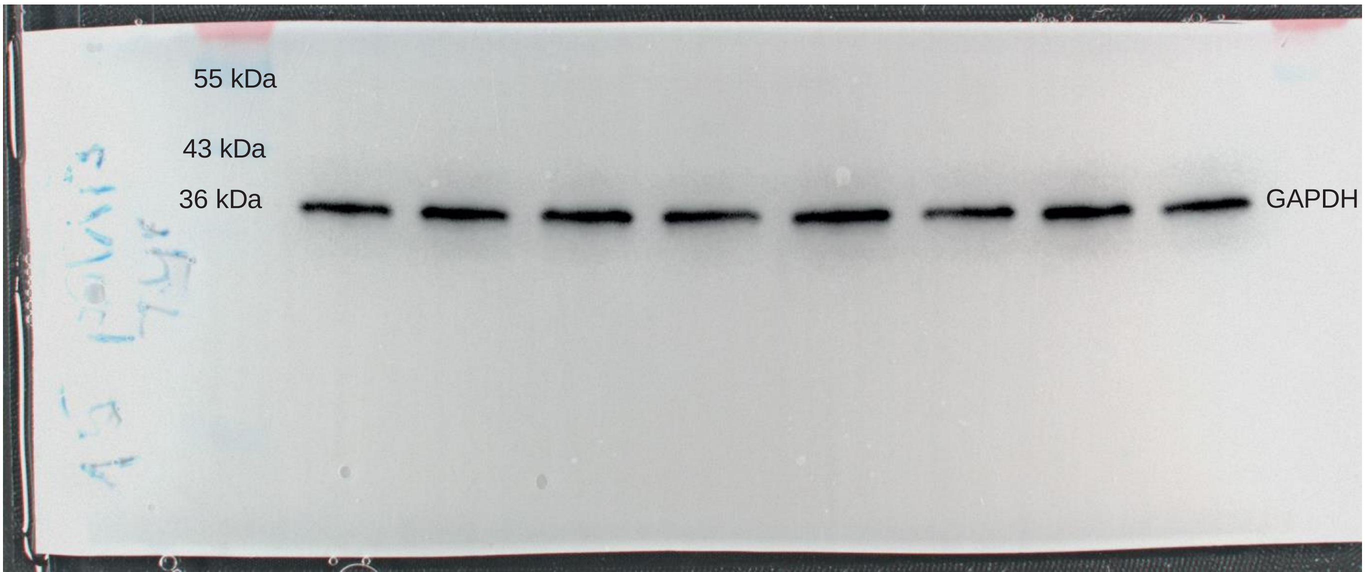

Full unedited blot for Figure 9A + Suppl. Fig. S7 #1

pSTAT5
90 kDa

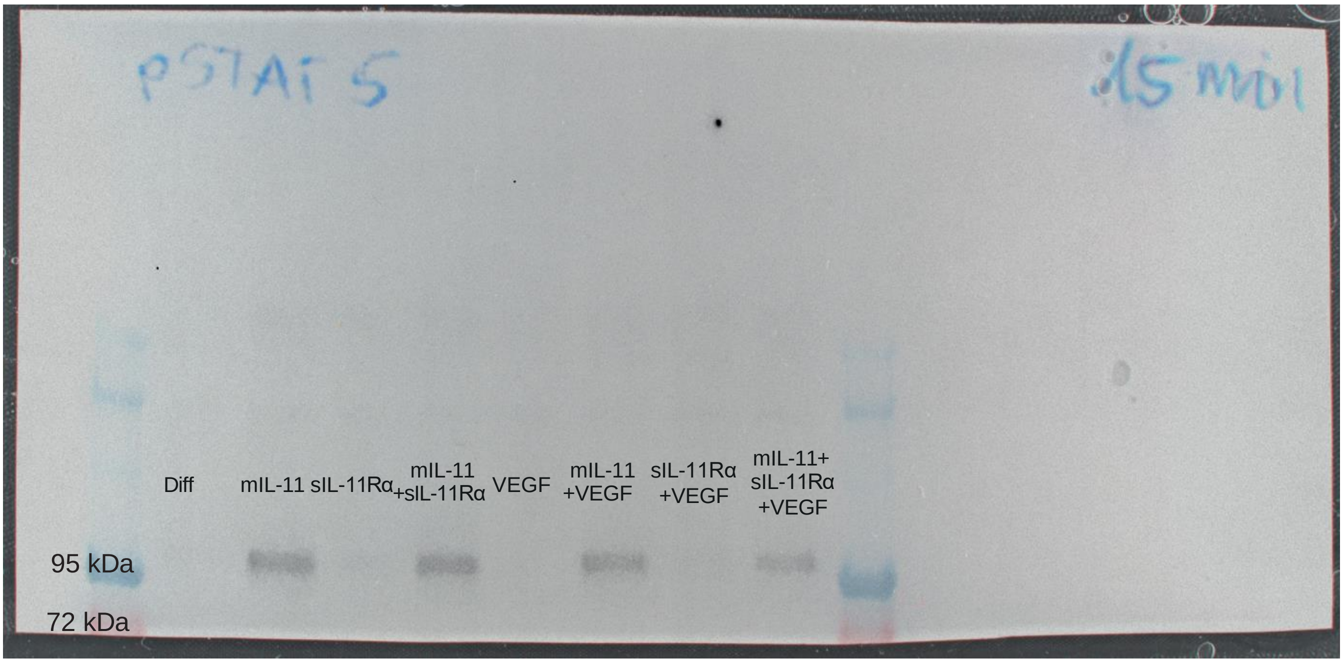

pERK
44, 42 kDa

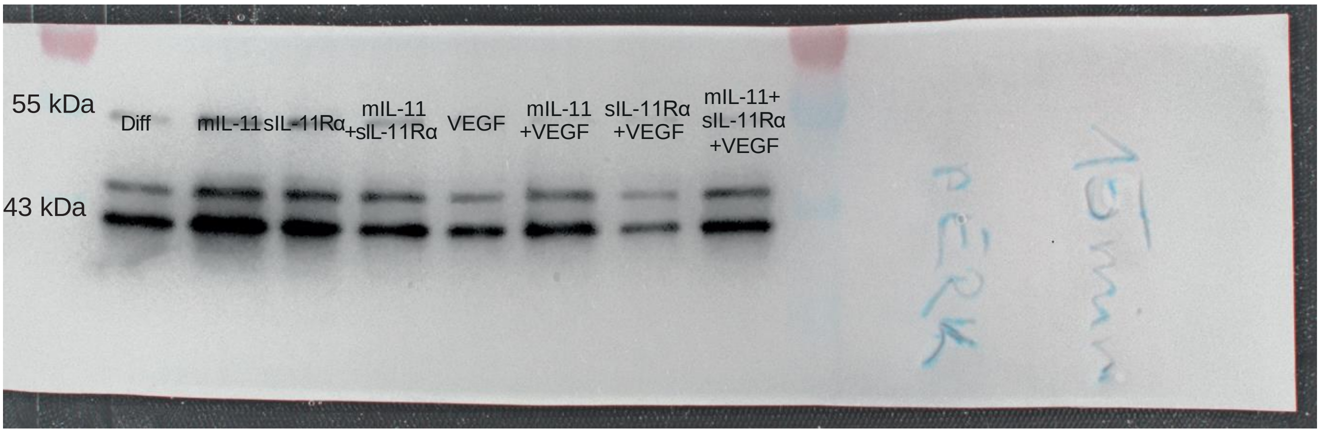

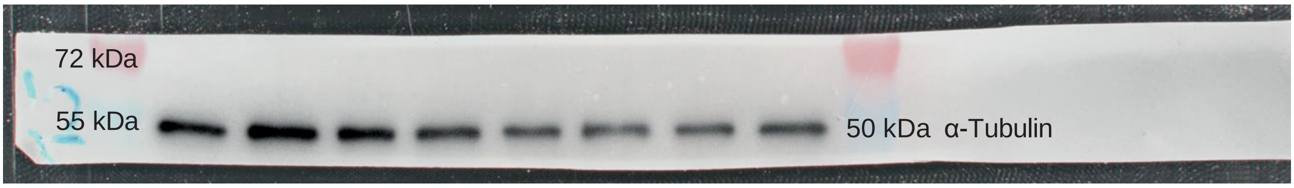

Full unedited blot for Figure 9A + Suppl. Fig. S7 #1

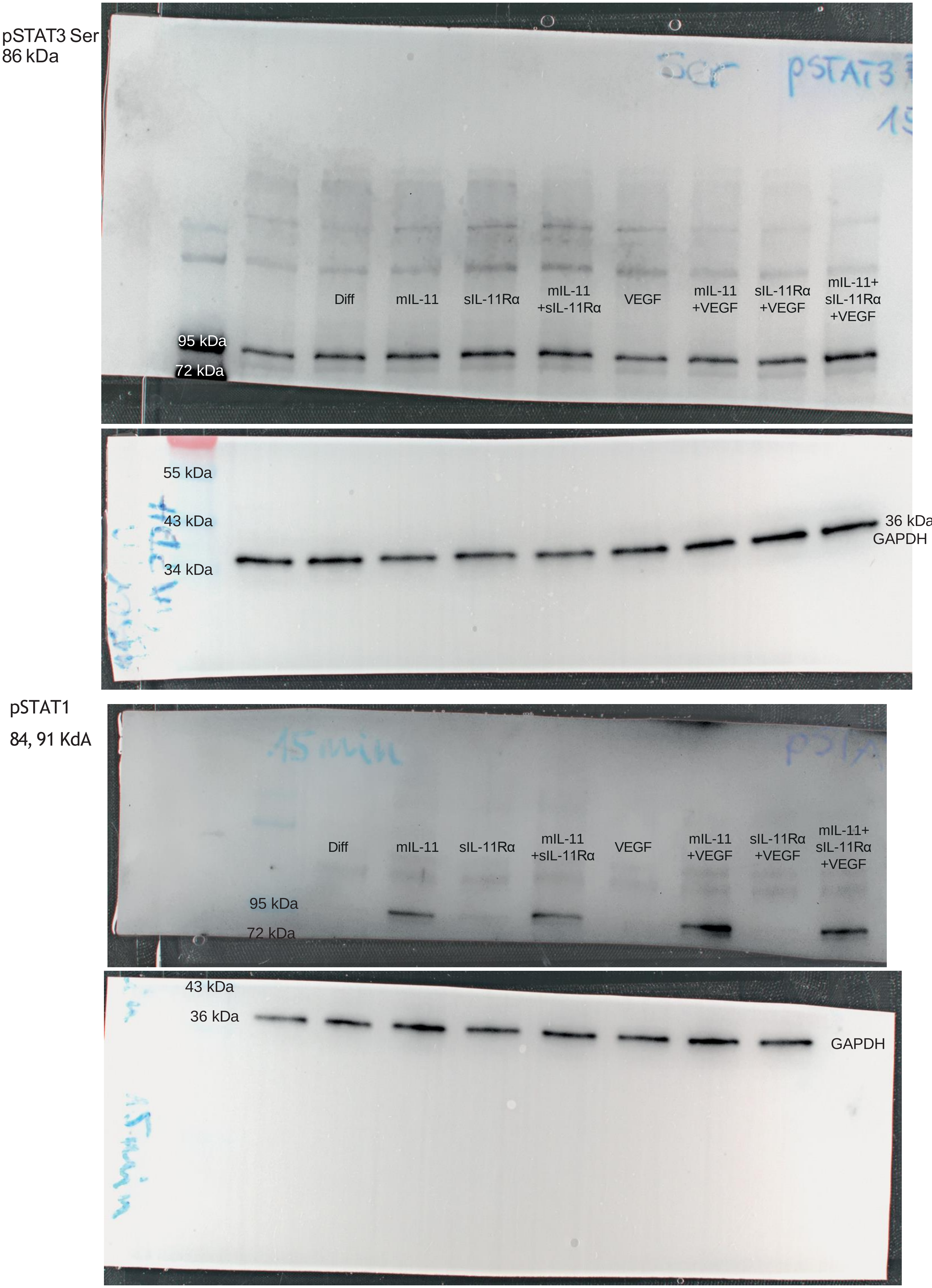

Full unedited blot for Figure 9A + Suppl. Fig. S7 #2

pSTAT5
90 kDa

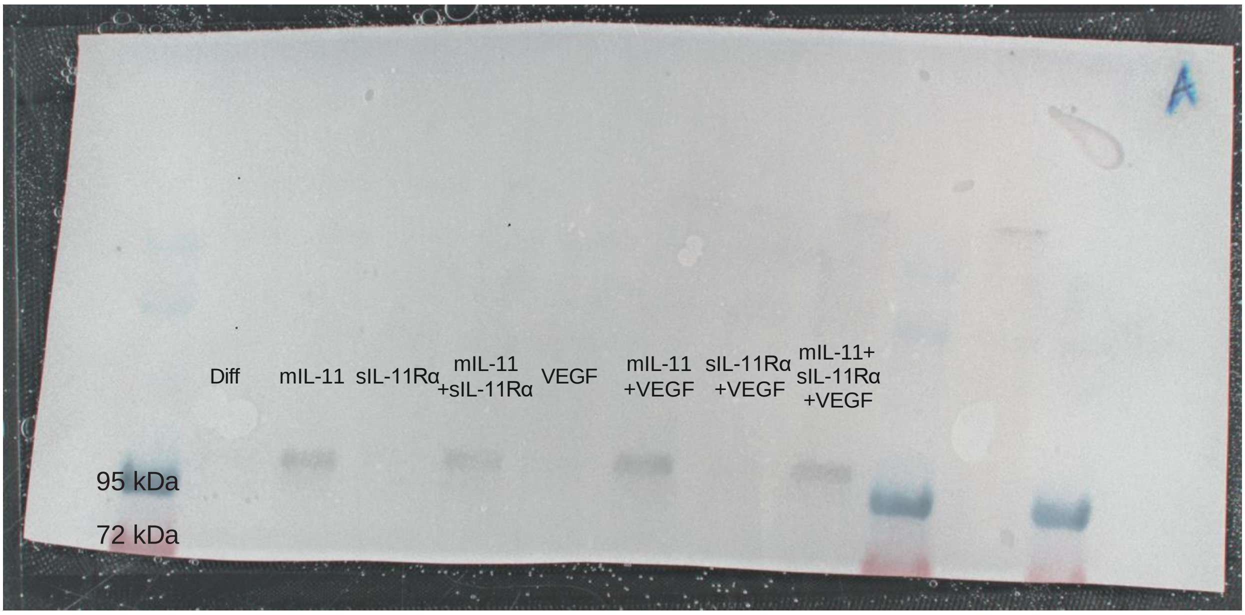

pAkt
60 kDa

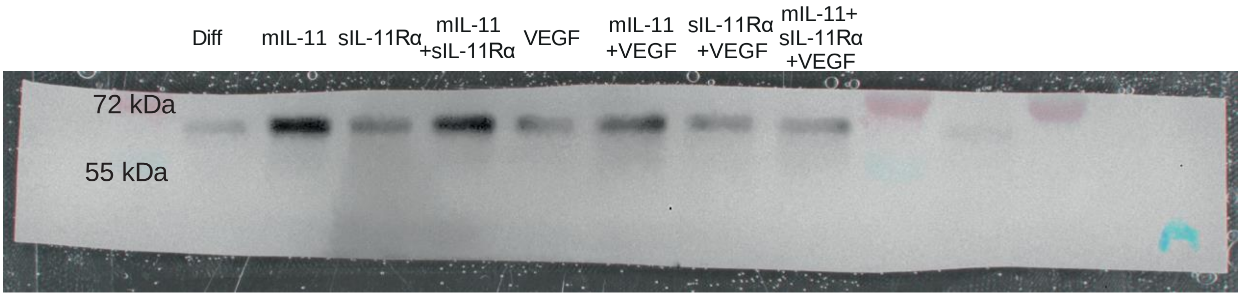

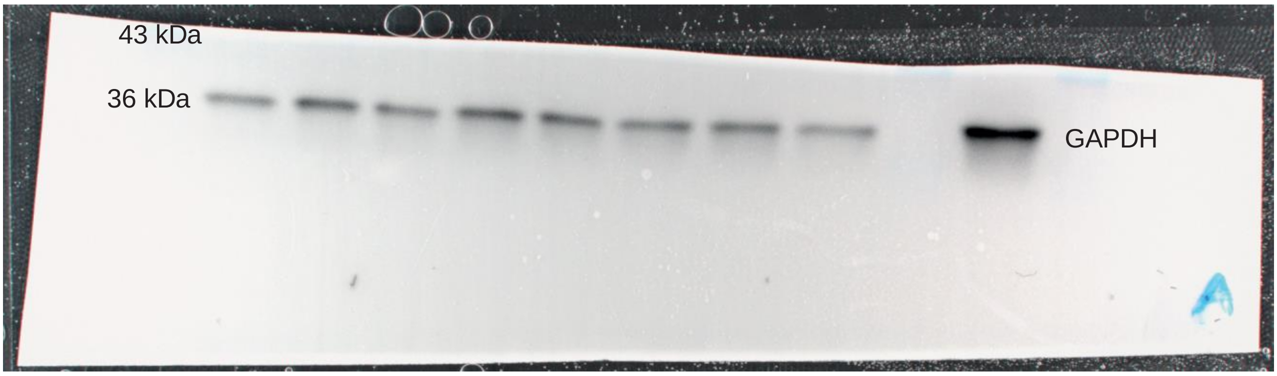

Full unedited blot for Figure 9A + Suppl. Fig. S7 #2

pSTAT1
84, 91 kDa

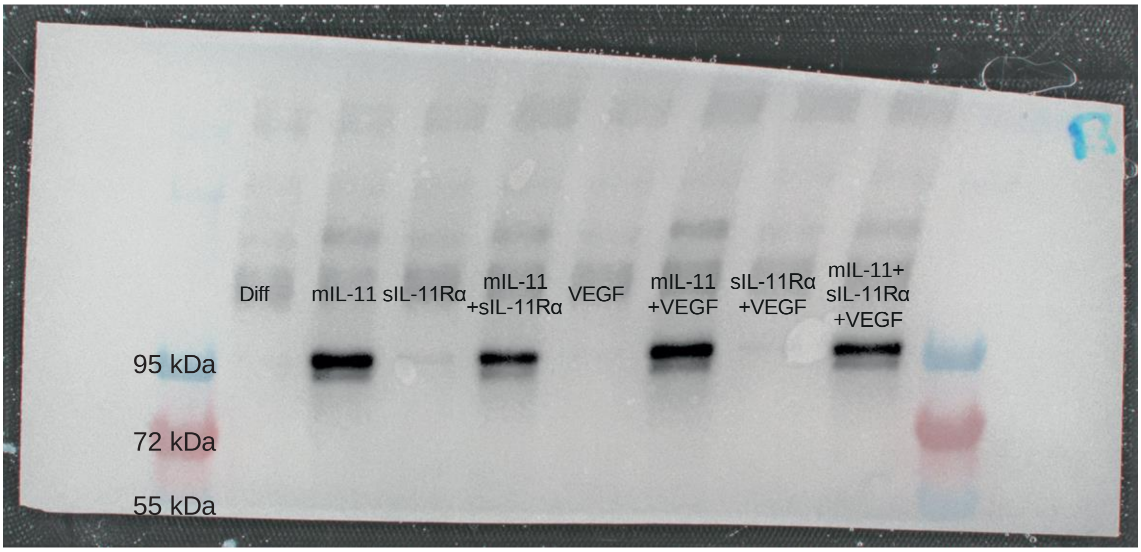

pERK
44, 42 kDa

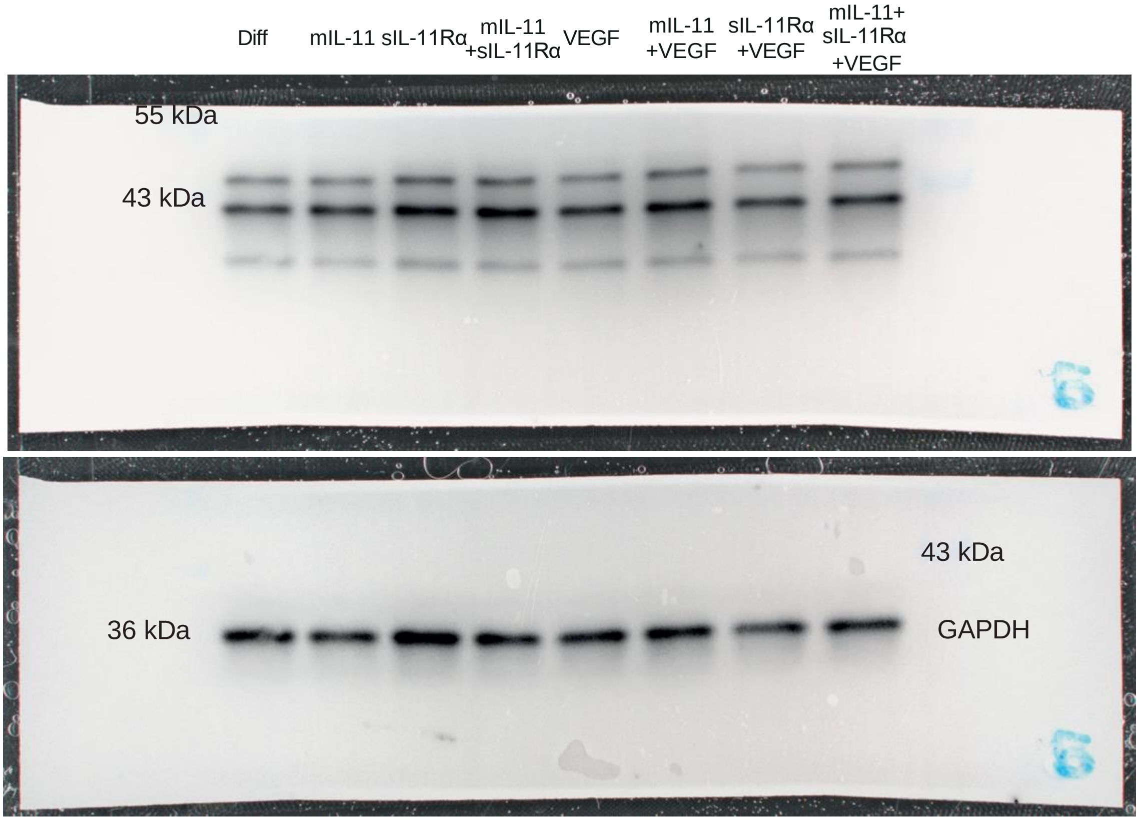

Full unedited blot for Figure 9A + Suppl. Fig. S7 #2

pSTAT3 Tyr
79, 86 kDa

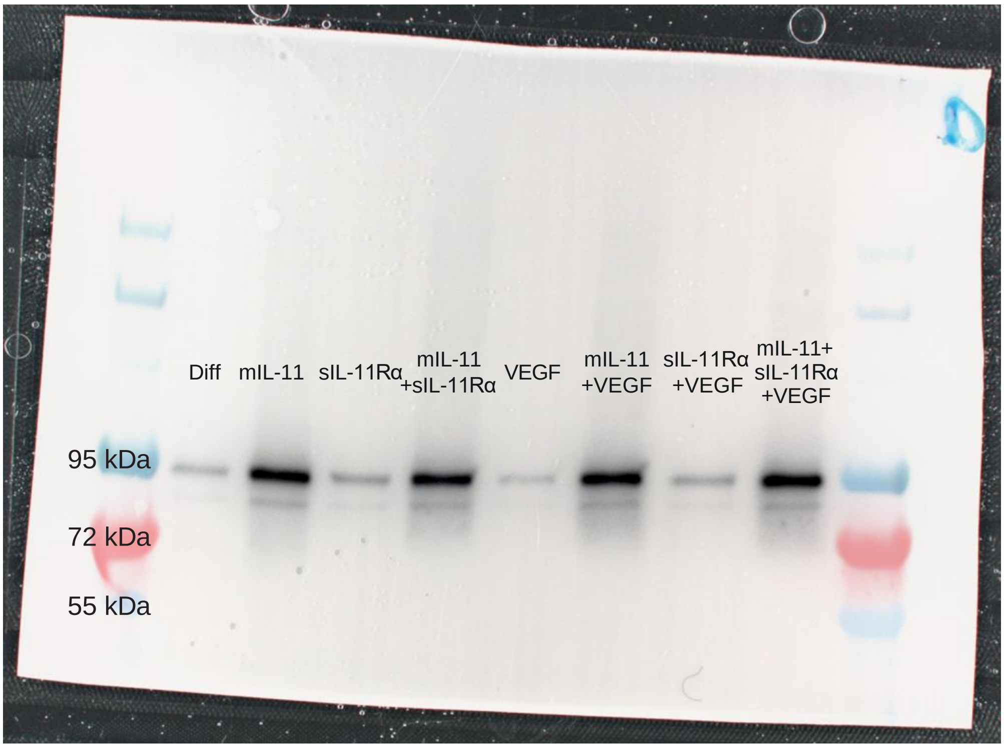

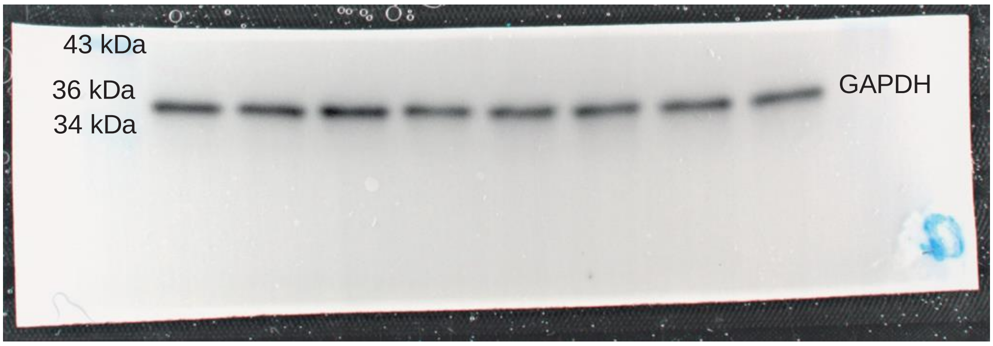

pSTAT3 Ser
86 kDa

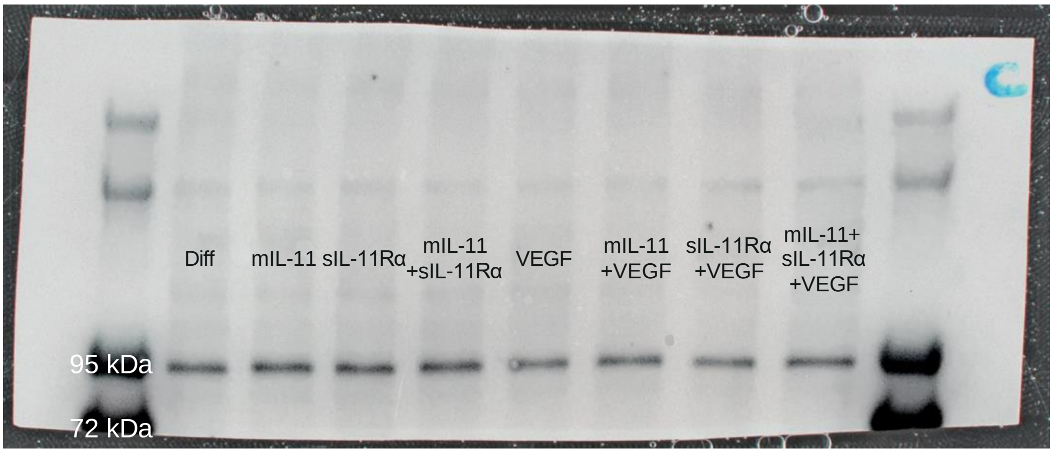

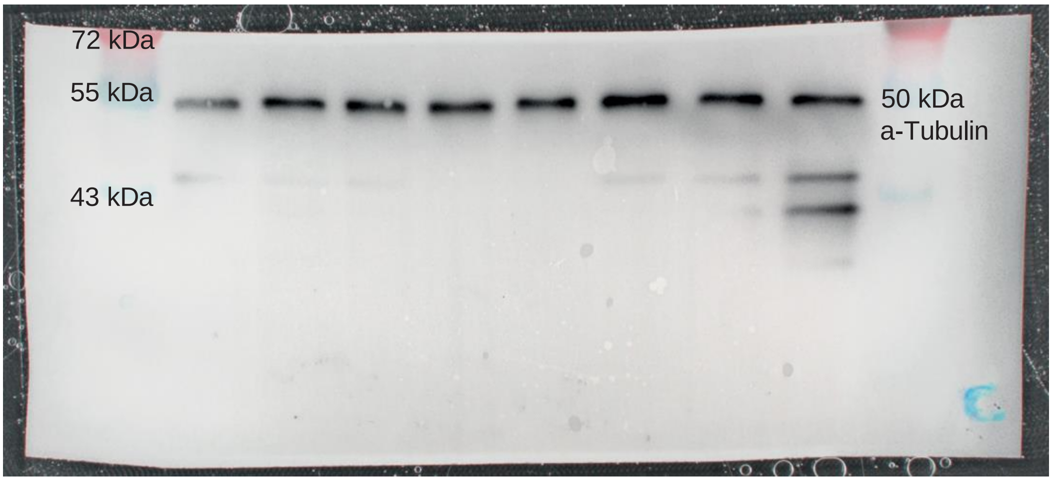

Full unedited blot for Fig. 9A #3

pERK
44, 42 kDa

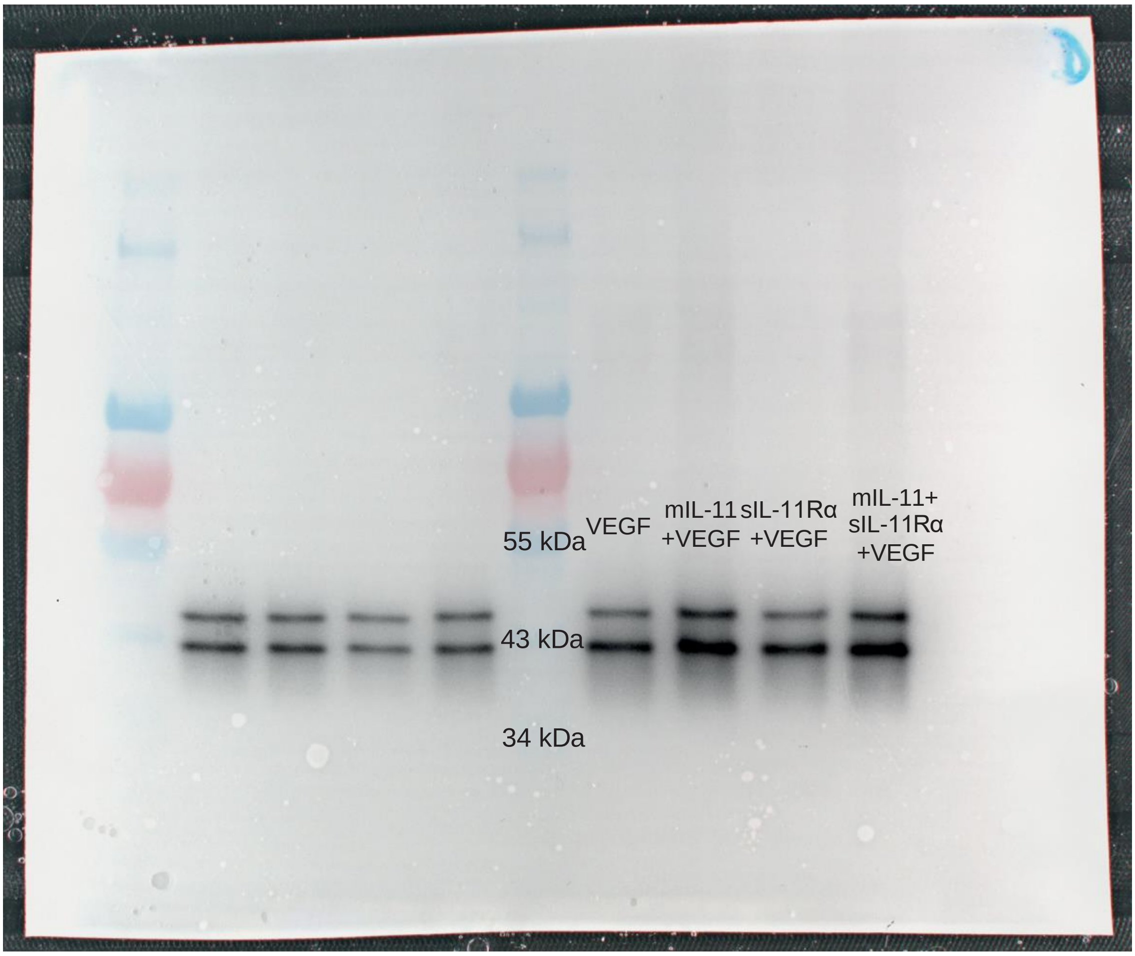

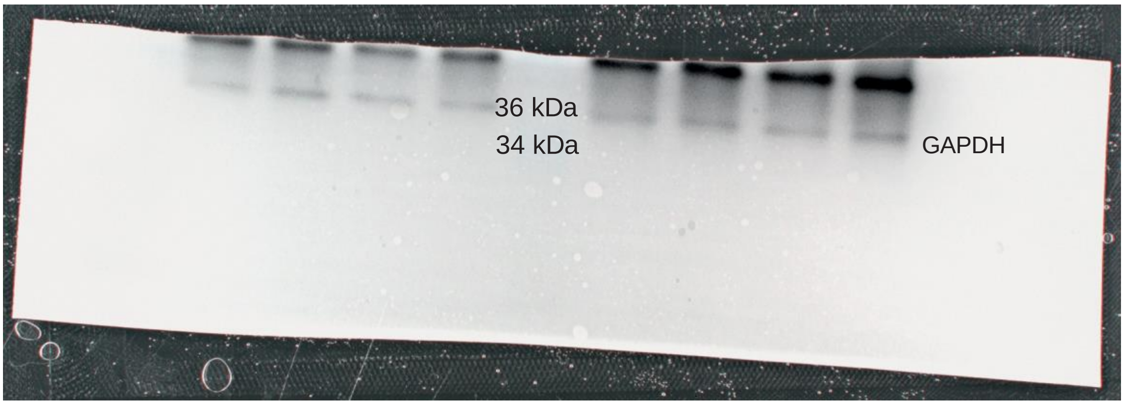

Full unedited blot for Fig. 9A #3

pSTAT3 Ser
86 kDa

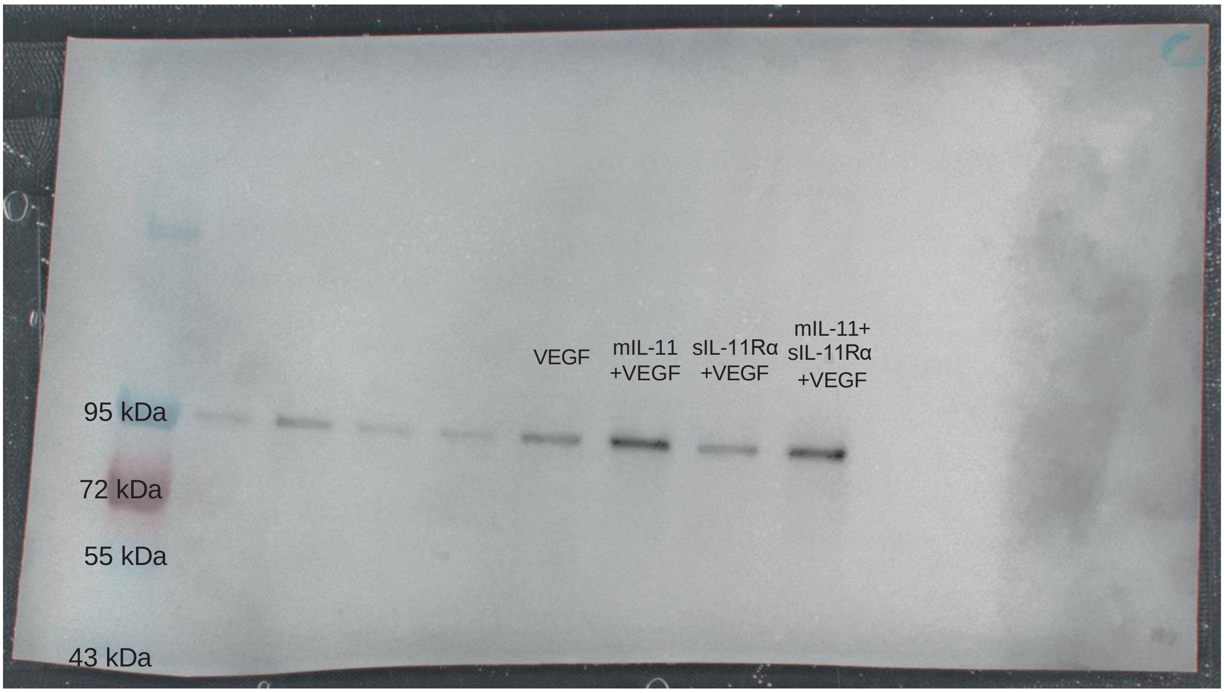

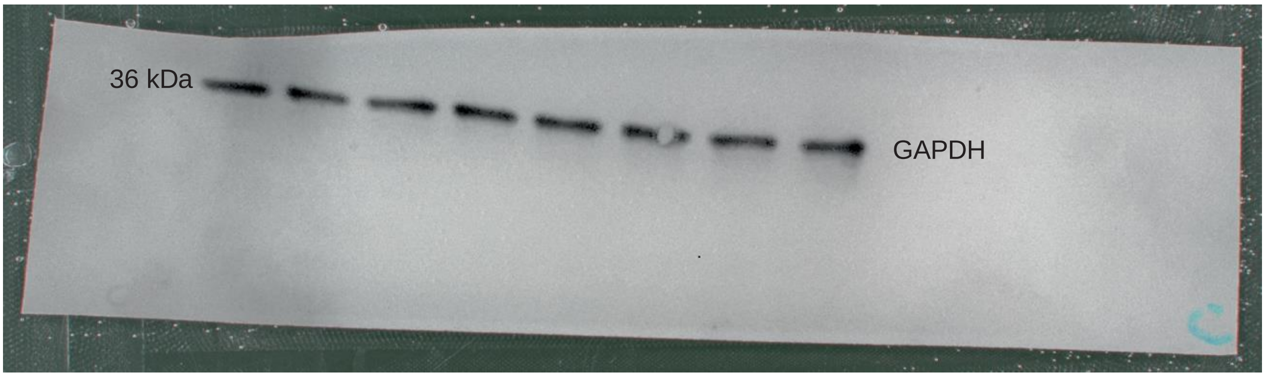

pSTAT1
84, 91 kDa

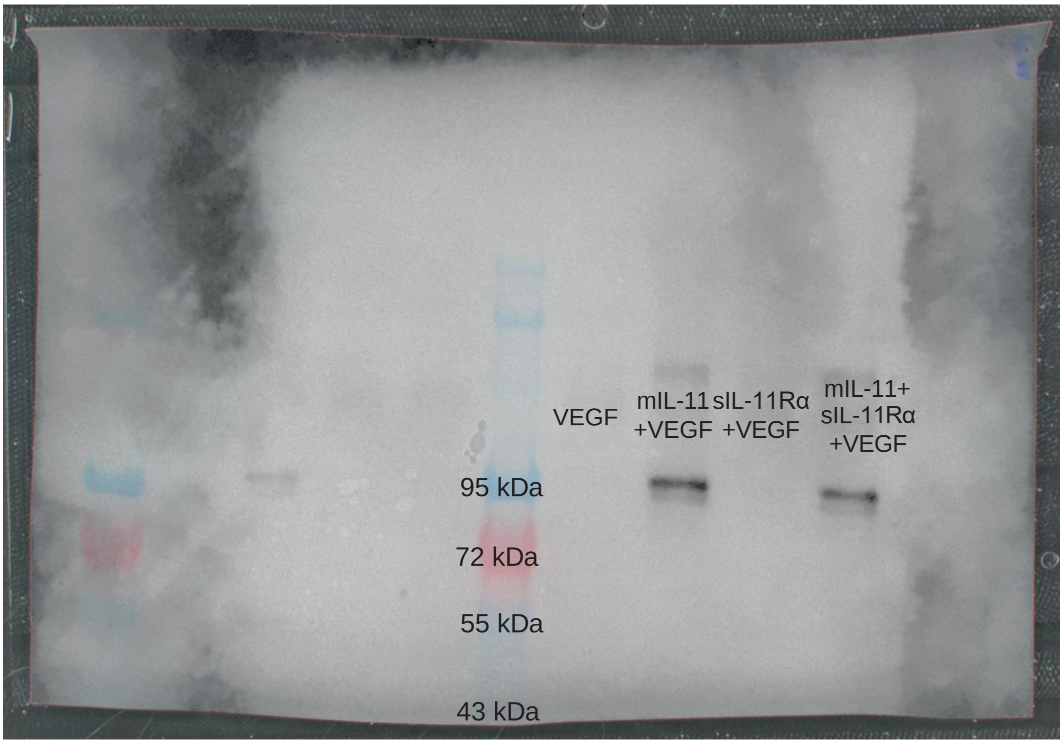

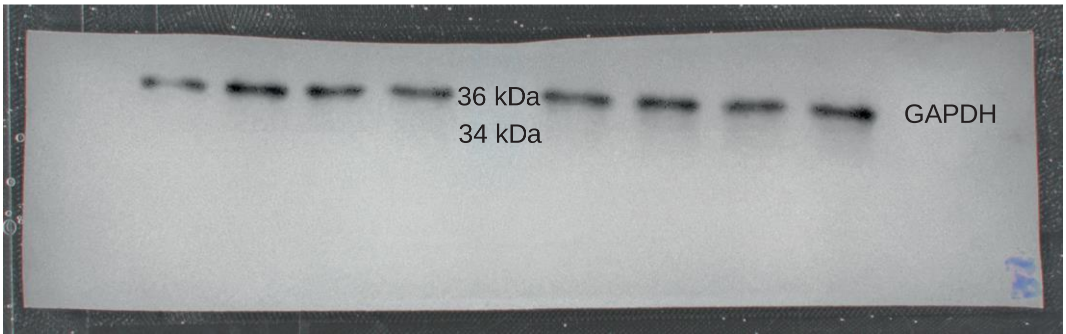

Full unedited blot for Fig. 9A #3

pSTAT3 Tyr
79, 86 kDa

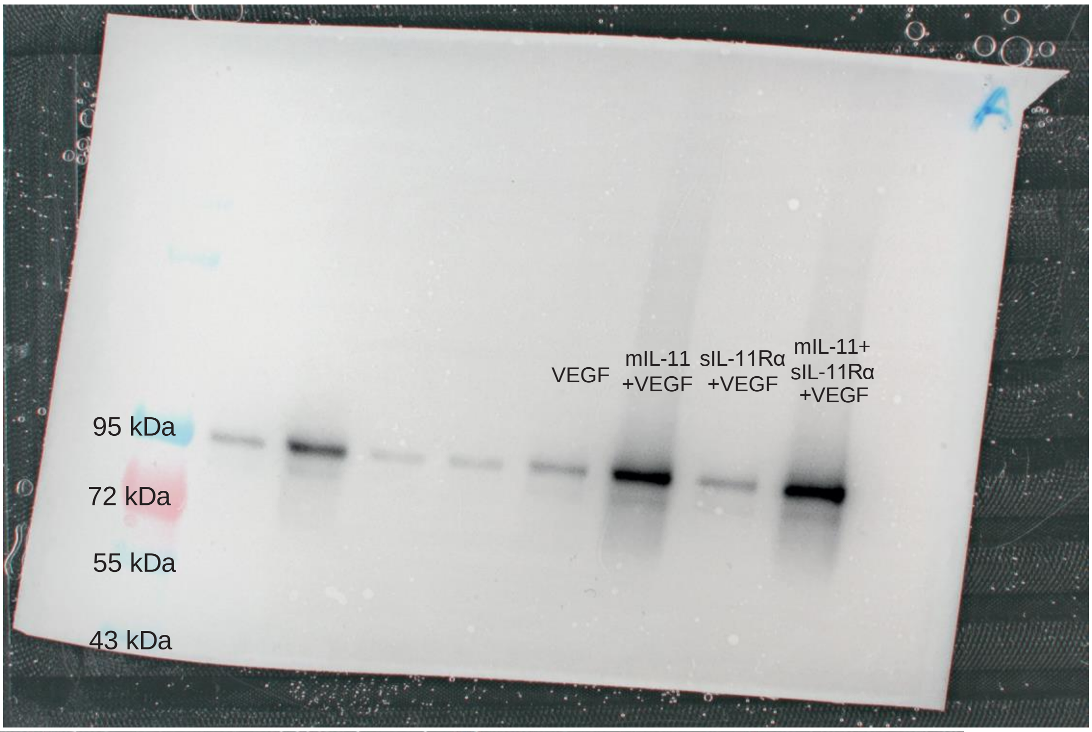

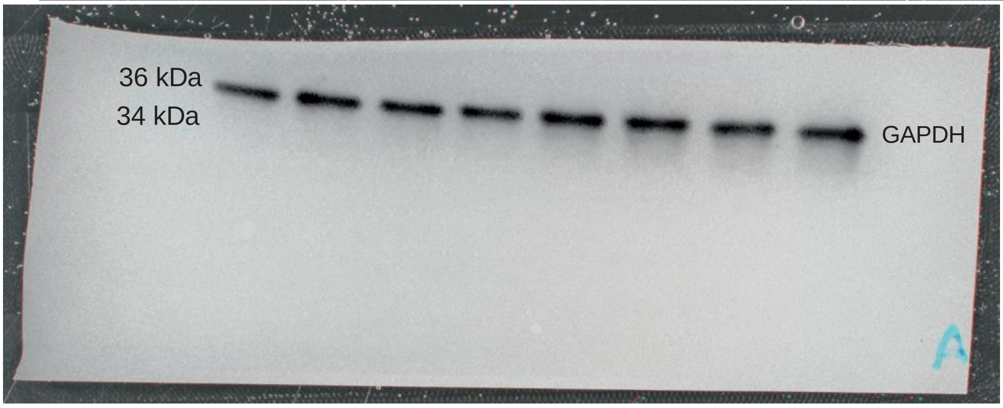

Full unedited blot for Fig. 9A #3

pSTAT5
90 kDa

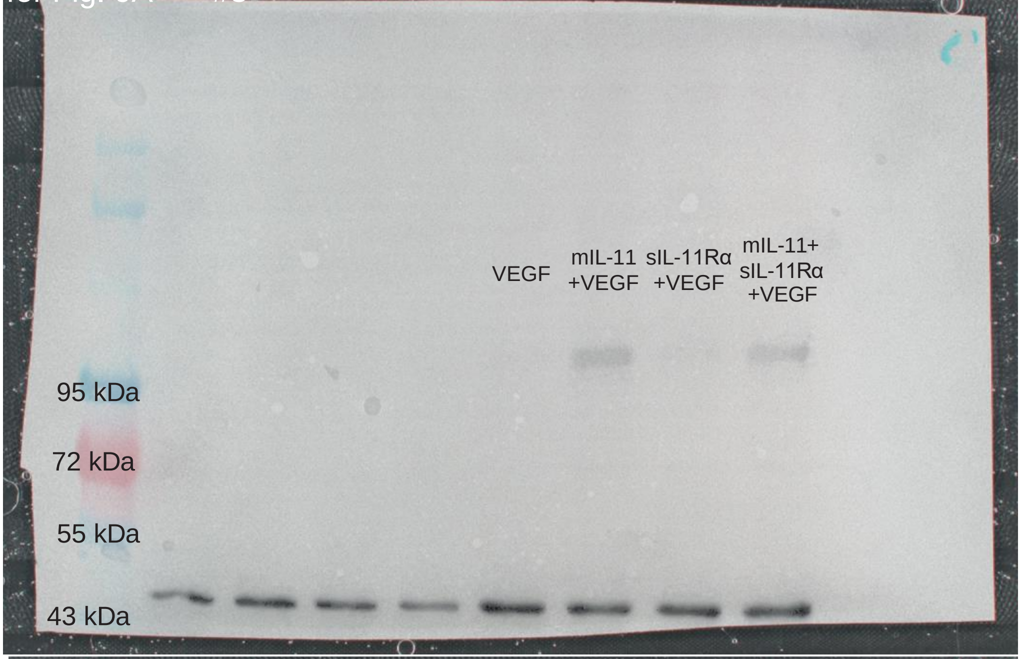

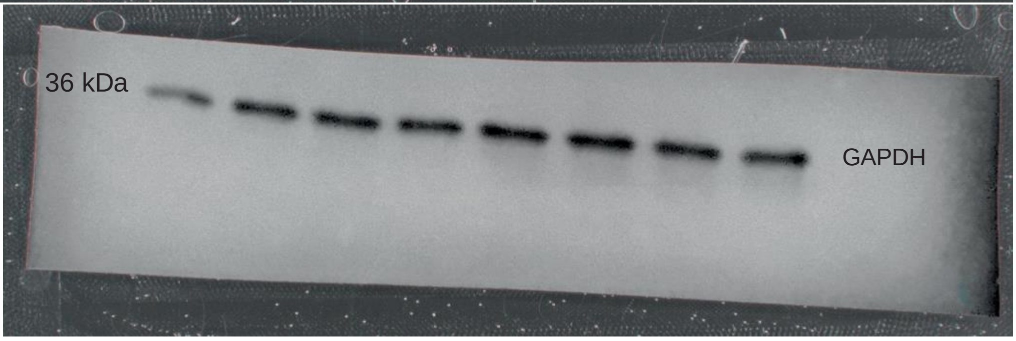

pAkt
60 kDa

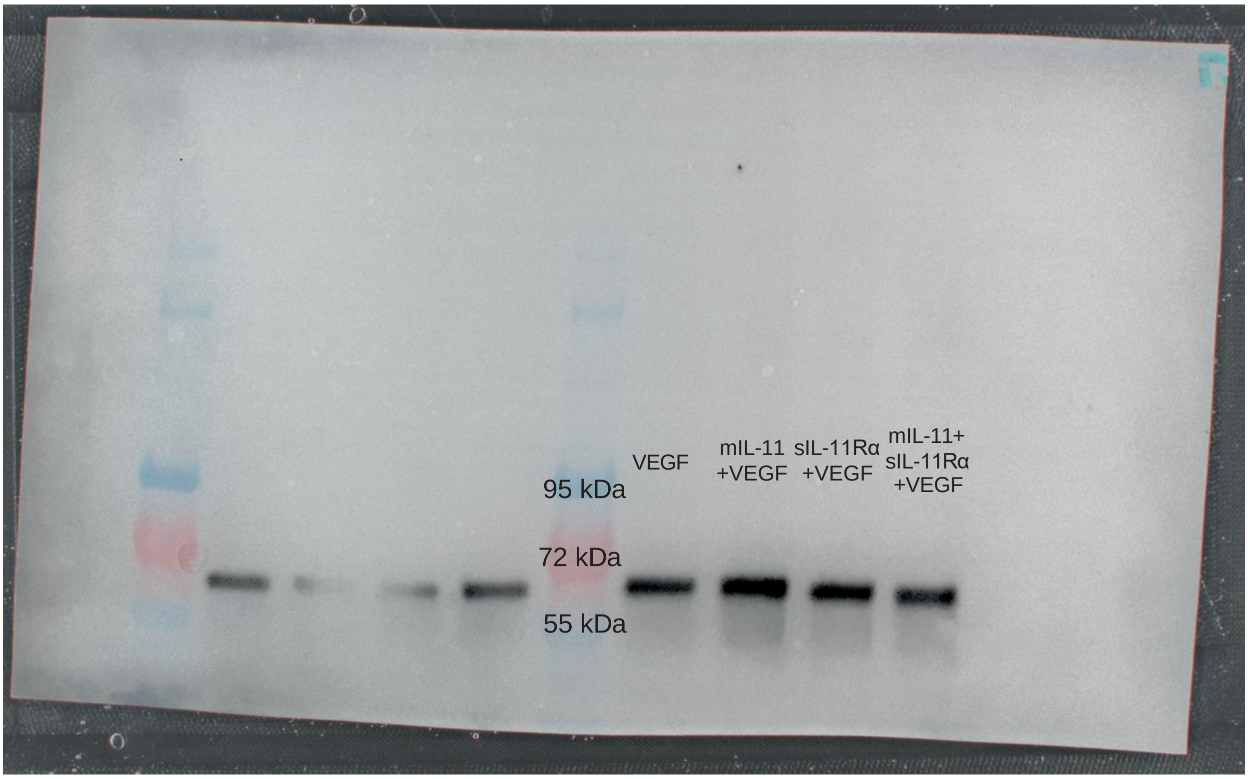

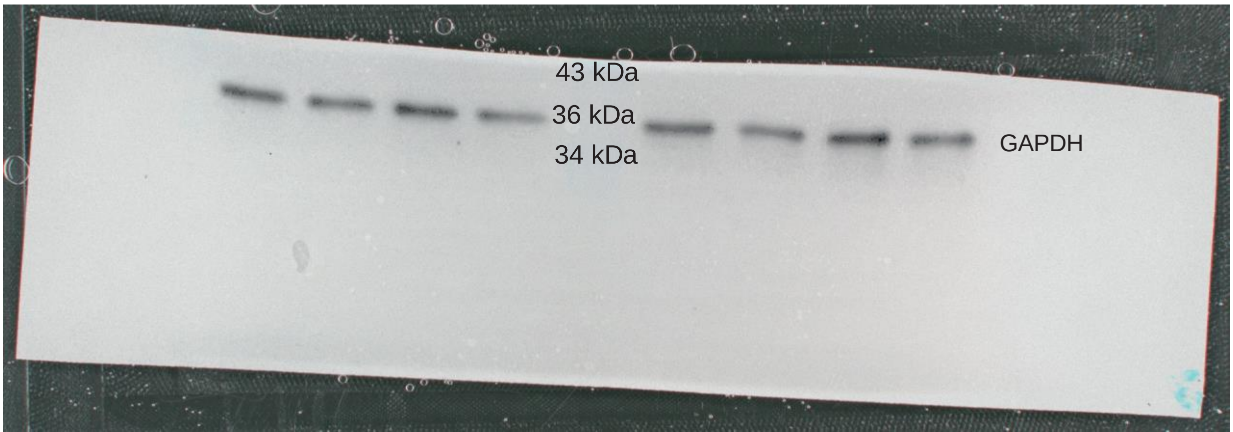

Full unedited blot for Suppl. Fig. S7 #3

pSTAT3 Tyr
79, 86 kDa

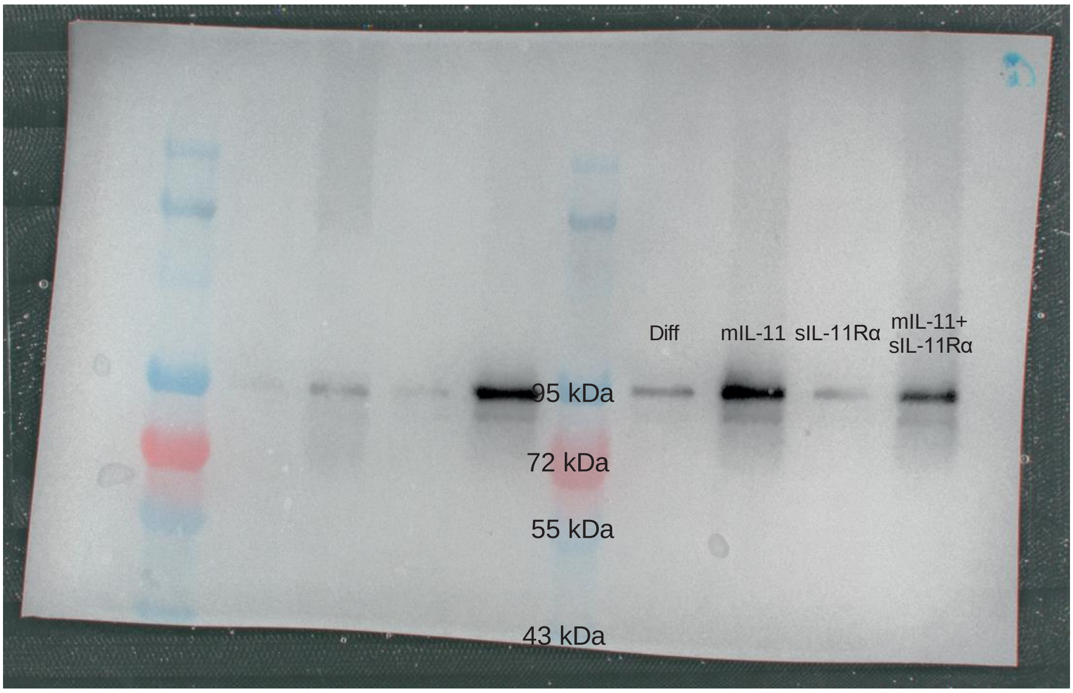

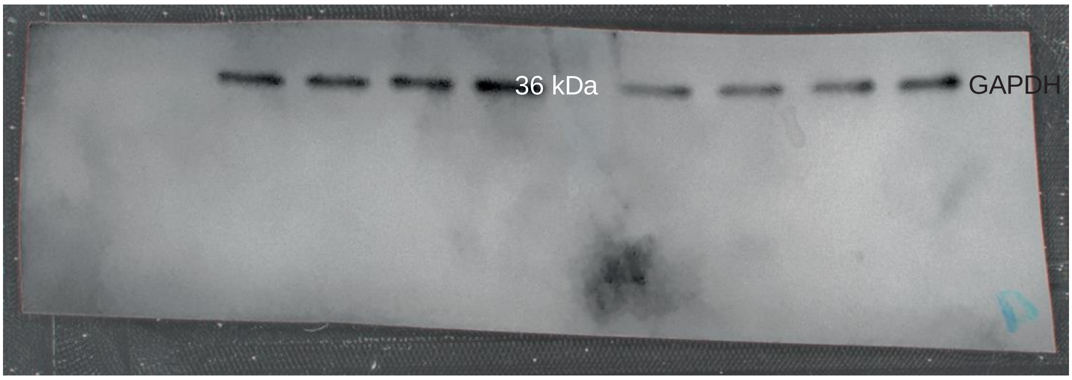

Full unedited blot for Suppl. Fig. S7 #3

pERK

44, 42 kDa

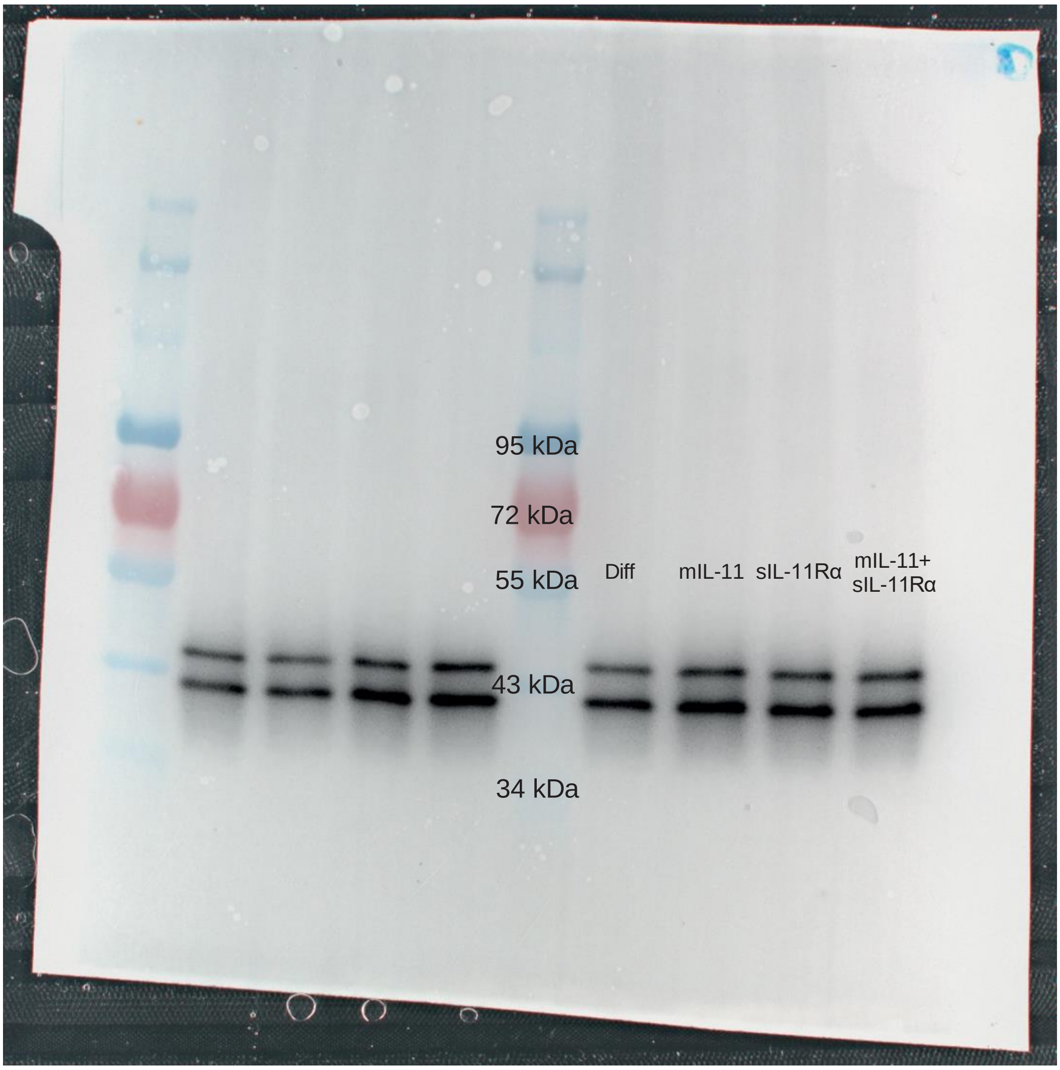

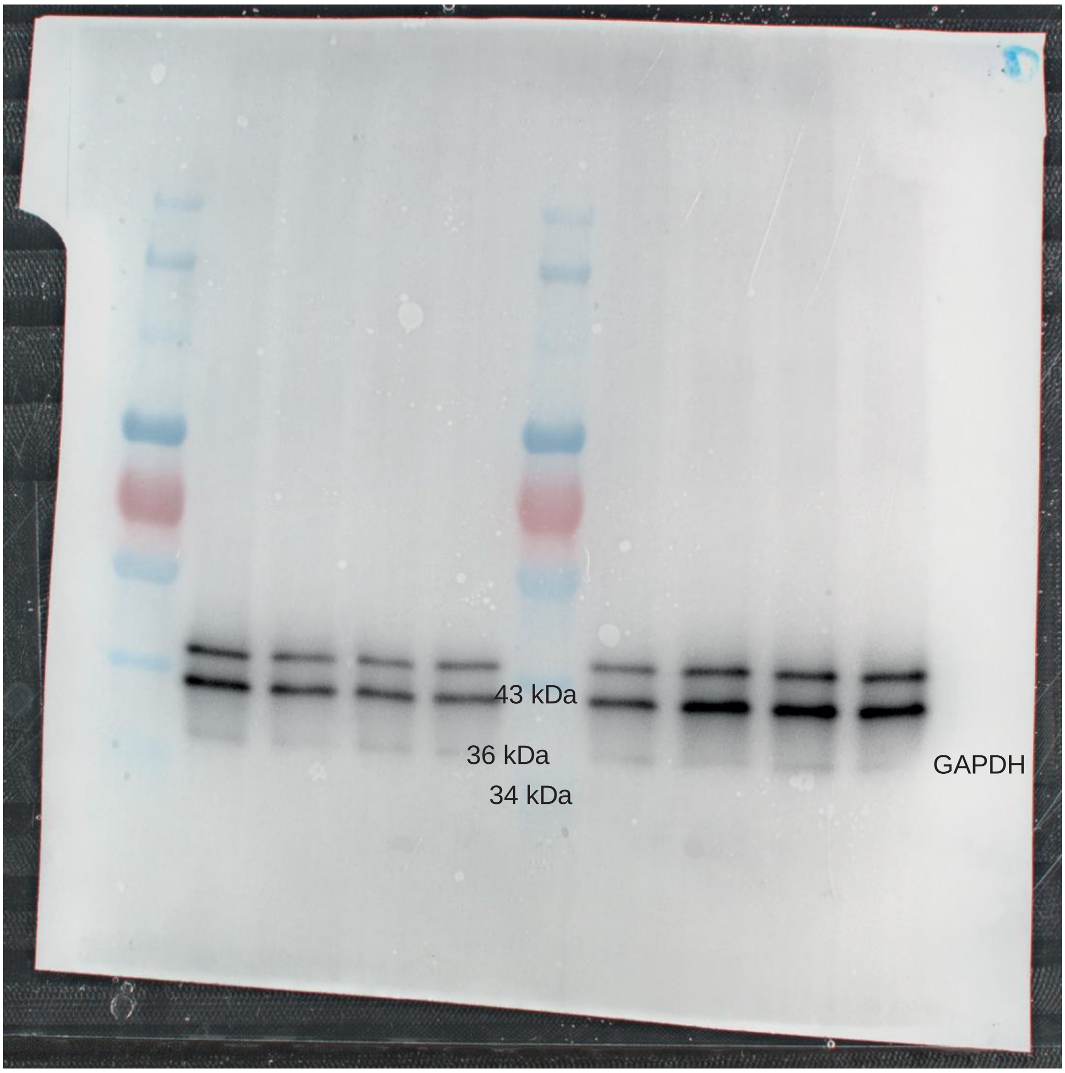

Full unedited blot for Suppl. Fig. S7 #3

pSTAT3 Ser

86 kDa

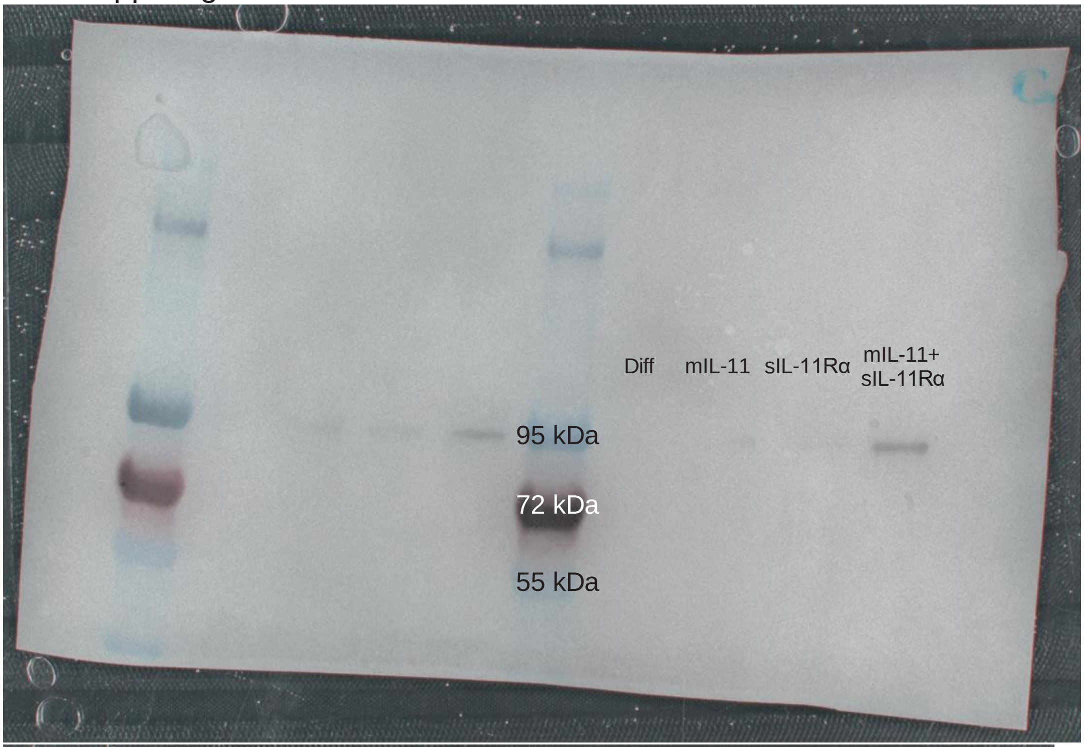

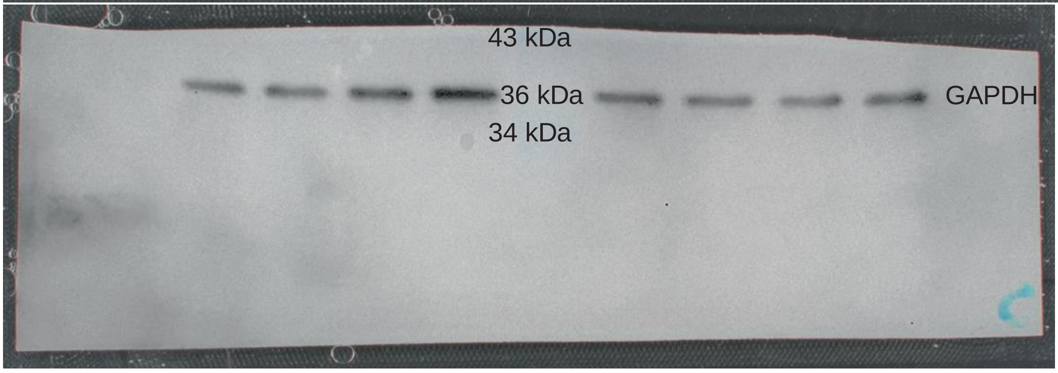

pSTAT1

84, 91 kDa

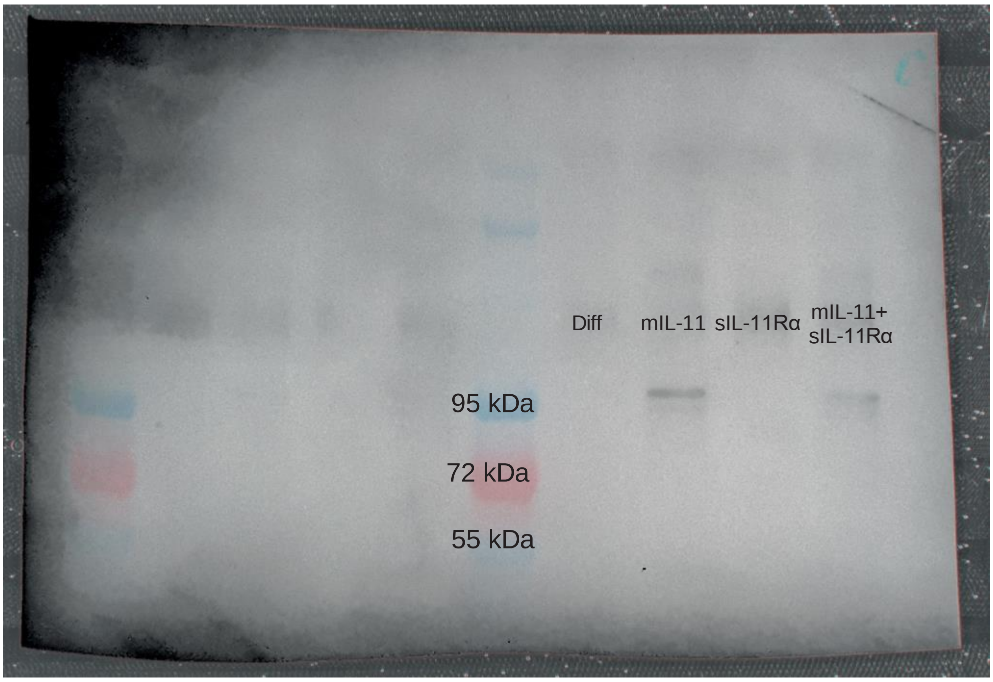

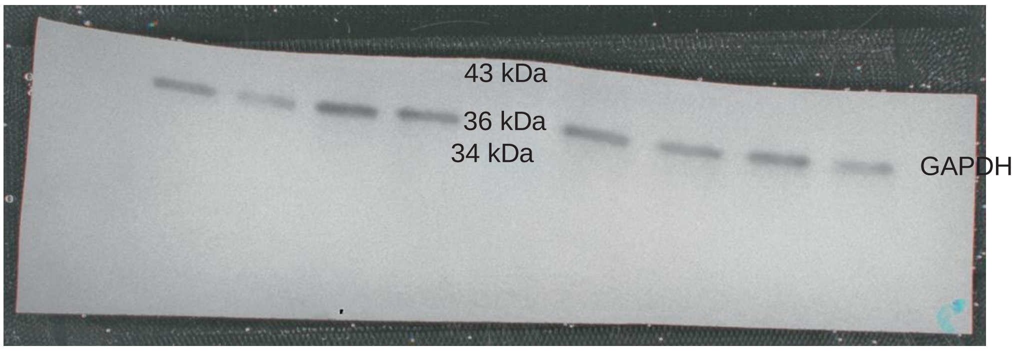

Full unedited blot for Suppl. Fig. S7 #3

pSTAT5
90 kDa

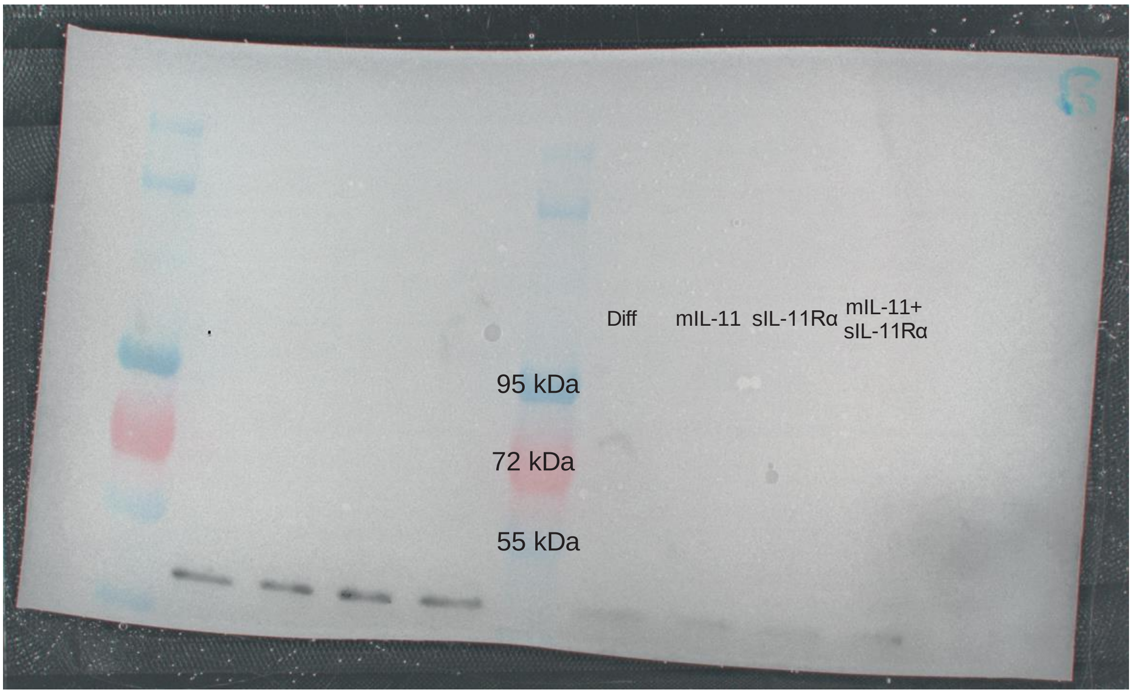

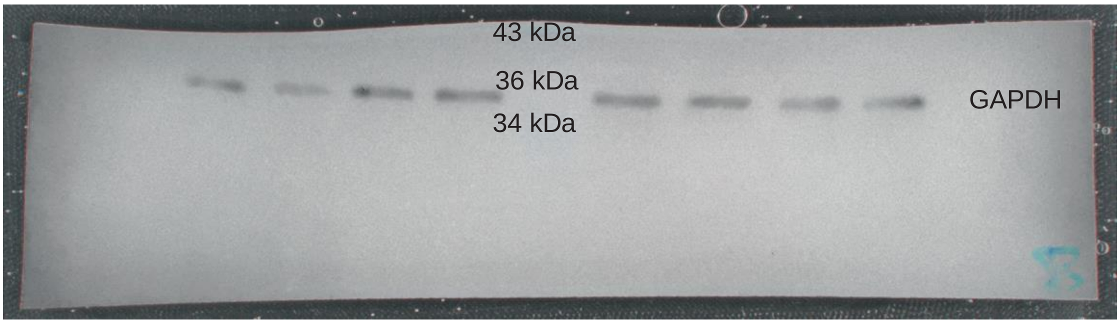

pAkt
60 kDa

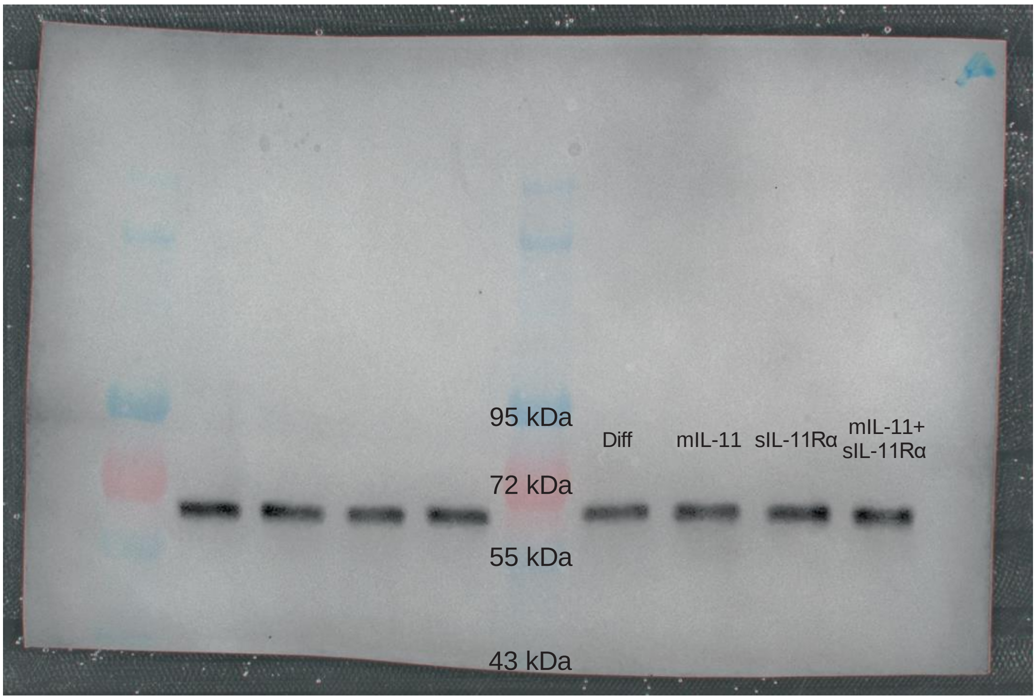

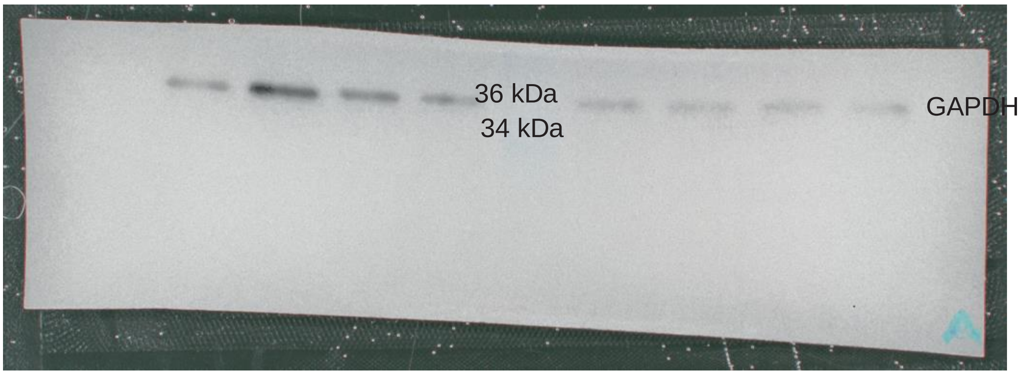

Full unedited blot for Fig. 9A #4

pSTAT3 Tyr

79, 86 kDa

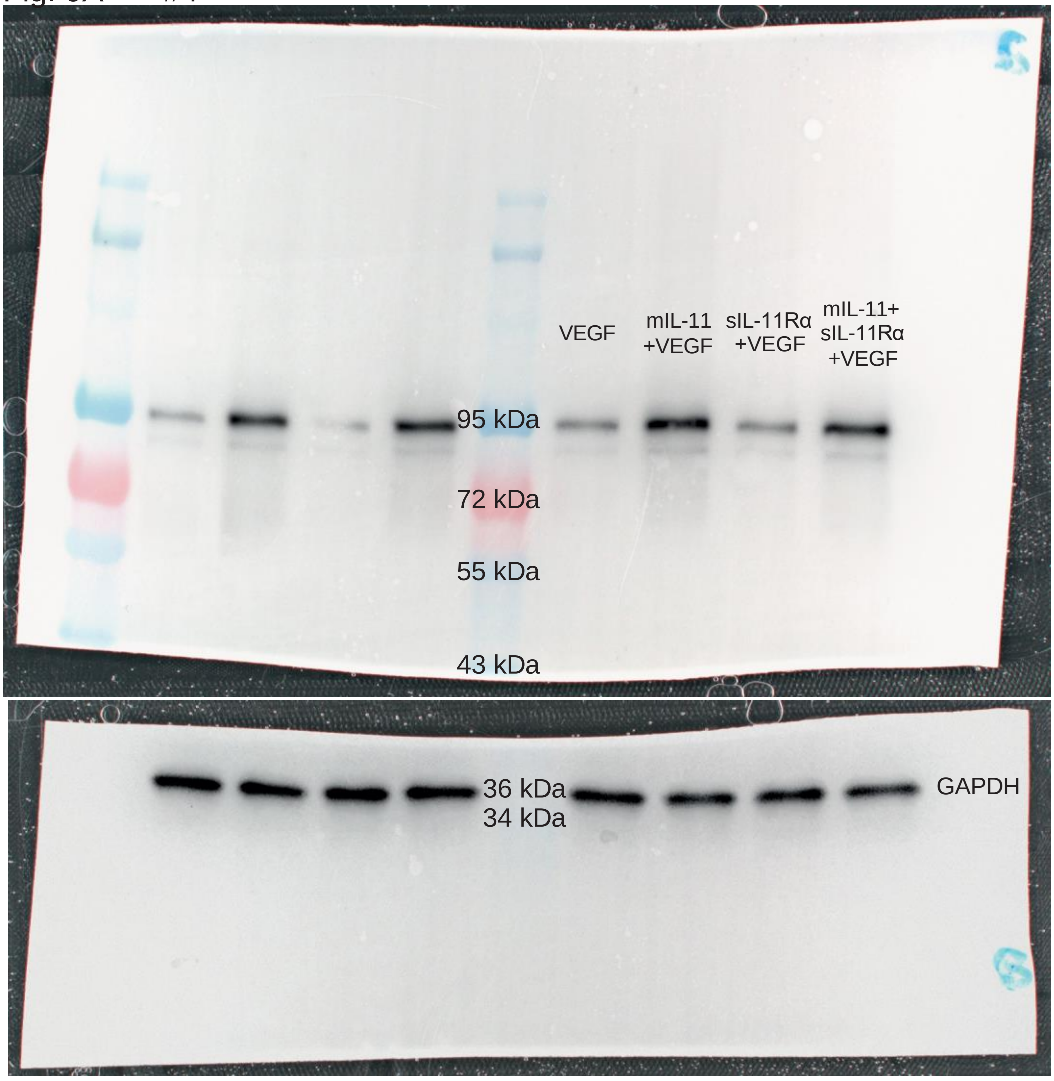

pSTAT1

84, 91 kDa

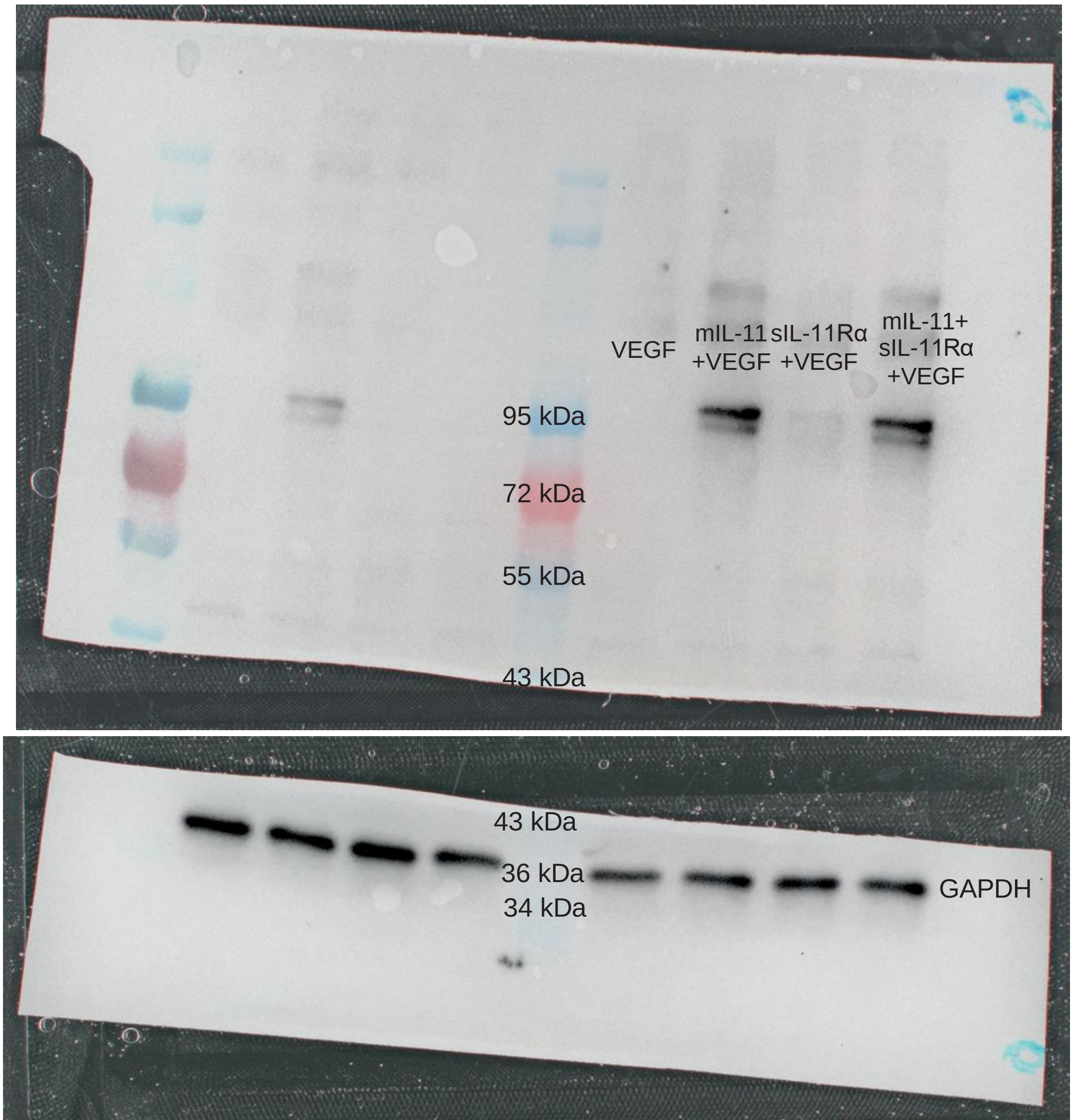

Full unedited blot for Fig. 9A #4

pERK
44, 42 kDa

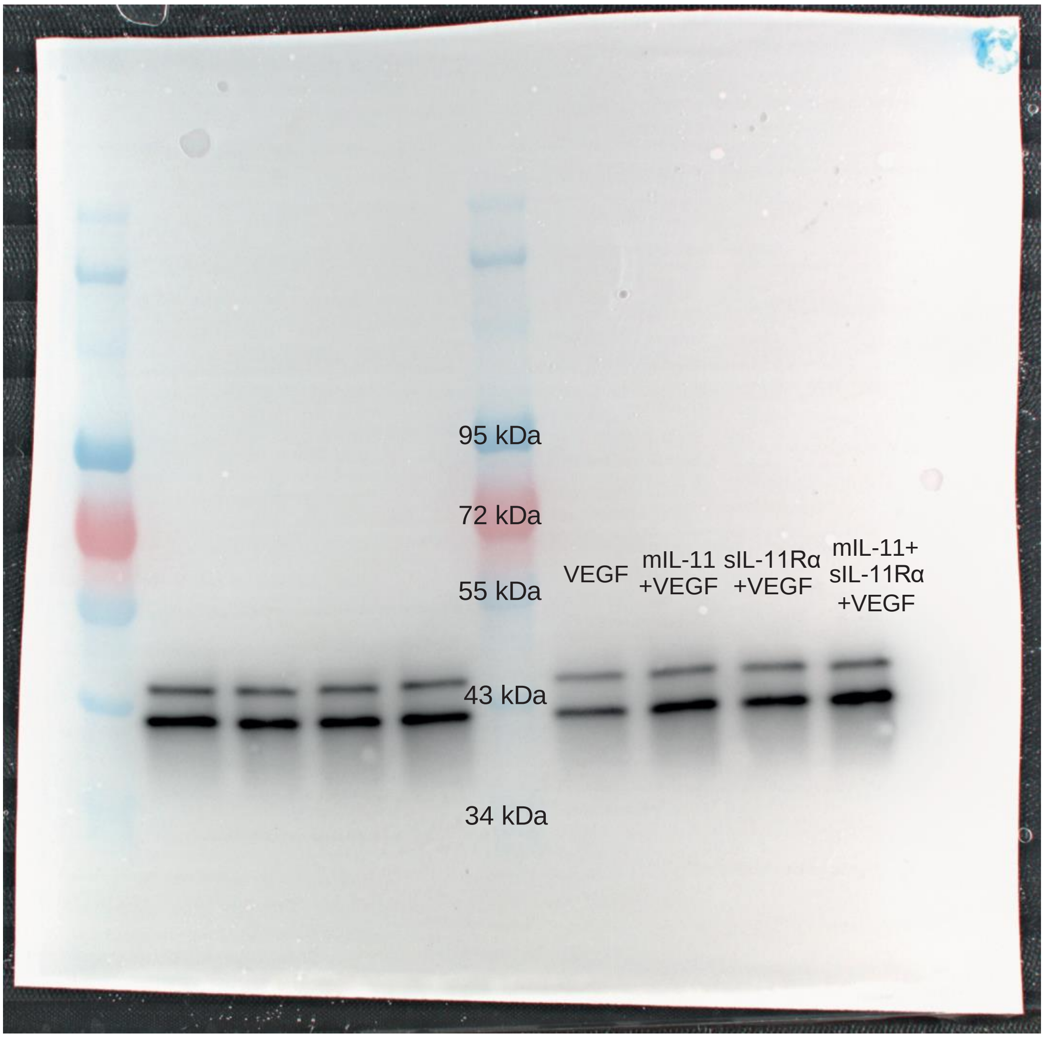

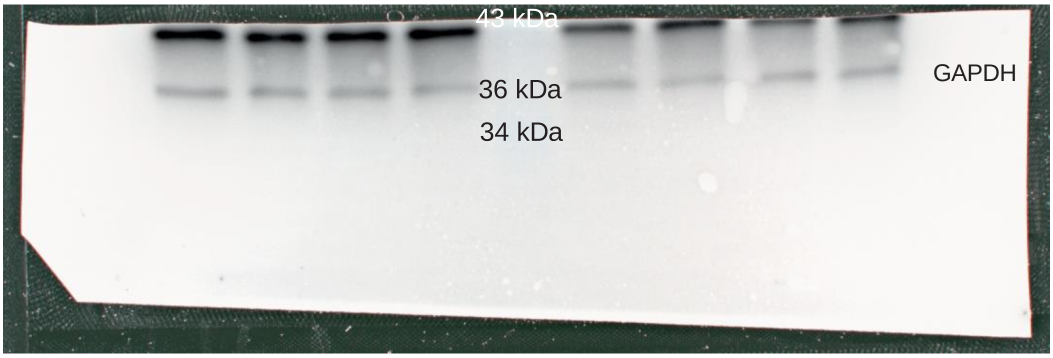

Full unedited blot for Fig. 9A #4

pSTAT3 Ser
86 kDa

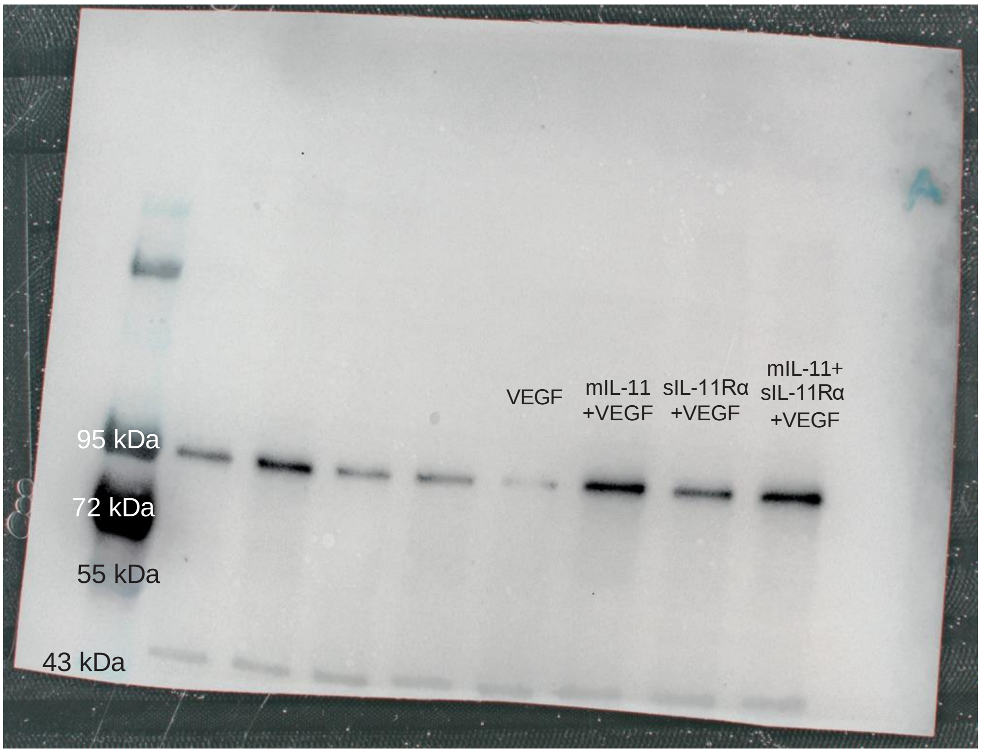

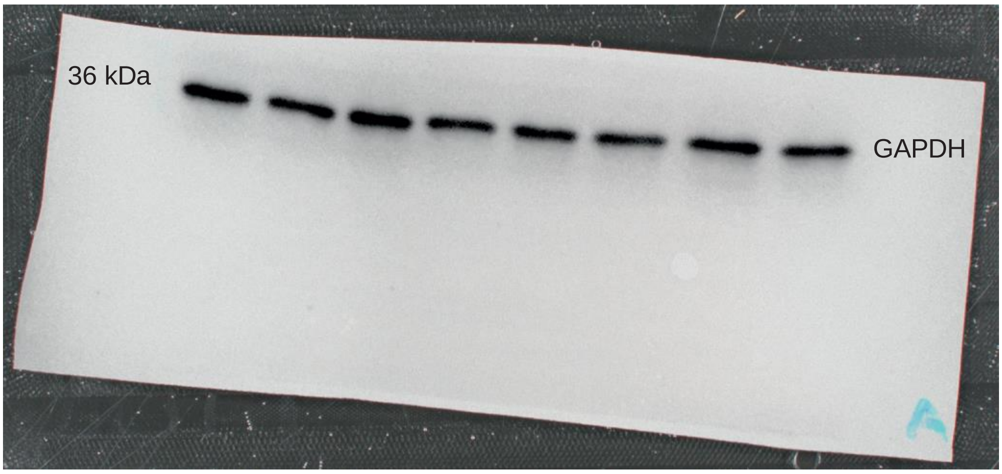

Full unedited blot for Fig. 9A #4

pSTAT5
90 kDa

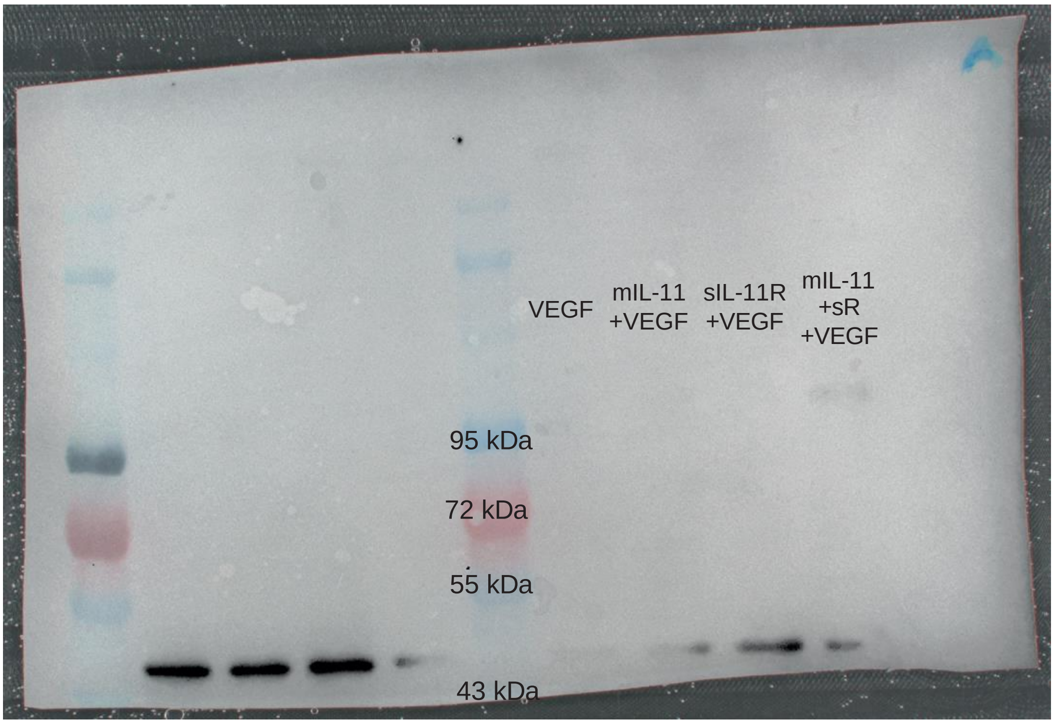

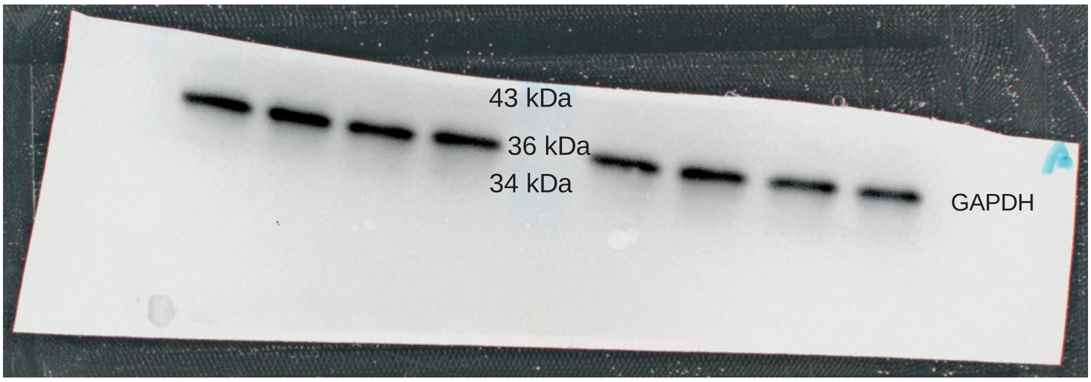

pAkt
60 kDa

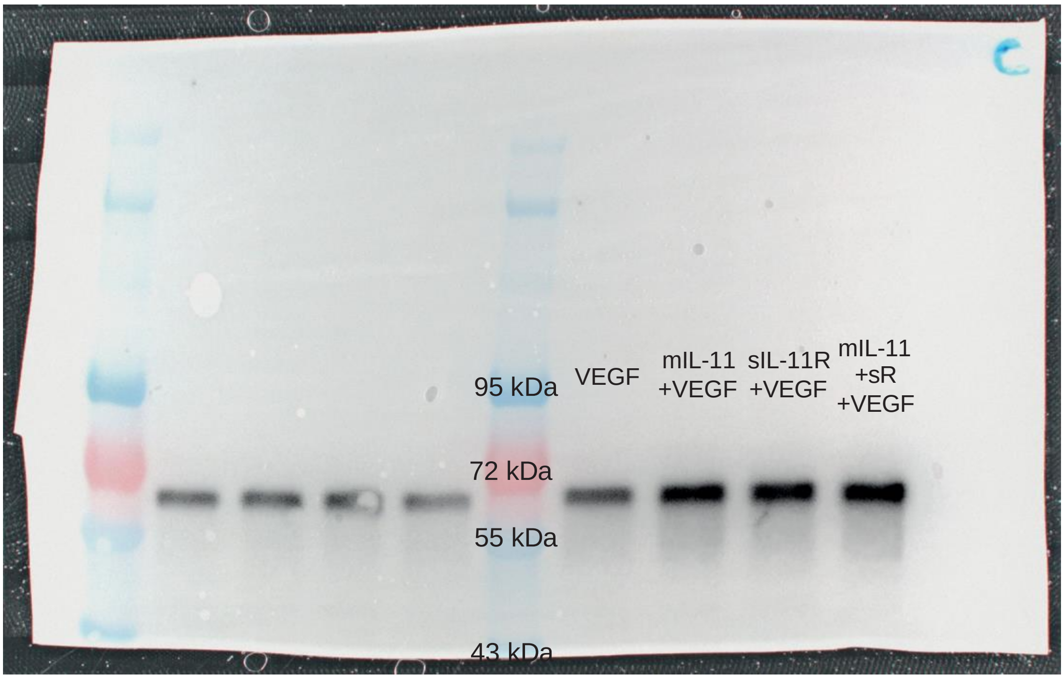

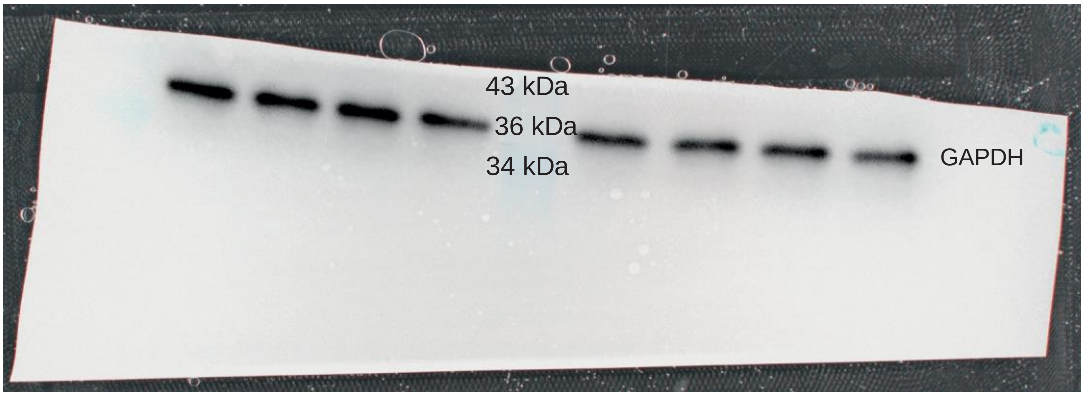

Full unedited blot for Suppl. Fig. S3B

pSTAT3 Tyr

79, 86 kDa

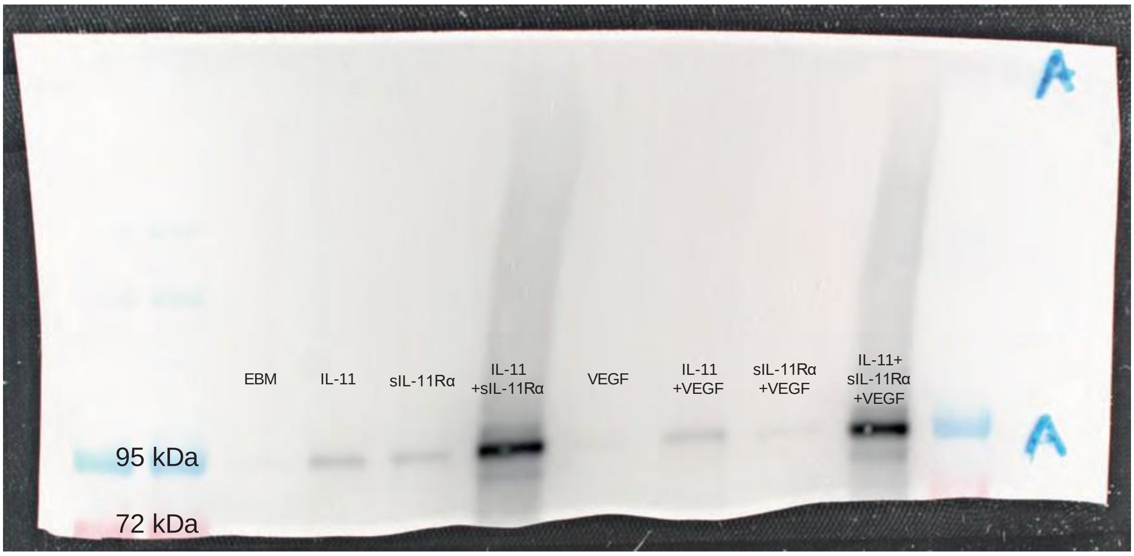

pAkt

60 kDa

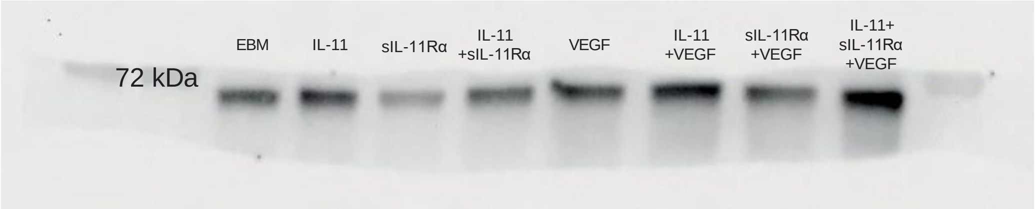

pERK

44, 42 kDa

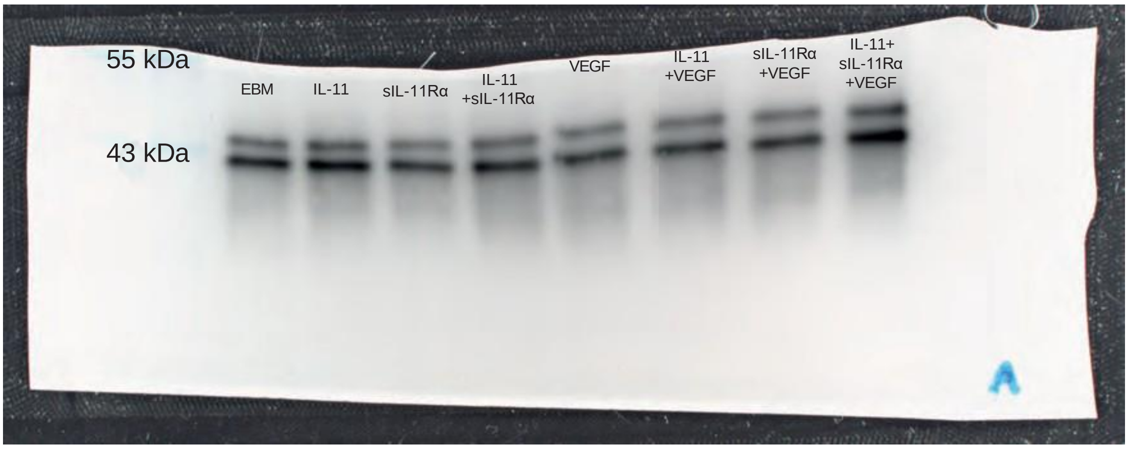

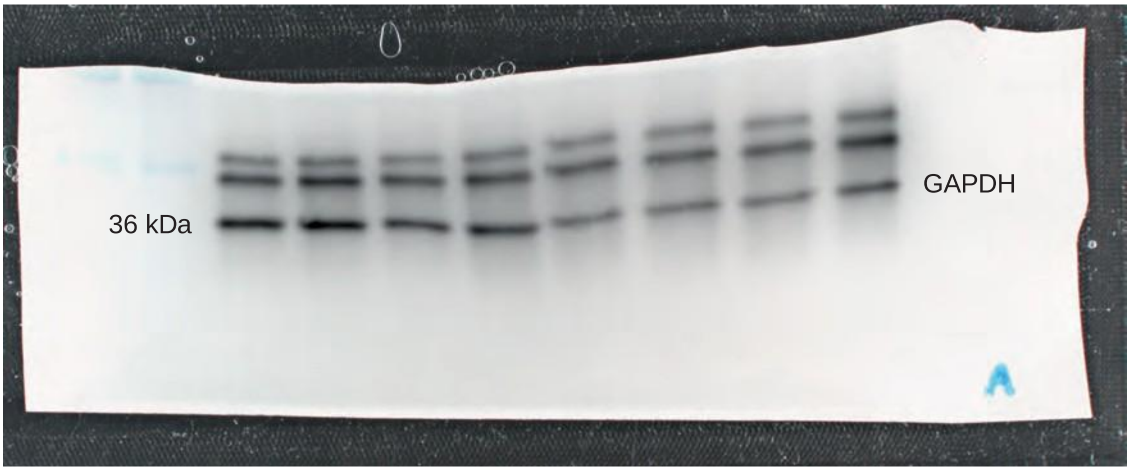

Full unedited blot for Suppl. Fig. S3B

pSTAT3 Ser
86 kDa

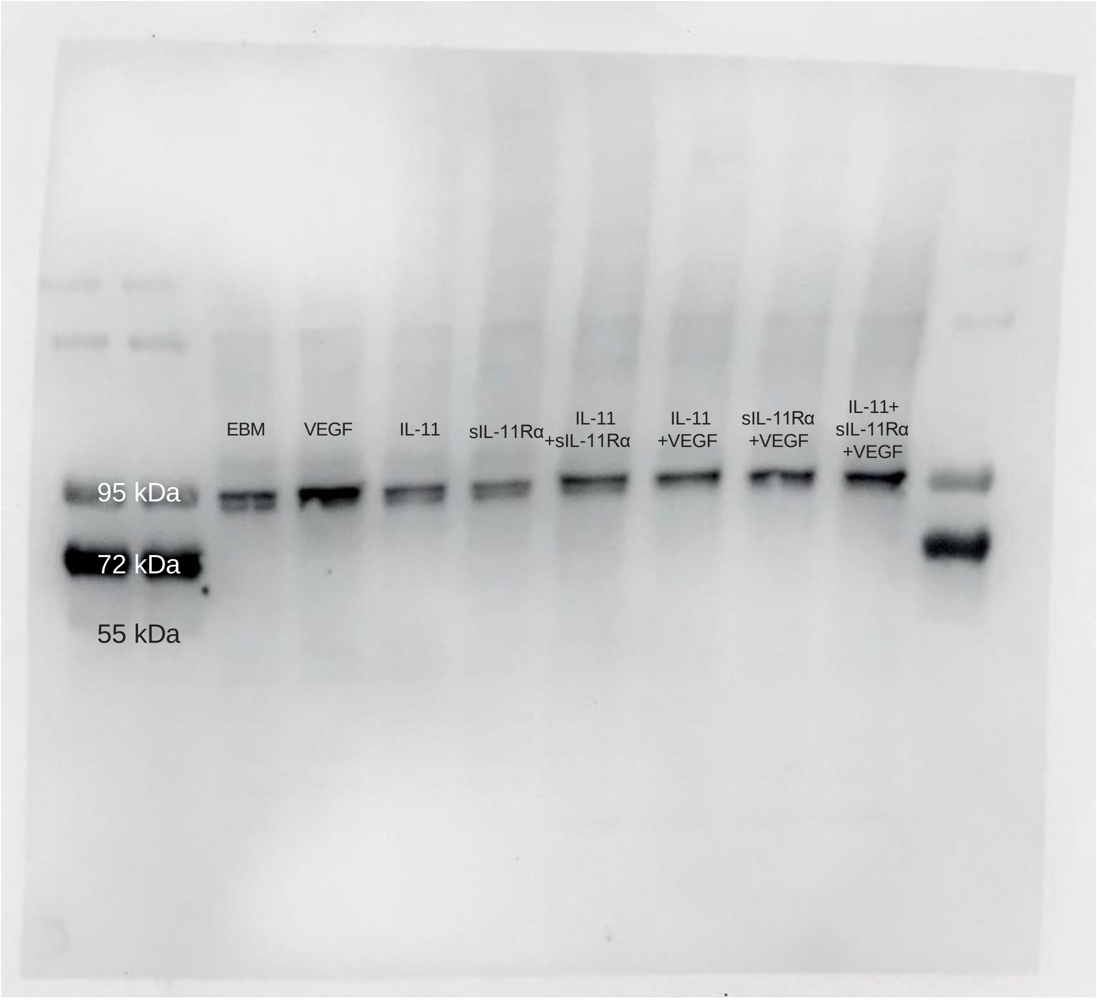

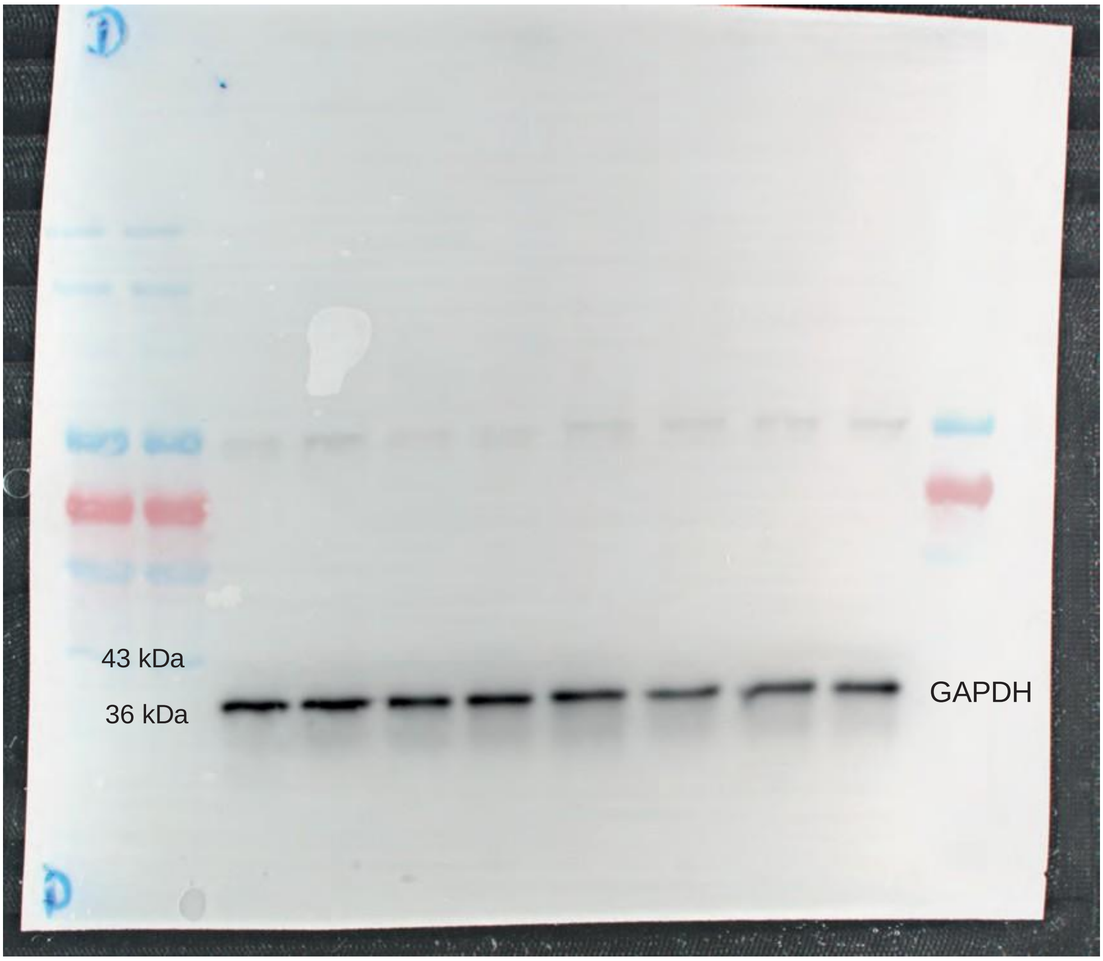

Full unedited blot for Suppl. Fig. S3B

pSTAT1

84, 91 kDa

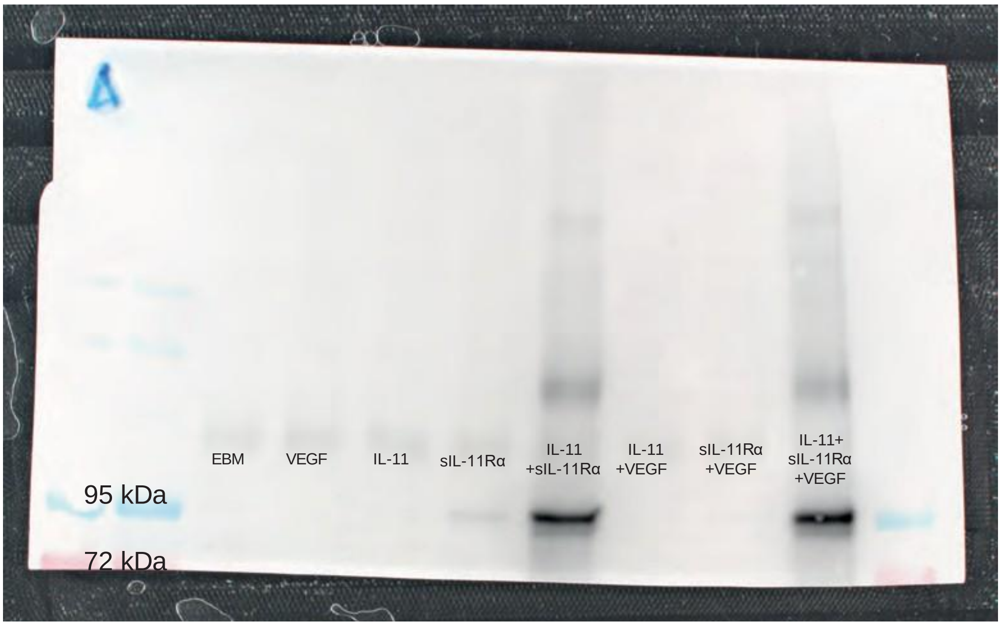

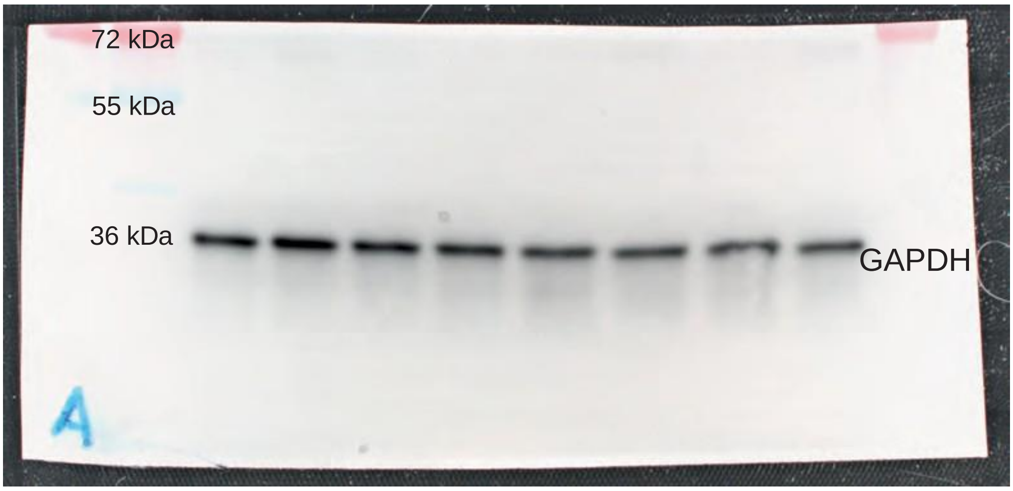

Full unedited blot for Suppl. Fig. S3B

pSTAT5

90 kDa

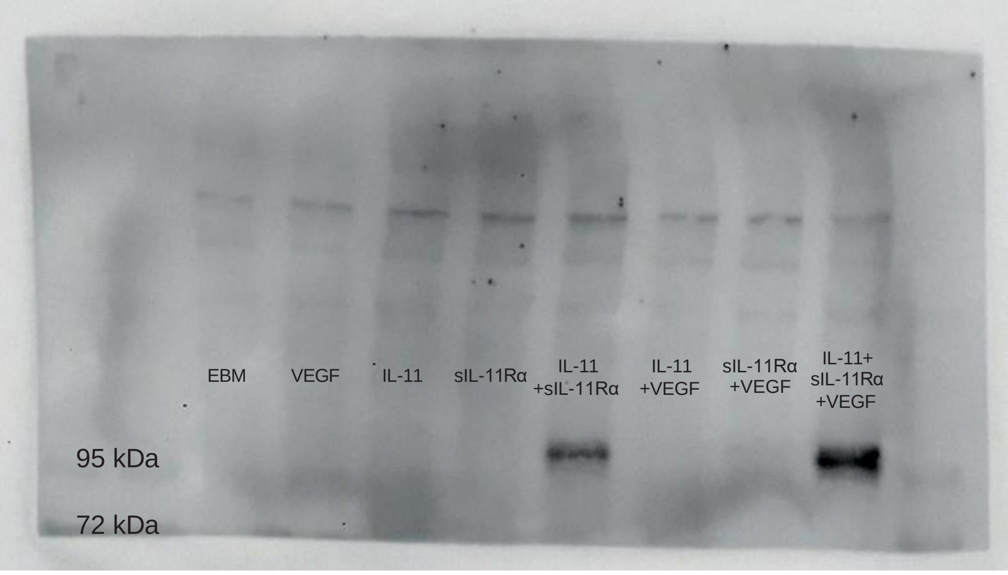

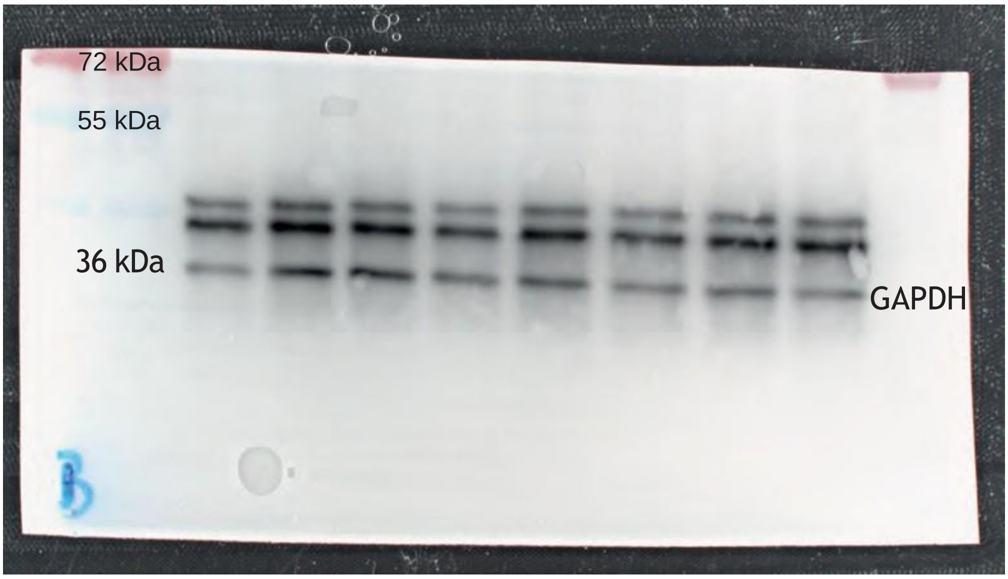

Supplement: Supplementary file 1 — Supplementary Material 1 [file 12974_2024_3223_MOESM1_ESM.pdf]
