# Supplementary material for: Characterization of the angiomodulatory effects of Interleukin 11 cis- and trans-signaling in the retina
Source: J Neuroinflammation. 2024 Sep 18;21:230. doi: 10.1186/s12974-024-03223-3 (PMC11412048; doi:10.1186/s12974-024-03223-3)
Supplement: Supplementary file 2 — Supplementary Material 2 [file 12974_2024_3223_MOESM2_ESM.pdf]

A

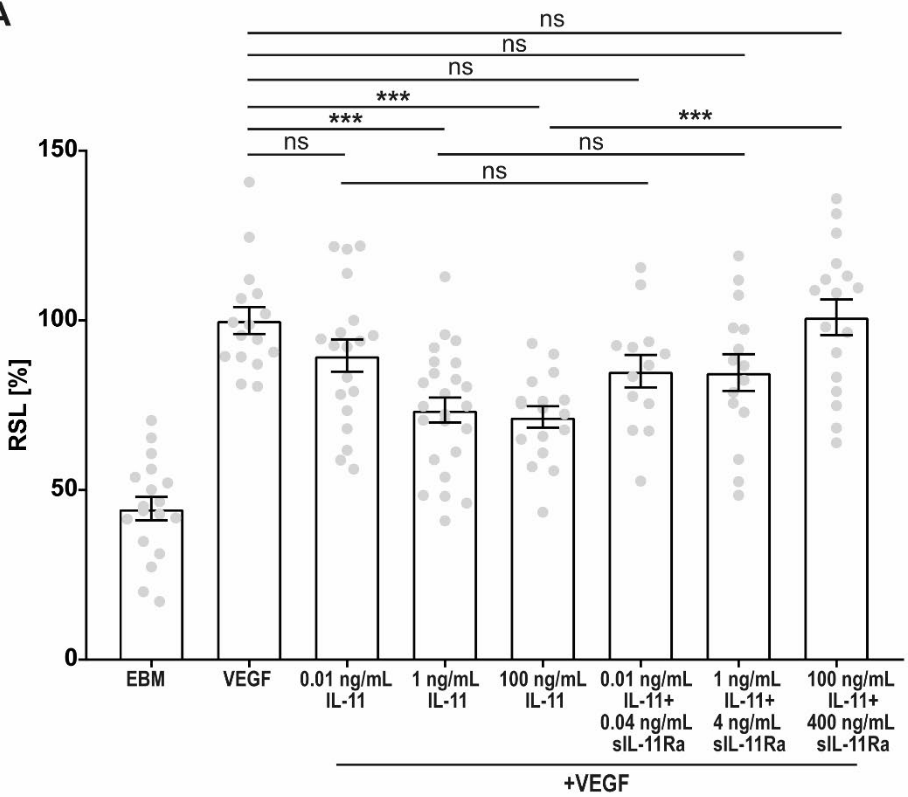
ns
ns
ns
***
***
***
ns
ns
ns
150
100
50
0
RSL [%]
EBM
VEGF
0.01 ng/mL IL-11
1 ng/mL IL-11
100 ng/mL IL-11
0.01 ng/mL IL-11+ 0.04 ng/mL sIL-11Ra
1 ng/mL IL-11+ 4 ng/mL sIL-11Ra
100 ng/mL IL-11+ 400 ng/mL sIL-11Ra
+VEGF

B

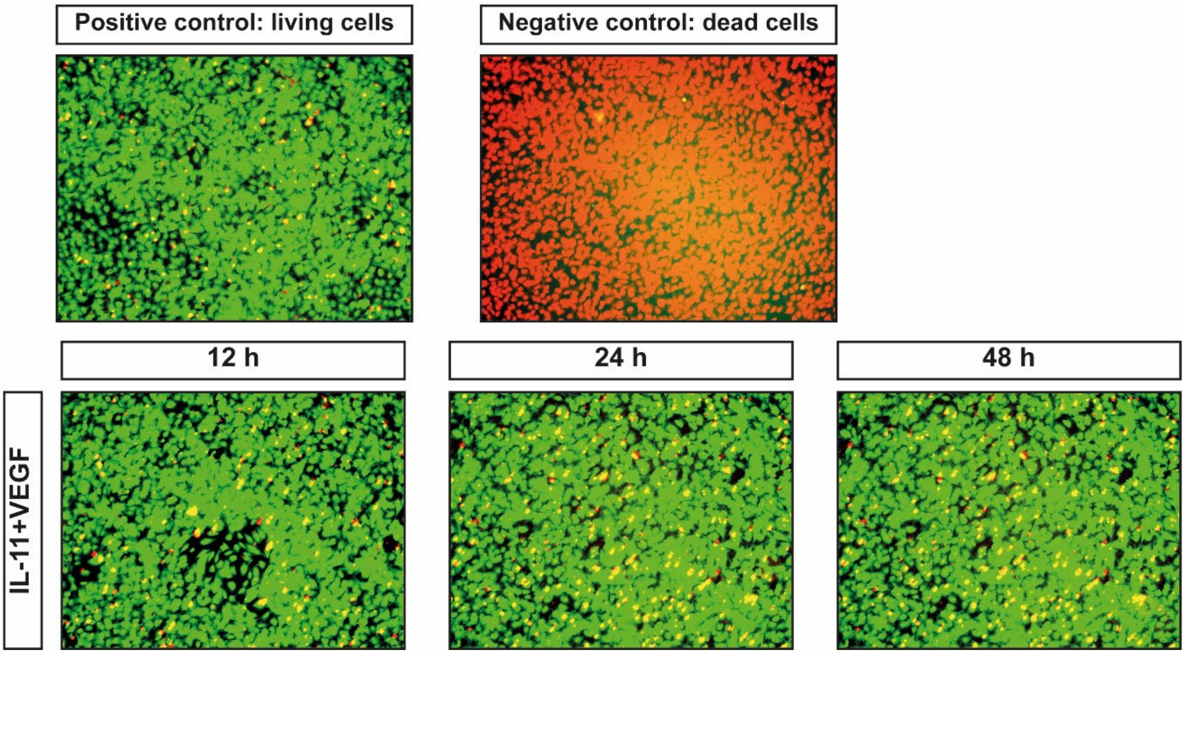
Positive control: living cells
Negative control: dead cells
12 h
24 h
48 h
IL-11+VEGF

Supplemental Figure S1: Comprehensive Analysis of IL-11 cis- and trans-signaling

(A) Dose-response experiment in the spheroid sprouting assay using HUVECs. N = 1 experiment with 13-24 spheroids per group. A significant difference in sprouting was observed between cis- and trans-signaling pathways only at 100 ng/mL IL-11 and 400 ng/mL sIL-11Rα. Statistical test: Kruskal-Wallis Test adjusted for multiple testing, *$p<0.001$. Relative sprouting length (RSL).

(B) Calcein staining of HUVECs after incubation of IL-11+VEGF for 12 h, 24 h and 48 h. Red-stained cells represent dead cells.

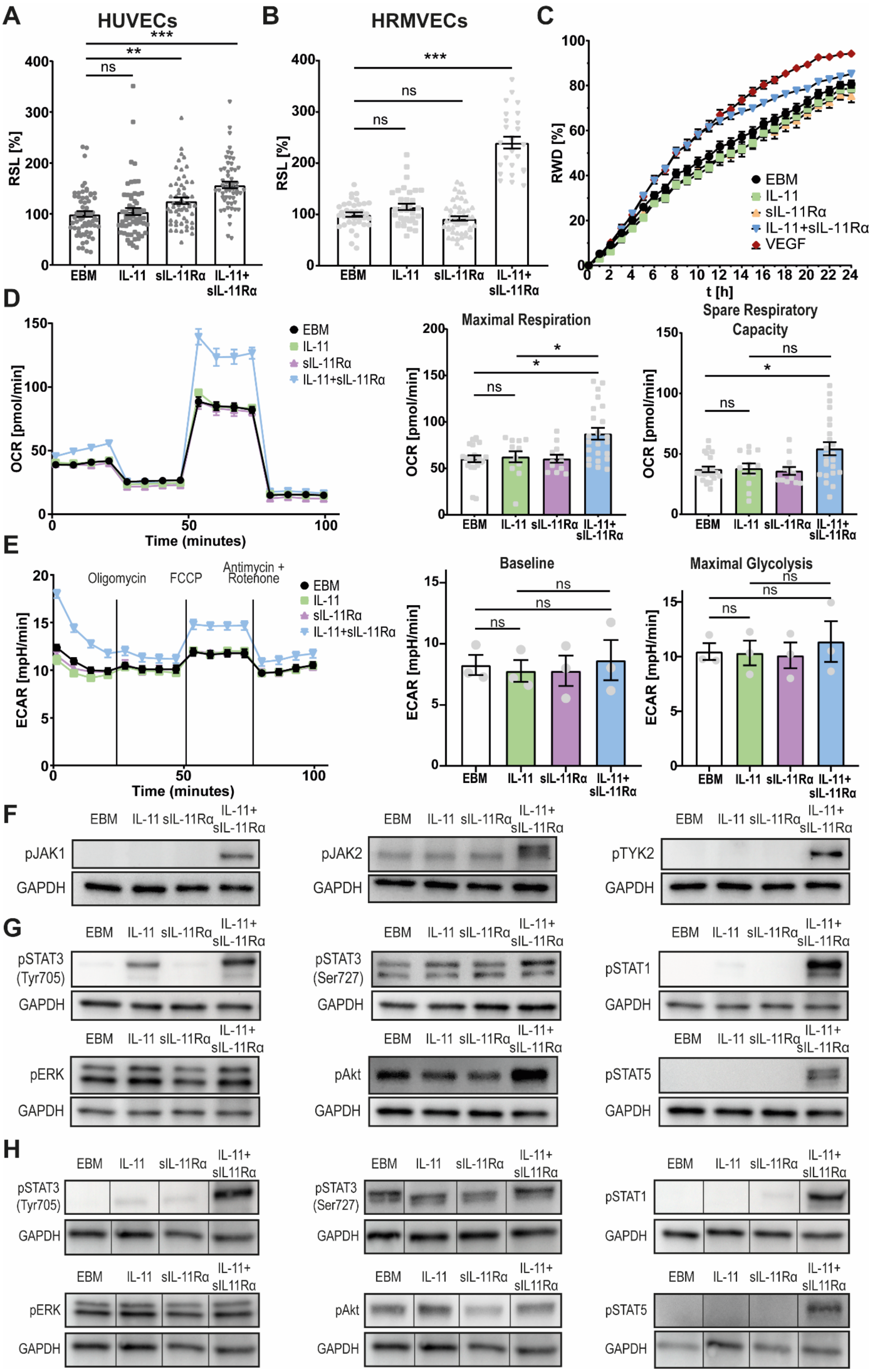

A
HUVECs
B
HRMVECs
C
RSL [%]
EBM
IL-11
sIL-11Rα
IL-11+ sIL-11Rα
ns
**
***
RWD [%]
t [h]
VEGF
IL-11+sIL-11Rα
D
OCR [pmol/min]
Time (minutes)
Maximal Respiration
Spare Respiratory Capacity
E
ECAR [mpH/min]
Oligomycin
FCCP
Antimycin + Rotenone
Baseline
Maximal Glycolysis
F
pJAK1
pJAK2
pTYK2
GAPDH
G
pSTAT3 (Tyr705)
pSTAT3 (Ser727)
pSTAT1
pERK
pAkt
pSTAT5
H
IL-11+ sIL11Rα

Supplemental Figure S2: Effects of IL-11 cis- and trans-signaling

(A) Spheroid sprouting assay: Relative sprouting length (RSL) of HUVECs: IL-11+sIL-11Rα showed significantly more sprouting compared to EBM (negative control). N = 3 independent experiments with 15-22 spheroids per group and experiment. Statistical test: Kruskal-Wallis Test adjusted for multiple testing, *p<0.01.

(B) Spheroid sprouting assay: Relative sprouting length (RSL) of HRMVECs: IL-11+sIL-11Rα showed significantly more sprouting compared to EBM (negative control). N = 2 independent experiments with 12-25 spheroids per group and experiment. Statistical test: Kruskal-Wallis Test adjusted for multiple testing, *p<0.01.

(C) Scratch wound assay using HUVECs: IL-11+sIL-11Rα exerts a pro-migratory effect while IL-11 showed no effect. Relative Wound Density (RWD). N = 3 independent experiments each including 6-8 technical replicates.

(D) Representative graph of the oxygen consumption rate (OCR) of HUVECs after pretreatment with above mentioned cytokines for 15 h. Normalized quantification of Seahorse Stress Test regarding maximal respiration and spare respiratory capacity. N = 3 with each 4-8 technical replicates. Statistical test: Kruskal-Wallis Test adjusted for multiple testing, *p<0.05.

(E) Representative graph of the normalized extracellular acidification rate (ECAR) of HUVECs after pretreatment with above mentioned cytokines for 15 h. N = 3 with each 4-8 technical replicates. Statistical test: Friedman Test adjusted for multiple testing, *p<0.05.

(F) Western blot screening for possible JAK activation. HUVECs were stimulated with EBM, IL-11, sIL-11Rα or IL-11+sIL-11Rα for 15 min. N = 3 independent experiments.

(G) Western blot screening for the following possible activated signaling molecules: pSTAT3 Tyr705, pSTAT3 Ser727, pSTAT1, pERK, pAkt, pSTAT5. HUVECs were stimulated with EBM, IL-11, sIL-11Rα or IL-11+sIL-11Rα for 15 min. N = 3 independent experiments.

(H) Western blot of activated signaling pathways in HRMVECs after stimulation with EBM, IL-11, sIL-11Rα, IL-11+sIL-11Rα for 15 min. N = 1 experiment.

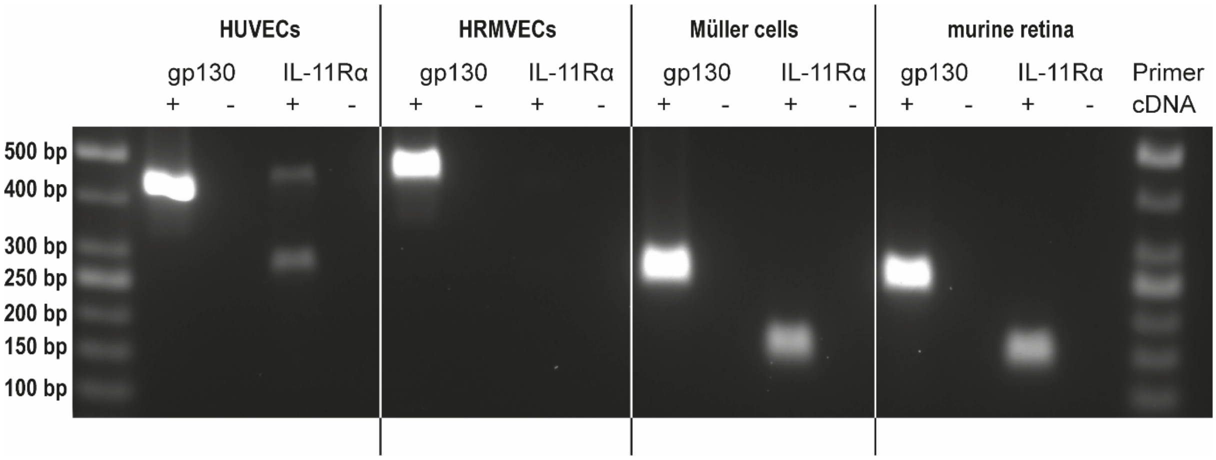

Supplemental Figure S3: Expression status of gp130 and IL-11Rα in HUVECs, HRMVECs, murine Müller cells and murine retina

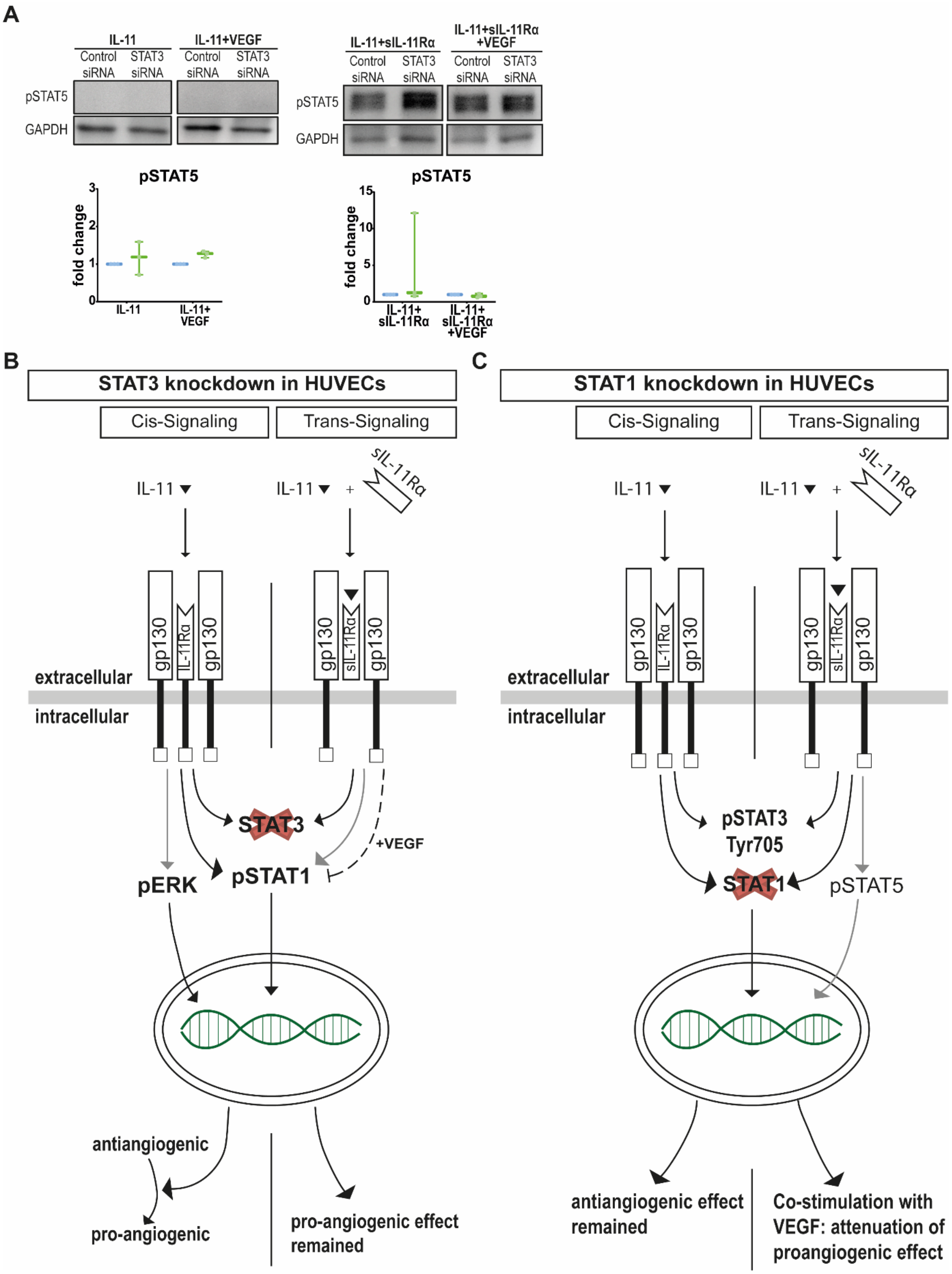
A
IL-11
IL-11+VEGF
Control siRNA
STAT3 siRNA
pSTAT5
GAPDH
IL-11+sIL-11Rα
IL-11+sIL-11Rα +VEGF
pSTAT5
fold change
IL-11
IL-11+ VEGF
IL-11+ sIL-11Rα
IL-11+ sIL-11Rα +VEGF
B
STAT3 knockdown in HUVECs
Cis-Signaling
Trans-Signaling
IL-11
sIL-11Rα
gp130
IL-11Rα
sIL-11Rα
extracellular
intracellular
STAT3
+VEGF
pERK
pSTAT1
antiangiogenic
pro-angiogenic
pro-angiogenic effect remained
C
STAT1 knockdown in HUVECs
Cis-Signaling
Trans-Signaling
pSTAT3 Tyr705
STAT1
pSTAT5
antiangiogenic effect remained
Co-stimulation with VEGF: attenuation of proangiogenic effect

Supplemental Figure S4: STAT3 and STAT1 knockdown

(A) Semi-quantitative Western blot analysis of pSTAT5 in HUVECs after STAT3 knockdown and stimulation with EBM, IL-11, VEGF and IL-11+VEGF or IL-11+sIL-11Rα and IL-11+sIL-11Rα+VEGF for 15 min. Interleaved box and whiskers: the whiskers represent the minimum and maximum values while the line in between represents the median value. N = 3 independent experiments.

(B) Graphical summary of altered signaling pathways in IL-11 cis- and trans-signaling following STAT3 knockdown and its resulting angiogenic phenotype.

(C) Graphical summary of changed signaling patterns by cis- and trans-signaling following STAT1 knockdown and its resulting angiogenic phenotype.

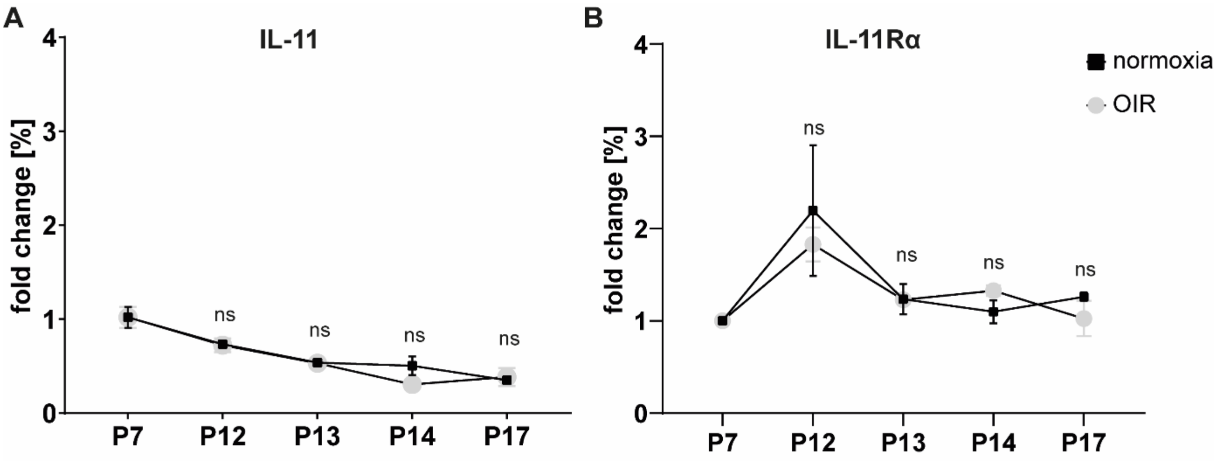

Supplemental Figure S5: IL-11 and sIL-11Rα levels in the OIR model

(A) Changes in IL-11 expression levels under normoxic and oxygen-induced retinopathy (OIR) conditions at postnatal days 12, 13, 14, and 17. N = 2 – 4 mice per time point. Statistical test: Statistical test: Kruskal-Wallis Test adjusted for multiple testing.

(B) Changes in IL-11Rα expression levels under normoxic and oxygen-induced retinopathy (OIR) conditions at postnatal days 12, 13, 14, and 17. N = 3 – 4 mice per time point. Statistical test: Kruskal-Wallis Test adjusted for multiple testing.

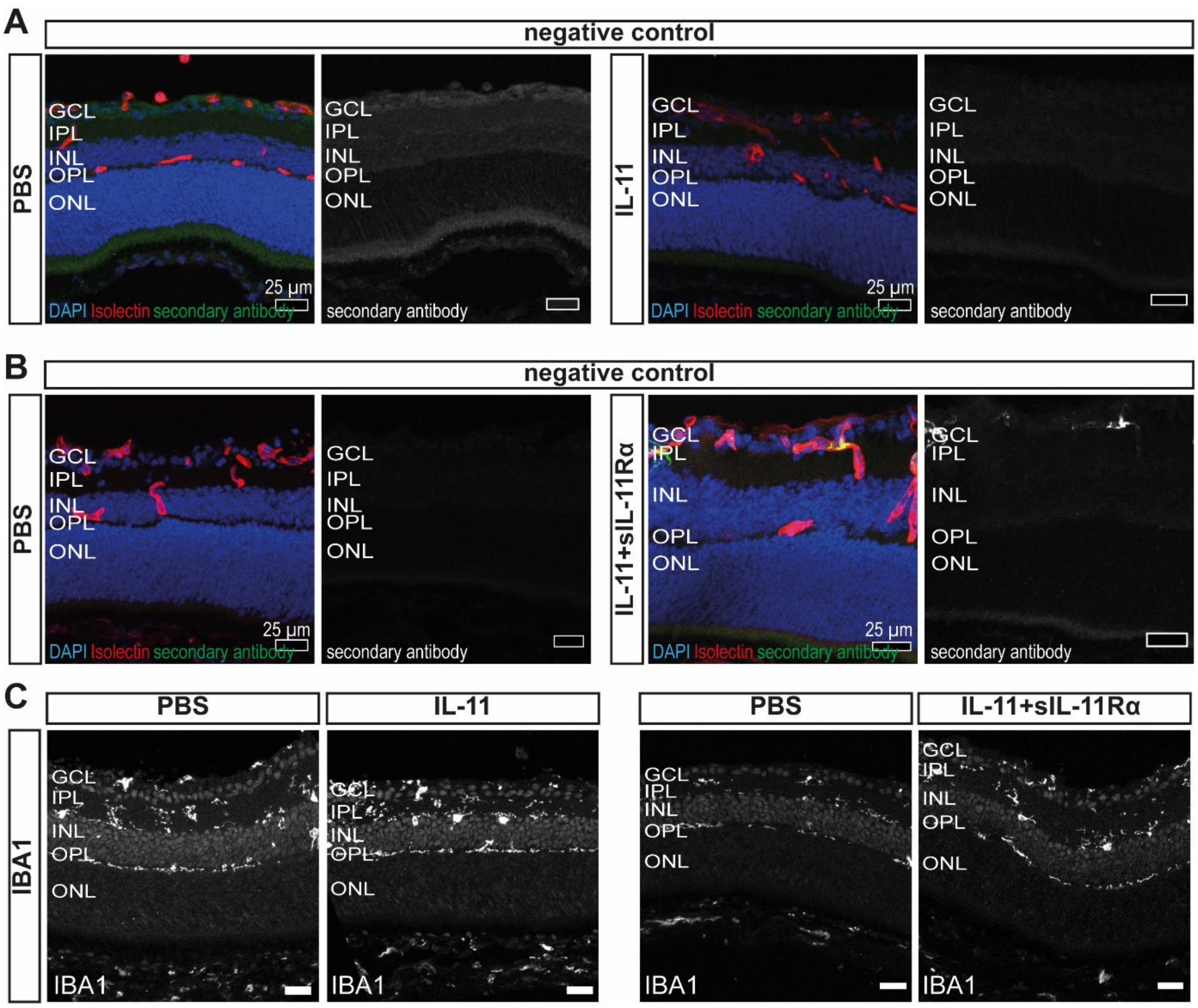

Supplemental Figure S6: Negative control and IBA1 staining of retinal cryosections

(**A**) Representative images of retinal cryosections of C57BL/6J mice 12 h after injection with IL-11 or PBS control at OIR P12. Staining was performed with the secondary antibody only. Scale bar: 25 µm.

(B) Representative images of retinal cryosections of C57BL/6J mice 12 h after injection with IL-11+sIL-11Rα or PBS control at OIR P12. Staining was performed with the secondary antibody only. GCL = ganglion cell layer, IPL = inner plexiform layer, INL = inner nuclear layer, OPL = outer plexiform layer, ONL = outer nuclear layer. Scale bar: 25 µm.

(C) Representative images of retinal cryosections of ALDH1L1-GFP^{-} transgenic mice for IBA1 12 hours after injection with IL-11, IL-11+sIL-11Rα or PBS control at OIR P12. GCL = ganglion cell layer, IPL = inner plexiform layer, INL = inner nuclear layer, OPL = outer plexiform layer, ONL = outer nuclear layer. Scale bar: 25 µm.

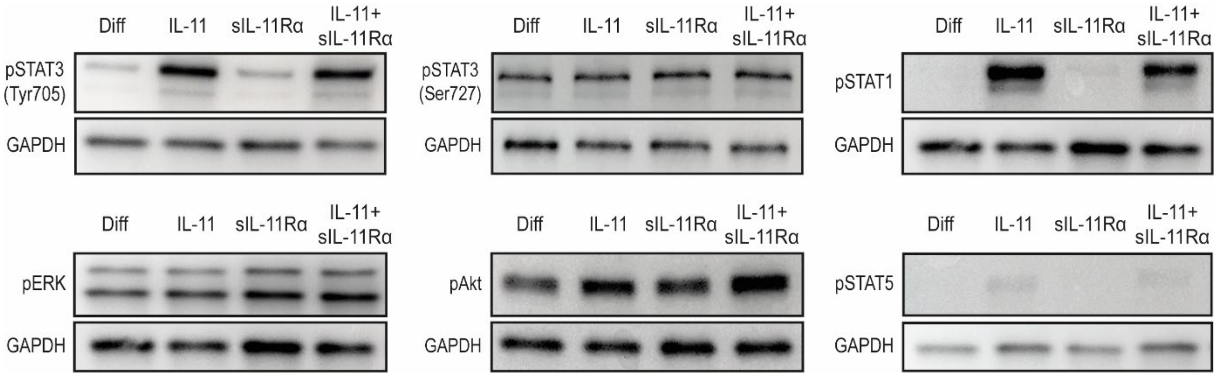

Supplemental Figure S7: Activation of signaling pathways in Müller cells without VEGF co-stimulation

Western blot screening for the following possible activated signaling molecules: pSTAT3 Tyr705, pSTAT3 Ser727, pSTAT1, pERK, pAkt, pSTAT5. Müller cells were stimulated with Diff (negative control), mIL-11, sIL-11Rα or mIL-11+sIL-11Rα for 15 min. N = 3 independent experiments.
