# Supplementary figures and images for: Characterization of the angiomodulatory effects of Interleukin 11 cis- and trans-signaling in the retina
Source: J Neuroinflammation. 2024 Sep 18;21:230. doi: 10.1186/s12974-024-03223-3 (PMC11412048; doi:10.1186/s12974-024-03223-3)

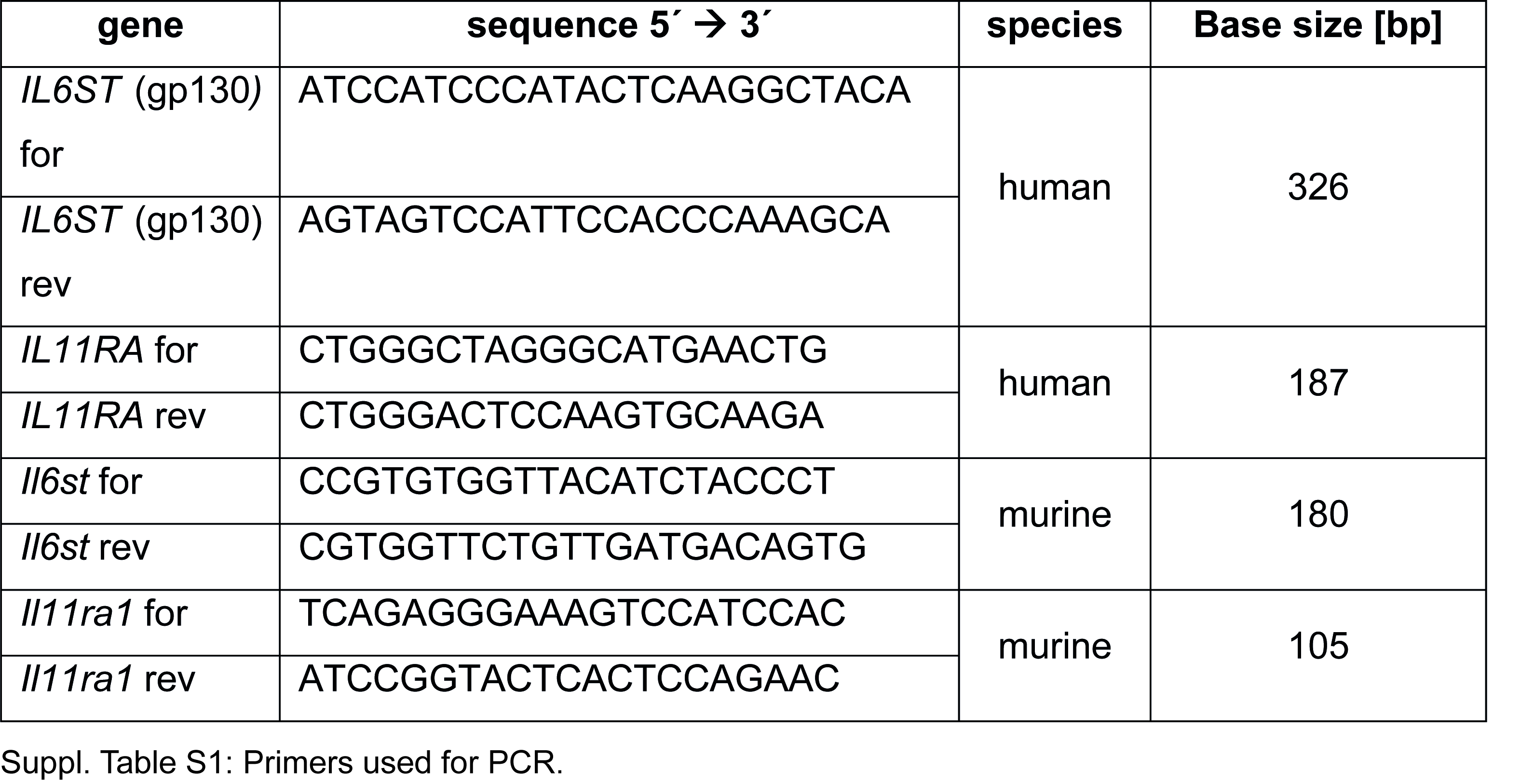

Supplement: Supplementary file 3 — Supplementary Material 3 [file 12974_2024_3223_MOESM3_ESM.tif]

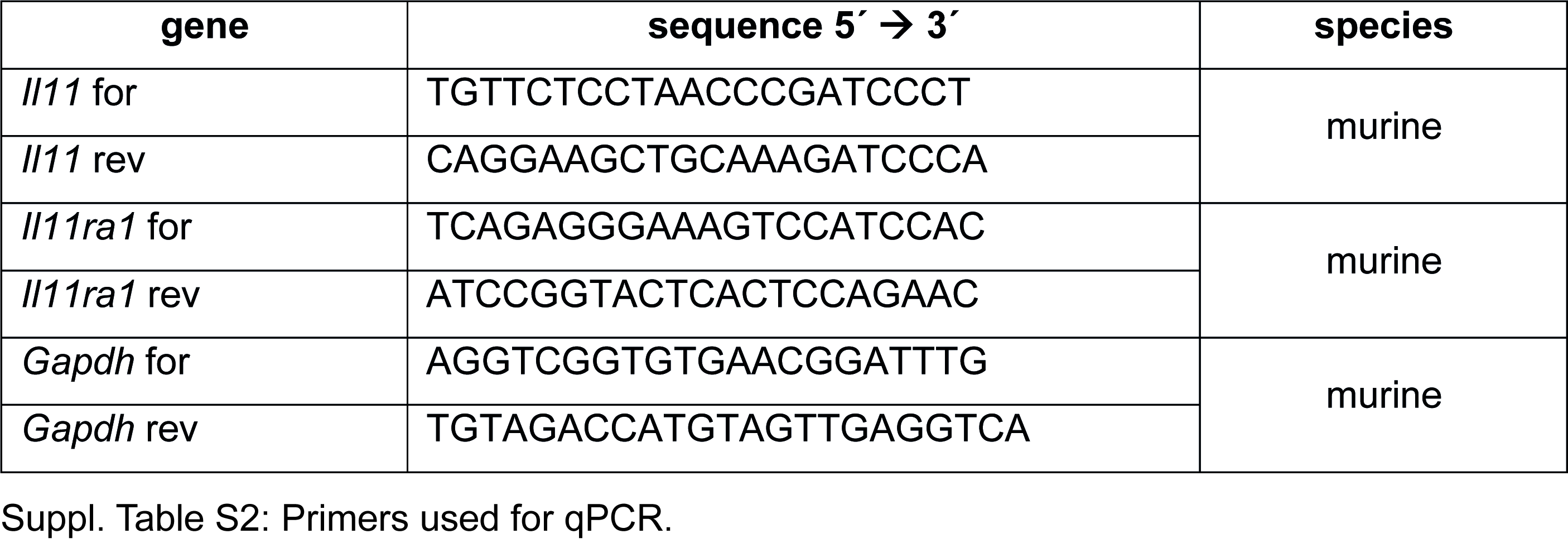

Supplement: Supplementary file 4 — Supplementary Material 4 [file 12974_2024_3223_MOESM4_ESM.tif]
